# Recent expansion of metabolic versatility in *Diplonema papillatum*, the model species of a highly speciose group of marine eukaryotes

**DOI:** 10.1186/s12915-023-01563-9

**Published:** 2023-05-04

**Authors:** Matus Valach, Sandrine Moreira, Celine Petitjean, Corinna Benz, Anzhelika Butenko, Olga Flegontova, Anna Nenarokova, Galina Prokopchuk, Tom Batstone, Pascal Lapébie, Lionnel Lemogo, Matt Sarrasin, Paul Stretenowich, Pragya Tripathi, Euki Yazaki, Takeshi Nara, Bernard Henrissat, B. Franz Lang, Michael W. Gray, Tom A. Williams, Julius Lukeš, Gertraud Burger

**Affiliations:** 1 Department of Biochemistry, Robert-Cedergren Centre for Bioinformatics and Genomics, Université de Montréal, Montreal, QC Canada; 2 School of Biological Sciences, University of Bristol, Bristol, UK; 3 Institute of Parasitology, Biology Centre, Czech Academy of Sciences, České Budějovice, Czech Republic; 4 Faculty of Science, University of South Bohemia, České Budějovice, Czech Republic; 5 Faculty of Science, University of Ostrava, Ostrava, Czech Republic; 6 Present address: High Performance Computing Centre, Bristol, UK; 7 Architecture et Fonction des Macromolécules Biologiques (AFMB), CNRS, Aix Marseille Université, Marseille, France; 8 Present address: Environment Climate Change Canada, Dorval, QC Canada; 9 Present address: Canadian Centre for Computational Genomics; McGill Genome Centre, McGill University, Montreal, QC Canada; 10 RIKEN Interdisciplinary Theoretical and Mathematical Sciences Program (iTHEMS), Hirosawa, Wako, Saitama Japan; 11 Laboratory of Molecular Parasitology, Graduate School of Life Science and Technology, Iryo Sosei University, Iwaki City, Fukushima, Japan; 12 Present address: DTU Bioengineering, Technical University of Denmark, Lyngby, Denmark; 13 Department of Biological Sciences, King Abdulaziz University, Jeddah, Saudi Arabia; 14 Department of Biochemistry and Molecular Biology, Institute for Comparative Genomics, Dalhousie University, Halifax, NS Canada

**Keywords:** *Paradiplonema papillatum*, Protists, Genome, Transcriptome, Proteome, Gene-family evolution, Lateral gene transfer, CAZymes, Feeding strategy, Geographical distribution, Ecological distribution

## Abstract

**Background:**

Diplonemid flagellates are among the most abundant and species-rich of known marine microeukaryotes, colonizing all habitats, depths, and geographic regions of the world ocean. However, little is known about their genomes, biology, and ecological role.

**Results:**

We present the first nuclear genome sequence from a diplonemid, the type species *Diplonema papillatum*. The ~ 280-Mb genome assembly contains about 32,000 protein-coding genes, likely co-transcribed in groups of up to 100. Gene clusters are separated by long repetitive regions that include numerous transposable elements, which also reside within introns. Analysis of gene-family evolution reveals that the last common diplonemid ancestor underwent considerable metabolic expansion. *D. papillatum*-specific gains of carbohydrate-degradation capability were apparently acquired via horizontal gene transfer. The predicted breakdown of polysaccharides including pectin and xylan is at odds with reports of peptides being the predominant carbon source of this organism. Secretome analysis together with feeding experiments suggest that *D. papillatum* is predatory, able to degrade cell walls of live microeukaryotes, macroalgae, and water plants, not only for protoplast feeding but also for metabolizing cell-wall carbohydrates as an energy source. The analysis of environmental barcode samples shows that *D. papillatum* is confined to temperate coastal waters, presumably acting in bioremediation of eutrophication.

**Conclusions:**

Nuclear genome information will allow systematic functional and cell-biology studies in *D. papillatum*. It will also serve as a reference for the highly diverse diplonemids and provide a point of comparison for studying gene complement evolution in the sister group of Kinetoplastida, including human-pathogenic taxa.

**Supplementary Information:**

The online version contains supplementary material available at 10.1186/s12915-023-01563-9.

## Background

Diplonemids are heterotrophic, flagellated, unicellular eukaryotes. Overlooked for decades, they have recently been characterized as the most species-rich group of known marine protists [1, 2]. Global metabarcoding surveys have estimated at least 67,000 species [3], revealing that diplonemids populate not only all biogeographic and pelagic zones of the oceans [4, 5], but also thrive in anoxic zones [3] and dominate deep-sea sediments [6]. Diplonemids inhabit fresh water as well, but in moderate abundance and diversity, suggesting recent habitat transitions [7].

Due to their abundance, distribution, and diversity, diplonemids are thought to play an important role in the marine food web. However, we have very little data regarding their nutrition. Views about their predominant feeding strategy are controversial, ranging from parasitism [6] to epibiosis of water plants and invertebrates, to predation of diverse algae including diatoms and dinoflagellates, to saprotrophy [8, 9]. In addition, a few diplonemid species seem to be bacterivorous [10, 11]. New research also indicates that diplonemids may significantly contribute to the cycling of certain heavy metals [12], though the actual extent and relevance for the marine ecosystem remains to be determined.

**Fig. 1:**
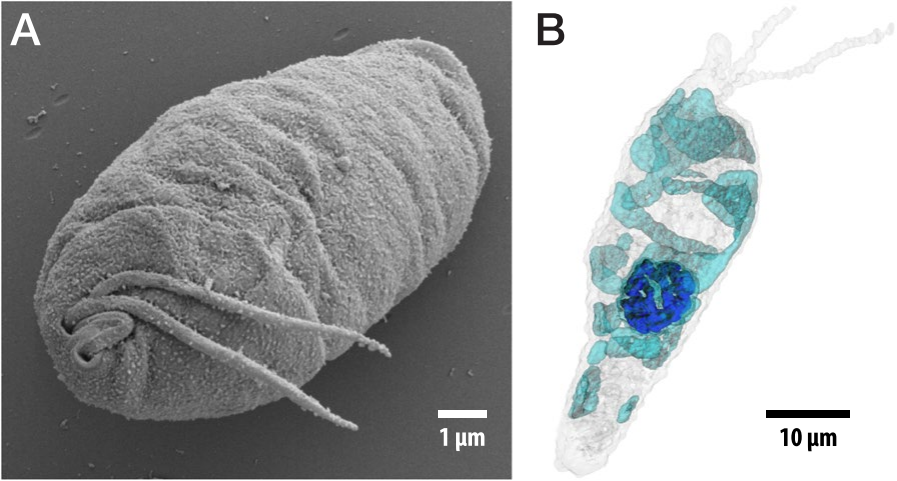
Morphology and ultrastructure of *D. papillatum*. **A** Scanning electron microscopy image. The anterior end of the cell exposes the two flagella emanating from the flagellar pocket (left), the cytopharynx (right), and the conspicuous lip-like papillum between these two openings. Measurements taken from this image (using the ImageJ software): cell length > 10.5 μm (the exact size cannot be measured from this image because the cell does not lie flat); cell width ~ 5.9 μm; flagella length 6.5 μm; and cytostome width 0.31 μm. **B** Expansion microscopy-based model showing the nucleus and mitochondrion of a typical cell. Light gray, cell-surface tubulin; cyan, reticulated mitochondrion; blue, reticulated nuclear heterochromatin. The three-dimensional model was built from the Z-stack series of images after staining with DAPI and anti-tubulin antibodies. For details see Additional file 1: Sect. 1. Physical structure and size of the *Diplonema papillatum* nuclear genome

Diplonemids (Diplonemea) are subdivided into four monophyletic lineages, the classical diplonemids (Diplonemidae), hemistasiids (Hemistasiidae), and the Deep-*s*ea pelagic diplonemid clades I and II (DSPDI and II) [13], the former now classified as Eupelagonemidae [14]. Currently, about nine diplonemid genera comprising nearly two dozen species are formally recognized and morphologically characterized [15]. However, axenic cultures have been established for only a handful of species that mostly belong to the Diplonemidae [16, 17], including the type species *Diplonema papillatum* (Fig. 1) (alternatively referred to as *Paradiplonema papillatum* [18]), whose genome is described here. From the Eupelagonemidae, the ecologically most prominent diplonemid group, just a few cells have been examined by microscopy and single-cell sequencing, while the vast majority of taxa is only known from environmental barcoding surveys [1, 19, 20].

In global eukaryotic phylogenies, diplonemids are placed together with euglenids, kinetoplastids, and symbiontids within the phylum Euglenozoa. Diplonemids form the sister group to kinetoplastids, which include the human pathogens *Trypanosoma* and *Leishmania* alongside several free-living taxa (e.g., *Bodo saltans*). Euglenozoa belong to the deeply diverging eukaryotic supergroup Discoba [21], which differs in essentially all aspects of biology from the familiar and best-studied eukaryotes—animals, fungi, and plants.

Diplonemids have also attracted interest because of their highly unusual mitochondrial genome, which has been most intensively investigated in *D. papillatum*. In contrast to conventional mitochondrial DNAs, that of *D. papillatum* not only makes up an overwhelming portion of cellular DNA [22], but also consists of hundreds of small chromosomes, each of which carries a single gene piece. Consequently, the assembly of full-length mRNAs and ribosomal RNAs (rRNAs) requires a range of RNA ligation events, which are accompanied by RNA editing [23, 24]. Studies across Diplonemidae and Hemistasiidae have revealed similar mitochondrial gene fragmentation and RNA editing features as seen in *D. papillatum*, reaching unprecedented degrees in certain species [25–27].

Establishing a diplonemid model system required not only a reasonably fast-growing axenic culture but also procedures to genetically modify the corresponding organism. In the past few years, we have developed protocols to transform *D. papillatum* with exogenous DNA [28] leading to homologous integration, which allows efficient knock-ins of tagged genes [29]. Now we have a powerful toolbox at hand for efficiently investigating the cellular and molecular biology of *D. papillatum*.

Available genome and transcriptome data from diplonemids are currently limited, with single gene and partial single-cell genome sequences generated for phylogenies [30, 31], biodiversity studies [19, 32, 33], or the investigation of metabolic adaptations in certain diplonemids [34, 35]. While suited for the questions addressed in the corresponding studies, the data are too sparse to provide insight into the functions encoded in and the broader evolution of diplonemid nuclear genomes.

Here we present the nuclear genome and transcriptome sequence of *D. papillatum*. In addition to serving as a reference sequence for the diplonemids as a whole, our data provide insight into complex gene structures and expression modes. Analysis of the gene repertoire reveals a diverse metabolic potential of *D. papillatum*, but also, for a euglenozoan, unexpected conservatism of certain basic molecular machineries such as the cytosolic ribosome. Comparative genomics demonstrates that genes and pathways involved in carbohydrate degradation have undergone a major evolutionary expansion in diplonemids. The inferred metabolism, backed by feeding experiments, support the view that *D. papillatum* is extraordinarily versatile in using diverse carbon sources from myco-, phyto-, and zooplankton, as well as marine water plants. Taken together, our experiments and comparative genomic analyses strongly suggest that diplonemid protists play a crucial and previously unrecognized role in the food web of aquatic environments.

## Results and discussion

### Genome assembly, genome size, and ploidy

We generated ~ 900 million short paired-end reads (Illumina) and ~ 700,000 long reads (PacBio) summing to 187 Gbp. Reads were assembled into 6181 contigs ≥ 200 bp long totaling 280,293,864 bp, with an N50 value of 190,080 bp and a maximum contig length above 1 Mbp. The completeness of the assembly is estimated to be above 95%. Although BUSCO benchmarking [36] of conceptually translated *D. papillatum* proteins against the set of highly conserved eukaryotic core proteins recovered only 89%, we determined that more than half of the proteins reported as missing are too divergent to fall within BUSCO’s inclusion threshold, and that a quarter are absent from all diplonemids examined (Additional file 1: Sect. 2. Assembly and annotation of the nuclear genome and transcriptome of *Diplonema papillatum*). We therefore consider the *Diplonema* genome assembly as *quasi* complete.

Genome assemblies are typically larger than the genome because of repeats. The actual nuclear genome size of *D. papillatum* was calculated by various methods. The estimate of 260 Mbp, based on k-mer frequencies in short reads, is deemed most accurate because this procedure is least affected by artifacts (Additional file 1: Sect. 3. The ploidy level of *Diplonema papillatum*). Assessment of the *Diplonema* nuclear genome size by pulsed-field gel electrophoresis was inconclusive as it yielded numerous unresolved molecules of length 1.1 to 1.8 Mbp, with only two distinct bands at 0.5 and 1.0 Mbp. It appears that the genome consists of hundreds of similarly sized chromosomes. A clear separation of individual chromosomes is probably impeded by the complex reticulated nuclear DNA structure observed by ultrathin-section and fluorescence microscopy of the *Diplonema* nucleus (Additional file 1: Sect. 1. Physical structure and size of the *Diplonema papillatum* nuclear genome).

The ploidy estimation of the *D. papillatum* nuclear genome is based on the frequency and distribution of k-mers in reads and single-nucleotide variants (SNVs) in the assembly. The extremely low SNV frequency (less than 600 sites in the 142 Mbp repeat-free genome portion) of the *Diplonema* genome and a symmetric, bell-shaped k-mer distribution frequency of short reads suggest haploidy (Additional file 1: Sect. 3. The ploidy level of *Diplonema papillatum*). While it is not possible to distinguish haploids from homozygous diploids (or higher ploidy levels) with computational methods alone, the most convincing confirmation for haploidy comes from gene replacement experiments, in which the transformation with engineered gene versions resulted reproducibly in single alleles [28, 29].

It should be noted that our assessment of haploidy refers to the standard laboratory *D. papillatum* strain, where the exclusive form of reproduction appears to be mitosis. Although sexual reproduction or a diploid stage has not yet been observed, the gene repertoire implies that *D. papillatum* has the potential to form diploid zygotes that undergo meiosis (Additional file 1: Sect. 11. Meiosis in *Diplonema papillatum*?).

### Genome annotation and quality assessment

Genome annotation was performed by a pipeline developed in-house, combining gene model prediction with evidence-based and ab initio gene prediction (Additional file 1: Sect. 2. Assembly and annotation of the nuclear genome and transcriptome of *Diplonema papillatum*). Evidence-based prediction of protein-coding regions was guided by curated SwissProt sequences and unreviewed Discoba sequences available from public data repositories, as well as assembled *D. papillatum* transcripts. The start of the 5′ *U*n*T*ranslated *R*egion (UTR) was positioned at the site at which a Spliced Leader (SL) is added to pre-mRNA by trans-splicing—a feature shared by all Euglenozoa [37, 38].

The completeness and quality of automatically predicted protein-coding gene models were assessed by expert inspection of the three largest contigs in the assembly. Together these contigs represent 1% of the total assembly and contained initially 319 gene models. By scrutinizing the coverage of RNA-Seq reads and transcripts assembled from these reads in the corresponding genome regions, we detected that 15 genes lacked corresponding models, 125 models had inaccurate gene structures, and 68 were false positives. Although the error rate appears high, it compares favorably with current automated annotations [39]. In *Diplonema*, most of the omitted or erroneous gene predictions are a consequence of the highly repetitive genome sequence in this organism as detailed below.

In sum, while the automated annotation procedure predicted ~ 37,000 protein-coding genes, the false positive and negative rates observed during manual curation indicate that their actual number in the *D. papillatum* nuclear genome assembly version 1.0 is rather ~ 32,000. *Diplonema*’s protein-coding genes contain on average 1.6 introns. Alternative splicing is estimated at 5% among multi-exonic genes, a proportion that is low compared to multicellular eukaryotes such as human (60%) or *Arabidopsis* (20%) [40], but in the range reported for other unicellular organisms [41].

Functional information was assigned to about 51% of the predicted protein-coding genes with an explicit molecular function available for about 35% of the models, and a conserved Pfam protein domain for an additional 15%. As in many other organisms, approximately 50% of the predicted protein-coding genes in *Diplonema* lack any indication as to their function.

### Nuclear gene structure

About 41% of the protein-coding genes in the *D. papillatum* nuclear genome assembly contain introns, the large majority of which are canonical, bearing GT at their 5′-end and AG at the 3′-end (GT-AG type) (Additional file 1: Sect. 4. Intron splicing and structural RNAs). A few non-canonical introns with GC-AG splice-site combinations were detected as well. It was shown for GC-AG introns from animals, fungi, and plants that these introns are typically spliced by the same major U2 spliceosome as GT-AG introns [42]. The generally rare AT-AC (U12-type) spliceosomal introns seem to be absent from *Diplonema*, which is consistent with the lack of the U4atac, U6atac, U11, and U12 RNAs among the set of spliceosomal RNAs identified in this organism. Moreover, we did not detect unconventional introns such as the ones present in *Euglena* that lack conserved splice boundaries, have extensive base pairing to bring intron ends together, and are apparently removed in a spliceosome-independent fashion ([43] and references therein). Certain non-classified diplonemids reportedly possess *Euglena*-like introns; however, in the absence of transcriptome data, this inference cannot be validated [19].

While the median size of *Diplonema* introns is below 1 kbp, a small percentage are considerably longer, often comprising complete or partial transposable elements with several open reading frames (ORFs) (see following section). The longest expert-validated intron is 72 kbp in size and resides in the gene DIPPA_22195, predicted to encode a protein with a conserved kinesin-motor domain. This is the largest known *Diplonema* gene (186 kbp), containing the highest number (20) of introns as confirmed by expert validation. It is noteworthy that several genes with confirmed alternative splicing combine more than one splicing mode. For example, the expression of DIPPA_03285 involves occasional exon skipping, intron retention, and alternative splice-site selection. The corresponding protein sequence has moderate similarity with the Pfam domain TFIIα (Transcription initiation factor II alpha) and a common structural domain called PDZ found in numerous cell-signaling proteins.

In all domains of life, the coding regions of genes are usually bounded by untranslated regions. At ~ 70 bp, the 5′-UTRs of *Diplonema* nuclear protein-coding genes are within the size range commonly observed across eukaryotes (Additional file 1: Sect. 5. Untranslated regions of nuclear genes). In contrast, the observed 3′-UTRs are exceptionally large; they sometimes extend up to several kbp and have a median size of ~ 800 bp, which is two to ten times longer than in other eukaryotes. In diplonemid’s sister group, the kinetoplastids, the 3′-UTR gene region is known to play a predominant role in the regulation of gene expression, in particular by controlling mRNA translation and decay rates [44]. Therefore, the long 3′-UTRs of *D. papillatum* genes may serve as a binding platform for numerous regulatory proteins. It would be worthwhile to investigate the identity of these postulated RNA-binding proteins experimentally, with those *Diplonema* genes possessing the longest 3′-UTRs presenting the most obvious first targets.

**Fig. 2:**
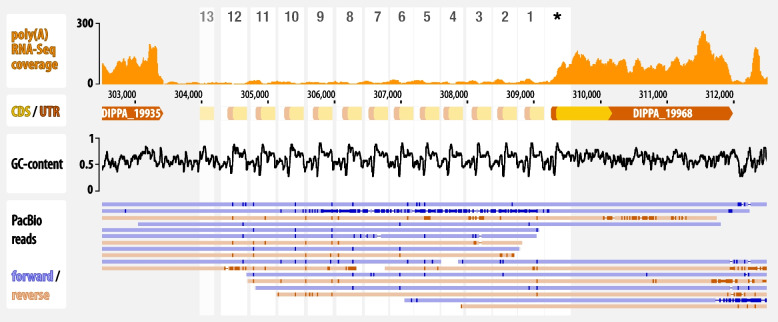
Repeat-bounded gene structures in the *D. papillatum* nuclear genome. An example of a gene with a terminal region repeated multiple times adjacent to the expressed portion of the gene. DIPPA_19968 encodes a SufC homolog, a protein involved in iron-sulfur cluster synthesis. The 5′-terminal segment, including the 5′-UTR and part of CDS, is repeated 13 times upstream of the transcribed gene portion. Copies #1–#12 display 71–96% sequence identity, while the most distal repeat has only 47%. Middle pane: the G + C content plot reflects the repetitiveness of the region. Lower panel: long reads covering this region confirm the correctness of the genome assembly in this region

During expert validation of the structural annotation of the assembly (Additional file 1: Sect. 2. Assembly and annotation of the nuclear genome and transcriptome of *Diplonema papillatum*), we identified dozens of gene models with adjacent sequence repeats. In many of these instances, a portion of the gene’s 5′-region, including parts of the 5′-UTR and coding sequence (CDS), is repeated in tandem. In other cases, the 3′-end of the first exon is repeated, forming a part of the first intron or—if the gene consists of a single exon—the 3′-UTR. The longest repeated gene extension was detected upstream of DIPPA_19968 encoding an ABC transporter. Here, a ~ 400-bp long sequence motif composed of a part of the gene’s 5′-UTR and the preceding intergenic region occurs in 12 tandemly arranged, degenerate copies, constituting a tandem array of nearly 5 kbp. PacBio reads support the assembly in this genome region, and RNA-Seq-read coverage indicates that the tandem array is not part of the mature mRNA (Fig. 2). Obviously, repeats adjacent to genes interfere with automated structural annotation, because RNA-Seq-reads can be aligned to multiple locations, occasionally resulting in gene models that are too long or include spurious introns.

### Non-coding regions and repeats

Nuclear genomes of free-living Discoba, for which near-complete assemblies are available, are all below 50 Mbp in size, with 20 Mbp for *Andalucia godoyi* (Jakobida) [45], 40 Mbp for *B. saltans* (Kinetoplastida) [46], and 41 Mbp for *Naegleria gruberi* (Heterolobosea) [47]. With the exception of the *E. gracilis* genome, estimated at 330–500 Mbp [48], the *D. papillatum* nuclear genome is 6–13 times larger than those known from other discobids. Introns contribute to some extent to this genome size difference, but the additional material mainly comprises repeat regions—mostly dispersed repeats*—*which make up 52% of the assembly (Additional file 1: Sect. 6. Repetitive sequences in the nuclear genome of *Diplonema papillatum* (assembly v_1.0); Additional file 2). Among the nearly 10,000 distinct dispersed repeat motifs, the most abundant one occurs about 6000 times when considering copies of ≥ 90% sequence identity. The longest, nearly 20-kbp dispersed repeat motif, which is found 13 times in the genome, is particularly notable because it is itself composed of an array of approximately eight 2.5 kbp-long motifs arranged in tandem. Each of these tandem repeats contains a 283-amino acid-long ORF that is apparently not transcribed, nor does it share similarity to proteins or conserved domains in public databases.

While many dispersed repeat units in the *Diplonema* nuclear genome have no obvious origin or function, others derive from transposable elements and encode proteins known from retrotransposons and DNA transposons described for a wide range of eukaryotes. The *Diplonema* nuclear genome assembly includes as many as ~ 2500 gene models annotated as retrovirus-related polyproteins, LINE-1 (Long interspersed nuclear element) ORFs, SLACS (Spliced-leader-associated-conserved sequence) reverse transcriptases, and DNA-directed RNA polymerase from mobile element R2 and jockey. In addition, more than 60 ORFs from *Diplonema* resemble proteins residing in DNA transposons including TATE (Telomere associated transposable element), MULE (mutator-like element), and Helitron [49]. A rigorous identification of transposable elements including non-coding regions will be warranted once a chromosome-scale genome assembly becomes available to eliminate artificially duplicated or collapsed repeat regions.

As expected, certain dispersed repeat units contain regular genes, one of which is the ribosomal DNA (rDNA) cluster that is composed of 18S, 5.8S, and 28S rRNA genes. The *Diplonema* nuclear genome assembly contains 13 copies of this cluster at ≥ 99% sequence identity. In addition to dispersed copies of complete genes, we also found multiple copies of gene fragments. For example, about 3600 28S rRNA gene pieces (up to 10% in length of the complete gene) are scattered throughout the genome. The nuclear genomes of human and other eukaryotes carry similar repeats that are referred to as terminal-repeat retrotransposons in miniature (TRIMs) and short interspersed elements (SINEs), but which contain gene portions of 5S rRNA and 28S rRNA [49].

Another source of extra sequence in the *Diplonema* nuclear genome are nuclear mitochondrial segments (NUMTs), i.e., portions of the mitochondrial genome [23, 24, 50] incorporated into the nuclear DNA, and which make up at least 343 kbp (1.2%) of the assembly (Additional file 1: Sect. 6. Repetitive sequences in the nuclear genome of *Diplonema papillatum* (assembly v_1.0)). We detected more than 1400 NUMTs (> 100 bp), including 11 complete mitochondrial chromosomes. NUMTs are inserted predominantly in intergenic regions, but ~ 20% occur in introns and UTRs of nuclear genes (Additional file 2). Nearly 2% of NUMTs are arranged in tandem. The longest array of nearly 10 kbp consists of 164 copies of a 68-bp stretch from the B-class constant region of mitochondrial chromosomes. The total length and proportion that NUMTs contribute to the *Diplonema* nuclear genome compares with the situation in animals and plants [51–53].

### Transcription, transcript maturation, and regulation of gene expression

Protein-coding genes in the *D. papillatum* genome assembly are conspicuously arranged in clusters with genes sharing the same transcriptional orientation (Additional file 1: Sect. 7. Polycistronic transcription units in the nuclear genome of *Diplonema papillatum*). Nearly 90% of all contigs larger than 50 kbp include unidirectional arrays of five up to 120 genes. The longest expert-validated gene cluster (in the contig tig00022654_12, which is 1,009,103 bp long) comprises as many as 108 genes. Inside clusters, genes are not particularly tightly spaced. For example, in tig00022654, several intergenic regions are longer than 10 kbp (Additional file 3). This gene arrangement is reminiscent of trypanosomes, where arrays of about 100 unidirectional genes are co-transcribed into several long primary polycistronic RNAs [54].

As already mentioned above, mRNAs of *Diplonema* and other Euglenozoa carry a spliced-leader (SL) sequence extension at their 5′-terminus that is encoded by a separate gene, transcribed independently and added by trans-splicing to pre-mRNAs [55]. Extrapolating from the set of expert-validated genes, essentially all mRNAs in *D. papillatum* carry an SL at their 5′ terminus (Additional file 1: Sect. 2. Assembly and annotation of the nuclear genome and transcriptome of *Diplonema papillatum*), strongly suggesting that the maturation of clustered genes proceeds as in kinetoplastids, involving the processing of long polycistronic RNAs to monocistronic units along with the posttranscriptional addition of an SL to the 5′-end [56].

A predominant co-transcription of *D. papillatum* nuclear genes implies that in contrast to most other eukaryotic groups, gene expression—probably in euglenozoans as a whole—is not primarily regulated by transcription initiation. Our finding of genes involved in DNA modification and transcript degradation points to alternative, gene-specific control mechanisms acting in the *Diplonema* nucleus. First, *D. papillatum* has the potential for synthesizing nucleobase J and 5mC, both reported to play an important role in gene regulation of model organisms (Additional file 1: Sect. 8. DNA modifications (5mC and J)). Base J (β-D-glucopyranosyl-oxymethyluracil) is a hyper-modified thymine derivative, which was detected early on in the nuclear DNA of Euglenozoa [57]. Its role in transcription termination has been demonstrated in trypanosomes [58]. The *D. papillatum* genome encodes counterparts of all proteins participating in the biosynthesis and proliferation of this nucleotide modification.

Similarly, we identified homologs of DNA methyltransferase genes known to synthesize 5-methyldeoxycytosine (*5mC*) in the *Diplonema* genome. This epigenetic mark mediates transcriptional repression, particularly of transposons and other repetitive elements in nuclear genomes of animals, plants, and fungi [59]. The presence of a dozen homologs of AlkB-type genes encoding oxidative demethylases in the *Diplonema* genome indicates that this organism uses methylation/demethylation to dynamically regulate gene expression.

Further, *D. papillatum* has the potential for RNA interference (RNAi) (Additional file 1: Sect. 9. RNA interference (RNAi)). We retrieved from the inferred proteome homologs of all components required for a functional RNAi pathway, two Dicer-like proteins, five Piwi proteins, three members of the Argonaute family, and one RNA-dependent RNA polymerase. In model organisms, RNAi has been shown to control RNA degradation and translational silencing of transposable elements and genuine nuclear genes [60, 61]. Key determinants of the RNAi machinery are also encoded in the nuclear genome of *Euglena gracilis* [62], but are incomplete or missing in many (but not all) kinetoplastid taxa [63].

**Fig. 3:**
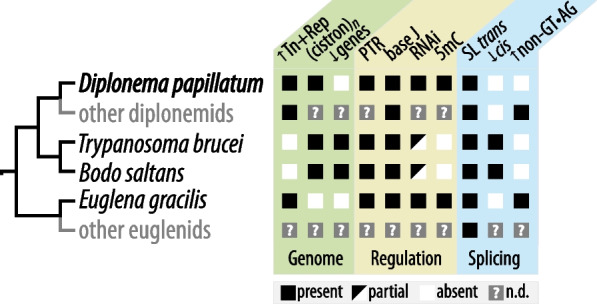
Comparison of genome and gene expression features across Euglenozoa. ↑Tn + Rep, conspicuous abundance of transposons and repetitive sequences; (cistron)_*n*_, polycistronic transcription; ↓genes, streamlined gene repertoire; PTR, posttranscriptional regulation of gene expression; base J, base J present in nuclear DNA; RNAi, RNA interference pathway; 5mC, 5-methyldeoxycytosine pathway; SL *trans*, spliced-leader *trans*-splicing; ↓*cis*, few cases of *cis*-splicing; ↑non-GT•AG, conspicuous abundance of unconventional *cis*-introns. n.d., not determined

Figure 3 summarizes the current knowledge of the shared and particular features related to gene expression as well as genome architecture across the euglenozoans.

### Structural RNA genes

The *D. papillatum* nuclear genome contains a total of ~ 37,000 genes, of which about 1000 encode structural RNAs, also referred to as non-(protein-)coding RNAs (ncRNAs).

Four rRNA species are associated with the cytosolic (cyto) ribosomes of *D. papillatum*, notably 28S, 5.8S, and 5S-sized rRNAs in the large subunit (LSU), and 18S rRNA in the small subunit (SSU) (Additional file 1: Sect. 4. Intron splicing and structural RNAs). We confirmed this number experimentally, because it is atypically low for cytoribosomes from euglenozoans. For example, the 28S rRNA is split into six or more pieces across kinetoplastids and euglenids [64, 65]. Despite the difference in the number of rRNA species, *Diplonema* cytoribosomes contain the same complement of canonical cytoribosomal proteins as kinetoplastids and euglenids, as well as one SSU ribosomal protein apparently unique to euglenozoans (Additional file 1: Sect. 10. The cytosolic ribosome of *Diplonema papillatum*). As in other eukaryotes, the *Diplonema* genome carries three of the rRNAs (28S, 5.8S, and 18S rRNAs) organized in a classical rDNA tandem unit. The genome assembly includes more than 20 of these ~ 7.6 kbp-long rDNA units arranged in clusters, but the actual length of these repeat arrays is not known. The 5S rRNA is not included in the rDNA repeat unit but rather in a separate repeat unit together with SL RNA, as discussed below.

The nuclear genome assembly contains a set of 211 high-scoring transfer RNA (tRNA) genes comprising up to 10 identical copies (tRNA-Lys_CUU_), with some carrying an intron in the anticodon loop. Collectively, this ensemble of tRNA genes represents 46 out of 64 possible anticodons. Among the missing anticodons are all those reported absent from eukaryotes in general [66], but one missing specifically from *Diplonema* is that of tRNA-Leu_UAA_ for decoding TTA codons. While this leucine codon is the one used most infrequently (2%) in protein-coding regions of *D. papillatum*, it does occur in vital genes. Assuming posttranscriptional modification of bases in the anticodon, UUA could be read by the anticodon of tRNA-Leu_CAA_ after conversion of the wobble cytosine to uracil or, alternatively, by the tRNA-Leu_AAG_ anticodon after deamination of adenine-34 to inosine. The genes from *Diplonema* that could catalyze such base-modification activities are the homologs of ADAT2 and ADAT3 encoding the two-component A-to-I tRNA editing enzyme known from other eukaryotes [67]. It is possible that in *D. papillatum*, the ADAT enzymes perform not only A-to-I but also C-to-U editing, since certain adenine deaminases have a relaxed nucleotide specificity [68].

We detected and manually validated the genes specifying five types of spliceosomal RNAs, namely U1, U2, U4, U5, and U6 small nuclear RNA (snRNA). U2 RNA occurs as often as 163 times in the assembly, with 151 identical copies. Most U2 RNA genes are part of a repeat region in which they alternate with the genes for 5S RNA and SL RNA, up to 27 times in a row.

Finally, we validated the predicted SL RNA genes of *Diplonema*, which are composed of a 39-bp long 5′-exon (the SL) and a 75-nt long intron. This gene occurs in 110 copies (at ≥ 90% sequence identity), forming a tandem repeat unit together with the 5S rRNA and U2 snRNA genes (Additional file 1: Sect. 6. Repetitive sequences in the nuclear genome of *Diplonema papillatum* (assembly v_1.0)). Among the eukaryotes possessing SL RNA, the gene is often part of a tandem repeat unit and associated with the 5S rRNA gene (e.g., in some animal and dinoflagellate groups, euglenids, and kinetoplastids [69, 70]. However, a repeat unit consisting of three alternating ncRNA genes as in *D. papillatum* (SL RNA–5S rRNA–U2 snRNA) is exceptional and also seems to be absent in other diplonemids. (For more details on structural RNAs, see Additional file 1: Sect. 4. Intron splicing and structural RNAs).

### Genes involved in the general cellular metabolism

Among the ~ 37,000 *D. papillatum* protein-coding genes, at least 15% are predicted to be involved in metabolism. Biochemical studies of metabolic processes in diplonemids have investigated glycolysis and gluconeogenesis [35, 71], carbon storage [72], respiration [73], and free-radical detoxification [74]. In addition, recent in silico transcriptome analyses have provided an overview of basic metabolic pathways such as fatty acid synthesis and degradation, pyruvate metabolism, and pentose phosphate pathway across diplonemids [34] and in *D. papillatum* specifically [75]. The limitation of transcriptome-based studies is that the data may include unrecognized contamination with mRNAs from other organisms or lack reads from genes poorly expressed under the examined conditions. Still, the metabolism of diplonemids inferred from the transcriptomes is overall in agreement with that inferred from the nuclear genome sequence presented here. In the following section, we will focus on the polycarbohydrate metabolism of *D. papillatum*, an aspect neglected in earlier work and, as we will show, one with important bearings on the ecological role of this protist in the marine environment.

### Gene complement participating in polycarbohydrate metabolism of *D. papillatum* and other euglenozoans

**Fig. 4:**
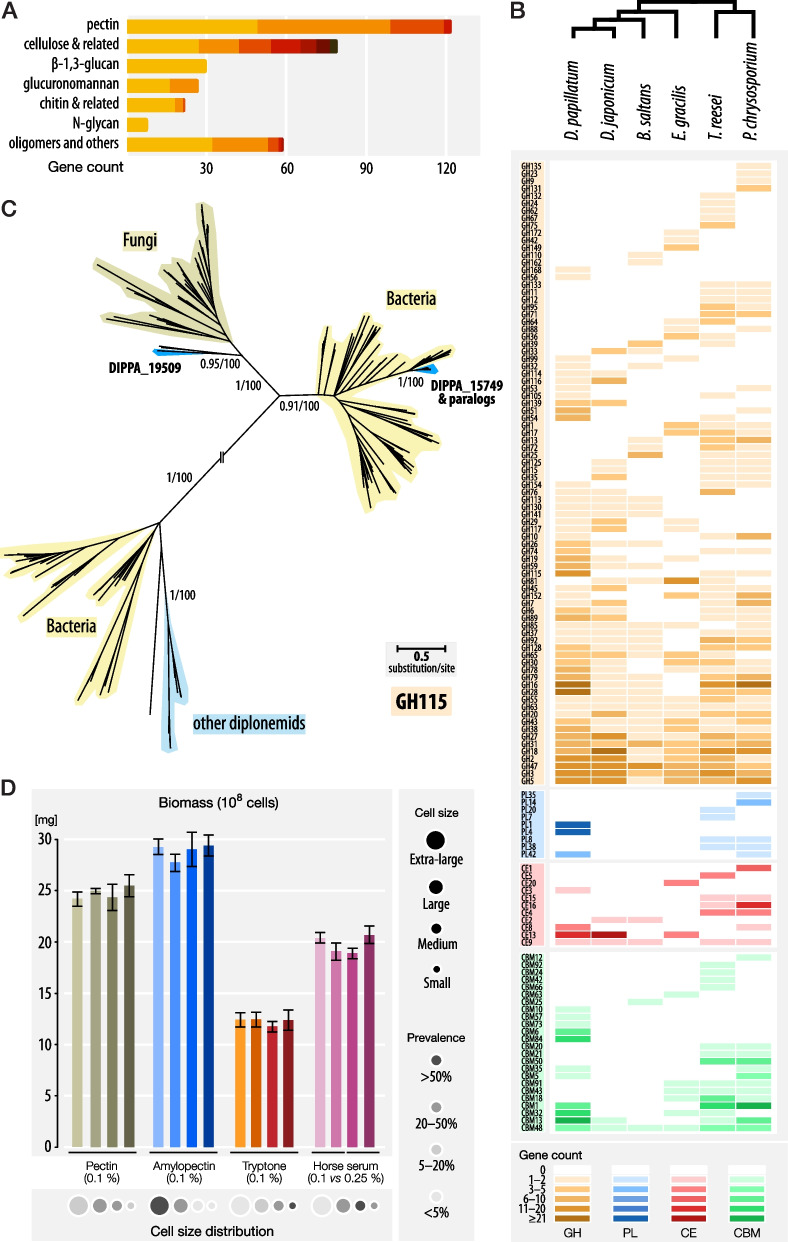
Polycarbohydrate metabolism in *D. papillatum*. **A** Proteins containing at least one CAZyme domain. Proteins were grouped by their cognate substrate class. The subdivision of the bars by different color shades represents the number of enzymes in the following subgroups. *pectin*: pectin hydrolases, pectin lyases, pectin acetylesterases, and pectin methylesterases. *cellulose & related*: cellulases, xylan-α-glucuronidases, xylan/cellulose and xylan/xyloglucan hydrolases, hemicellulases, β-glucan/β-xylan hydrolases, and β-mannanases. *β-1,3-glucan*: no subgroups. *glucuronomannan*: α-mannanases and β-glucuronidases. *chitin & related*: chitinases, glycosaminoglycan and glucosamine hydrolases. *N-glycan*: no subgroups. *oligomers and others*: α-glycosidases, β-glycosidases, trehalases, an α-fucanase, and an invertase. **B** Distribution of the CAZyme families GH (glycoside hydrolase), PL (polysaccharide lyase), CE (carbohydrate esterase), and CBM (carbohydrate-binding module) across four free-living euglenozoans (*D. papillatum*, *D. japonicum*, *B. saltans*, and *E. gracilis*) and two representative fungi (*Trichoderma reesei* and *Phanerochaete chrysosporium*). Rows correspond to individual CAZyme families with heatmap shading indicating the number of family members in each genome as detailed in the key (bottom). **C** DIPPA_15749, a GH115-family member and its 12 paralogs, were most likely acquired specifically by *D. papillatum* via horizontal transfer from diverse bacteria. Sequences that belong to bacteria, fungi, and diplonemids other than *D. papillatum* are highlighted in shades of yellow, beige, and light blue, respectively. For details, see Additional file 1: Sect. 15. Genes horizontally transferred from bacteria to *Diplonema papillatum*. **D** Biomass of *D. papillatum* cells grown in various substrates. The cell sizes are represented as circles of different diameter and the predominance of the various sizes by the grey shade of the fill. Cells were counted in triplicate after six days and weighed to calculate their biomass (wet weight per 10^8^ cells). Bars indicate the mean deviation of the cell counts for each of the four independent biological replicates. Note that the predominant cell size correlates with both biomass and the number of granules

Enzymes involved in the synthesis and degradation of polysaccharides (referred to here as Carbohydrate-active enzymes (CAZymes)) currently comprise ~ 350 distinct catalytic families and about 90 non-catalytic families (Additional file 1: Sect. 12. CAZyme-coding genes in *Diplonema papillatum*). The nuclear genome of *D. papillatum* encodes nearly 500 CAZymes from 52 families for metabolizing diverse polysaccharides (Fig. 4A). By far the most diverse and largest enzyme group is involved in the degradation of pectin (a heteropolysaccharide consisting mostly of methyl-esterified α-D-1,4-galacturonic acid units), with about 120 genes from nine distinct CAZyme families. The second largest group consists of 82 proteins that belong to CAZyme families breaking down the β-1,4-linked glucose polymers cellulose or hemicelluloses. Further, we retrieved 27 homologs of enzymes degrading sulfated glucuronomannan (α-1,3-mannan with β-D-glucuronic acid side chains), which is the main polysaccharide component in the cell wall of diatoms [76]. In addition, the presence of certain glycoside hydrolase homologs in the genome assembly suggests that *Diplonema* is most likely able to digest the β-1,3-glucan laminarin, which is the storage polysaccharide of numerous micro- and macroalgae [77]. Laminarin plays a major role in the marine carbon cycle representing ~ 10% of the carbon produced globally [78]. The *D. papillatum* genome assembly also revealed 18 genes which, in model organisms, were shown to break down chitin (polymer of N-acetylglucosamine) and glycosaminoglycans, both extracellular polysaccharides of animals and fungi. Finally, the *D. papillatum* genome encodes 90 CAZyme genes whose substrate cannot be inferred with confidence. Some of these genes might be involved in the breakdown of complex glycans such as the transparent exopolymer particles (TEPs) secreted by diverse marine eukaryotes [79].

CAZyme genes that are conspicuously lacking in the *D. papillatum* genome assembly are homologs of enzymes degrading bacterial cell-wall components, a finding that is corroborated by feeding experiments [11]. The seemingly strictly eukaryotic diet of *D. papillatum* contrasts with the food preference of, e.g., the diplonemid *Rhynchopus euleeides*, to our knowledge the first reported bacterivorous diplonemid [80]. Also missing in the *D. papillatum* genome are genes encoding poly-α-D-1,4-glucose-depolymerizing enzymes, which might suggest that this organism is unable to digest starch and glycogen, the carbon-storage compounds of Viridiplantae and Metazoa, respectively. However, the latter inference contradicts the results of our feeding experiments (see further below), which demonstrate that *Diplonema* readily utilizes amylopectin, the predominant constituent in starch (see Additional file 1: Sect. 13. Glycan and peptide assimilation by *Diplonema papillatum*). The gene(s) responsible for amylopectin degradation may be among the functionally unassigned CAZymes mentioned above or belong to novel families. Interestingly, polyglycan-degrading enzymes are one of the largest CAZyme class predicted to be secreted outside the *Diplonema* cell (Additional file 1: Sect. 14. Secretome prediction), indicating an important role of this activity for the feeding strategy of this microeukaryote (see below).

In the search for *Diplonema* genes involved in the formation of carbon storage, we identified homologs of β-1,3-glucanase indicating the synthesis of paramylon, a polysaccharide long known from *Euglena* and recently identified experimentally also in *D. papillatum* [72]. As not only *Euglena* and *Diplonema* but also *Bodo* store their carbon in that form [81], paramylon was probably already synthesized by the last common ancestor of Euglenozoa.

Among the examined euglenozoans—i.e., *D. papillatum*, its closest described relative *D. japonicum* [8], the free-living kinetoplastid *B. saltans* [46], and the recently sequenced euglenid *E. gracilis* [48]—it is *D. papillatum* that carries the largest complement and diversity of CAZyme families. The carbohydrate-degrading enzymes (GH, PL, CE) and carbohydrate-binding modules (CBM) are particularly expanded in the diplonemid type species (Fig. 4B; Additional file 1: Sect. 12. CAZyme-coding genes in *Diplonema papillatum*, Additional file 4). Most notably, none of the other euglenozoans appears to possess nearly as many enzymes for pectin and β-1,3-glucan degradation (only 2–33% of the *D. papillatum* numbers). The exceptionally large repertoire of CAZyme genes in *D. papillatum* is comparable to that of saprophytic fungi and should allow this protist to feed on a multitude of algal and plant species occurring in the natural marine habitat of diplonemids [82]. Furthermore, the striking differences in CAZyme complement between the two closely related diplonemids that we examined provide a new window not only into the dynamic nature of diplonemid gene repertoires, but also an opportunity to begin to understand how the gene content impacts the varying lifestyles of diplonemids in general [18].

### Horizontal gene transfer in *D. papillatum*

An important factor leading to differences in gene complements between closely related species is acquisition of genes by horizontal transfer (HGT). As bacteria-to-eukaryote gene transfers appear to be particularly frequent in marine ecosystems [83], we searched for similar signs of such HGTs in *D. papillatum*.

Genes that were likely acquired from bacteria by HGT (referred hereafter to as “HGT genes”) were identified by best reciprocal blast hits against NCBI nr and a set of custom proteomes representing all domains of life, followed by phylogenetic inference and selection of well-supported tree topologies. Validation of candidate HGT genes included visual inspection of trees to assure that the *Diplonema* protein is nested within a bacterial clade. We also verified that the corresponding gene resides on a contig that also encodes typical, presumably endogenous nuclear genes and that the transcript carries an SL, which provides an extra layer of confidence that the gene is indeed expressed (Additional file 1: Sect. 15. Genes horizontally transferred from bacteria to *Diplonema papillatum*; Additional file 5).

The *D. papillatum* nuclear genome assembly includes at least 96 genes likely acquired horizontally from bacteria. These HGT genes form 56 families with up to 14 members; all are transcribed. Ten families have multiple members, with some expansion being a result of tandem gene duplication into up to six copies. Two out of the three largest gene families play a role in the detoxification of reactive oxygen species, but the majority of families participate in metabolic pathways. Four HGT families with a total of 17 members are predicted to be CAZymes, which apparently were acquired specifically by *D. papillatum* because they are not detected in the transcriptomes of the 10 other diplonemids examined (Fig. 4B). The largest HGT-CAZyme family (expanded to 12 members) encodes xylan-α-glucuronidases of the glycoside hydrolase family GH115, which comprise enzymes that break down hemicelluloses. Phylogenetic analysis places these *D. papillatum* proteins as a sister clade to Planctomycetes, Bacteroidetes, Gammaproteobacteria, or Verrucomicrobia, reflecting highly diverse donors as well as potential HGT among bacteria themselves (Fig. 4C).

As observed in other systems, most genes transferred from bacteria to eukaryotes expand or rewire the metabolic capabilities of the recipient [84]. Similarly to what has been documented in other eukaryotes (e.g., [85, 86]), in *Diplonema*, genes encoding CAZymes represent one of the most frequently horizontally acquired functional categories.

### Gene-family evolution in *D. papillatum* and other diplonemids

In addition to gene acquisitions and losses, the *Diplonema* genome is also shaped by gene duplications followed by sequence divergence of copies, leading to multi-gene families that grow or shrink over time.

**Fig. 5:**
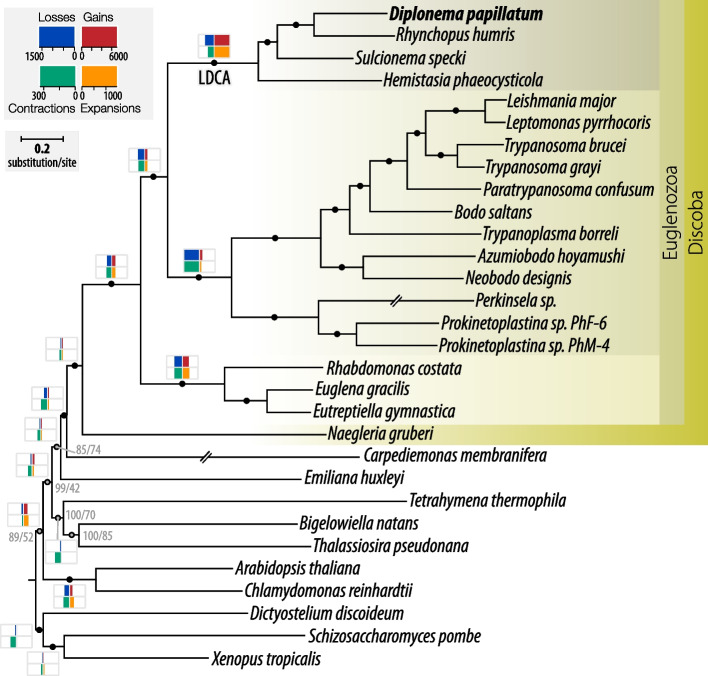
Gene-family evolution in Euglenozoa. A maximum-likelihood phylogenetic tree based on the concatenated alignment of 167 proteins containing 57,565 amino acid positions. Nodes with maximal statistical support are indicated with black circles, for the remaining nodes the supports are in grey in the following format: bootstrap support/SH-aLRT value. Double-crossed branches were reduced to half of their original length. The black horizontal bar indicates the number of substitutions per site. The number of gene families lost, gained, expanded, and contracted at selected nodes (based on the sum of probabilities of the respective events at each node/tip) is indicated by the width of blue, red, orange, and green boxes, respectively. Key, event scales. Note the exceptionally large gain and expansion of more than 7000 and 1400 gene families, respectively, on the ancestral diplonemid branch. Diplonemid, kinetoplastid, and euglenid taxa are highlighted in beige background shades. LDCA, last diplonemid common ancestor

To investigate the gene-family evolution of the *Diplonema* genome, we established a proteome dataset comprising 30 eukaryotes that include *D. papillatum* and three other diplonemids, eight additional euglenozoans, and 12 eukaryotic species from major groups outside Euglenozoa. We used OrthoFinder 2 [87] to infer gene families and orthologous groups for subsequent analysis. OrthoFinder retrieved nearly 200 orthologs with representation in 25 or more taxa, which we concatenated in order to infer a species tree using the LG + C60 + F substitution model, the best-fitting model as determined by the BIC criterion in IQ-TREE [88]. The resulting tree furnished a well-resolved backbone phylogeny for the euglenozoan clade (Fig. 5). The complete set of gene families was then used to estimate protein-family expansions, contractions, gains, and losses by using the phylogenetic birth–death model implemented in Count [89] (Additional file 1: Sect. 16. Evolution of gene families; Additional file 6).

Within the euglenozoan clade, gene family gains and expansions are much more frequent than losses and contractions. The highest count of gene-family gains in the entire tree is the ancestral diplonemid node, indicating a substantial diversification of the gene repertoire in the last diplonemid common ancestor (LDCA, Fig. 5). Similarly prolific is the expansion of protein families at that node. In addition to CAZymes and cytoribosomal proteins discussed in detail in previous sections, highly expanded families act in signal transduction, with a number of predicted protein kinases comparable to that in human [90] and plants [91]. Gene families involved in amino acid metabolism also expanded at the diplonemid node. A noteworthy finding is the gain of glycine amidination and methylation genes, which indicates that diplonemids are capable of converting glycine into creatine, a scarce compound in marine environments that is otherwise only supplied by metazoan and diatom excretion [92]. The gene families that expanded specifically in *D. papillatum* but not in the other diplonemids are involved in oxidative stress protection, including two families acquired horizontally from bacteria. This expansion might be an adaptation to life in the surface seawater layer penetrated by solar radiation that triggers the production of cytotoxic reactive oxygen species (ROS), an adaptation likely protecting also from ROS generated by man-made pollutants such as metals, polychlorinated biphenyls, and radioisotopes in coastal waters.

### Carbon nutrition

Earlier experimental studies showed that *D. papillatum* does not import glucose in any significant amount from the medium, but instead readily takes up and metabolizes amino acids [35]. The authors concluded from this observation that in its natural habitat, the primary energy source of *D. papillatum* is not carbohydrates as is the case in most heterotrophic eukaryotes, but rather poly- and oligo-peptides. However, these earlier inferences about *Diplonema*’s nutrition are in conflict with our finding described here of a large ensemble of highly transcribed carbohydrate-metabolizing genes in the inferred proteome and secretome.

Certain diplonemids (though not *D. papillatum*) have been observed to feed on microalgae and decaying water plants (e.g., [8, 82, 93]), strongly suggesting that in its natural habitat *D. papillatum* uses its large CAZyme arsenal to break down cell-wall components of diverse prey (Additional file 1: Sect. 12. CAZyme-coding genes in *Diplonema papillatum*). The question arises whether cell-wall degradation serves *D. papillatum* solely for gaining access to proteins inside the prey’s cell (referred to as protoplast feeding such as described recently for an amoeba [94]), or rather for assimilating the carbohydrates in the cell wall and/or intracellular storage glycans from starch to laminarins.

To test the hypothesis that *D. papillatum* is indeed able to assimilate cell-wall and storage glycans, we performed growth experiments in media of various compositions (Additional file 1: Sect. 13. Glycan and peptide assimilation by *Diplonema papillatum*). In agreement with previous studies [35, 72], our results confirm that this protist only poorly utilizes glucose as sole carbon source. However, our data also indicate that it does grow well on polycarbohydrates such as pectin and particularly amylopectin. Most importantly, *D. papillatum* utilizes carbohydrates as efficiently as peptides (Fig. 4D). Together with the identification of numerous CAZyme homologs and carbohydrate-transporter genes in the *D. papillatum* genome, we conclude that this organism degrades extracellular polysaccharides obtained from marine prey and imports oligomers into the cell for assimilation (Additional file 1: Sect. 17. Feeding strategy and food of *Diplonema papillatum*).

In addition, our findings question the view that *D. papillatum* is an exclusive osmotroph in marine environments, scavenging on debris of dead organisms. The results presented here suggest rather that in the wild, *D. papillatum* feeds mostly on living eukaryotes. We posit that this protist enzymatically pierces and ruptures live prey cells and then engulfs cell-wall particles and cytoplasm alike. This feeding strategy would allow *D. papillatum* to forage on living eukaryotes from a broad taxonomic range and of any physical size, from diatoms to dinoflagellates, macroalgae, and aquatic plants.

### Environmental distribution of *D. papillatum*

**Fig. 6:**
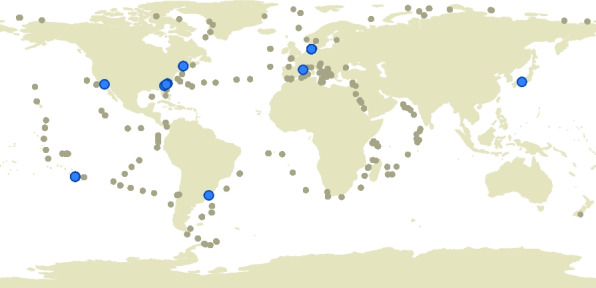
Oceanic distribution of *D. papillatum*. The world map shows the distribution of sampling locations from the three datasets in which *D. papillatum* was detected, namely the Tara project, “Ocean Sampling Day 2014,” and “Helgoland Roads 2016” (beige dots). Sites at which *D. papillatum*-representing OTUs (operational taxonomic units) were detected are highlighted in blue. For details see the main text and Additional file 7)

Diplonemids are essentially omnipresent in marine environments, found from the tropics to the poles, in the top layer down to abyssal/hadal zones (> 6000 m below the surface), and in both pelagic (planktonic) and benthic (sediment) habitats [3]. We have a good understanding of the environmental and geographical distribution of the major diplonemid groups but not of the type species. Therefore, we searched available datasets of the V9 [4, 95–97] and V4 [96–99] hypervariable regions of the 18S rDNA for the presence of *D. papillatum* sequences (Additional file 1: Sect. 18. Environmental distribution of *Diplonema papillatum*). We detected no *D. papillatum* reads in datasets from samples collected in the open ocean, or from waters below ~ 10 m depth and 6 °C temperature. Instead, signature sequences of the type species were present in datasets of temperate coastal regions, from Helgoland to Japan, and the Americas (Fig. 6).

To summarize, the abundance of the type species and many other Diplonemidae members in marine habitats is relatively low. *D. papillatum* occurs sporadically in temperate coastal surface waters of the world ocean. We suggest that it preferentially populates coastal regions because they are more eutrophic than the open sea and thus much richer in plants and algae, the postulated major food sources of this organism.

## Conclusions

Our analysis of the *D. papillatum* gene complement has provided insights into the previously unknown, central role of polysaccharide degradation in this organism, allowing inferences about its ecological role. However, these insights are not necessarily transferrable to other diplonemids, because their CAZyme complement is different from that of *D. papillatum*. Moreover, all examined diplonemid species belong to experimentally tractable Diplonemidae and Hemistasiidae. We know nearly nothing about the metabolic capabilities and ecological role of the DSPDII group and of Eupelagomenidae in particular, which is the most abundant and diverse diplonemid clade. With recent advancements in addressing the challenges of single-cell technologies, from single-cell genomics to metabolomics [100], we should soon be able to fill this knowledge gap.

## Methods

### Strains and culture conditions

*Diplonema papillatum* (ATCC50162) was cultivated axenically at 15–22 °C in liquid medium containing 33 g/L Instant Ocean Sea Salt (Instant Ocean) and supplemented with 1% (v/v) horse serum as described earlier [73].

### Extraction of nucleic acids and genome and transcriptome sequencing

Total cellular DNA was isolated from disrupted cells using Genomic-tip 100/G (Qiagen). Total cellular RNA was extracted using a home-made Trizol substitute [101], and residual DNA was removed by digestion with an RNase-free DNase. Poly(A) RNA was enriched by passage through oligo(dT)-cellulose. Library construction and Illumina and PacBio sequencing were performed by technology platforms. For details on strains, culture, nucleic acid extraction, and sequencing, see Additional file 1: Sect. 19. DNA and RNA preparation for high-throughput sequencing.

### Assembly, structural, and functional annotation

We generated 462 million pairs of short reads (Illumina) and ~ 725,000 long reads (PacBio) totaling 126.4 Gbp raw data. Short and long reads were assembled separately with the Celera Assembler [102] and Canu [103], respectively, and non-redundant contigs were merged. Transcript sequences were obtained by de novo assembly of the ~ 645 million reads from the strand-specific poly(A) RNA libraries, and used in gene model prediction. Structural genome annotation was performed with an in-house developed tool [45]. For quality assessment, the gene models of the three largest contigs were expert-validated. Functional information was assigned by protein-sequence similarity to the SwissProt database and Hidden Markov Model (HMM) searches [104]. The proteins without SwissProt information were labelled as “hypothetical proteins.” Transfer RNA genes were searched with tRNAscan-SE, rRNAs with Hmmerscan using profile HMMs from Rfam [105], and spliceosomal RNAs with Cmsearch [106] using home-built covariance models. For details on the assembly, annotation, and expert validation, see Additional file 1: Sect. 2. Assembly and annotation of the nuclear genome and transcriptome of *Diplonema papillatum* and Additional file 1: Sect. 4. Intron splicing and structural RNAs).

Otherwise, methods and data sources are described in detail in the corresponding Supplementary Information files with a focus on nuclear DNA structure and chromosome separation; ploidy; genomic repeats and NUMTs; RNA splicing, introns, and structural RNAs; cytoribosome; untranslated regions; polycistronic RNAs; DNA modifications; RNA interference machinery; gene complement; meiosis; CAZymes; nutrient assimilation; secretome; horizontal gene transfer; gene-family evolution; feeding behavior; and environmental distribution.

## Supplementary Information

**Additional file 1.** Supporting information with additional details on the various topics described in the main text.

**Additional file 2.** Curated list of high-confidence nuclear mitochondrial segments (NUMTs) that can be anchored to non-repetitive, coding sequences of the mitochondrial genome. See also Additional file 1: Section 6. Repetitive sequences in the nuclear genome of *Diplonema papillatum* (assembly v_1.0).

**Additional file 3.** Gene count and orientation across contigs of the v1.0 assembly. Columns labelled ‘Submitted genome annotation’ show the data for the final assembly and annotation as submitted to NCBI GenBank. For the three longest contigs, we provide the corresponding information after expert curation and masking of genes derived from transposons (columns labelled ‘Fully curated genome annotation and masked transposons’). See also Additional file 1: Section 7. Polycistronic transcription units in the nuclear genome of *Diplonema papillatum*.

**Additional file 4.** List of genes coding for carbohydrate-interacting proteins. A) CAZyme genes detected in *D. papillatum*. B) CAZyme genes detected in *D. japonicum*. See also Additional file 1: Section 12. CAZyme-coding genes in *Diplonema papillatum*.

**Additional file 5.** List of candidate genes horizontally transferred specifically from bacteria to *D. papillatum* or to the common ancestor of diplonemids. See also Additional file 1: Section 15. Genes horizontally transferred from bacteria to *Diplonema papillatum*.

**Additional file 6.** Detailed information on the evolution of gene families in diplonemids. A) Summary of gene-family gain/loss/expansion/contraction events. B) Diplonemid-specific gene-family gain events. Gene families gained on the ancestral diplonemid branch. C) Diplonemid-specific gene-family loss events. D) Diplonemid-specific gene-family expansion events. E) Diplonemid-specific gene-family contraction events. F) *D. papillatum*-specific gene-family gain events. Gene families gained on the ancestral diplonemid branch (posterior probability ≥0.5). G) *D. papillatum*-specific gene-family loss events. H) *D. papillatum*-specific gene-family expansion events. I) *D. papillatum*-specific gene-family contraction events. See also Additional file 1: Section 16. Evolution of gene families.

**Additional file 7.** List of samples from various locations of the world ocean investigated for the presence of *D. papillatum*. See also Additional file 1: Section 18. Environmental distribution of *Diplonema papillatum*.

**Additional file 8.** Uncropped gels and blots shown in the **Supplementary Figure S2.** See also Additional file 1: Section 1. Physical structure and size of the *D. papillatum* nuclear genome.

## Data Availability

The datasets supporting the conclusions of this article are included as additional files or have been deposited under NCBI BioSample ID SAMN30986590 [107] and BioProject ID PRJNA883718 [108], including genome and transcriptome assemblies, genome annotations, and the inferred proteome.

## Abbreviations

DSPD: Deep-sea pelagic diplonemid
rRNA: Ribosomal RNA
SNV: Single-nucleotide variant
UTR: Untranslated region
SL: Spliced-leader
ORF: Open reading frame
TFIIα: Transcription initiation factor II alpha
CDS: Coding sequence
LINE-1: Long interspersed nuclear element-1
SLACS: Spliced-leader-associated-conserved sequence
TATE: Telomere associated transposable element
MULE: Mutator-like element
rDNA: Ribosomal DNA
TRIM: Terminal-repeat retrotransposon in miniature
SINE: Short interspersed element
NUMT: Nuclear mitochondrial segment
Base J: β-D-glucopyranosyl-oxymethyluracil
5mC: 5-Methyldeoxycytosine
RNAi: RNA interference
ncRNA: Non-(protein-)coding RNA
tRNA: Transfer RNA
CAZyme: Carbohydrate-active enzyme
GH: Glycoside hydrolase
PL: Polysaccharide lyase
CE: Carbohydrate esterase
TEP: Transparent exopolymer particle
HGT: Horizontal gene transfer
LDCA: Last diplonemid common ancestor
ROS: Reactive oxygen species
OTU: Operational taxonomic unit
