# Supplementary material for: Recent expansion of metabolic versatility in Diplonema papillatum, the model species of a highly speciose group of marine eukaryotes
Source: BMC Biol. 2023 May 4;21:99. doi: 10.1186/s12915-023-01563-9 (PMC10161547; doi:10.1186/s12915-023-01563-9)
Supplement: Supplementary file 1 — Additional file 1. Supporting information with additional details on the various topics described in the main text. [file 12915_2023_1563_MOESM1_ESM.pdf]

Supporting Information for

Recent expansion of metabolic versatility in *Diplonema papillatum*, the model species of a highly speciose group of marine eukaryotes

Matus Valach[1]*, Sandrine Moreira[1], Celine Petitjean[2], Corinna Benz[3], Anzhelika Butenko[3,4,5], Olga Flegontova[3,5], Anna Nenarokova[2,3], Galina Prokopchuk[3,4], Tom Batstone[2,6], Pascal Lapébie[7], Lionnel Lemogo[1,8], Matt Sarrasin[1], Paul Stretenowich[1,9], Pragya Tripathi[3,4], Euki Yazaki[10], Takeshi Nara[11], Bernard Henrissat[7,12,13], B. Franz Lang[1], Michael W. Gray[14], Tom A. Williams[2], Julius Lukeš[3,4] and Gertraud Burger[1]*

[1] Department of Biochemistry, Robert-Cedergren Centre for Bioinformatics and Genomics, Université de Montréal, Montreal, Quebec, Canada.
[2] School of Biological Sciences, University of Bristol, Bristol, UK.
[3] Institute of Parasitology, Biology Centre, Czech Academy of Sciences, České Budějovice, Czech Republic.
[4] Faculty of Science, University of South Bohemia, České Budějovice, Czech Republic.
[5] Faculty of Science, University of Ostrava, Ostrava, Czech Republic.
[6] Current address: High Performance Computing Centre, Bristol, UK.
[7] Architecture et Fonction des Macromolécules Biologiques (AFMB), CNRS, Aix Marseille Université, Marseille, France.
[8] Current address: Environment Climate Change Canada, Dorval, Quebec, Canada.
[9] Current address: Canadian Centre for Computational Genomics; McGill Genome Centre, McGill University, Montreal, Quebec, Canada.
[10] RIKEN Interdisciplinary Theoretical and Mathematical Sciences Program (iTHEMS), Hirosawa, Wako, Saitama, Japan.
[11] Laboratory of Molecular Parasitology, Graduate School of Life Science and Technology, Iryo Sosei University, Iwaki City, Fukushima, Japan.
[12] Current address: DTU Bioengineering, Technical University of Denmark, Lyngby, Denmark;
[13] Department of Biological Sciences, King Abdulaziz University, Jeddah, Saudi Arabia.
[14] Department of Biochemistry and Molecular Biology, Institute for Comparative Genomics, Dalhousie University, Halifax, Nova Scotia, Canada.
* Corresponding authors.

Matus Valach
Email: matus.a.valach@gmail.com
Gertraud Burger
Email: gertraud.burger@umontreal.ca

This PDF file includes:

Supporting Information Text
Supplementary Figures
Supplementary Tables
Supporting Information References

Supporting Information Text

Contents

1. **Physical structure and size of the *Diplonema papillatum* nuclear genome**
2. **Assembly and annotation of the nuclear genome and transcriptome of *Diplonema papillatum***
3. **The ploidy level of *Diplonema papillatum***
4. **Intron splicing and structural RNAs**
5. **Untranslated regions of nuclear genes**
6. **Repetitive sequences in the nuclear genome of *Diplonema papillatum* (assembly v 1.0)**
7. **Polycistronic transcription units in the nuclear genome of *Diplonema papillatum***
8. **DNA modifications (5mC and J)**
9. **RNA interference (RNAi)**
10. **The cytosolic ribosome of *Diplonema papillatum***
11. **Meiosis in *Diplonema papillatum*?**
12. **CAZyme-coding genes in *Diplonema papillatum***
13. **Glycan and peptide assimilation by *Diplonema papillatum***
14. **Secretome prediction**
15. **Genes horizontally transferred from bacteria to *Diplonema papillatum***
16. **Evolution of gene families**
17. **Feeding strategy and food of *Diplonema papillatum***
18. **Environmental distribution of *Diplonema papillatum***
19. **DNA and RNA preparation for high-throughput sequencing**

1. Physical structure and size of the *D. papillatum* nuclear genome

INTRODUCTION

Long repetitive regions such as centromeres or stretches rich in modified nucleotides have been refractory to complete genome assembly in many organisms. Consequently, we do not know the exact size of the corresponding genomes nor the number and topology of chromosomes. Still, these genomic features can be investigated by various alternative approaches, which we have employed to characterize the nuclear genome of *D. papillatum*.

Flow cytometry

One of the most common experimental methods to estimate the size of a nuclear genome is flow cytometry, which involves the isolation of intact nuclei, DNA staining with a fluorescence dye, and the measurement of the fluorescence signal intensity of individual nuclei. The signal is then compared to that from nuclei of organisms whose genome size is known. Flow cytometry measures the DNA *amount* in cell nuclei, whereby typically the DNA content in both the G1 and G2 phases can be determined. For inferring the genome size, the ploidy level of the organism must be known. If the species is haploid, i.e., mitotic proliferation takes place in the haploid stage, then the genome size corresponds to the DNA amount in the G1 phase. If, however, the species is diploid or of higher ploidy, the genome size would be half etc., of the DNA amount in G1.

To measure the relative nuclear DNA content of *D. papillatum* by flow cytometry, we used a diploid *Saccharomyces cerevisiae* strain (24 Mbp) and a diploid *Drosophila melanogaster* cell line (360 Mbp) as references. *D. papillatum* and the organismal material used as a reference have a single nucleus per cell. The DNA content was measured by the fluorescence intensity after staining the nuclei with the fluorophore 4′,6-diamidino-2-phenylindole (DAPI). **Supplementary Figure S1** shows the distribution of the DNA amount across the nuclei examined. All three samples display a prominent peak of G1-phase cells and a minor population in G2, since the cultures were asynchronous. The DNA content of *D. papillatum* is smaller than that of the fly but considerably larger than that of yeast.

However, extrapolating the exact DNA content of *D. papillatum* nuclei is not feasible in this case due to several limitations. One issue is that DAPI binds preferentially to A+T-rich DNA and that the A+T content of the three nuclear genomes varies between yeast 52% (yeast), *Diplonema* (55%) and fly (57%). Still, the advantage of DAPI is that, in contrast to other dyes, DNA binding is not influenced by chromatin structure, which fluctuates drastically between the three organisms. The yeast genome contains nearly no heterochromatin, whereas that of the fly includes 30%, and that of *D. papillatum* is estimated at 50% considering the large portion of repetitive sequence and mobile elements. The second issue is the difference in cell-wall composition and rigidity that necessitated specifically adapted cell-rupture and nuclei-isolation procedures, which in turn produced nuclei of various purity and yield. The elevated fluorescence background that we observed in *D. papillatum* was most likely due to the contamination by mitochondrial DNA, which is present in an extraordinarily high proportion in this species (LUKEŠ *et al.* 2018).

In conclusion, the flow-cytometry determination of the DNA content in the *D. papillatum* nucleus shown here is in rough agreement with the haploid genome size inferred from the DNA sequence, i.e. 260 Mbp estimated from the k-mer distribution in reads and 280 Mbp, the size of the nuclear genome assembly (see main text).

Yet, to infer the actual genome size of an organism, the ploidy level must be known. As detailed in the **Supplementary Information**: Section 3. The ploidy level of *Diplonema papillatum*, the *D. papillatum* nuclear genome displays extremely low heterozygosity as revealed by the unimodal k-mer distribution and the low number of single-nucleotide variants. This indicates that the *D. papillatum* nuclear genome is either haploid or alternatively, autodiploid (or even tetraploid, etc.) originating from a recent genome duplication without allelic divergence. However, autodiploidy of *Diplonema* is highly unlikely because the nuclear DNA content would then be >500 Mbp, which contradicts the flow cytometry experiments shown here. Therefore, we conclude that the *D. papillatum* nuclear genome is haploid with a (haploid) genome size between 100 and 300 Mbp. Considering the size obtained by k-mer counts in sequencing reads, we consider 260 Mbp to be the most accurate estimate of the *D. papillatum* nuclear genome.

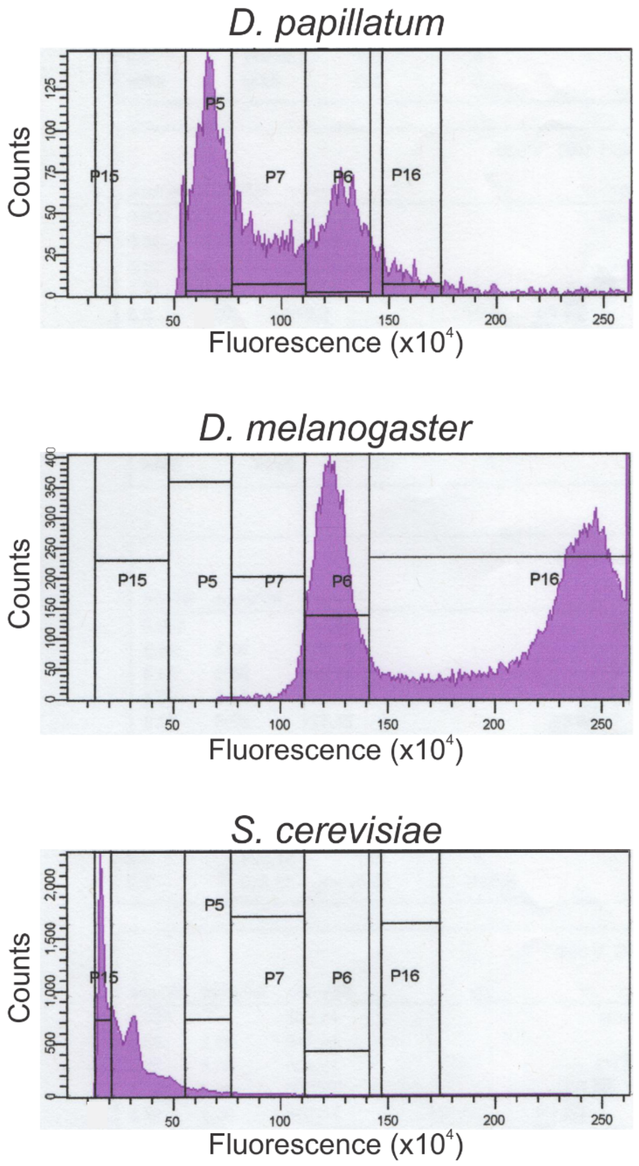

Supplementary Figure S1. Histograms of fluorescence intensity corresponding to nuclear DNA content. The left and right peaks represent nuclei in the cell-cycle phases G1 and G2, respectively. Nuclei were stained with DAPI, and fluorescence was measured with a FACScalibur instrument. Counts, number of nuclei. P5 to P16, nuclei populations selected by gating.

Chromosomes and nuclear organization

D. papillatum cells have a single nucleus (Marande *et al.* 2005), but the number of chromosomes and their condensation state are unknown. We addressed these questions through pulsed-field gel electrophoresis and electron microscopy experiments.

Number and size of chromosomes

Pulsed-field gel electrophoresis (PFGE) separated readily intact nuclear chromosomes from the 6-7 kbp circles of mitochondrial DNA (Marande et al. 2005; Moreira et al. 2016). After migration, the nuclear DNA was visualized by Southern hybridization using a probe that contains telomeric repeats. Three signals were observed: a smear spanning from 1.1 to 1.8 Mbp with only a slight indication of a banding pattern and, in addition, two thin bands at ~500 kbp and ~1 Mbp (**Supplementary Figure S2**). Most of the nuclear DNA in the 1.1–1.8 Mbp zone most likely corresponds to a large number of unresolved chromosomes, whereas the thin bands appear to represent single chromosomes. At an estimated size of ~260 Mbp for the entire genome (which is haploid, see main text), the *D. papillatum* nucleus is predicted to contain approximately 180 chromosomes.

Compared to other Euglenozoa, the *Diplonema* nuclear genome appears much more fragmented than that of its relatives. Trypanosomes and leishmanias (nuclear genome size ~18–33 Mbp) have 11–36 chromosomes ranging in size from 0.5 to 6 Mbp. In addition, trypanosomes also contain up to a hundred smaller, non-Mendelian chromosomes (Maslov et al. 2019). Similarly, the *Euglena gracilis* nuclear genome (size ~500 Mbp (Ebenezer et al. 2019)) has an estimated chromosome number of 42 (Dooijes et al. 2000).

DNA arrangement in the nucleus

The arrangement of nuclear DNA was assessed by thin-section transmission electron microscopy and classical and expansion fluorescence microscopy. The nucleus of *Diplonema* cells occupied a volume of, on average, 14.35 μm^3 and was roughly spherical (0.895 sphericity). In all three nuclei analyzed in detail, the chromosomes formed a network structure (0.104 sphericity) that took up ~18% of the nucleus (**Supplementary Figure S3A-D, S4**). Despite the size variation of the nuclei examined, the proportions of sphericity and volume of nuclei and reticulated chromatin were constant. Occasionally, we observed small (<0.117 μm^3) chromatin granules detached from the chromosomal network that might represent analogs of Cajal bodies or nuclear speckles. When cells were stained with DAPI or other DNA-binding dyes and inspected by fluorescence microscopy, the network appeared as a pattern of bright clusters in a spherical arrangement characteristic of diplonemids (Lukeš et al. 2018) (**Supplementary Figure S3E, S4A,E**). This indicates that certain chromatin regions accumulated more dye and thus were likely more condensed. Inside the nucleus of the *D. papillatum* cells resided a clearly discernable nucleolus that was spherical and slightly granular and filled ~8% of the nucleus (**Supplementary Figure S3**).

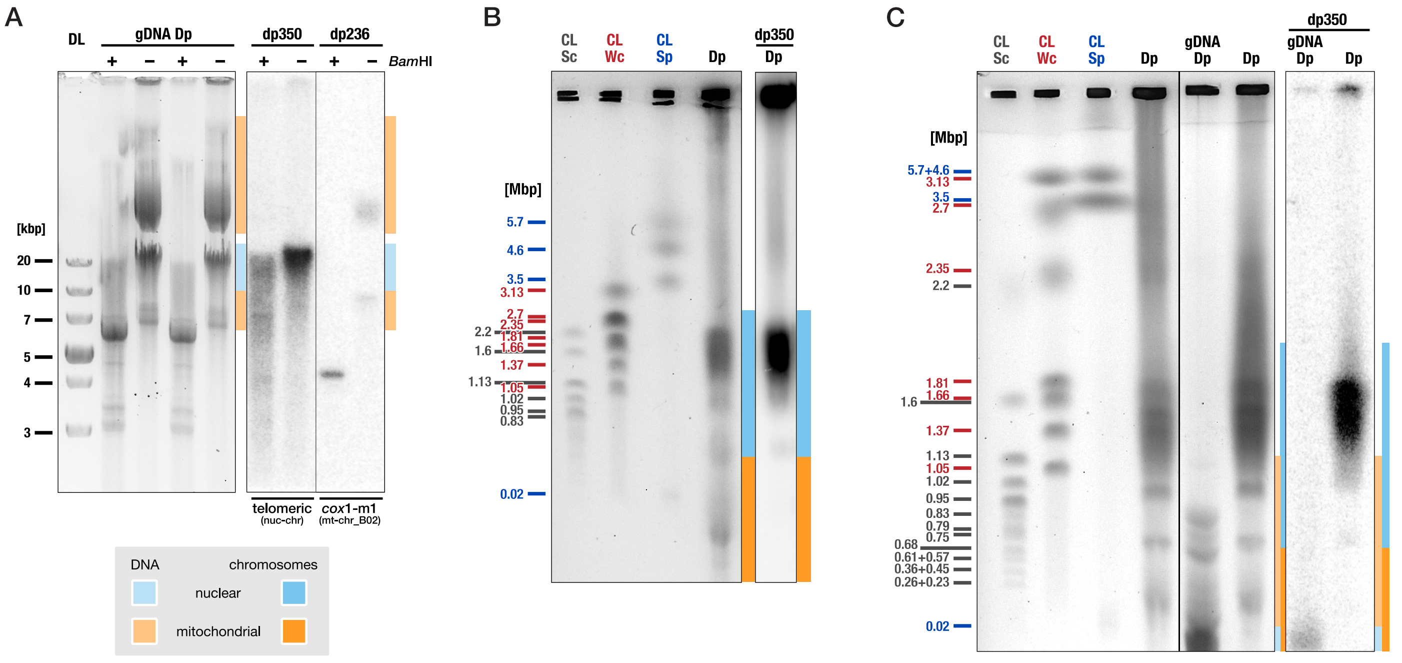

Supplementary Figure S2. Electrophoretic separation of *D. papillatum* nuclear chromosomes. (**A**) Total genomic DNA from *D. papillatum* cells was separated by conventional agarose gel electrophoresis after (+) or prior to (−) digestion with BamHI, blotted, and hybridized to a nuclear telomeric probe (dp350) or a probe targeting the mitochondrial gene piece cox1-m1 (dp236; located on the circular chromosome B02). DL, DNA ladder (GeneRuler 1kb Plus; Thermo Fisher). For oligonucleotide-probe sequences, see **Supplementary Table S2**. (**B**, **C**) DNA from in-gel-lysed cells (Dp) or total genomic DNA (gDNA Dp) was separated by PFGE using two different programs (#1 in B, #2 in C; for details, see **Supplementary Table S2**). After Southern blotting, membranes were hybridized to the telomeric probe dp350. CL, chromosomal ladders (Bio-Rad): Sc, *Saccharomyces cerevisiae*; Wc, *Wickerhamomyces canadensis* (*Hansenula wingei*); Sp, *Schizosaccharomyces pombe*. Key, highlighted zones in which most of the DNA staining-signal originates from nuclear DNA (lighter and darker orange) or mitochondrial DNA (lighter and darker blue).

The study presented here is the first high-resolution examination of the chromatin organization in a diplonemid and, to the best of our knowledge, in a euglenozoan. The first morphological studies of *D. papillatum* already reported that the cells bear a relatively **large nucleus** (~3-4 µm in diameter) of a somewhat ellipsoidal shape and medial location (PORTER 1973). Similar observations were made in other diplonemid species (TASHYREVA *et al.* 2018a; TASHYREVA *et al.* 2018b; PROKOPCHUK *et al.* 2019). A large nucleus is a hallmark of Euglenozoa in general (ANGELER *et al.* 1999; BREGLIA *et al.* 2010; LUKEŠ *et al.* 2021). Another particular feature of the diplonemid nucleus is that the shape and size do not change with growth conditions and stage (e.g., starvation or exponential vs. stationary phase), while the opposite has been reported for trypanosomatids (VICKERMAN AND PRESTON 1970; SCHENKMAN *et al.* 2011).

The **nucleolus** of *D. papillatum* is a conspicuous structure positioned centrally within the nucleus, a trait shared with other Euglenozoa. Size-wise, the nucleolus of *D. papillatum* is comparable with that of kinetoplastids and euglenids examined so far: it occupied a volume slightly larger than in *T. brucei* (ERSFELD 2011), but smaller than in *Euglena gracilis* (O'DONNELL 1965).

The most noticeable nuclear substructure is the permanently reticulated **heterochromatin** (**Supplementary Figures S3A–E, S4**), probably representing permanently condensed subdomains of the chromosomal network. Hemistasiids (PROKOPCHUK *et al.* 2019) and symbiotids (YUBUKI *et al.* 2009) display similar patches and agglomerated masses of heterochromatin in the nucleoplasm, but the corresponding studies did not reconstruct the nuclear ultrastructure in three dimensions and therefore did not inform about the degree of reticulation.

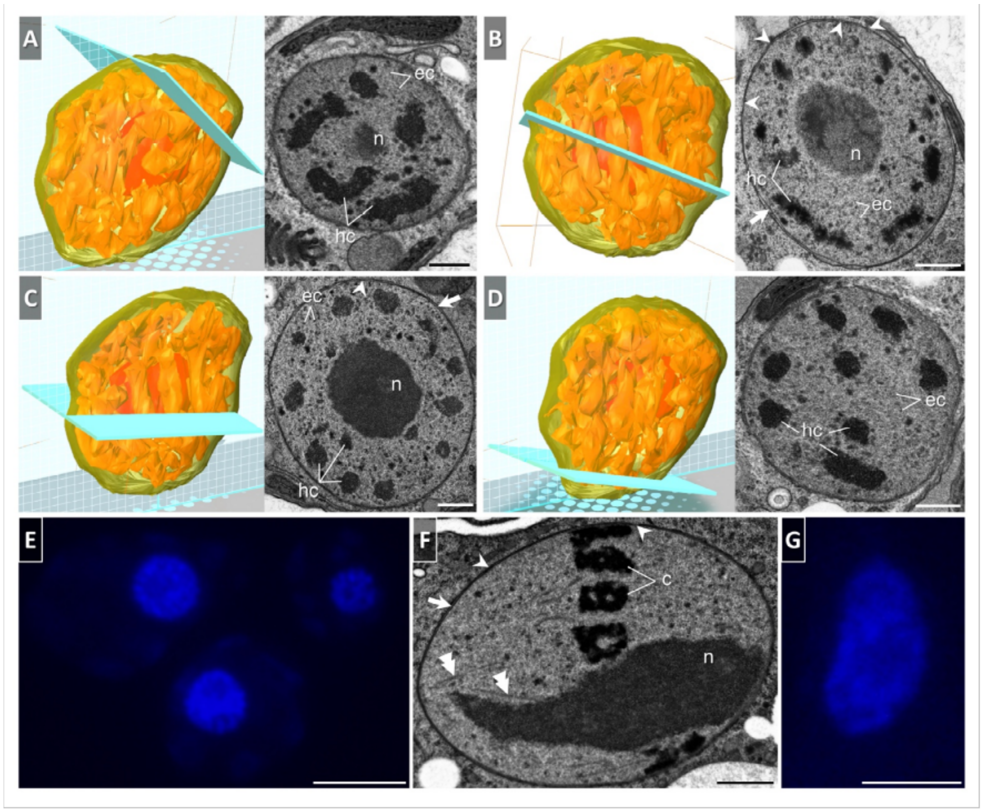

Supplementary Figure S3. Nuclear organization of *D. papillatum*. (**A-D**) TEM images showing cross sections through different parts of the nucleus. The planes of sections are marked in cyan on a 3D model of the entire nucleus. Yellow – nuclear membrane; orange, hc – heterochromatin; red, n – nucleolus; ec – euchromatin; arrows – perinuclear space; arrowheads – nuclear pores. (**E**) DAPI-stained nucleus. (**F-G**) TEM and DAPI-stained nucleus during division; c – chromosomes. Spindle fibers are seen (double arrowheads). Scale bar: 500 nm (**A-D**, F), 5 µm (**F**), 2.5 µm (**G**).

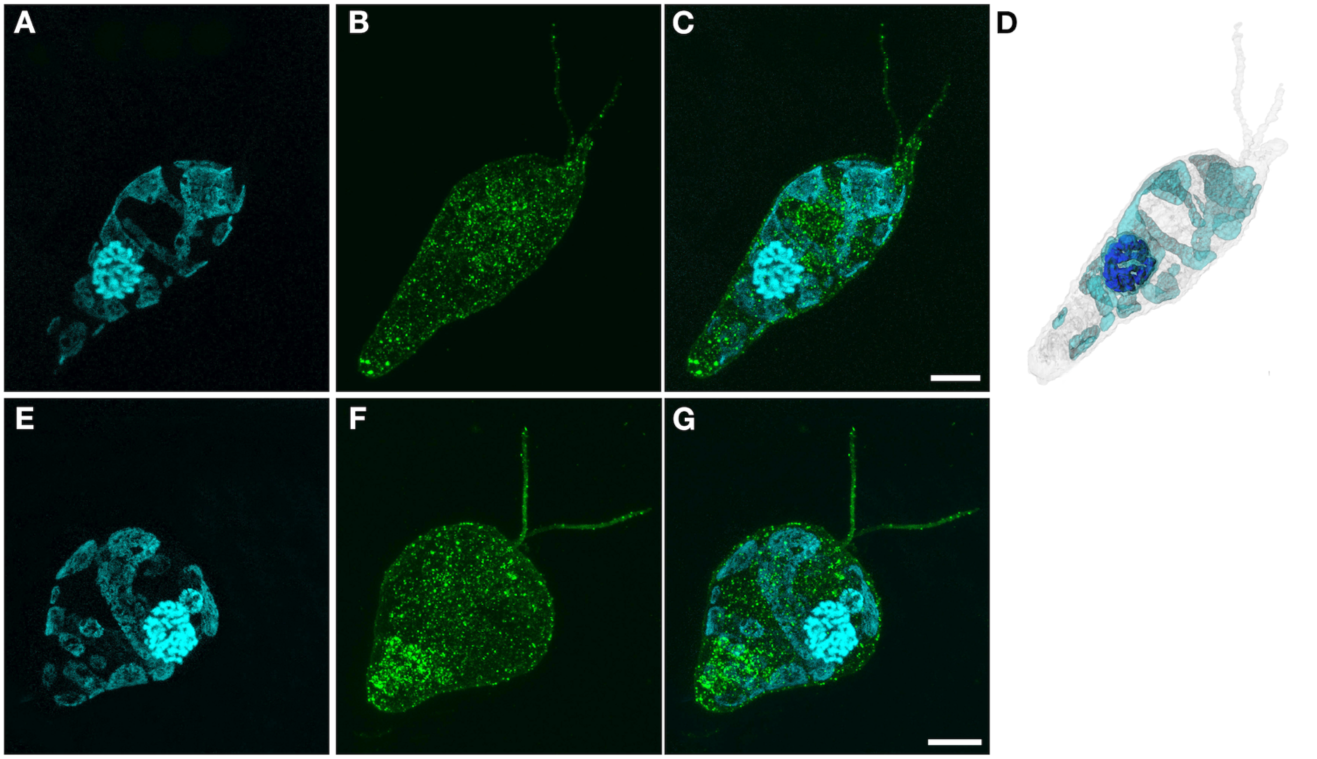

Supplementary Figure S4. Nuclear and mitochondrial organization of *D. papillatum*. (**A-C, E-G**) Expansion microscopy (ExM) images of cells stained with DAPI (**A, E**) and anti-tubulin antibodies (**B, F**), and their merged display (**C, G**). ExM enables more even staining of DNA in the nucleus and mitochondria (compare with **Supplementary Figure S3E–F**). (**D**) 3D model of a *D. papillatum* cell based on the Z-stack series of the cell shown in **A–C**. Light gray – cell surface tubulin; cyan – reticulated mitochondrion; blue – reticulated nuclear heterochromatin. Scale bars: 10 µm.

In *D. papillatum* the **chromatin distribution** is uniform throughout the nuclear space. Such a pattern is common across diplonemids (ROY *et al.* 2007; TASHYREVA *et al.* 2018a; TASHYREVA *et al.* 2018b; PROKOPCHUK *et al.* 2019) and *E. gracilis* (O'DONNELL 1965). However, in most kinetoplastids, symbiotids, and certain euglenids, the chromatin distribution pattern is very different, with condensed heterochromatin occupying the periphery of the nucleus close to the envelope (ELIAS *et al.* 2002; BREGLIA *et al.* 2010).

D. papillatum, and Discoba in general, undergo closed **mitosis** (PATTERSON 1999), whereby the nuclear membrane and the nucleolus structure–which elongates and divides into two daughter nucleoli–are preserved. Further, during cell division, diplonemids form a distinct metaphase plate (**Supplementary Figure S3F**) (PORTER 1973; TRIEMER 1992), while kinetoplastids and certain euglenids assemble chromosomes in a loose equatorial plane (TRIEMER AND FARMER 1991). Metaphase plates occur only in a few other protist groups, but are a typical feature of mitotic division in animals and plants.

Lastly, we show here that expansion microscopy (ExM), a powerful technique allowing visualization of sub-cellular ultrastructures through physical (or, more precisely, mechano-chemical) rather than optical magnification (WASSIE *et al.* 2019), can be successfully applied to diplonemids. ExM readily exposed the reticulated nature of *Diplonema*'s mitochondrion, initially described almost two decades ago (MARANDE *et al.* 2005). In addition, ExM corroborated the reticulated structure of the nuclear heterochromatin (**Supplementary Figure S4D**), which otherwise requires the more resource-demanding TEM.

METHODS

Flow cytometry

D. papillatum was cultured as described earlier (VALACH *et al.* 2014). From ~3×10^6 cells, a nuclei-enriched fraction was recuperated after sucrose gradient centrifugation from the 60% bottom layer and treated with RNase. The diploid *S. cerevisiae* strain BY 4743 (diploid genome size 24 Mbp) was kindly provided by Dr. S. Michnick. Yeast was cultured in a liquid medium containing 0.5 % yeast extract plus 3% glucose and digested with Zymolase to obtain spheroplasts (KISELEVA *et al.* 2007). The *D. melanogaster* male cell line S2 (diploid genome size 360 Mbp) from an asynchronous culture was kindly provided by Dr. V. Archambault. Nuclei from all three eukaryotes were extracted with the Biosciences BD cycleTEST DNA Reagent Kit. We essentially followed the manufacturer's instructions, but for yeast, we ruptured spheroplasts by repeatedly pipetting the suspension up and down through a yellow tip. In addition to the propidium iodine dye included in the kit, the nuclei were stained overnight with DAPI at a final concentration of 1 µg/mL. Fluorescence was measured with a FACScalibur instrument at an FSC setting of 500 V. Approximately 32,000 (yeast), 42,000 (fly) and 100,000 events (*Diplonema*) were collected. Signals from contaminating cytosolic material were excluded based on the scatter signal. The measurements were performed by the flow-cytometry facility of the Institute for Research in Immunology (IRIC) at the Université de Montréal.

Pulsed-field gel electrophoresis and Southern blot hybridization

Separation of chromosomes by pulsed-field gel electrophoresis (PFGE) was performed using a CHEF DR-II Chiller System (Bio-Rad). Briefly, whole cells from an exponential-phase culture were embedded in a 0.7% (w/v) low-gelling temperature agarose solution (50 mM EDTA pH8.0), incubated overnight at 50 °C in a lysis solution (0.45 M EDTA pH8.0, 1% N-lauroylsarcosine, 1 mg/mL proteinase K), and then stored at 5 °C (in 0.45 M EDTA pH8.0, 0.1% N-lauroylsarcosine). DNA (from ~10^8 cells per lane) was then separated in a 1% (w/v) agarose gel in 0.5× TBE buffer at 10 °C and two alternative separation programs (**Supplementary Table S1**). DNA was then blotted onto a nylon membrane (Zeta-Probe, Bio-Rad) *via* capillary alkaline transfer (Brown 2001) and fixed for 60 min at 80 °C. As a probe, we used the oligonucleotide dp350 (**Supplementary Table S2**) that targets telomeric repeats. Hybridization in ULTRAhyb-oligo buffer (Ambion) with radioactively labeled oligo-deoxynucleotides, washing, and detection were performed as detailed previously (Valach et al. 2014).

Electron microscopy and fluorescence microscopy

For fluorescence and electron microscopy, cell growth took place in an artificial sea salt solution (Sigma; 36 g/L) enriched with 1% (v/v) heat-inactivated fetal bovine serum (FBS) and 0.1% (w/v) tryptone (TASHYREVA *et al.* 2018b). Cells were collected at the exponential growth phase by centrifugation at 3,000×g for 15 min and were frozen with a Leica EM PACT2 high-pressure freezer (Leica Microsystems). Successive ultra-thin (100 nm) serial sections were prepared as described elsewhere (YURCHENKO *et al.* 2014). Observations were performed using a JEOL 7401-F microscope at an accelerating voltage of 6 kV. High-resolution micrographs of nuclei were aligned to build 3D reconstructions using the Amira software (Thermo Fisher). Sphericity of nucleus and chromatin was calculated based on their volume and surface area (nucleus ~32

μm², chromatin ~87 μm²). For fluorescence microscopy, cells were harvested from cultures grown for 72 h, fixed for 30 min with 4% paraformaldehyde in artificial seawater, washed in PBS, and allowed to adhere onto poly-L-lysine coated slides. The samples were subsequently mounted in ProLong Gold antifade reagent (Life Technology) containing 4',6-diamidino-2-phenylindole (DAPI) and examined by an Olympus BX53 fluorescence microscope.

Expansion microscopy

Cells were grown as for the other microscopy applications (see above). Typically 10^6 cells were pelleted by centrifugation for 5 min at 800×g, followed by a wash with seawater. For fixation, the cells were re-suspended in seawater and transferred onto a coverslip. The fixation solution containing 4% formaldehyde and 4% acrylamide in seawater was added, and the cells were incubated overnight at room temperature, then washed in seawater. For gelation, the monomer solution containing 19% sodium acrylate, 10% acrylamide, and 0.1% N, N'-methylene bisacrylamide in PBS was mixed with N, N, N0, N0-tetramethylethylenediamine and ammonium persulfate (at a final concentration of 0.5% each). The mixture was quickly transferred onto parafilm in a wet chamber. Coverslips with the cells facing down were placed on the drop of the monomer solution and incubated for 5 min. Subsequently, the wet chamber was transferred to 37 °C and incubated for 30 min to allow gel polymerization. The specimens were then transferred to a well of a 12-well plate containing 1 mL of the denaturation buffer (50 mM Tris-HCl pH9.0, 200 mM NaCl, 200 mM SDS), detached from the parafilm, and then moved to a microcentrifuge tube with additional denaturation buffer and incubated for 1 h at 95 °C. The denatured gels were transferred to Petri dishes and expanded by three 20-min incubations with 15 mL of water. An approximately 10 × 10-mm piece of the gel was cut per staining, performed in the dark while gently rocking. Primary (mouse anti-tubulin) and secondary (anti-mouse) antibodies were diluted in ExM blocking buffer (2% bovine serum albumin in PBS) at 1:50 and 1:1,000 ratios, respectively. The gels were incubated overnight at room temperature and washed 3× for 20 min with water (first with primary, then with secondary antibodies). Selected specimens were also incubated for 30 min at room temperature with 10 μg/mL of 40 ,6-diamidino-2-phenylindole (DAPI) in PBS. Finally, the gels were washed 5× for 15 min with 4 mL of water. A stained piece of the gel was transferred to the center of a glass-bottom dish coated with poly-L-lysine. Specimens were imaged using a Leica TCS SP8 confocal microscope with an HC PL apochromatic 150x/NA 1.40 oil immersion objective. Excitation was performed with a 405 nm diode laser (50 mW) in the case of DAPI and Hoechst 33342 and a 488 nm solid-state laser (20 mW) in the case of Alexa Fluor 488; emissions were detected using hybrid detectors (HyD). Z-stacks were acquired with a step size of 100 nm (without averaging). The pixel size and dwell time were between 52 and 97 nm and 400 ns, respectively. The size of the pinhole was adjusted based on the signal strength, but was typically around 0.4 Airy unit to improve resolution.

Supplementary Table S1. Parameters of PFGE separation programs.

Program	Step	Switch duration	Angle	Electric field strength	Run time
#1	1	1,200 s	94°	2.0 V/cm	30 h
	2	1,500 – 12,000 s	106°	1.5 V/cm	90 h
	3	120 – 480 s	120°	2.5 V/cm	22 h
#2	1	120 – 500 s	100°	3.5 V/cm	10 h
	2	500 s	106°	3.0 V/cm	40 h

Supplementary Table S2. Oligonucleotides used in this study.

Oligonucleotide	Sequence (5' → 3')	Target
dp350	CAAACCCGCAAACCCGCAAACCCGCA	telomeric repeat
dp236	ACACGCACCCTATGAGCTTAGCATTGGTAGT	cox1-m1

ACKNOWLEDGEMENTS

We thank Shona Teijeiro (UdeM) for performing the flow-cytometry experiments, Dr. Eva Doleželová (Institute of Parasitology, Biology Centre, Czech Academy of Sciences, Ceske Budejovice, Czech Republic) for critical discussion of the results, Dr. Stephen Michnick, Dr. Vincent Archambault, and Louise Cournoyer for providing cell lines and strains that were used as references in the flow cytometry experiments, and Monique Vasseur (all from UdeM) for assistance in nuclei staining.

AUTHOR CONTRIBUTIONS

Conceptualization, Data curation, Formal analysis, Investigation, Visualization, Writing, original draft – G.P., P.T., G.B., M.V.; **Writing, review & editing –** all co-authors.

REFERENCES

Angeler, D. G., A. N. Muellner and M. Schagerl, 1999 Comparative ultrastructure of the cytoskeleton and nucleus of *Distigma* (Euglenozoa). Eur J Protistol 35**:** 309-318.

Breglia, S. A., N. Yubuki, M. Hoppenrath and B. S. Leander, 2010 Ultrastructure and molecular phylogenetic position of a novel euglenozoan with extrusive episymbiotic bacteria: *Bihospites bacati* n. gen. et sp. (Symbiontida). BMC Microbiol 10**:** 145.

Elias, M. C., M. Faria, R. A. Mortara, M. C. Motta, W. de Souza *et al.*, 2002 Chromosome localization changes in the *Trypanosoma cruzi* nucleus. Eukaryot Cell 1**:** 944-953.

Ersfeld, K., 2011 Nuclear architecture, genome and chromatin organisation in *Trypanosoma bruce*i. Res Microbiol 162**:** 626-636.

Kiseleva, E., T. D. Allen, S. A. Rutherford, S. Murray, K. Morozova *et al.*, 2007 A protocol for isolation and visualization of yeast nuclei by scanning electron microscopy (SEM). Nat Protoc 2**:** 1943-1953.

Lukeš, J., B. Kaur and D. Speijer, 2021 RNA editing in mitochondria and plastids: weird and widespread. Trends Genet 37**:** 99-102.

Lukeš, J., R. Wheeler, D. Jirsová, V. David and J. M. Archibald, 2018 Massive mitochondrial DNA content in diplonemid and kinetoplastid protists. IUBMB Life 70**:** 1267-1274.

Marande, W., J. Lukeš and G. Burger, 2005 Unique mitochondrial genome structure in diplonemids, the sister group of kinetoplastids. Eukaryot Cell 4**:** 1137-1146.

O'Donnell, E. H. J., 1965 Nucleolus and Chromosomes in *Euglena gracilis*. Cytologia 30**:** 118-154.

Patterson, D. J., 1999 The diversity of eukaryotes. Am Nat 154**:** S96-s124.

Porter, D., 1973 *Isonema papillatum* sp. n., a new colorless marine flagellate: a light- and electronmicroscopic study. J Protozool 20**:** 351-356.

Prokopchuk, G., D. Tashyreva, A. Yabuki, A. Horák, P. Masařová *et al.*, 2019 Morphological, ultrastructural, motility and evolutionary characterization of two new Hemistasiidae species. Protist 170**:** 259-282.

Roy, J., D. Faktorová, J. Lukeš and G. Burger, 2007 Unusual mitochondrial genome structures throughout the Euglenozoa. Protist 158**:** 385-396.

Schenkman, S., S. Pascoalino Bdos and S. C. Nardelli, 2011 Nuclear structure of *Trypanosoma cruzi*. Adv Parasitol 75**:** 251-283.

Tashyreva, D., G. Prokopchuk, J. Votýpka, A. Yabuki, A. Horák *et al.*, 2018a Life cycle, ultrastructure, and phylogeny of new diplonemids and their endosymbiotic bacteria. MBio 9**:** e02447-02417.

Tashyreva, D., G. Prokopchuk, A. Yabuki, B. Kaur, D. Faktorová *et al.*, 2018b Phylogeny and morphology of new diplonemids from Japan. Protist 169**:** 158-179.

Triemer, R. E., 1992 Ultrastructure of mitosis in *Diplonema ambulato*r Larsen and Patterson (Euglenozoa). Eur J Protistol 28**:** 398-404.

Triemer, R. E., and M. A. Farmer, 1991 An ultrastructural comparison of the mitotic apparatus, feeding apparatus, flagellar apparatus and cytoskeleton in euglenoids and kinetoplastids. Protoplasma 164**:** 91-104.

Valach, M., S. Moreira, G. N. Kiethega and G. Burger, 2014 Trans-splicing and RNA editing of LSU rRNA in *Diplonema* mitochondria. Nucleic Acids Res 42**:** 2660-2672.

Vickerman, K., and T. M. Preston, 1970 Spindle microtubules in the dividing nuclei of trypanosomes. J Cell Sci 6**:** 365-383.

Wassie, A. T., Y. Zhao and E. S. Boyden, 2019 Expansion microscopy: principles and uses in biological research. Nat Methods 16**:** 33-41.

Yubuki, N., V. P. Edgcomb, J. M. Bernhard and B. S. Leander, 2009 Ultrastructure and molecular phylogeny of *Calkinsia aureus*: cellular identity of a novel clade of deep-sea euglenozoans with epibiotic bacteria. BMC Microbiol 9**:** 16.

Yurchenko, V., J. Votýpka, M. Tesarová, H. Klepetková, N. Kraeva *et al.*, 2014 Ultrastructure and molecular phylogeny of four new species of monoxenous trypanosomatids from flies (Diptera: Brachycera) with redefinition of the genus *Wallaceina*. Folia Parasitol (Praha) 61**:** 97-112.

2. Assembly and annotation of the nuclear genome and transcriptome of *Diplonema papillatum*

INTRODUCTION

Despite the recent development of powerful algorithms, the *de novo* assembly of genomes can be difficult. The main challenge encountered when assembling the *D. papillatum* nuclear genome was the large proportion of long repeat regions. In contrast, the assembly of transcripts was straightforward, because these repeat regions are not transcribed (at least in the conditions under which the protist was cultured). Structural annotation was also complicated by repeats, because RNA-Seq reads that otherwise assist in the selection of the most probable gene structure may be misleading if they can align with multiple locations in the genome. To assess the correctness of gene models predicted by the automated pipeline, the three largest contigs were manually curated by an expert. Finally, predicted protein-coding genes were assigned a molecular function by annotation transfer from model organisms to the examined species based on sequence similarity. Due to the high divergence of *Diplonema* sequences and the absence of closely related model organisms, we are left without any functional information for more than half of the gene models.

RESULTS AND DISCUSSION

Genome assembly and polishing

The overall approach involved five steps: (i) generation of short and long reads; (ii) correction of long reads, which have a high error rate, with short reads; (iii) separate assembly of the two read types; (iv) merging of contigs from the two assemblies; and (v) detection and splitting of misassembled contigs.

More specifically, we generated 462 million pairs of short reads (Illumina) and ~725,000 long reads (PacBio) totalling 126.4 Gbp raw data (**Supplementary Table S3**). Short and long reads were assembled separately with the Celera Assembler (MYERS *et al.* 2000) and Canu (KOREN *et al.* 2017). The 'primary merged assembly' was compiled by complementing the PacBio contigs with those contigs from the Celera assembly that are not included in the former, and the resulting contig set was deduplicated to yield the 'primary merged deduplicated (PMD) assembly' (**Supplementary Figure S5**). We noted a low rate of contig removal by the latter step, which is indicative for a genome of very low heterozygosity (i.e., haploid or autodiploid; see **Supplementary Information**: Section 3. The ploidy level of *Diplonema papillatum*).

The PMD assembly was further processed to split contigs that arose by incorrectly assembled reads. Such erroneous joining is due to a low percentage of chimeric reads, i.e., reads originating from unrelated DNA fragments that were ligated together during library construction. Erroneous joining sites were detected by low read coverage after aligning short and long reads with the contigs of the PMD assembly. Unless high-confidence transcripts spanned such a trough in coverage of both short and long reads, contigs were split at these positions and contig portions smaller than 100 bp removed (**Supplementary Figure S6**). Note that a more conventional scaffolding approach, which generally produces a more contiguous assembly, was not implemented, because such assemblies contained an even higher number of obviously chimeric contigs than the PMD assembly.

The final product, the *D. papillatum* nuclear genome assembly version 1.0 described here, comprises 6,181 contigs ≥200 bp, has a length of 280,293,864 bp and an N50 value of 190,080 bp (**Supplementary Table S4**).

Supplementary Table S3. Libraries and reads used in this study.

Library name	Material	Prep kit (approx. insert size)	Techno-logy[a]	Service provider	SRA ID	Nr. of raw reads[b]	Read Length
Dp-nucDNA	Total DNA	Illumina TruSeq DNA (2–8 kbp)	HiSeq MP	Genome Quebec	/[c]	17,772,001	150 nt
Dp_301113	Total DNA	Illumina TruSeq DNA (0.5 kbp)	MiSeq PE	Genome Quebec	SRR21741423	5,740,630	250 nt
Dp_301113-2nd	Total DNA	Illumina TruSeq DNA (0.5 kbp)	MiSeq PE	Genome Quebec	SRR21741422	20,139,574	250 nt
Dp_HiSeq_Dp_gDNA	Nuclear DNA	Eurofins (3 kbp)	HiSeq PE	Eurofins	SRR21741421	13,305,159	100 nt
Dp_MiSeq_Dp_gDNA	Nuclear DNA	Eurofins (8 kbp)	MiSeq PE	Eurofins	SRR21741420	18,367,804	250 nt
PacBio-20kbp	Size-selected DNA (>20 kbp)	BluePippin+SMRTbell (PacBio Large Insert)	Sequel	Genome Quebec	SRR21741419	149,527	2,500 nt[d]
Dp_PacBio	Size-selected DNA (>20 kbp)	BluePippin+SMRTbell (PacBio Large Insert)	Sequel II	Takara Bio	SRR21741418	575,764	8,500 nt[d]
PA	Poly(A) RNA	Epicentre ScriptSeq RNA (0.2 kbp)	HiSeq PE SS	Macrogen Korea	SRR21741417	30,618,141	100 nt
DPA2	Poly(A) RNA	Illumina TruSeq RNA (0.25 kbp)	HiSeq PE SS	Macrogen Korea	SRR21741416	261,280,954	125 nt
Dp_RNASeq_Dpa	Poly(A) RNA	Illumina TruSeq RNA (0.25 kbp)	MiSeq PE SS	Novogene	SRR21741415	67,496,825	150 nt

[a] MP, mate-paired (long inserts); PE, paired-end (short inserts); SS, strand-specific.
[b] Read pairs in the case of Illumina reads; single reads in the case of PacBio reads.
[c] Not used in the assembly.
[d] Average read length (up to ~45,000 nt).

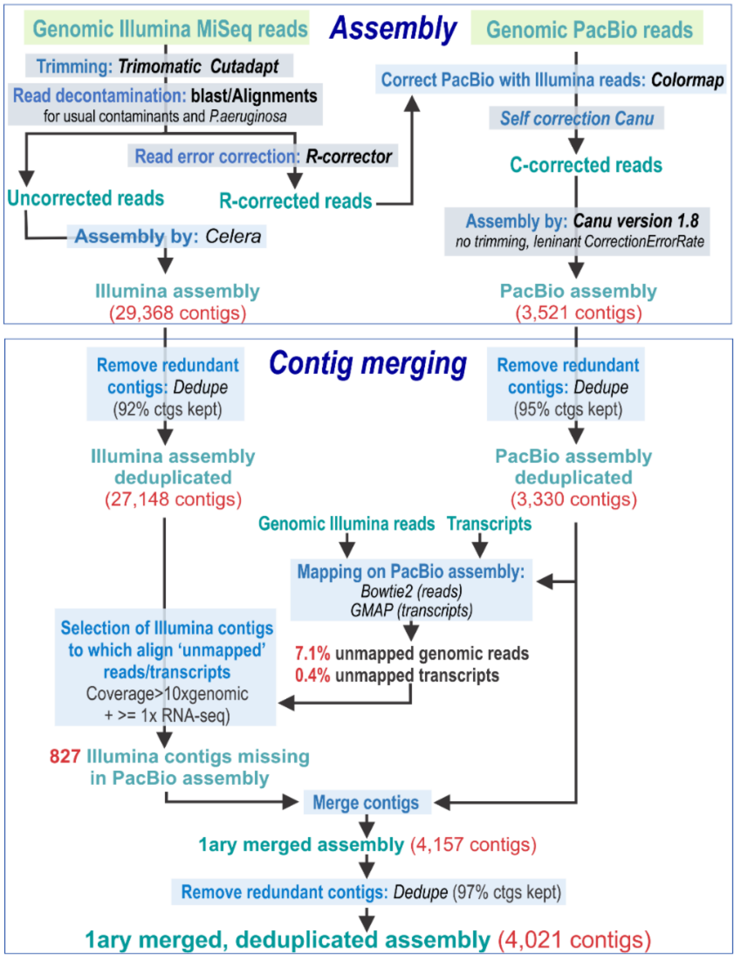

Supplementary Figure S5. Procedure of nuclear genome assembly and contig merging. The procedure consists of the '*Assembly*' phase in which short (Illumina MiSeq) and long (PacBio) reads are assembled separately. In the subsequent '*Contig merging*' phase, genomics reads and transcripts (the latter from RNA-Seq assembly) were identified that do not align to the PacBio assembly. Then, the contigs in the Illumina assembly were selected to which these sequences could be mapped. These Illumina contigs were merged with the PacBio contigs and then deduplicated. Subsequent polishing steps are depicted in Supplementary Figure S6.

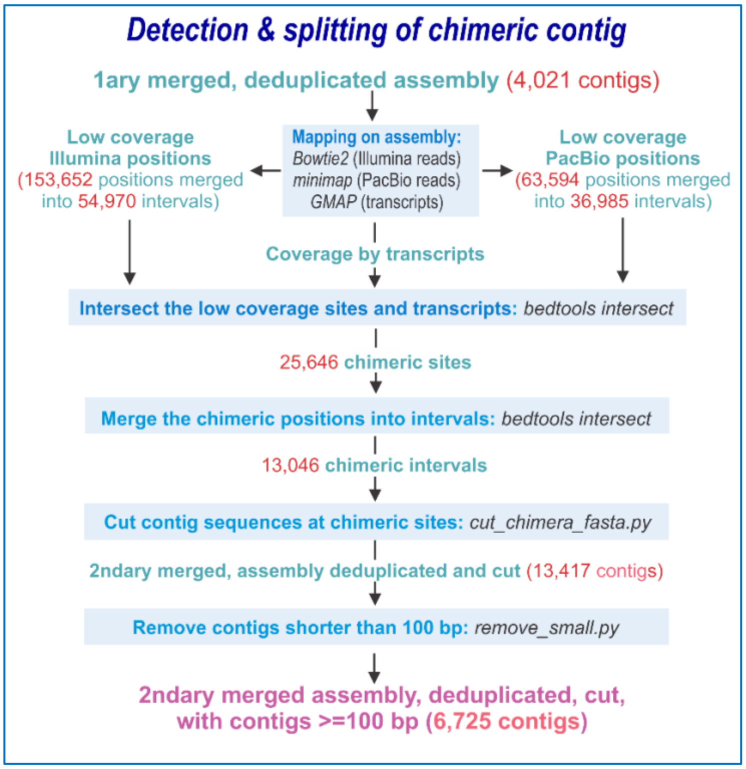

Supplementary Figure S6. Procedure of assembly polishing. Potentially mis-assembled (chimeric) contigs are recognized by a sudden drop in read coverage. The screen for low-coverage (LC) genomic positions is performed separately for short and long reads. Adjacent LC positions are merged into intervals. Then, shared LC intervals from Illumina and PacBio contigs are determined and compared with the locations where transcripts align to the genome. Only LC intervals that are not spanned by transcripts are further processed. In the next step, adjacent LC intervals are again combined, and the contigs are then split at the interval borders. Contig pieces shorter than 100 bp are eliminated to yield the final assembly.

Transcriptome assembly

Transcript sequences were obtained by *de novo* assembly of about 645 million reads from the two strand-specific poly-A RNA libraries PA and DPA2 (see **Supplementary Table S3**), using the Trinity software. The inferred transcriptome contains nearly 200,000 contigs (transcripts) with a cumulative length of ~16 Mb (**Supplementary Table S4**). Transcript sequences assisted in the structural genome annotation, and a subset of the sequences referred to as ‘high-confidence transcriptome’ (mRNAs) were employed for detecting mis-joined contigs (see section Genome assembly). The nearly 22,000 high-confidence mRNAs were selected based on the presence of a Spliced-Leader (SL) sequence at their 5′ end and a minimum overall read coverage of 100.

As a measure of genome completeness complementary to the BUSCO benchmarking (see below), we determined the rate of mapping transcriptome-derived reads to the genome sequence. Out of 174,212,093 RNA-Seq reads >40 nt (after contaminant filtering, as well as quality and adaptor trimming), 96.8% mapped onto the genome. Of the remaining reads, 2,608,091 (representing 1.5%) mapped exclusively onto the transcriptome assembly (usually onto transcripts split across multiple genomic contigs), while 2,930,982 reads (representing 1.7%) could not be mapped onto either assembly. Many of the unmapped reads contained homopolymer tracts (e.g., poly-A) and low-complexity repeats. These observations suggest that the *D. papillatum* genome assembly version 1.0 contains essentially all actively transcribed regions.

Supplementary Table S4. Statistics of genome and transcriptome assemblies.

Assembly	Total length	Nr. of contigs	Contig size range	N50	Average A+T content (range)
Genome Dp_v1.0	280,293,864 bp	6,181	200 - 1,009,103 bp	190,080	44.8% (20.0%-75.6%)
Transcriptome (*de novo*)	161,272,633 nt	194,546	182 - 43,150 nt	/	43.8% (16.4%-73.3%)
High-confidence transcriptome[a]	46,055,449 nt	21,747	298 - 43,118 nt	/	40.9% (22.6%-68.6%)

[a]Minimum read coverage per contig 100x; SL sequence at 5’end (see text)

Structural genome annotation

Structural genome annotation, also referred to as gene-model prediction, denotes the identification of genome regions that have the potential to code for proteins or specify structural (non-(protein-)coding) RNAs. Our automated annotation pipeline constructed about ~37,000 gene models, predominantly protein-coding gene models (**Supplementary Table S5, S6**). Most *D. papillatum* protein-gene model structures are simple, lacking introns and isoforms. Furthermore, most models are

complete with standard start/stop codons. Only a small minority appear to be incomplete either at the 5′ (~2.5%) or the 3′ (~2.5%) end, and an even smaller proportion (~1.5%) lack both start and stop codons.

To further assess the completeness of protein-coding gene models, their respective conceptually translated sequences were benchmarked against the so-called 'universal single-copy ortholog' set of proteins (BUSCO (SEPPEY *et al.* 2019)) using v10 of the Eukaryota OrthoDB (built 2019-11-20 (KRIVENTSEVA *et al.* 2019) (**Supplementary Table S7, S8**). The results indicated that 84% of models are complete with ~17% in duplicate, whereas only 4% were reported as fragmented, and 11% (29) as apparently missing. As for the models reported as duplicated, around 1/3 are exact copies and thus might be due to either recent genomic duplications or assembly errors, whereas non-identical duplicates most likely represent paralogs.

A closer inspection of the orthologs for which BUSCO reported missing matches revealed that four are also absent in other diplonemids and one is missing in the sister group, *Kinetoplastida*, whereas 16 are in fact present in *D. papillatum* but are divergent to the degree that falls short of BUSCO's cut-off criteria. Eight BUSCO Hidden Markov Models (HMMs) have inconclusive hits that would need further verification. In summary, BUSCO completeness is above 90% when excluding models known to be missing from Euglenozoa. Since detecting the remaining ≤10% of BUSCO orthologs appears to be hampered by extreme sequence divergence, potential assembly errors, or incorrect gene modelling, we consider the *D. papillatum* genome assembly version 1.0 as quasi complete.

Structural annotation also included the prediction of spliced-leader trans-splicing sites (for details see below), which were pinpointed by aligning RNA-Seq reads to the genome assembly and determining the positions where soft-clipped portions of the reads consist of the spliced leader sequence.

Supplementary Table S5. Summary of structural genome annotation.

Prediction type	Initial, fully automated annotation	Annotation partially expert-curated
Protein-coding genes	35,978	37,054
Non-coding RNAs	7,362	573
rRNA[a]	5,111[b]	151
tRNAs[c]	375	211
Small nucleolar (sno) RNAs[a]	9	9
Spliceosomal (sn) RNAs[a]	202	202
MicroRNAs	1,250	0
Other RNAs[a,e]	417	0
Total	43,340	37,627

[a] Identified by Rfam HMM
[b] Includes rRNAs fragments
[c] Identified by tRNAScan-SE
[d] includes pseudo tRNAs, and those with undetermined anticodon
[e] HDV ribozymes, rsrG Hfq binding RNA, etc.

Supplementary Table S6. Summary of protein-coding gene models.

Feature	Status	Count (percent)	Average length	Median length	Average coding length	Median coding length
Transcripts	Complete	36,763 (94%)	4,044 bp	2,424 bp	1,699 bp	1,152 bp
	Incomplete	2,354 (6%)	4,381 bp	2,070 bp	2,126 bp	1,206 bp
	Single exon	21,485 (55%)	1,823 bp	1,333 bp	1,318 bp	927 bp
	All	39,117	4,065 bp	2,409 bp	1,724 bp	1,155 bp
Exons	Initial	15,259	832 bp	516 bp	/	/
	Internal	23,016	616 bp	284 bp	/	/
	Terminal	15,113	593 bp	279 bp	/	/
	Single	21,384	1,319 bp	927 bp	/	/
	All	74,764	857 bp	472 bp	/	/
UTRs [a]	3′ UTR	21,896	1020 bp	777 bp	/	/
	5′ UTR	20,251	144 bp	66 bp	/	/
Introns		36,604	1,528 bp	671 bp	/	/

[a] Statistics were calculated based on annotated UTRs, i.e., genes that lack UTR annotations were ignored.

Supplementary Table S7. BUSCO report.

BUSCO report for assembly version 1.0	Nr. of models reported			
	Complete	duplicate	fragmented	missing
	215/255 (84.3%)	42 (16.5%)	11 (4.3%)	29 (11.4%)

Supplementary Table S8. BUSCO benchmarking of assembly completeness.

Busco category	Total (percentage)
Complete	215 (84.3%)
Complete and single-copy	173 (67.8%)
Complete and duplicated	42 (16.5%)
Fragmented	11 (4.3%)
Missing	29 (11.4%) [a]
Total	255 (100%)

[a] Manual curation showed that 16 of these are present in *D. papillatum* but divergent, and that 4 are absent from all 11 diplonemids, for which RNA-Seq data are available.

Quality assessment of the automated structural annotation

Models of genes predicted to encode a protein delineate exons, introns, and 5′ and 3′ UnTranslated Regions (UTRs). Here we assess the accuracy of predicted gene models *via* inspection by an expert. The structural annotation of the automated pipeline was validated by human curation of the three longest contigs (tig00022654_12, tig00022740_1, and tig00022679). Together these contigs represent ~1% of the total assembly and contain 319 predicted protein-coding genes (**Supplementary Table S9**). As detailed in the following, inaccuracies in automated gene-model prediction include omissions, false positives, and incorrect UTR termini and exon-intron boundaries. The underlying causes for these shortcomings will be discussed.

5′ UTRs. The nuclear gene expression of diplonemids (and kinetoplastids) relies on the addition of a short sequence called spliced leader (SL) upstream of a primary transcript containing a coding sequence (CDS) to generate the 5′ end of the mRNA (STURM *et al.* 2001; CLAYTON 2019). SL addition takes place by a trans-splicing reaction that is catalyzed by the nuclear spliceosome. Several inspected gene models were lacking a 5′ UTR annotation, which was generally due to the concealed Spliced Leader (SL) Trans-Splicing (SLTS) acceptor site upstream of the predicted ORF. Visual inspection revealed spurious, short matches of the 3′ moiety of the SL to other locations upstream of the gene, thus counteracting the diagnostic soft-clipping at the SLTS site. The RNA-Seq read-mapping algorithm considers such matches valid when the interval is flanked by the GT and AG dinucleotides to model an intron. This mapping artifact can misplace the inferred SLTS-site up to a dozen kbp upstream or impede identification of the splice site and the 5′ UTR. In all validated cases, the matches of the SL to upstream regions were short (≤6 nt), making alternative splicing unlikely.

3′ UTRs. The structural annotation algorithm was trained to place a gene's end, i.e., the 3′ terminus of its 3′ UTR, at the position where a transcript aligning with the genomic region ended. However, we observed a sharp drop in RNA-Seq read coverage downstream of many CoDing Sequences (CDSs) coinciding with low complexity or highly repetitive regions, followed further downstream by a coverage similar to the CDS. In most such instances, the region immediately downstream of the annotated CDS was predicted to contain a short ORF without a SLTS acceptor site, strongly suggesting that these ORFs were spurious. We reasoned that the fluctuating RNA-Seq read coverage indicated a 3′ UTR extending beyond the pipeline-assigned end and that the premature positioning of the gene termini was actually due to artificially shortened transcripts. Indeed, during transcriptome assembly by the Trinity software (GRABHERR *et al.* 2011), transcript sequences are not further elongated in two instances: (i) when the number of reads is high with no clear path along the assembly graph e.g., in highly repetitive regions; or (ii) when the number of reads is low, e.g., in a homopolymeric stretch that is not efficiently sequenced. The gene model DIPPA_17048 is a typical example. The region covered by the transcript sequence terminates with a succession of ~50 bp-long T, G and C homopolymeric runs forming an ~6.7 kbp-long 3′ UTR. However, downstream of the predicted end of the gene, a reasonable coverage of RNA-Seq read extends for an additional ~2.5 kbp. Thus the 3′ UTR is considered to have a total length of ~9.2 kbp, which is 2.5 kbp longer than predicted by the annotation pipeline.

Supplementary Table S9. Expert validation of gene-models from the three largest contigs.

		Total	Contig ID		
			tig00022654_12	tig00022740_1	tig00022679
Gene models	All [a]	334	131	108	95
	Before curation	319	123	104	92
	UTRs, introns corrected [b]	125	47	38	40
	New [c]	15	8	4	3
	False positive [d]	68	15	32	21
	After curation, unsupported [e]	34	10	18	6
	After curation, supported	232	107	57	68
Corrections	5′ UTR [f]	51	18	9	24
	3′ UTR [g]	101	38	32	31
	Introns	23	7	9	7
Gene models with spliced leader	Automated detection [h]	181	90	52	39
	Addit. Expert detection [i]	27	11 (10+1)	4 (1+3)	12 (3+9)
	No evidence [j]	18	6 (3+3)	1 (0+1)	11 (5+6)
SLTS sites	Genes with multiple sites [k]	30	14	11	5

[a] The sum of all processed gene models combining those prior to curation and the newly added ones.
[b] UTR boundaries and/or introns were corrected.
[c] Unrecognized by the automated annotation pipeline, but evident from transcriptome data.
[d] Models that became obsolete, because e.g., located within a newly recognized intron.
[e] Models that are typically a part of a transposable or other repetitive element and without detectable SLTS site or RNA-Seq read coverage.
[f] Generally, the UTR has been shortened (see text).
[g] In most cases, the UTR has been considerably extended (see text).
[h] Recognized by the soft-clipped spliced-leader (SL) sequence of ≥11 bp in RNA-Seq reads; only counting a single, primary site per gene model.
[i] Failure to detect the site by the automated approach was due to (i) misaligned reads, or (ii) soft-clipped SL segments of ≤10 bp.
[j] Soft-clipped SL sequences are absent, while the gene has RNA-Seq read coverage. The failure to detect the SL may be due to repeats or low-complexity regions upstream of the CDS impeding correct alignment of RNA-Seq reads. In addition, the gene's 5′ UTR region may have a low read coverage.
[k] Exclusively sites reported by the automated procedure were counted.

Repercussions of repetitive genome regions. Repeats, and tandem repeats in particular, represented a major source of difficulty for the annotation algorithm caused by the incorrect alignment of transcripts and RNA-Seq reads to the genome assembly. This problem affected all gene features, but most frequently led to the annotation of spurious introns and to missed genes. For example, two genes in a tandem arrangement were often annotated as a single gene. We also detected multiple cases where tandemly repeated portions of a CDS flanked by different UTRs or including different intron sequences were annotated as a single gene by combining the 5′ moiety of the upstream CDS and 3′ moiety of the downstream CDS. DIPPA_17054 represented such a case. Curation consisted in splitting off its 3′ portion, from which the new gene model DIPPA_70071 was generated. In all such instances the accuracy of the genome assembly in the corresponding region was verified by inspecting the mapped PacBio reads.

Missing gene models. Rarely, protein-coding genes were not recognized by the annotation pipeline despite unequivocal evidence from transcript (RNA-Seq) data, and the absence of other issues such as complex repeats. Missing models tended to cluster within contigs; the largest cluster comprised 11 consecutive unidentified gene models in contig tig00023309_11. The reason for this problem remains unclear. Nevertheless, most of these omissions could be rectified by a post-processing procedure that used transcripts carrying a SL sequence at their 5′ ends as a lead.

Reconstructed transcripts absent from the genome. A total of 18,480 reconstructed transcripts were found to contain ORFs preceded by a splice-leader sequence. Surprisingly, a considerable portion (~20%) of those transcripts were not identified in the genome assembly. Genome mis-assemblies may partially explain this problem since subsequences of some missing transcripts were identified across two or more contigs. For downstream analyses, we established a comprehensive sequence collection by combining into a non-redundant set all the missing transcripts with the manually curated set and all remaining protein-coding genes automatically generated by the pipeline.

Genes split by genomic sequence gaps. An issue encountered in contigs other than the three longest were genes artificially split within their intron by a sequence gap in the scaffold. This gap led the annotation pipeline either to infer an incorrect CDS terminus, or to omit the annotation altogether. In all investigated cases, the problem was due to highly repetitive sequence elements in the corresponding introns.

Alternatively spliced introns. Only a few genes with alternatively spliced introns were identified in the validated contigs, at least under the growth conditions used to prepare the RNA-Seq libraries. Therefore, a random sample of intron-containing genes models were examined (DIPPA_00001 to DIPPA_03296). Among these, 11 cases had convincing support by RNA-Seq data for alternative splicing. Several genes with confirmed alternative splicing combine more than one splicing type. For example, the expression of gene DIPPA_00069 (tig00013476_1) involves both exon skipping and intron retention, while that of gene DIPPA_03285 (tig00022668_1) implicates in addition alternative splice-site selection.

Conclusion. The performance of the structural annotation pipeline used in this study ranks among the best available tools. Still, among the set of expert-inspected gene models, about ¼ had incorrectly placed UTRs or (more rarely) exon/intron boundaries, another ¼ were false positives, and ~4% were not retrieved. As discussed above, these shortcomings are mostly due to the high proportion of long sequence repeats in the *Diplonema* nuclear genome. While the automated annotation provides a fair picture of the *Diplonema* nuclear genome, thorough expert validation is warranted should seemingly unusual or unique genes and gene features be encountered.

Functional genome annotation

Protein-coding genes. As shown in Supplementary Figure S7, about 51% of all gene models were assigned functional information by either sequence similarity to the SWISS-PROT database and the downloaded GenBank accessions, or probabilistic HMM searches against Pfam v31.0, or both. The products of the gene models without SWISS-PROT information were labelled as 'hypothetical proteins'. Most models with assigned functional information have significant hits by both similarity to protein sequence databases and to protein domains. As there are currently few publicly available sequences from Discoba species, and those that are available come mostly from derived kinetoplastids (e.g., *Trypanosoma*), neither taxonomically broad nor Discoba-specific profile HMMs can be built for all nucleus-encoded proteins. This implies that functional information transfer must be made through less sensitive searches, notably searches for sequence similarity and conserved protein domains.

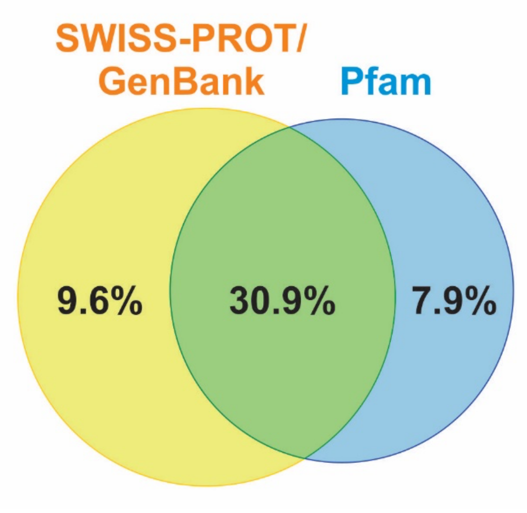

Supplementary Figure S7. **Summary of gene models assigned a function.** Functional information was assigned to a total of 19,078 protein-coding gene models. SWISS-PROT/GenBank (yellow plus green intersection) represents the portion with explicit information about the gene product. Pfam (blue plus green intersection) represents the portion that includes a conserved protein domain.

Non-coding (structural) RNAs. Transfer RNA genes were searched with tRNAscan-SE, and rRNAs, snoRNAs and microRNAs with Hmmerscan using profile HMMs from Rfam (GRIFFITHS-JONES *et al.* 2003). The employed version of the annotation procedure did not report if a gene is incomplete, which explains the large number of rRNA gene models initially reported, which was corrected secondarily (see **Supplementary Table S5**).

METHODS

Genome assembly

Read cleaning. For the genome assembly, we first cleaned Illumina and PacBio reads. We used cutadapt v1.2.1 (http://journal.embnet.org/index.php/embnetjournal/article/view/200) on MiSEQ reads to clip adaptors (-e 0.1, -O 10 -m 20) and then trimmed low-quality sequences at the ends of reads (-q 20 -m 20). Reads with more than three non-defined bases ('N') in a row were discarded. PacBio reads were self-corrected and quality trimmed with Canu (KOREN *et al.* 2017) and then corrected with CoLoRMap (HAGHSHENAS *et al.* 2016) (default options) by mapping onto these long reads a set of Illumina reads equivalent to 50X of the genome coverage. Using the CoLoRMap program suite, we applied a scrupulous two-step correction, first using the shortest-path algorithm and second the One-End-Anchor based algorithm. The quality of libraries was evaluated along the cleaning process with Prinseq v.0.20.4 (SCHMIEDER AND EDWARDS 2011) and FastQC (EDWARDS, 2010, https://www.bibsonomy.org/bibtex/2b6052877491828ab53d3449be9b293b3/ozborn).
Read decontamination. Illumina and PacBio reads were screened for potential contaminations with sequences from other organisms and organelles. For that, we generated a crude assembly. Illumina reads were assembled with the Celera package (DENISOV *et al.* 2008) executed by the script runCA release 8.3.rc2 (http://wgs-assembler.sourceforge.net/wiki/index.php/RunCA), and long reads with Canu. We downloaded 16S and 18S rRNA sequences from the SILVA database (QUAST *et al.* 2013) (file SILVA_128_SSURef_Nr99_tax_silva.fasta) and performed a BLAST search against the provisional assemblies, shaft contigs and remaining non-assembled reads. We selected all hits with an E-value larger than 1.0e-2, a length above 300 bp, and a similarity >80%. Only four species remained after the filtering: *Pseudomonas fluorescens*, an uncultured *Bacillus* sp., *Candidatus amoebophilus*, and *Streptococcus agalactiae*. To the list of possible contaminants were further added the phage PhiX, which is used as a spike-in during sequencing, *E. coli* and *Homo sapiens*. The complete genome sequence of all contamination candidates was downloaded from NCBI. *Diplonema* reads from all genomic libraries were mapped onto the 'contaminant' genome sequences with Bowtie2 v.2.3.4 (LANGMEAD AND SALZBERG 2012) in local mode (--local), and aligning reads (plus their mates) were discarded. As a final decontamination step, we removed reads originating from the mitochondrial genome. Because previous analyses suggested that some mitochondrial sequences may be present in the nuclear genome (NUMTs), we discarded read pairs where both mates aligned with mitochondrial sequences (GenBank acc. Nos. EU123536-8 and HQ28819-33) after mapping with Bowtie2. Among unpaired reads, we removed those whose sequence identity with mtDNA was above 95%.
Genome assembly. Canu was used with parameters specifying the expected error rate and not allowing the trimming of read ends (errorRate=0.035, corMinCoverage=0). For Celera we used parameters (defined in the specification file) to fit the memory and threads available on our server and to set error correction thresholds (cnsErrorRate=0.10, ovlErrorRate=0.10). Duplicated contigs were removed with dedupe2.sh (https://jgi.doe.gov/data-and-tools/bbtools/bb-tools-user-guide/dedupe-guide/, http://sourceforge.net/projects/bbmap/), setting the parameters as follows: maxedits=10 (allows up to 10 substitutions or indels), minidentity=90 (absorbs contained sequences with at least 90% identity), findoverlap=t (finds overlaps between contigs) and cluster=t (groups overlapping contigs into clusters); the other parameters were default. The command used was:

```
$ dedupe2.sh in=genome.fasta out=genome_dedup.fasta threads=4 absorbrc=t absorbmatch=t absorbcontainment=t
overwrite=true maxedits=10 minidentity=90 findoverlap=t cluster=t outd=genome_del.fasta
```

As expected, the long-read (Canu) assembly was more contiguous, while the short-read assembly (Celera) was more complete as assessed by the number of transcripts mapped to the contigs.

Merging of the short- and long-read assemblies. The two assemblies were merged by adding to the long-read assembly those contigs that were exclusively present in the short-read assembly. To identify contigs missing in the long-read assembly, we mapped short reads and selected transcripts against the long-read assembly. The mapping of the Illumina reads was performed using Bowtie2 with specific parameters (--end-to-end –no-unal –un-conc –no-mixed) to extract the unmapped reads.

1. Build genome index and map reads:

```
$ bowtie2-build -f PacBioAssembly_dedup.fasta PacBioAssembly_dedup
$ bowtie2 --end-to-end --no-unal -p 40 --un-conc MiSeq.unmapped --no-mixed -x PacBioAssembly_dedup -1
MiSeqReads_1.fastq.gz -2 MiSeqReads_2.fastq.gz -U MiSeqReads_S.fastq.gz 1> MiSeqReads.mapped.sam 2>
```

```
MiSeqReads.mapped.log | sambamba view -S -f bam /dev/stdin | sambamba sort /dev/stdin -o
MiSeqReads.mapped.bam ; sambamba index MiSeqReads.mapped.bam
```

Transcripts were mapped to the long-read assembly, and unmapped transcripts collected.

1. Build genome index and map transcripts with the split-read aligner gmap version 2017-11-15 (WU AND WATANABE 2005):

```
$ gmap_build -D . -d PacBioAssembly_dedup PacBioAssembly_dedup.fasta > gmapbuild.out 2>&1
$ gmap --format gff3_gene --nofails -t 12 --min-identity=0.90 --min-intronlength=20 --gff3-add-
separators=0 -D . -d PacBioAssembly_dedup/ transcripts.fasta > ranscrpits.mapped.gff3 2>
transcripts.mapped.log
```

2. Extract the complete transcripts list:

```
$ grep ">" transcripts.fasta | cut -d " " -f 1 | cut -d ">" -f2 | sort > transcripts.list
```

3. Extract the list of mapped transcripts:

```
$ awk 'NR>2 {if($3=="gene") {split($9, a, ";"); split(a[1], b,"="); split(b[2], c,"."); print c[1]}}'
transcripts.mapped.gff3 | sort | uniq > transcripts.mapped.list
```

4. Extract the list of unmapped transcripts:

```
$ awk 'NR==FNR{a[$0]=1;next}!a[$0]' transcripts.mapped.list
 transcripts.list > transcripts.unmapped.list
```

5. Retrieve transcripts that do not align with long-read contigs:

```
$ xargs samtools faidx file.fasta < transcripts.unmapped.list > transcripts.unmapped.fasta
```

To identify the short-read contigs that were absent from the long-read assembly, we mapped the Illumina reads that did not align with the long-read assembly, against the short-read assembly.

1. Build genome index and map reads with Bowtie2 in a way that labels non-primary-mapping reads. Then extract a list of mapped reads sorted by the number of occurrences per contig:

```
$ bowtie2-build -f MiSeqAssembly_dedup.fasta MiSeqAssembly_dedup
$ bowtie2 -p 40 -x MiSeqAssembly_dedup -1 MiSeq.unmapped.1.fastq.gz -2 MiSeq.unmapped.2.fastq.gz --no-unal
-k 2 1> MiSeqReads.unmapped.mapped.sam 2>Dp_Mi_M-B_ctg_dedup-unmapped.log
```

From the above short reads, we removed the non-primary mapped Illumina reads and generated a list of reads aligning to the short-read (but not the long-read) assembly and then sorted them by the number of occurrences per contig.

1. Remove non-primary mapping reads:

```
$ samtools view -Sh -F 256 MiSeqReads.unmapped.mapped | grep -v "XS:i:" >
MiSeqReads.unmapped.mapped.no_mult.sam
```

2. Generate the sorted list of Illumina reads:

```
$ grep -v '^@' MiSeqReads.unmapped.mapped.no_mult.sam | cut -f 3 | sort | uniq -c | sort -rn >
nbReadsMappedToMiSeqCtgs.list
```

Similarly, we mapped the transcripts not aligning with the long-read assembly onto the short-read assembly. For that step, we used gmap. Subsequently, we generated a list of transcripts that aligned with the short-read (but not the long-read) assembly, sorted by the number of occurrences per contig.

1. Build genome index and map transcripts:

```
$ gmap_build -D . -d MiSeqAssembly_dedup  MiSeqAssembly_dedup.fasta > gmapbuild.out 2>&1
$ gmap --format gff3_gene --nofails -t --min-identity=0.90 --min-intronlength=20 --gff3-add-separators=0 -
D . -d MiSeqAssembly_dedup/ transcripts.unmapped.fasta > transcripts.unmapped.mapped.gff3 2>
transcripts.unmapped.mapped.log
```

2. Generate the sorted list of transcripts:

```
$ grep 'gene' file.gmap.gff3 | grep -v '^#' | cut -f 1 | sort | uniq -c | sort -rn >
nbTranscriptsMappedToMiSeqCtgs.list
```

Based on the above outputs, we then selected short-read contigs to be added to the long-read assembly by applying two criteria: coverage by either (i) one or more transcripts, or (ii) more than 10× coverage of Illumina reads on a given contig. This was performed by the following seven steps.

1. Combine the lists short reads aligning to the short-read (but not the long-read) assembly:

```
$ awk 'NR==FNR { split($0, s, " "); a[s[2]]=s[1]"\t"s[2]; next } {split($0, ss, " "); if(ss[2] in a)
{print ss[1]"\t"ss[2]"\t"a[ss[2]]} else {print ss[1]"\t"ss[2]} }' nbTranscriptsMappedToMiSeqCtgs.list
nbReadsMappedToMiSeqCtgs.list > nbReads+TranscriptsMappedToMiSeqCtgs.list
```

2. Count the length of each contig in the short-read assembly with the in-house script ‘cn’:

```
$ cn MiSeqAssembly_dedup.fasta > MiSeqAssembly_dedup.cn
```

3. Add counts to the file generated in step 1 and arrange data in four columns: NameOfCtg, LengthOfCtg, NbReads, NbTranscripts

```
$ awk 'NR==FNR { split($9, s, "="); a[$1]=s[2]; next } {if($2 in a) {print $2"\t"a[$2]"\t"$1"\t"$3} }'
MiSeqAssembly_dedup.cn nbReads+TranscriptsMappedToCtgs.list >
ctgLengt+nbReadsMappedToCtgs+nbTranscripts.list
```

4. Add in the 1st column the coverage calculated by NbReads*200/(LengthCtg+1) (Average of 200 bp per Illumina read):

```
$ awk '{print ($3*200)/($2+1)"\t"$0 >> "ctgLengt+nbReadsMappedToCtgs+nbTranscripts+cov.list"}'
ctgLengt+nbReadsMappedToCtgs+nbTranscripts.list
```

5. Parse the file generated in 4. To retain only contigs with at least one transcript (unmapped_transcript) mapped or 10x short-read coverage:

```
$ awk '$1>10 || $5 > 0 ' ctgLengt+nbReadsMappedToCtgs+nbTranscripts+cov.list >>
ctgLengt+nbReadsMappedToCtgs+nbTranscripts+cov_covgt10_transcgt0.list
```

6. Create the list of short-read contigs absent from the long-read assembly:

```
$ awk '{print $2}' ctgLengt+nbReadsMappedToCtgs+nbTranscripts+cov_covgt10_transcgt0.list >
miseq_ctg_to_extract.list
```

7. Extract the contig sequences:

```
$ xargs samtools faidx MiSeqAssembly_dedup.fasta < miseq_ctg_to_extract.list >
MiSeqAssembly_dedup_missingctg.fasta
```

Contigs from the short-read assembly that were absent from the long-read assembly were combined with those from the long-read assembly and, after merging, the assembly was deduplicated.

1. Concatenate short-read contigs missing in long-read assembly:

```
$ cat MiSeqAssembly_dedup_missingctg.fasta PacBioAssembly_dedup.fasta > merged_genome_assembly.fasta
```

2. Deduplicate assembly:

```
$ dedupe2.sh in=merged_genome_assembly.fasta out=merged_genome_assembly_dedup.fasta threads=40 absorbrc=t
absorbmatch=t absorbcontainment=t overwrite=true maxedits=10 minidentity=90 findoverlap=t cluster=t
outd=merged_genome_assembly_del.fasta
```

Correction of mis-junctions. We realized that both assemblies contained incorrectly assembled contigs (referred to as chimeric contigs, or briefly "chimera"). To detect mis-junctions, we screened contigs for read and transcript coverage. This involved mapping of reads against the merged assembly followed by computing the read coverage for each position.

1. Build genome index and map Illumina reads to the assembly with Bowtie2 using options to consider relevant reads only, and sort bam file with sambamba v.0.6.7 (TARASOV *et al.* 2015) :

```
$ bowtie2-build -f merged_genome_assembly_dedup.fasta merged_genome_assembly_dedup
$ bowtie2 --local --no-unal -p 40 --no-mixed --no-discordant -x merged_genome_assembly_dedup.fasta -1
MiSeqReads_1.fastq.gz -2 MiSeqReads_2.fastq.gz -U MiSeqReads_S.fastq.gz 2> MiSeqReads.mapped.log |
sambamba view -S -f bam /dev/stdin | sambamba sort /dev/stdin -o MiSeqReads.mapped.sorted.bam ; sambamba
index MiSeqReads.mapped.sorted.bam
```

2. Calculate the coverage by position with bedtools genomecov (BEDTools v.2-2.25.0, (QUINLAN AND HALL 2010)):

```
$ bedtools genomecov -bga -ibam MiSeqReads.mapped.sorted.bam > MiSeqReads.mapped.sorted.genomecov.bedgraph
```

3. Extract genome positions where the read coverage is below 3:

```
$ awk '{if($4<3) print $0}' MiSeqReads.mapped.sorted.genomecov.bedgraph >
MiSeqReads.mapped.sorted.genomecov.lowcov.bedgraph
```

A similar procedure was performed with long reads, using the long-read mapper minimap2 (LI 2018) with recommended options for this type of reads.

1. Build genome index and map PacBio reads to assembly with minimap2 and sort output:

```
$ minimap2 -t 40 -d merged_genome_assembly_dedup.mmi merged_genome_assembly_dedup.fasta
$ minimap2 -ax map-pb -t 40 merged_genome_assembly_dedup.mmi PacBioReads.fasta 2> PacBioReads.mapped.log |
sambamba view -S -f bam /dev/stdin | sambamba sort /dev/stdin -o PacBioReads.mapped.sorted.bam ; sambamba
index PacBioReads.mapped.sorted.bam
```

2. Keep the reads with a mapping quality >0 and a length >1, and remove unmapped reads:

```
$ sambamba view -f bam -F "not unmapped and sequence_length > 1 mapping_quality > 0" -o
PacBioReads.mapped.sorted.filtered.bam PacBioReads.mapped.sorted.bam
```

3. Extract genome positions where the read coverage is below 2:

```
$ bedtools genomecov -bga -ibam PacBioReads.mapped.sorted.filtered.bam >
PacBioReads.mapped.sorted.filtered.genomecov.bedgraph
$ awk '{if($4<2) print $0}' PacBioReads.mapped.sorted.filtered.genomecov.bedgraph >
PacBioReads.mapped.sorted.filtered.genomecov.lowcov.bedgraph
```

A similar procedure was performed with transcripts.

1. Build genome index and map transcript sequences to assembly with gmap and sort output:

```
$ gmap_build -D . -d merged_genome_assembly_dedup  merged_genome_assembly_dedup.fasta > gmapbuild.out 2>&1
$ gmap --format gff3_gene --nofails -t --min-identity=0.90 --min-intronlength=20 --gff3-add-separators=0 -
D . -d merged_genome_assembly_dedup/ transcripts.fasta > transcripts.mapped.gff3 2> transcripts.mapped.log
$ awk 'NR>3' transcripts.mapped.gff3 | sort -k 1,1 -k 4,4n > transcripts.mapped.sorted.gff3
```

Next, we examined which low-coverage positions were spanned by transcripts or long reads, and would therefore not be considered as mis-junctions anymore.

1. Intersect short-read low-coverage positions with intervals spanned by at least one transcript and report *absence* of overlap:

```
$ bedtools intersect -v -a MiSeqReads.mapped.sorted.genomecov.lowcov.bedgraph -b
transcripts.mapped.sorted.gff3 > MiSeq_lowcov-Transcripts.intersect.bedgraph
```

2. Intersect non-overlapping positions from step 1 with intervals of low (<2) long-read coverage and report overlap (i.e., report that none of the datasets provides evidence for contiguity of the contig):

```
$ bedtools intersect -a MiSeq_lowcov-Transcripts.intersect.bedgraph -b
PacBioReads.mapped.sorted.filtered.genomecov.lowcov.bedgraph > MiSeq_lowcov-Transcripts-
PacBio_lowcov.intersect.bedgraph
```

3. Sort positions and merge them to create intervals:

```
$ sort -k1,1 -k2,2n MiSeq_lowcov-Transcripts-PacBio_lowcov.intersect.bedgraph > MiSeq_lowcov-Transcripts-
PacBio_lowcov.intersect.sorted.bedgraph
$ bedtools merge -i MiSeq_lowcov-Transcripts-PacBio_lowcov.intersect.sorted.bedgraph > MiSeq_lowcov-
Transcripts-PacBio_lowcov.intersect.sorted.merged.bed
```

Finally, we cut the merged genome at the mis-junction sites and removed the resulting contig 'pieces' that are shorter than 100 bp.

1. Cut contigs with the in-house script 'cut_chimera_fasta.py':

```
$ cut_chimera_fasta.py -b MiSeq_lowcov-Transcripts-PacBio_lowcov.intersect.sorted.merged.bed -f
merged_genome_assembly_dedup.fasta -o merged_genome_assembly_dedup_cut.fasta
```

2. Remove all contigs shorter than 100 bp with the in-house script 'remove_small.pl':

```
$ remove_small.pl 100 merged_genome_assembly_dedup_cut.fasta  >
merged_genome_assembly_dedup_cut_longer100.fasta
```

Transcriptome assembly

RNA-seq reads (Illumina HiSEQ) from the two stranded poly-RNA libraries PA and PA2 were collectively assembled *de novo* with Trinity v.2.6.6 (https://github.com/trinityrnaseq/trinityrnaseq; (GRABHERR *et al.* 2011)) using default parameters. We also generated a genome-guided transcriptome assembly, again with default parameters. A set of high-confidence transcripts was generated to test whether genomic contig regions with low-read coverage were due to mis-joining or were spurious (see Section 1.5. Correction of mis-joining). These were selected based on two criteria: (i) presence of a Spliced-Leader (SL) sequence at the 5′ end, and (ii) an overall read coverage of >100. To quantify gene expression, the corresponding transcript sequence was first inferred for each protein ID from the 'submission' proteome (see below). Next, the poly-A RNA-Seq reads mentioned above were mapped on individual transcripts using bowtie2 v2.4.5 (LANGMEAD AND SALZBERG 2012) in the end-to-end mode, assuming the maximum insert length of 600 bp and only allowing alignment concordant with strand-specific expression. The SAM file was converted to a BAM file with sambamba v0.7.1 (TARASOV *et al.* 2015). Transcript levels were then calculated using salmon v1.9.0 (PATRO *et al.* 2017) in the alignment mode (default parameters, library type 'ISF').

Structural genome annotation

The structural genome annotation pipeline employed here was implemented similarly as described earlier (GRAY *et al.* 2020).

Structural annotation of protein-coding genes. Compared to the earlier pipeline, we used a modified RNA-Seq read-mapping step here. Briefly, the genome assembly was first masked for simple repeats using RepeatScout v1.0.5 (PRICE *et al.* 2005) and RepeatMasker v4.0.9 [unpublished: https://www.repeatmasker.org/].

```
$ build_lmer_table -sequence Dp_PB-MI_190104_dedup_cut_l100.fasta -freq lmer_table.txt
$ RepeatScout -minthres 150 -sequence Dp_PB-MI_190104_dedup_cut_l100.fasta -output repscout.fasta -freq
lmer_table.txt
RepeatMasker -xsmall -gff -s -pa 40 -lib repscout.fasta Dp_PB-MI_190104_dedup_cut_l100.fasta
```

RNA-Seq reads were then mapped to the genome assembly using STAR v2.6.1b (DOBIN *et al.* 2013) to retrieve the positions of SL sequences, inferred within soft-clipped regions, using an in-house script.

```
$ STAR --runThreadN 40 --runMode genomeGenerate --genomeDir STAR-index --genomeFastaFiles Dp_PB-
MI_190104_dedup_cut_l100.fasta --genomeSAindexNbases 13
$ STAR --runThreadN 40 --genomeDir build-index --alignEndsType Local --readFilesIn PA+DPA2_1.fastq.gz
PA+DPA2_2.fastq.gz --outSAMtype BAM SortedByCoordinate --outSJfilterIntronMaxVsReadN 100 300 500 --
alignIntronMin 19 --alignIntronMax 20000 --outFileNamePrefix STAR_ --outSAMattributes All --
outSAMattrIHstart 0 --outSAMstrandField intronMotif --limitBAMsortRAM 27643756136 --readFilesCommand zcat
```

Reads were then depleted of the SL sequence (5′–AACCAACGATTTAAAAGCTACAGTTTCTGTACTTTATTG), using trimmomatic v0.30 (BOLGER *et al.* 2014):

```
$ java -classpath trimmomatic-0.30.jar org.usadellab.trimmomatic.TrimmomaticPE -threads 24 -phred33
PA+DPA2_1.fastq.gz PA+DPA2_2.fastq.gz PA+DPA2_1.filtered.fastq PA+DPA2_2_unpaired.fastq
PA+DPA2_2.filtered.fastq PA+DPA2_2_unpaired.fastq ILLUMINACLIP:splicedleader.fasta:3:30:9:10 MINLEN:50
```

and then remapped to the genome assembly using STAR

```
$ STAR --runThreadN 40 --genomeDir build-index --alignEndsType Local --readFilesIn PA+DPA2_1.fastq.gz
PA+DPA2_2.fastq.gz --outSAMtype BAM SortedByCoordinate --outSJfilterIntronMaxVsReadN 100 300 500 --
alignIntronMin 19 --alignIntronMax 20000 --outFileNamePrefix STAR_ --outSAMattributes All --
outSAMattrIHstart 0 --outSAMstrandField intronMotif --limitBAMsortRAM 27643756136 --readFilesCommand zcat
```

The SL-trimmed reads, both unmapped and mapped, were assembled *de novo* and guided by the genome assembly, respectively, using Trinity v2.6.6 (GRABHERR *et al.* 2011).

```
$ Trinity --seqType fq --max_memory 150G --left PA+DPA2_2.filtered.fastq --right PA+DPA2_1.filtered.fastq
--CPU 40 --output trinity-denovo --full_cleanup --SS_lib_type RF
$ Trinity --genome_guided_max_intron 20000 --max_memory 250G --CPU 40 --genome_guided_bam
STAR_Aligned.sortedByCoord.out.bam --output trinity-gg --full_cleanup --SS_lib_type RF
```

The resulting transcriptome assemblies were combined into a single file, trinity-comprehensive.fasta, and aligned to the genome assembly using PASA v2.3.3 (HAAS *et al.* 2003).

```
$ Launch_PASA_pipeline.pl -c alignAssembly.config -C -r -R -g Dp_PB-MI_190104_dedup_cut_l100.fasta -t
trinity-comprehensive.fasta.clean -T -u trinity-comprehensive.fasta --ALIGNERS gmap,blat --CPU 40 --TDN
tdn.accs -I 20000 --stringent_alignment_overlap 30.0 –transcribed_is_aligned_orient
```

The alignments were then combined into a single, comprehensive assembly:

```
build_comprehensive_transcriptome.dbi -c alignAssembly.config -t Dp_PB-
MI_190104_dedup_cut_l100.sqlite.assemblies.fasta --min_per_ID 95 --min_per_aligned 95
```

Protein sequence accessions GCA_000002725.2, GCA_000002845.2, GCA_000002875.2, GCA_000209065.1, GCA_000227375.1, GCA_000691245.1, GCA_001457755.2, GCA_001680005.1, GCA_002087225.1, GCA_002157705.1, GCA_003719475.1, GCA_003719485.1, GCA_900002335.1, GCA_900005765.1, GCA_900005855.1, GCA_900090025.2, GCA_900090045.1, GCA_900097015.1, GCA_900240055.1, along with other available sequences from *Discoba,* were aligned to the genome using Spaln v2.2.2 (GOTOH 2008).

```
$ spaln -C1 -O12 -Q5 -yL20 -yX -t40 -dDp_PB-MI_190104_dedup_cut_l100 all_protein_data.faa
```

The *ab initio* predictors employed were Genemark v4.33 with intron intervals as hints derived from RNA-Seq read mapping (LOMSADZE *et al.* 2014),

```
$ gmes_petap.pl --soft 1000 --ET=introns.gff --et_score=3 --cores=40 --sequence=Dp_PB-
MI_190104_dedup_cut_l100.fasta
```

CodingQuarry v2.0 was run with transcript alignments as hints (TESTA *et al.* 2015):

```
$ CodingQuarry -p 40 -f Dp_PB-MI_190104_dedup_cut_l100.fasta -t pasa_transcripts.gff3
```

Augustus v3.3.2 (STANKE *et al.* 2006) was employed with spliced-leader-sequence genomic positions as transcription-start-site (tss) hints, along with protein sequence alignments, RNA-Seq read coverage, and transcript alignments (as described at https://bioinf.uni-greifswald.de/bioinf/wiki/pmwiki.php?n=Augustus.Augustus), and Snap (KORF 2004) trained on Augustus models with a score of 1 (as per the instructions on https://github.com/KorfLab/SNAP). Finally, the PASA assembly, Spaln alignments, as well as Augustus, Snap, and Codingquarry gene models, were combined into a single consensus with Evidencemodeler v1.1.1 (HAAS *et al.* 2008) following the instructions at https://evidencemodeler.github.io/.

The comprehensive, non-redundant transcript and protein sequence collection was generated by first combining the manually curated gene models with the remaining automated models. Models manually identified as split were conceptually translated and reassembled. Those combined sequences were clustered with the assembled transcripts containing ORFs using CD-HIT (default parameters of v4.8.1) (FU *et al.* 2012). Transcript sequences with no corresponding matches were combined with the curated and automated model sequences and the nucleotide sequences were then conceptually translated. This comprehensive sequence collection was used for downstream analyses.

Structural annotation of non-coding RNA genes. Modeling of tRNAs was performed using tRNAscan-SE v1.3.1 (LOWE AND EDDY 1997):

```
$ tRNAscan-SE --brief --codons --output tRNAscan-SE.out Dp_PB-MI_190104_dedup_cut_l100.fasta
```

Infernal v1.1.1 (NAWROCKI AND EDDY 2013) was used to identify other classes of non-coding RNAs using the covariance models from Rfam v12.1 (GRIFFITHS-JONES *et al.* 2003):

```
$ cmscan --rfam --fmt 2 -E 1e-3 --nohmmonly --oskip --clanin Rfam.clanin --cpu 40 --tblout rfam.out –noali
Rfam.cm Dp_PB-MI_190104_dedup_cut_l100.fasta
```

Functional annotation of protein-coding gene models

Coding sequences from gene models were extracted from the output of Evidencemodeler and conceptually translated. The translated sequences were then searched against UniProt/SWISS-PROTKB (downloaded March 23, 2018; (BOUTET *et al.* 2007)), as well as the GenBank accessions and other *Discoba* sequences used for structural annotation, to identify the single best hit below the maximum threshold E-value of 1.0e-7 using Blastp v2.2.31+.

```
$ makeblastdb -dbtype prot -in all_protein_data.faa
$ blastp -db all_protein_data.faa -num_threads 40 -outfmt '6 qseqid sseqid stitle pident length qlen slen
evalue bitscore' -max_target_seqs 5 -evalue 1e-7 -query Dp_PB-MI_190104_dedup_cut_l100.faa -out Dp_PB-
MI_190104_dedup_cut_l100.faa.blastp_all_protein_data
```

Product names were transferred to *D. papillatum* gene models by taking the single best hit against the SWISS-PROT database, i.e., lowest E-value below a global cutoff of 1e-7. Precedence was given to hits of GenBank accessions if the E-value was lower than a competing hit to the SWISS-PROT database, otherwise the product name was automatically transferred in the absence of a SWISS-PROT hit provided the E-value was below the global cutoff. All remaining models without hits below the threshold were assigned 'hypothetical protein' as their 'product'. Hmmer v3.1b1 was also used to search for conserved domains described in Pfam v31.0 using the model-specific noise threshold as E-value cutoff. Blastp and Hmmer search hits were included in the 9th column of the gff3 file (https://www.ncbi.nlm.nih.gov/genbank/genomes_gff/) as 'product' and 'inference' attributes, respectively, as per the NCBI eukaryotic genome annotation guidelines (https://www.ncbi.nlm.nih.gov/genbank/eukaryotic_genome_submission_annotation/).

Genome viewing and annotation curation

For viewing contigs, read coverage, gene models and functional assignments, and editing structural and functional annotations, we used the web-based Apollo software v.2.6.2 (https://github.com/GMOD/Apollo/releases/latest; (DUNN *et al.* 2019). Apollo allows real-time, collaborative and simultaneous genome annotation editing, whereby models can later be exported in gff3 format. Manually curated models were exported from Apollo in gff3 format and incorporated into the automated annotation.

AUTHOR CONTRIBUTIONS

Conceptualization – S.M., C.P., M.S., G.B.; **Data curation –** S.M., M.S.; **Formal analysis, Investigation, Writing, original draft –** S.M., C.P., M.S., P.S., G.B., M.V.; **Visualization –** P.S., G.B.; **Writing, review & editing –** all co-authors.

REFERENCES

Bolger, A. M., M. Lohse and B. Usadel, 2014 Trimmomatic: a flexible trimmer for Illumina sequence data. Bioinformatics 30**:** 2114-2120.

Boutet, E., D. Lieberherr, M. Tognolli, M. Schneider and A. Bairoch, 2007 UniProtKB/Swiss-Prot. Methods Mol Biol 406**:** 89-112.

Clayton, C., 2019 Regulation of gene expression in trypanosomatids: living with polycistronic transcription. Open Biol 9**:** 190072.

Denisov, G., B. Walenz, A. L. Halpern, J. Miller, N. Axelrod *et al.*, 2008 Consensus generation and variant detection by Celera Assembler. Bioinformatics 24**:** 1035-1040.

Dobin, A., C. A. Davis, F. Schlesinger, J. Drenkow, C. Zaleski *et al.*, 2013 STAR: ultrafast universal RNA-seq aligner. Bioinformatics 29**:** 15-21.

Dunn, N. A., D. R. Unni, C. Diesh, M. Munoz-Torres, N. L. Harris *et al.*, 2019 Apollo: democratizing genome annotation. PLoS Comput Biol 15**:** e1006790.

Fu, L., B. Niu, Z. Zhu, S. Wu and W. Li, 2012 CD-HIT: accelerated for clustering the next-generation sequencing data. Bioinformatics 28**:** 3150-3152.

Gotoh, O., 2008 A space-efficient and accurate method for mapping and aligning cDNA sequences onto genomic sequence. Nucleic Acids Res 36**:** 2630-2638.

Grabherr, M. G., B. J. Haas, M. Yassour, J. Z. Levin, D. A. Thompson *et al.*, 2011 Full-length transcriptome assembly from RNA-Seq data without a reference genome. Nat Biotechnol 29**:** 644-652.

Gray, M. W., G. Burger, R. Derelle, V. Klimeš, M. M. Léger *et al.*, 2020 The draft nuclear genome sequence and predicted mitochondrial proteome of *Andalucia godoyi*, a protist with the most gene-rich and bacteria-like mitochondrial genome. BMC Biol 18**:** 22.

Griffiths-Jones, S., A. Bateman, M. Marshall, A. Khanna and S. R. Eddy, 2003 Rfam: an RNA family database. Nucleic Acids Res 31**:** 439-441.

Haas, B. J., A. L. Delcher, S. M. Mount, J. R. Wortman, R. K. Smith, Jr. *et al.*, 2003 Improving the *Arabidopsis* genome annotation using maximal transcript alignment assemblies. Nucleic Acids Res 31**:** 5654-5666.

Haas, B. J., S. L. Salzberg, W. Zhu, M. Pertea, J. E. Allen *et al.*, 2008 Automated eukaryotic gene structure annotation using EVidenceModeler and the Program to Assemble Spliced Alignments. Genome Biol 9**:** R7.

Haghshenas, E., F. Hach, S. C. Sahinalp and C. Chauve, 2016 CoLoRMap: Correcting Long Reads by Mapping short reads. Bioinformatics 32: i545-i551.
Koren, S., B. P. Walenz, K. Berlin, J. R. Miller, N. H. Bergman *et al.*, 2017 Canu: scalable and accurate long-read assembly via adaptive k-mer weighting and repeat separation. Genome Res 27: 722-736.
Korf, I., 2004 Gene finding in novel genomes. BMC Bioinformatics 5: 59.
Kriventseva, E. V., D. Kuznetsov, F. Tegenfeldt, M. Manni, R. Dias *et al.*, 2019 OrthoDB v10: sampling the diversity of animal, plant, fungal, protist, bacterial and viral genomes for evolutionary and functional annotations of orthologs. Nucleic Acids Res 47: D807-d811.
Langmead, B., and S. L. Salzberg, 2012 Fast gapped-read alignment with Bowtie 2. Nat Methods 9: 357-359.
Li, H., 2018 Minimap2: pairwise alignment for nucleotide sequences. Bioinformatics 34: 3094-3100.
Lomsadze, A., P. D. Burns and M. Borodovsky, 2014 Integration of mapped RNA-Seq reads into automatic training of eukaryotic gene finding algorithm. Nucleic Acids Res 42: e119.
Lowe, T. M., and S. R. Eddy, 1997 tRNAscan-SE: a program for improved detection of transfer RNA genes in genomic sequence. Nucleic Acids Res 25: 955-964.
Myers, E. W., G. G. Sutton, A. L. Delcher, I. M. Dew, D. P. Fasulo *et al.*, 2000 A whole-genome assembly of *Drosophila*. Science 287: 2196-2204.
Nawrocki, E. P., and S. R. Eddy, 2013 Infernal 1.1: 100-fold faster RNA homology searches. Bioinformatics 29: 2933-2935.
Patro, R., G. Duggal, M. I. Love, R. A. Irizarry and C. Kingsford, 2017 Salmon provides fast and bias-aware quantification of transcript expression. Nat Methods 14: 417-419.
Price, A. L., N. C. Jones and P. A. Pevzner, 2005 De novo identification of repeat families in large genomes. Bioinformatics 21 Suppl 1: i351-358.
Quast, C., E. Pruesse, P. Yilmaz, J. Gerken, T. Schweer *et al.*, 2013 The SILVA ribosomal RNA gene database project: improved data processing and web-based tools. Nucleic Acids Res 41: D590-596.
Quinlan, A. R., and I. M. Hall, 2010 BEDTools: a flexible suite of utilities for comparing genomic features. Bioinformatics 26: 841-842.
Schmieder, R., and R. Edwards, 2011 Quality control and preprocessing of metagenomic datasets. Bioinformatics 27: 863-864.
Seppey, M., M. Manni and E. M. Zdobnov, 2019 BUSCO: assessing genome assembly and annotation completeness. Methods Mol Biol 1962: 227-245.
Stanke, M., O. Schöffmann, B. Morgenstern and S. Waack, 2006 Gene prediction in eukaryotes with a generalized hidden Markov model that uses hints from external sources. BMC Bioinformatics 7: 62.
Sturm, N. R., D. A. Maslov, E. C. Grisard and D. A. Campbell, 2001 *Diplonema* spp. possess spliced leader RNA genes similar to the Kinetoplastida. J Eukaryot Microbiol 48: 325-331.
Tarasov, A., A. J. Vilella, E. Cuppen, I. J. Nijman and P. Prins, 2015 Sambamba: fast processing of NGS alignment formats. Bioinformatics 31: 2032-2034.
Testa, A. C., J. K. Hane, S. R. Ellwood and R. P. Oliver, 2015 CodingQuarry: highly accurate hidden Markov model gene prediction in fungal genomes using RNA-seq transcripts. BMC Genomics 16: 170.
Wu, T. D., and C. K. Watanabe, 2005 GMAP: a genomic mapping and alignment program for mRNA and EST sequences. Bioinformatics 21: 1859-1875.

3. The ploidy level of *Diplonema papillatum*

INTRODUCTION

Most eukaryotes alternate between a diploid and a haploid phase. Such an alternation is a consequence of sexual reproduction, because gamete fusion leads to a diploid phase and meiosis to a haploid phase (MABLE AND OTTO 1998). However, the duration of these phases varies considerably across eukaryotes. In most metazoans and land plants, the diploid stage of the life cycle is the predominant state, i.e., mitotic cell divisions occur exclusively in the diploid phase. Other types of alternation are observed in fungi and diverse protist groups. In the haplontic cycle, mitotic cell division occurs entirely in the haploid form (e.g., in the fission yeast *Schizosaccharomyces*), whereas in the haploid-diploid cycle, mitosis takes place in both phases, often forming morphologically different organisms (e.g., in jellyfish).

Diplonema papillatum has never been observed to reproduce sexually. Here we attempt to infer the ploidy status of the strain propagated in the laboratory over many years, *via* the heterozygosity of its nuclear genome.

RESULTS AND DISCUSSION

Extremely low heterozygosity of the *Diplonema* nuclear genome

Heterozygosity of a genome is typically determined by analyzing either single-nucleotide polymorphisms (SNPs) in reads aligned to the reference genome —which requires an assembly— or k-mers occurring in sequencing reads —which does not require a genome assembly.

The approach using **SNPs** (also called single-nucleotide variants (SNVs)) relies on accurate read mapping to the reference genome. However, ~50% of the 280.4 Mbp *Diplonema* nuclear genome assembly consists of repeats up to >20 kbp long, some of which occur in nearly 5,000 copies. These repeats cause erroneous mapping of Illumina reads to the assembly, and thus generate spurious polymorphic sites. Therefore, for this analysis we only used read pairs that congruently and uniquely align with the *Diplonema* genome assembly and we excluded all variants that fall in repeat regions. After removing sites with low mapping quality and other biases (strand bias, etc.), only 557 SNPs remained in the 142 Mbp-long genome portion outside repeats. As commonly observed, nucleotide transitions (327) are more frequent than transversions (230) in this final set of variants (**Supplementary Table S10**). The resulting heterozygous SNP rate of the *Diplonema* nuclear genome is extremely low. With only 4 e-06 per 1 kbp, it is merely 0.2% of that of a diploid eukaryote, e.g., human (SACHIDANANDAM *et al.* 2001). In addition, the profile of allele frequencies within the *Diplonema* genome (**Supplementary Figure S8**) shows no peaks, in contrast to profiles from diploid and polyploid organisms.

The second approach to evaluating genome heterozygosity counts **k-mers** directly in reads. In theory, k-mer profiles of homozygous (haploid or higher ploidies) genomes are Poisson distributions centred at the average read coverage, but several biases cause distortions. Repeats in the genome over-amplify certain k-mers, thus adding a drifting-off shoulder at higher coverage, whereas sequencing errors generate numerous low-frequency k-mers, adding a high peak toward zero coverage. For analysing the k-mer distribution, we used nuclear Illumina reads (57,742,700 reads totalling 13,139,885,848 bp; mitochondrial reads were removed; see Methods). The resulting k-mer profile (**Supplementary Figure S9**) resembles a Poisson distribution typical for homozygotic genomes. The only slight deviation from a true Poisson distribution is most likely due to sequencing errors and repeats.

K-mer frequencies of reads also allow one to estimate the genome size, notably based on the total number of k-mers and the peak position of the distribution. For the k-mer length range of 17, 19, 21, and 23, the average inferred genome length is 259,725,615 bp.

Supplementary Table S10. Single nucleotide variants[a].

Reference allele	Alternative Allele				Sum of reference alleles	Cumulative substitutions
	A	C	G	T		
A	/	41	67	24	132	**A↔G: 172**
C	32	/	25	94	151	A↔C: 73
G	105	21	/	25	151	A↔T: 57
T	33	84	36	/	153	C↔G: 46
Sum of alternative alleles	170	146	128	143		**C↔T: 178**
						T↔G: 66
Total transitions						350 (59%)
Total transversions						242 (41%)

[a]Significant variants after filtering; see Methods

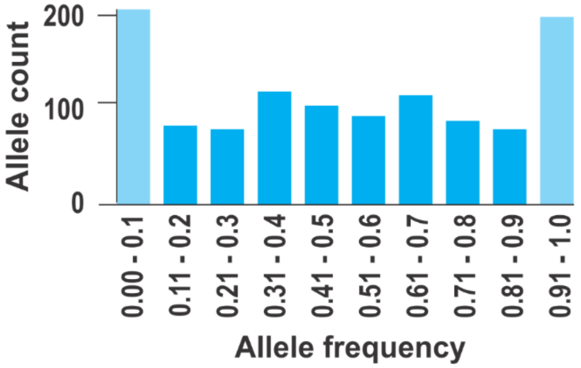

Supplementary Figure S8. Allele frequency outside repeat regions of the *D. papillatum* nuclear genome. After filtering sites for mapping quality, strand and location biases, a total of 557 SNPs are retained in the 142,402,538 Mbp nuclear genome regions outside repeats. Bars represent bins for allele frequencies. The left-most and right-most bins most likely include sequencing and base-calling errors. The absence of pronounced peaks in the frequency range of 0.2 to 0.8 and the low heterozygosity level corroborate that the genome is haploid.

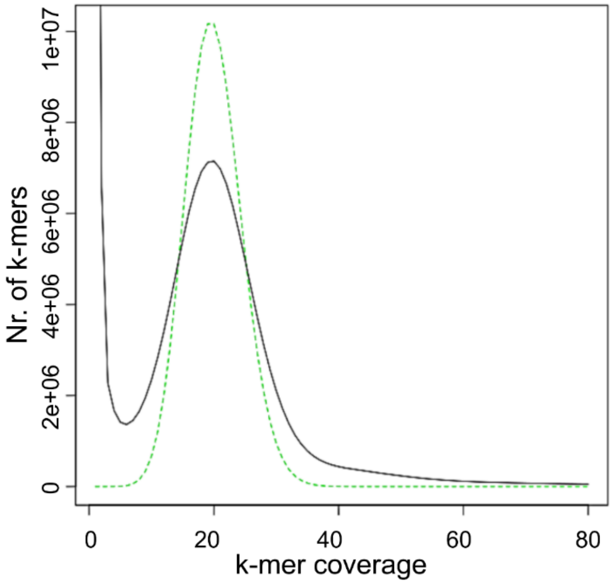

Supplementary Figure S9. K-mer profile of reads from the *D. papillatum* nuclear genome. X-axis, k-mer coverage, i.e., number of 'individuals' per k-mer species; Y-axis, number of distinct k-mer species. Black line, the 17-mer profile of *Diplonema* nuclear MI-Seq reads. The shape differs only marginally from those for 19-mers, 21-mers and 23-mers. Green dotted line, Poisson distribution centered at the mean coverage. The reads' k-mer distribution lacks a shoulder left to the main peak, otherwise indicative of heterozygosity. The high number of unique k-mers and k-mers with a very low coverage is most probably due to reads with sequencing errors.

Diplonema is most likely haploid

The lack of heterozygosity is inherent in haploid genomes but also in diploid lines originating from recent spontaneous chromosome duplication (autodiploidization leading to whole genome duplication). Lack of heterozygosity can also occur when haploid cells in a clonal population mate and when homolog chromosomes fail to separate during meiosis, thus giving rise to diploid gametes.

With computational methods, it is impossible to distinguish homozygous haploid from homozygous 2N or >2N genomes. However, gene replacement experiments are evidence for a haploid *Diplonema* nuclear genome (KAUR *et al.* 2018). In diploid organisms, gene replacement leads nearly always to two gene versions, the original one on one chromosome, and the replaced one on the other, which can be tested by PCR amplification and Southern hybridization of the corresponding genomic region. In multiple knock-in transformation experiments in *Diplonema* with an engineered gene version, only a single allele was ever detected, strongly suggesting haploidy.

It should be noted that the statement of haploidy refers to the *D. papillatum* culture used in the laboratory. Here, the exclusive form of reproduction appears to be mitosis, which is probably the predominant reproduction mode in *Diplonema's* natural habitat. While as of now, **sexual reproduction** or a diploid stage have not been observed in this protist, the gene repertoire (see main text) implies that this organism has the potential to form diploid zygotes that undergo meiosis.

METHODS

Mapping of reads onto the nuclear genome sequence. We used all genomic MiSeq reads of *Diplonema* generated by us and removed mitochondrial reads by eliminating those that aligned with sequences of complete mitochondrial chromosomes (GenBank acc. nos. EU123536.1; EU12353637.1; HQ288823.1, HQ288824.1; JQ302962.1, JQ314396.1) using Bowtie2 version 2.3.4.3 (command line arguments --local --un) (LANGMEAD AND SALZBERG 2012). The ~58 million 'cleaned' reads (~13 Gbp) were mapped to the reference assembly using Bowtie2 (--no-discordant --no-mixed --no-unal) yielding 98.7% alignment rate. The resulting sam output file was converted with Samtools v1.8 subcommand view (LI *et al.* 2009) to the bam format, and further processed with the Picard tools v.1.139, using subcommands Sortsam, MarkDuplicates, AddGroup, and IndexBam (https://broadinstitute.github.io/picard/), all in default mode.

Determination of single nucleotide variants. Sequence variants across reads per alignment column were called with Freebayes v1.2.0-2-g29c4002 (--ploidy 4 to allow for higher ploidy) (GARRISON E AND MARTH G 2012). To remove from the resulting vcf file variants that fall into genomic regions containing repeats, we used the corresponding gff file generated by our genome annotation pipeline (Sarrasin et al., unpublished). This pipeline calls Repeatmasker v.4.0.6 (http://repeatmasker.org) developed by A.F.A. Smit, R. Hubley & P. Green, and uses RepBase24.06, release 06-20-2019 (JURKA *et al.* 2005). The relative complement between repeat intervals and variant positions in the vcf file were obtained by executing BEDTools v2-2.28 subcommand intersect (-a -v) (QUINLAN 2014). Then, indels and low quality variants were removed with Vcftools v0.1.12b (--minQ 30 –remove --indels --recode --recode-INFO-all) (http://vcftools.sourceforge.net/docs.html; (DANECEK *et al.* 2011)), and variant sites were further filtered by read placement score (RPP >20) and strand bias (SRP <20) using an in-house script. The number of single-nucleotide variants (with frequencies between >0 and <1) recovered at each step are listed in **Supplementary Table S11**. The vcf file was converted to an allele-frequency table with the Genome Analysis ToolKit (GATK; (MCKENNA *et al.* 2010)) subcommand VariantToTable, by extracting two fields, the reference allele observation count (RO) and the alternate allele observation count (AO). The table was then imported into Excel to generate the chart.

Supplementary Table S11. Number of single-nucleotide variants at the diverse filtering steps.

Type of variants	Count
Initial variant count across the entire genome and including indels and substitutions	2,469,945
Nr. of variants outside repeats	871,013
Nr. of variants after indel removal and quality filtering (minQ 30)	16,640
Nr. of variants after read-placement and strand-bias filtering (RPP>20; SAP, SRP<20)	587

Determination of k-mer distribution and estimation of genome size. We 'decontaminated' Illumina reads from those containing mitochondrial sequences by mapping reads against the nuclear genome assembly. A total of 58 million 'decontaminated' reads remained summing up to 13 Gbp. The k-mer distribution of these genomic reads was calculated with the k-mer counter Jellyfish (MARÇAIS AND KINGSFORD 2011) using the command jellyfish count with the option -C for

counting k-mers from both strands and -m 17 to -m 23 for k-mer sizes from 17 to 23. The histogram of k-mer occurrences were generated with jellyfish histo and default parameters following the tutorial described at http://koke.asrc.kanazawa-u.ac.jp/HOWTO/kmer-genomesize.html. Histograms were plotted with R, including the comparison with a Poisson distribution and the estimation of the genome size inferred from the total number of k-mers (area under the curve) divided by the mean k-mer coverage. The size estimates based on the k-mer counts of lengths 17, 19, 21, and 23 are listed in **Supplementary Table S12**.

Supplementary Table S12. Genome-size estimation based on K-mer distribution.

K-mer length	17	19	21	23	**Average**
Size estimate	250,341,083 bp	258,503,293 bp	267,598,590 bp	262,459,497 bp	**259,725,615 bp**

Determination of allele frequencies. From the vcf file containing only single-nucleotide variants of high quality, we extracted the allele frequencies (AD) and coverage (DP) with the GATK subcommand VariantsToTable (-GF AD –GF DP). Then the numbers were imported into Excel to calculate the quotient AD/DP for each allele and generate a frequency graph.

AUTHOR CONTRIBUTIONS

Conceptualization, Data curation, Formal analysis, Investigation, Visualization, Writing, original draft – G.B., L.L.; **Writing, review & editing –** all co-authors.

REFERENCES

Danecek, P., A. Auton, G. Abecasis, C. A. Albers, E. Banks *et al.*, 2011 The variant call format and VCFtools. Bioinformatics 27**:** 2156-2158.

Garrison E, and Marth G, 2012 Haplotype-based variant detection from short-read sequencing, pp., edited by arXiv.

Jurka, J., V. V. Kapitonov, A. Pavlicek, P. Klonowski, O. Kohany *et al.*, 2005 Repbase Update, a database of eukaryotic repetitive elements. Cytogenet Genome Res 110**:** 462-467.

Kaur, B., M. Valach, P. Peña-Diaz, S. Moreira, P. J. Keeling *et al.*, 2018 Transformation of *Diplonema papillatum*, the type species of the highly diverse and abundant marine microeukaryotes Diplonemida (Euglenozoa). Environ Microbiol 20**:** 1030-1040.

Langmead, B., and S. L. Salzberg, 2012 Fast gapped-read alignment with Bowtie 2. Nat Methods 9**:** 357-359.

Li, H., B. Handsaker, A. Wysoker, T. Fennell, J. Ruan *et al.*, 2009 The Sequence Alignment/Map format and SAMtools. Bioinformatics 25**:** 2078-2079.

Mable, B. K., and S. P. Otto, 1998 The evolution of life cycles with haploid and diploid phases. BioEssays 20**:** 453-462.

Marçais, G., and C. Kingsford, 2011 A fast, lock-free approach for efficient parallel counting of occurrences of k-mers. Bioinformatics 27**:** 764-770.

McKenna, A., M. Hanna, E. Banks, A. Sivachenko, K. Cibulskis *et al.*, 2010 The Genome Analysis Toolkit: a MapReduce framework for analyzing next-generation DNA sequencing data. Genome Res 20**:** 1297-1303.

Quinlan, A. R., 2014 BEDTools: the swiss-army tool for genome feature analysis. Curr Protoc Bioinformatics 47**:** 11.12.11-34.

Sachidanandam, R., D. Weissman, S. C. Schmidt, J. M. Kakol, L. D. Stein *et al.*, 2001 A map of human genome sequence variation containing 1.42 million single nucleotide polymorphisms. Nature 409**:** 928-933.

4. Intron splicing and structural RNAs

INTRODUCTION

Nuclear protein-coding genes of eukaryotes often contain intervening sequences, which are removed from pre-mRNAs by RNA splicing. The large majority of these **spliceosomal introns** have a GT dinucleotide at their 5′ end and an AG at their 3′ end, and are referred to as the 'major intron type'. Introns with AT-AC splice-site combinations (the 'minor intron type') occur occasionally in embryophyte plants and most metazoan groups, but have also been reported in oomycetes, mycetozoans, and a few basally-branching fungal lineages. Among all introns in plant and animal nuclear genomes, less than 0.5% have AT-AC boundaries, while those of baker's and fission yeast are exclusively of the major type (TURUNEN *et al.* 2013).

GT-AG and AT-AC introns are processed by the major and minor **spliceosome**, respectively, that are composed of five small nuclear RNAs (U RNAs) and several proteins (PATEL AND STEITZ 2003; TURUNEN *et al.* 2013). The most clear-cut diagnostic feature for distinguishing the two spliceosome types is their RNA-subunit composition, with U1, U2, U4, U5, and U6 RNAs defining the major spliceosome, whereas U11, U12, U4atac, U5, and U6atac RNAs are hallmarks of the minor spliceosome. Thus, the two spliceosome types share U5, whereas U1 and U11, U2 and U12, U4 and U4 atac, and U6 and U6atac are functionally analogous. They share most features of their two and three-dimensional structure but differ significantly in sequence (TARN AND STEITZ 1996b; TARN AND STEITZ 1996a). Yet, in some eukaryotes, intron splice sites deviate from the conserved GT-AG or AT-AC motifs (e.g., AT-AG), and in others, the number of 'minor introns' has increased significantly in both absolute numbers and relative proportion (LARUE *et al.* 2021). Therefore, the notion of 'major' and 'minor' introns or spliceosomes has been abandoned in favour of U2-type and U12-type. Note also that based on intron boundaries, one cannot infer with certainty whether the intron is spliced by a U12 or a U2-spliceosome (SHARP AND BURGE 1997).

Both U2- and U12-type introns and their cognate spliceosomes are believed to derive from a common eukaryotic ancestor (RUSSELL *et al.* 2006). This view is corroborated by the recent publication of the draft nuclear genome sequence from *Andalucia godoyi*, a slowly-evolving member of the early-branching Discoba supergroup (GRAY *et al.* 2020), whose nuclear genome encodes exclusively U RNA counterparts of the U2-type spliceosome. The same is true for the rapidly evolving discoban group, kinetoplastids, although their U RNAs are highly derived (GÜNZL 2010).

The spectrum of U-RNA variants across eukaryotes is likely much larger than currently appreciated. Although U RNAs are best identified using covariance models (CM) that capture the conserved, distinct sequence features and higher-order structure of U RNAs, recognition of homologs from poorly studied eukaryotic groups can still be challenging because available CMs (e.g., those compiled in the RFAM database (KALVARI *et al.* 2021)) are built with a taxonomically biased set of sequences.

The spliceosome not only catalyzes *cis*-splicing, i.e., intron excision and exon joining within the same pre-mRNA molecule, but also ***trans*-splicing** by which separate transcripts are joined. More specifically, in some metazoan groups and in euglenozoans including *Diplonema*, a short (~15-50 nt) leader sequence termed spliced-leader (SL) RNA is added to the 5′ end of pre-mRNAs. SL-*trans*-splicing in euglenozoans provides a 5′ cap structure to mRNAs and resolves long polycistronic transcripts into single-gene RNAs (HASTINGS 2005). Among euglenozoans, the spliceosome of trypanosomes, and in particular *Trypanosoma brucei*, has been studied in great detail. It belongs to the U2-type and is responsible for both the predominant SL-*trans*-splicing, as well as for *cis*-splicing of the rare introns (the *T. brucei* nuclear genome contains only two introns, one in a gene encoding a poly(A)-polymerase and the other in an RNA-helicase gene) (MICHAELI 2011).

RESULTS AND DISCUSSION

Intron types and spliceosome components in *D. papillatum*

The large majority of **introns** in the *D. papillatum* nuclear genome assembly are canonical, bearing GT at their 5′-end and AG at the 3′-end (U2-type). In addition, non-canonical introns with GC-AG splice-site combinations were also detected. As in the case of numerous animals, fungi, and plants (FREY AND PUCKER 2020), GC-AG introns from *Diplonema* are most likely spliced by the same major U2-type spliceosome as GT-AG introns.

The lack of U12-type spliceosomal introns (AT-AC and variants thereof) in *Diplonema* is consistent with the lack of the U4atac, U6atac, U11, and U12 **RNAs** among the set of spliceosomal RNAs detected. To guarantee a highly sensitive identification of U RNAs, we used the corresponding RFAM CMs (KALVARI *et al.* 2021), and constructed new CM versions representing the largest range possible for eukaryotes (for details, see Methods). Compared to the RFAM models, our

improved CMs yielded significantly higher scores in identifying U RNAs of *Diplonema* and across eukaryotes as a whole, which strongly suggests the absence of a U12-type spliceosome in *D. papillatum*.

To identify potential structural variations of the five *Diplonema* spliceosomal RNAs, we designed two-dimensional (2D) diagrams following the characteristics of the human and yeast counterparts for which atomic structures are available ((YAN *et al.* 2015; ZHANG *et al.* 2017) (**Supplementary Figure S10**). Contrary to the situation in kinetoplastids (GÜNZL 2010), all five U-RNAs from *Diplonema* closely resemble their homologs from human and yeast U2-type spliceosomes regarding conserved sequence motifs and 2D structure. This lends further support to the view that the U2-type spliceosomal machinery has an ancient evolutionary origin and generally underwent only minor changes except in certain extremely fast-evolving species such as *Trypanosoma*.

Conservation of **spliceosomal proteins** is far more difficult to assess and interpret. According to the most recent data in the UniProt database (UNIPROTKB 2021), more than 200 spliceosomal proteins have been identified in human, but only 67 in yeast. When searching the complete set of *Diplonema* proteins for homologs of the yeast spliceosomal proteins using the highly sensitive Hidden Markov model (HMM) search algorithm (EDDY 2011), not more than a half returned hits below a confidence E-value threshold of 1.0 e-5. Among these, it was often difficult to distinguish between true orthologs and other members of the corresponding protein family, or even to detect the mere presence of conserved protein domains. This leaves U-RNA sequences and structures as the only clear-cut criterion for diagnosing the presence of U2- versus U12-type spliceosomes in organisms that are phylogenetically distant from human and yeast. In summary, from the intron-boundary sequences and U RNA features, we conclude that *Diplonema* possesses a single kind of spliceosome, the U2-type spliceosome.

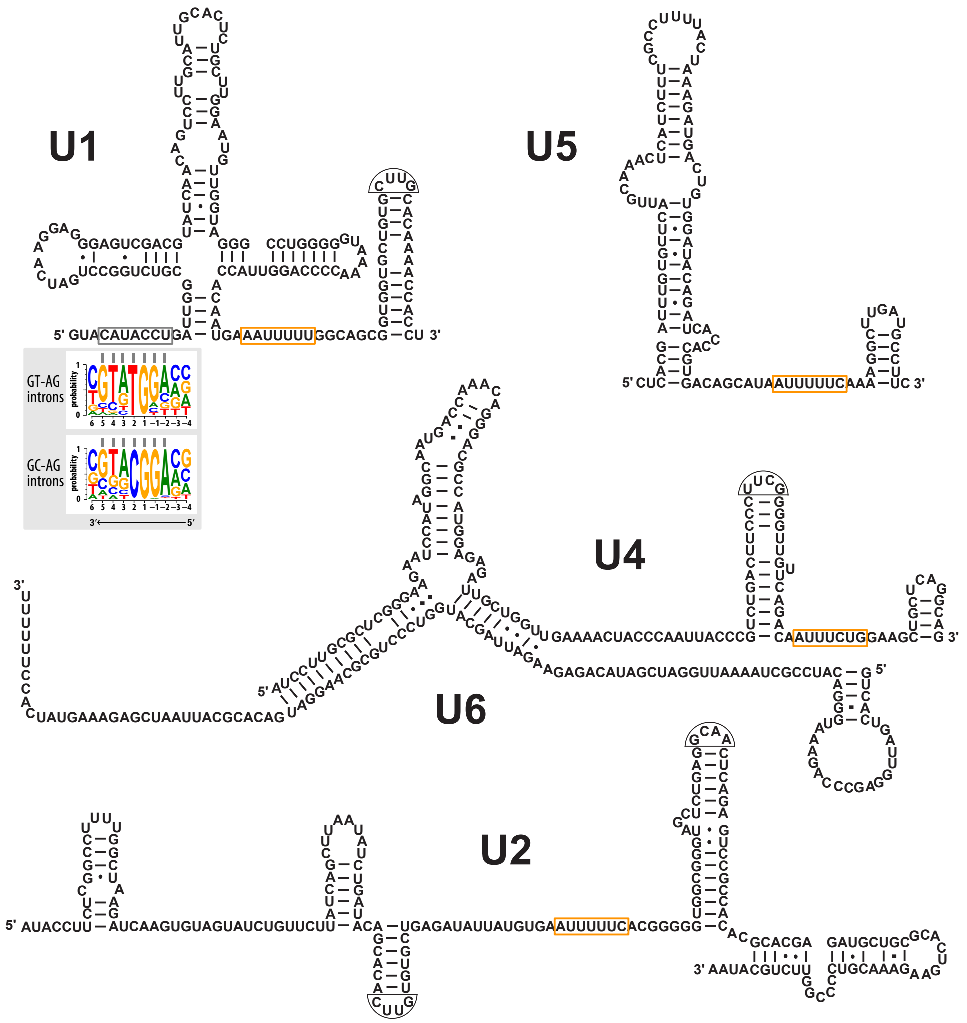

Supplementary Figure S10. 2D structure diagrams of the five *Diplonema* spliceosomal RNAs. The 2D structures were drawn following the conserved sequence and helical structure conservation in the alignments of respective CMs, and in a style found in many publications including (ZHAO *et al.* 2018). Orange rectangles highlight putative Sm protein-binding sites. The grey rectangle indicates the 5′ splice-site recognition motif in U1 RNA; the potential base-pairing interactions between the motif and 5′ splice sites of GT-AG and GC-AG introns are shown in the grey box.

Spliced-leader RNA genes

The gene encoding the SL RNA in *D. papillatum* was determined already two decades ago (STURM *et al.* 2001). It is 114 bp-long and includes in its 5′-portion the 39-bp non-coding exon (outron) that will be covalently linked to the 5′- terminus of pre-mRNAs *via trans*-splicing. We found as many as 109 SL-RNA gene copies (at ≥95% sequence identity) in the *Diplonema* nuclear genome assembly. All these genes are part of a tandem repeat, which also contains the 5S rRNA and U2 snRNA genes and occurs in multiple contigs **(Supplementary Figure S11)** (see also the Supplementary Information on repeats). This particular gene organization appears to be shared by all euglenozoans (KELLER *et al.* 1992; SANTANA *et al.* 2001; STURM *et al.* 2001). Our nuclear genome assembly confirms the earlier conjecture that all SL-RNA genes of *Diplonema* occur in SL-5S-U2 repeat clusters. However, we note that hundreds of fragments of the SL RNA gene—a vast majority representing partial 39 bp-long exon portion—are spread across the genome. These do not appear to be functional because the SL sequences capping mRNAs are identical in *Diplonema*, unlike in some dinoflagellates (ALACID *et al.* 2022). Furthermore, because virtually all SL gene fragments occur in intergenic regions and introns of protein-coding genes, we hypothesize that they arose as a collateral damage of retrotransposon mobility, i.e., reverse transcription of mRNAs.

When inspecting mRNA sequences in RNA-Seq data, we realized that the 5′-most A (indicated in lower case in the sequence: 5′–aACCAACGATTTAAAAGCTACAGTTTCTGTACTTTATTG–3′) of the SL sequence was consistently missing. Apparently, the 5′-A fails to be synthesized during the reverse-transcription step of RNA-Seq library construction, because of nucleotide modifications that diplonemids most probably share with kinetoplastids: studies in trypanosomes have revealed a distinctive cap-4 structure at the 5′-terminus of their SL RNA that arises from the methylation of seven sites within its first four nucleotides (AACU). More specifically, the cap consists of m^7guanosine -ppp- N^6,N^6,2′-*O*-trimethyladenosine -p- 2′-*O*-methyladenosine -p- 2′-*O*-methylcytosine -p- N^3,2′-*O*-methyluridine, of which the m^7guanosine is added by a guanylyltransferase, and the other nucleotides are chemically modified *in situ* (BANGS *et al.* 1992).

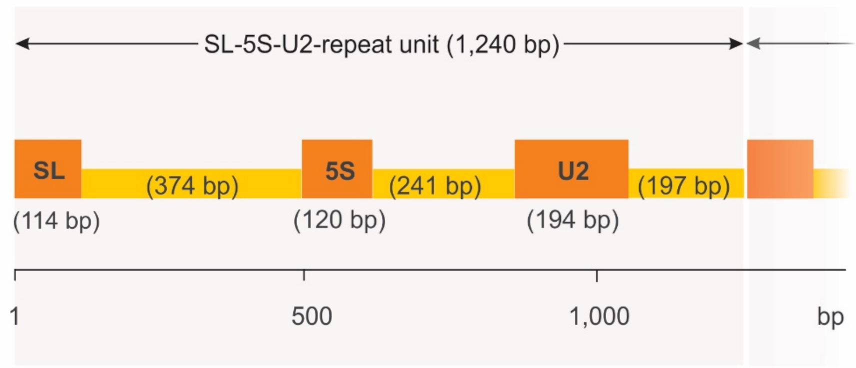

Supplementary Figure S11. Repeat unit including the spliced-leader RNA gene of *D. papillatum*. Genes are represented as orange rectangles. Transcribed spacers are shown as dark yellow bars. The sizes of the elements are shown in parentheses. The SL-5S-U2-repeat unit comprises the genes specifying the spliced-leader RNA (SL), the 5S rRNA (5S), and the U2 spliceosomal RNA.

Spliced-leader *trans*-splicing

We identified spliced-leader *trans*-splicing (SLTS) sites by an automated procedure that searched for SL-sequence matches in soft-clipped segments of RNA-Seq reads (see Materials and Methods). Of the 23,720 SLTS sites detected in the genome assembly, about half are located exactly at the 5′ end of gene models, which is expected since SLTS sites were used in the annotation procedure as one of the hints supporting protein-coding gene models. The vast majority of the remaining sites occurred in close vicinity of gene starts, which indicates that many genes possess multiple SLTS sites. The site closest to the 5′ end of a gene model is typically the highest scoring one and thus represents the primary SLTS site.

According to this automated search, only ~41% of *Diplonema* gene models possess at least one associated upstream SLTS site. This suggested initially that only a fraction of the *Diplonema* protein-coding genes require the attachment of an SL, as for example, in *Drosophila* (LASDA AND BLUMENTHAL 2011). Yet, this observation contrasts with the situation in kinetoplastids, in which an SL is *trans*-spliced to each protein-coding gene transcript. Therefore, we re-examined the RNA-Seq read alignment to the three longest and expert-validated contigs of the *D. papillatum* assembly (see Supplementary Information on *D. papillatum* genome assembly and annotation). Upon visual inspection, we found that 90% of the gene models had at least one upstream SLTS site (with 14% of genes having multiple sites). The principal reason for SLTS sites not being recognized by the automated procedure was the short length (≤10 bp) of the soft-clipped SL portion in RNA-Seq reads. About half of the remaining gene models with a seemingly absent SLTS site were preceded by repeats or low-complexity regions that complicated read mapping. The other half had an RNA-Seq read coverage that was low throughout or decreased progressively towards the 5′ end of the gene model. Based on these observations, we conclude that all cytosolic mRNAs of *Diplonema* are decorated with an SL.

Sequence context of acceptor and donor splice sites in *cis*- and *trans*-spliced introns

In kinetoplastids, a large body of experimental data document that *trans*-splicing of the SL sequence requires only a few SLTS-specific factors, but otherwise, the set of spliceosomal proteins is the same as for splicing (the few) *cis* introns (reviewed in (MICHAELI 2011)). Further, the AG-splice acceptor motif in kinetoplastids has a slight preference for an upstream C residue (DILLON *et al.* 2015). In *Diplonema*, we observed a similar trend at the corresponding position and a bias toward a G residue immediately downstream of the AG acceptor (**Supplementary Figure S12A**). We did not note significant differences in sequence composition between primary and non-primary sites or between automated and expert-curated datasets within a window of 30 bp up and downstream of the SLTS sites.

In *D. papillatum,* the upstream-sequence context of the AG-splice-acceptor from *trans*-spliced introns is very similar to that of *cis*-spliced introns, only that the latter have an even more pronounced preference for the upstream C (**Supplementary Figure S12B**). In contrast, the downstream sequences differ, which is expected because they fall into functionally different regions, 5′ UTR and CDS, which in *Diplonema* are slightly more A+G- and C+G-rich, respectively (**Supplementary Figure S12A,B**).

The acceptor site motif of *cis*-introns in *Diplonema* conforms to the 5′–YAG–3′ motif largely conserved across eukaryotes (**Supplementary Figure S12C**). In contrast, the GT-donor site motif of the *Diplonema* GT-type *cis*-introns is slightly more divergent, albeit still within the range of variation observed in other organisms (**Supplementary Figure S12D,E**). The only atypical aspect in *D. papillatum* is a higher than usual frequency of A and G at two positions just upstream of the donor site motif. Interestingly, in almost all of the few GC-AG-type *cis*-introns, the GT-donor site is preceded by an AG (**Supplementary Figure S12F**).

The 5′ splice-site recognition motif of the *Diplonema* U1 RNA is 5′–**CAUACCU**–3′ (see box in **Supplementary Figure S10**), which is reverse complementary to the motif 5′–**aGGTaTG**–3′ at the 5′ boundary of GT-AG introns (lower case indicates less conserved positions). In GC-AG introns of *D. papillatum*, the AG-splice acceptor context is 5′–**AGGCatg**–3′. Thus, the **A-C** mismatch between the **A**4 of the U1-RNA motif and splice acceptor region of GC-AG introns is compensated for by base pairing between **U**7 of the spliceosomal RNA and a strongly conserved **A** in the adjacent exon of this intron type.

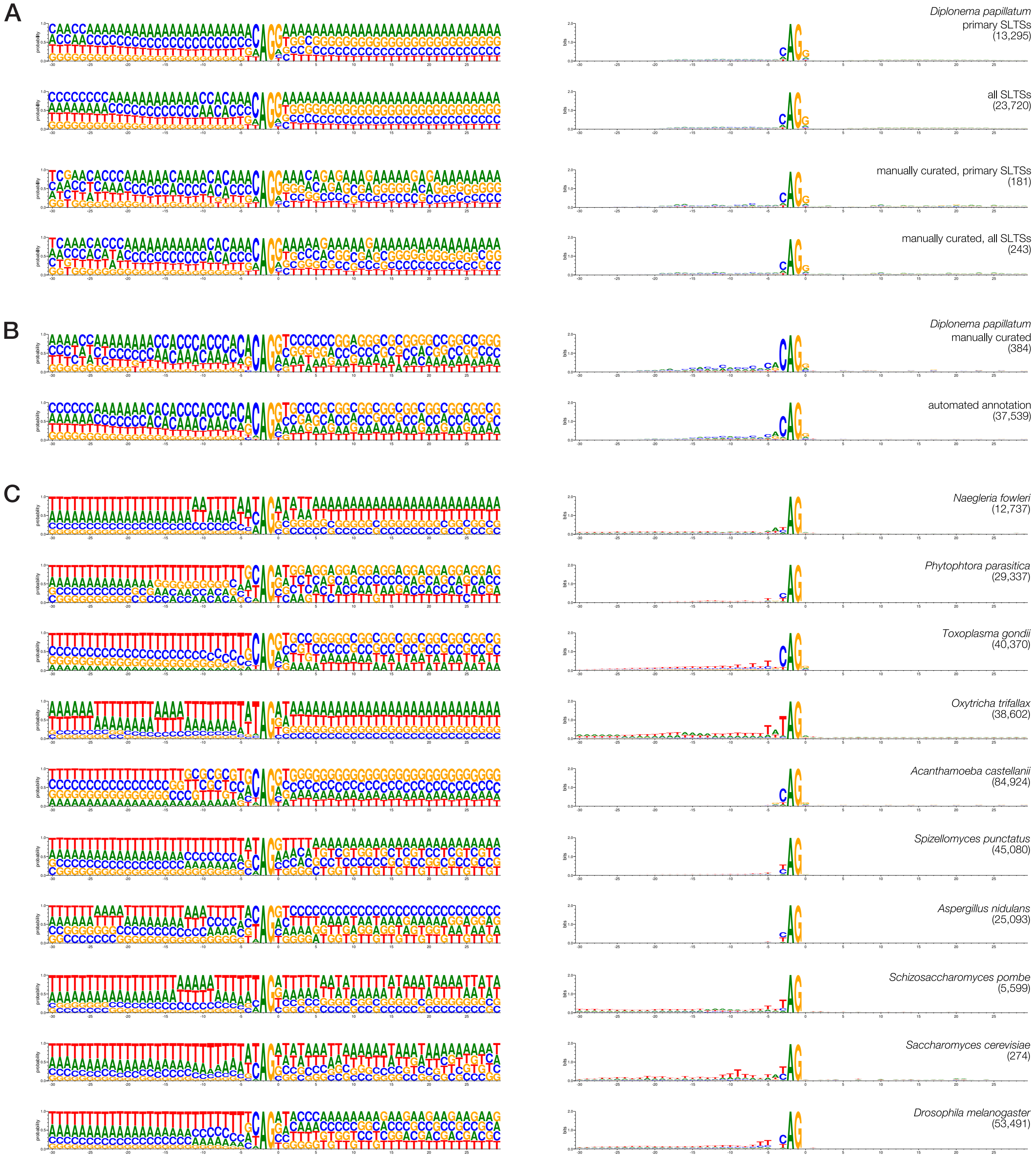

Supplementary Figure S12. Sequence logos of splicing acceptor and donor motifs in *Diplonema* and other eukaryotes. Sequence logos are displayed as probabilities or bits (left and right column, respectively). The number of analyzed unique sequences is indicated in parentheses. **A**, Spliced-leader *trans*-splicing (SLTS) acceptor sites in *D. papillatum*. **B**, *Cis*-intron acceptor sites in *D. papillatum*. **C**, *Cis*-intron acceptor sites in selected eukaryotes (see Methods).

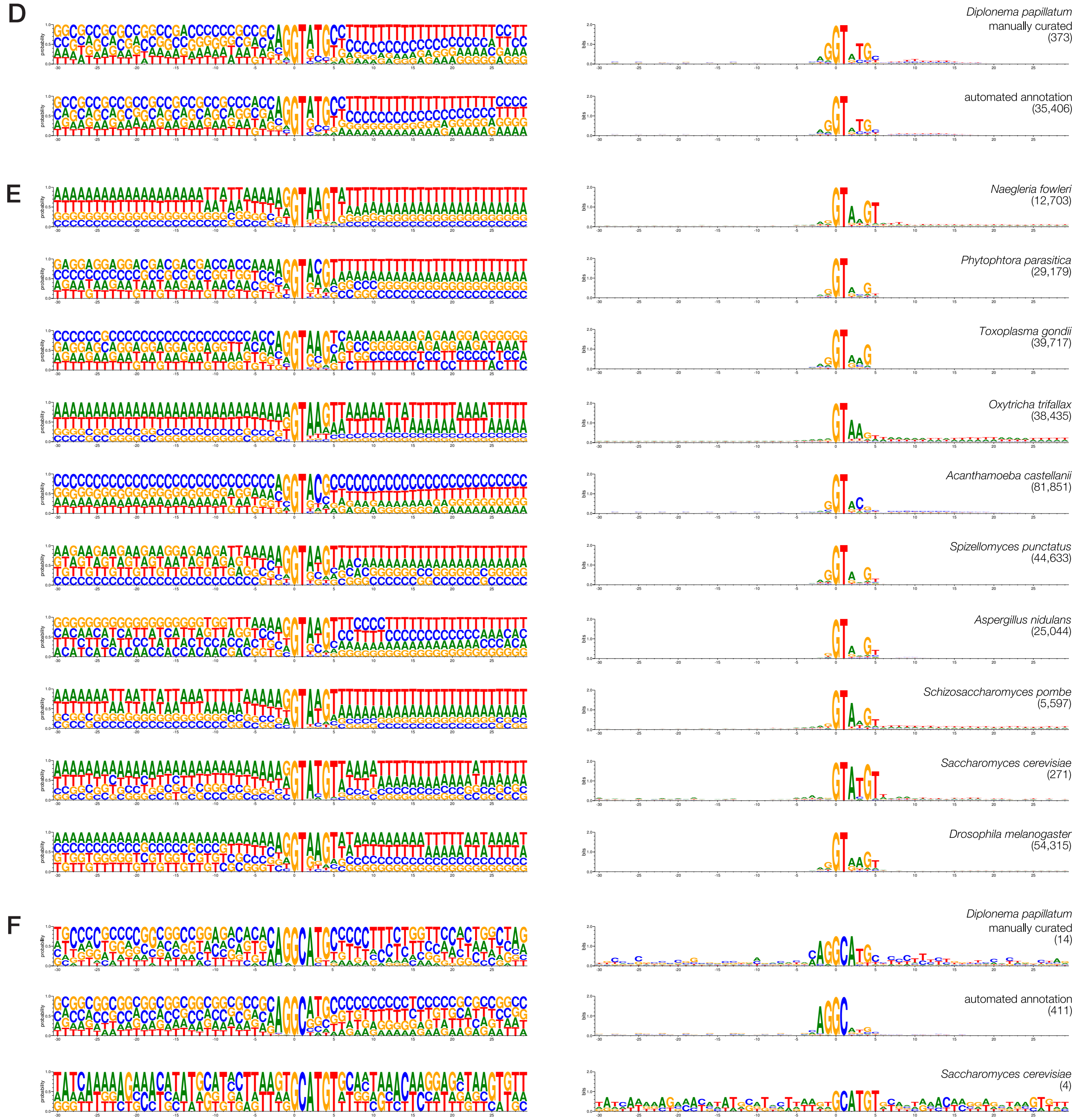

Supplementary Figure S12, continued. Sequence logos of splicing acceptor and donor motifs in *Diplonema* and other eukaryotes. Sequence logos are displayed as probabilities or bits (left and right column, respectively). The number of analyzed unique sequences is indicated in parentheses. **D**, GT-type *cis*-intron donor sites in *D. papillatum*. **E**, GT-type *cis*-intron donor sites in selected eukaryotes. **F**, GC-type *cis*-intron donor sites in *D. papillatum* and *Saccharomyces cerevisiae*.

STRUCTURAL RNAS INVOLVED IN PROTEIN SYNTHESIS

Ribosomal RNA genes and rDNA clusters

In eukaryotes, the RNA components of the cytosolic ribosomes are encoded by the following genes: *rns* or 18S associated with the small subunit (SSU), and *rnl* or 25S–28S, *rrn5.8* or 5.8S, and *rrn5* or 5S associated with the large subunit (LSU). The genes *rns*, *rrn5.8*, and *rnl* are arranged in tandem, forming the classical rDNA repeat from which all three components are co-transcribed. In the *D. papillatum* genome assembly, an rDNA repeat unit plus the adjacent spacer has a total length of

7,628 bp (**Supplementary Figure S13**). The assembly includes a total of 21 such rDNA units (at ≥90% sequence identity) with contigs containing up to two adjacent ribosomal DNA clusters situated in most cases at contig boundaries. This arrangement indicates that in reality, the clusters are more extended but were not assembled due to multiple conflicting solutions.

As mentioned above and similar to the situation in other eukaryotes, the gene of the cytosolic 5S rRNA component is not part of the *rns*-*rrn5.8*-*rnl* rDNA repeat unit but is rather located in a separate repeat unit.

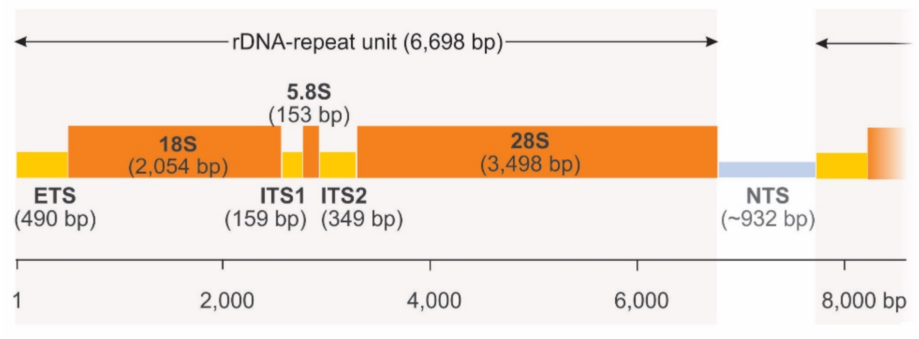

Supplementary Figure S13. The ribosomal DNA-repeat unit of *D. papillatum*. Genes are represented as orange rectangles. Transcribed spacers are shown as dark yellow bars, and non-transcribed spacer regions as light-blue bars. The sizes of the elements is shown in parentheses. The rDNA-repeat unit consists of the external transcribed spacer (ETS), two internal transcribed spacers (ITS1, ITS2), and the genes encoding the small subunit rRNA (18S), the 5.8S rRNA (5.8S), and the large subunit rRNA (28S). The non-transcribed spacer (NTS) separates the tandemly arranged rDNA repeat units. Note that in other euglenozoans (kinetoplastids, euglenids), post-transcriptional excision of additional ITSs generates more fragmented cytosolic LSU rRNAs (for details, see for example (Matzov *et al.* 2020)).

Transfer RNA genes and codon usage

A stringent tRNA-SE scan retrieved 211 tRNA genes in the *D. papillatum* genome assembly. This gene set allows the recognition of all standard amino acids. Several tRNA genes occur in multiple identical copies with numbers varying from two (trnA(UGC)) to 10 (trnK(CUU)). Further, up to five tRNA genes have the same anticodon but a distinct sequence (e.g., trnR(ACG)). The set of tRNA genes represents 47 out of 64 possible anticodons. Isoacceptor tRNAs missing from the *Diplonema* gene complement coincide with those that have been reported absent from many species either across all domains of life or throughout eukaryotes (Ehrlich *et al.* 2021) (**Supplementary Table S13**). The only exception is the apparent absence of trnL(UAA). It could either be missing in the *D. papillatum* genome assembly, or be a diplonemid or *D. papillatum*-specific gene loss. Since TTA codons do exist in essential nucleus-encoded and mitochondrion-encoded protein-coding genes of *Diplonema*, these codons might be decoded by tRNAs with anticodons other than UAA and carrying nucleotide modifications (Jackman and Alfonzo 2013). Transfer RNA^{Leu}(UAA) is most likely functionally substituted by one of the tRNA^{Leu}(CAA) —for which two identical gene copies exist— provided C34, the wobble position, has been post-transcriptionally deaminated to U. In *Trypanosoma*, an adenosine deaminase complex acting on tRNAs (ADAT2/3) is responsible not only for A-to-I, but also for a C-to-U modification in trnT(AGU) (Rubio *et al.* 2006; Rubio *et al.* 2017). The *D. papillatum* genome encodes homologs of both deaminase subunits (DIPPA_16350: ADAT2, and DIPPA_33161: ADAT3), which would allow *Diplonema* to compensate for the missing anticodon by tRNA modification. Even if the process were not efficient, this limitation might not be detrimental since UUA is the most rarely used codon across all *Diplonema* nuclear genes (**Supplementary Table S14**). Not only UUA, but A+U-rich codons in general are under-represented, with an A+T content of *Diplonema*'s nuclear coding sequences amounting to only 38.72%. The bias towards G+C-rich codons is especially prominent at the third position (74.74%).

Supplementary Table S13. Missing genes for specific isoacceptor tRNAs in the *D. papillatum* genome assembly[a]

**Anticodon**	AAA	ACA	ACC	ACU	AUA	AUC	AUG	AUU	GAC	GAG	GAU	GCG	GGA	GGC	GGG	GGU	UAA
**Amino acid**	Phe	Cys	Gly	Ser	Tyr	Asp	His	Asn	Val	Leu	Ile	Arg	Ser	Ala	Pro	Thr	Leu
**Taxa**[b]	A,B,E	A,B,E	A,B,E	A,B,E	A,B,E	A,B,E	A,B,E	A,B,E	E	E	E	E,B	E	E	E	E	*D. p.*

[a] Data from taxa other than *D. papillatum* taken from (Ehrlich *et al.* 2021)
[b] A, Archaea, B, Bacteria, E, Eukaryota, *D. p.*, *D. papillatum*, this report.

Supplementary Table S14. **Codon frequency of *D. papillatum* nuclear protein-coding genes**[a]

F	TTT	0.22	S	TCT	0.09	Y	TAT	0.14	C	TGT	0.18
F	TTC	0.77	S	TCC	0.19	Y	TAC	0.85	C	TGC	0.81
L	TTA	0.02	S	TCA	0.08	*	TAA	0.18	*	TGA	0.53
L	TTG	0.15	S	TCG	0.30	*	TAG	0.28	W	TGG	1.00
L	CTT	0.11	P	CCT	0.15	H	CAT	0.20	R	CGT	0.09
L	CTC	0.30	P	CCC	0.26	H	CAC	0.80	R	CGC	0.28
L	CTA	0.03	P	CCA	0.12	Q	CAA	0.30	R	CGA	0.12
L	CTG	0.37	P	CCG	0.46	Q	CAG	0.69	R	CGG	0.28
I	ATT	0.20	T	ACT	0.11	N	AAT	0.18	S	AGT	0.06
I	ATC	0.69	T	ACC	0.33	N	AAC	0.81	S	AGC	0.26
I	ATA	0.10	T	ACA	0.15	K	AAA	0.28	R	AGA	0.08
M	ATG	1.00	T	ACG	0.39	K	AAG	0.71	R	AGG	0.13
V	GTT	0.14	A	GCT	0.14	D	GAT	0.21	G	GGT	0.13
V	GTC	0.39	A	GCC	0.31	D	GAC	0.78	G	GGC	0.43
V	GTA	0.07	A	GCA	0.15	E	GAA	0.40	G	GGA	0.15
V	GTG	0.38	A	GCG	0.38	E	GAG	0.59	G	GGG	0.27

[a] Coding regions of 21,747 assembled mRNAs carrying a 5′ spliced leader

From among the three usual stop codons, UGA appears to be the most frequent in *D. papillatum* (**Supplementary Table S14**). Yet, in addition to the set of genes encoding tRNAs that load standard amino acids, the genome assembly also contains two distinct genes specifying **selenocysteine** tRNA (tRNA^{Sec}(UGA)). In the nascent polypeptide chain, selenocysteine incorporation at UGA codons that otherwise signal translation termination is directed by a particular structural signal in the mRNA (COMMANS AND BÖCK 1999). However, automated gene predictors do not recognize such signals and will infer truncated open reading frames in genes containing selenocysteine codons. Therefore, we inspected the gene models of two proteins known to contain Sec, notably Selenoprotein SelG (DIPPA_01860) and glutathione peroxidase BsaA (DIPPA_26877). Compared to homologs from other taxa, the reading frame of DIPPA_01860 was slightly shorter in the C-terminal region, whereas that of DIPPA_26877 was considerably shorter in its N-terminal region. This is because the start codon chosen as the N-terminus of the latter gene was the first ATG codon downstream of the TGA codon. Replacing the TGA-stop codons in the conceptual translations by "X" clearly improved the alignment of the *Diplonema* inferred proteins with counterparts from other eukaryotes (**Supplementary Figure S14**). This strongly suggests that the cytosolic translation in *D. papillatum* indeed decodes certain TGA codons as Sec.

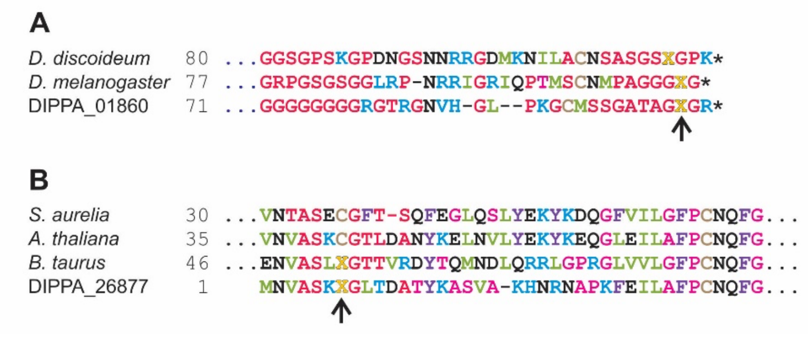

Supplementary Figure S14. Multiple sequence alignments of selenocysteine-containing proteins. The residue selenocysteine is represented by an 'X'. **A**, Selenoprotein SelG. *D. discoideum*, *Dictyostelium discoideum* (sp|Q55EX3); *D. melanogaster*, *Drosophila melanogaster* (sp|Q7Z2C4), and the tentative homolog from *D. papillatum*. **B**, Glutathione peroxidase BsaA/GPX. *S. aureus*, *Staphylococcus aureus* (sp|Q6GHD0); *A. thaliana*, *Arabidopsis thaliana* (sp|O04922); *B. taurus*, *Bos taurus* (sp|P00435); and the homolog from *D. papillatum.*

Finally, one tRNA gene was identified to carry a UUA anticodon, classifying it as an ochre suppressor codon. However, the predicted codon recognition of this gene is at odds with the observation that the TAA termination codon is used frequently in *D. papillatum* nuclear genes (as well as mitochondrial genes that are translated exclusively with imported, nucleus-encoded tRNAs). Since this potential suppressor-tRNA is an abundant transcript with an RNA-Seq read coverage higher than that of many regular tRNA genes, it must play another yet unknown role, for instance, as a regulator of certain biological processes (RAINA AND IBBA 2014). It should be noted that we only detected transcripts comprising the 5′ half of tRNA(UUA). Such tRNA fragments could arise by premature transcription (e.g., termination at modified bases) during RNA-Seq library construction since we also observed this phenomenon in certain regular *Diplonema* tRNAs. However, the 5′ half of tRNA(UUA) could also be a tRNA-derived RNA fragment (tRFs) produced by specific RNase processing (MEGEL

et al. 2015) and function for example, as a signaling molecule in stress response or as a regulator of gene silencing (MARTINEZ *et al.* 2017).

METHODS

Construction of covariance models used for the identification of spliceosomal RNAs

Most covariance models (CM) available in the RFAM database ((KALVARI *et al.* 2021); https://rfam.xfam.org/) have been built with sequences related to biochemically well-investigated species such as human, yeast and *Arabidopsis*, i.e., they are biased towards animals, fungi, and plants. The bias in taxon sampling makes these models less sensitive when attempting to identify structural RNAs in evolutionarily remote protists, leaving some homologs unrecognized due to structural variation or low sequence conservation. To improve CM sensitivity, we have assembled a broad, taxonomically balanced collection of nuclear genome sequences from GenBank, including select representatives from each of the major eukaryotic groups. For example, Metazoa are represented by five taxa: human, *C. elegans*, a cnidarian, a demosponge, and a placozoan. The final collection includes a total of 207 eukaryotic genome assemblies. The first step of building more sensitive CMs involved searching our genome collection with *cmsearch* (Infernal package; (NAWROCKI AND EDDY 2013)) using a given RFAM model. The –A option of *cmsearch* was applied to produce structured nucleotide sequence alignments of the search results, from which only the first best hit was retained for each species. The alignment was visually inspected, manually corrected for apparent errors, and used to build a new CM with a balanced eukaryotic taxon sampling. For further refinement, the process of searching, alignment, manual curation and CM building was repeated three times. The final CMs almost always have elevated scores for detecting homologs across eukaryotes, particularly in protists. The CMs of spliceosomal U RNAs built by us are available upon request.

Protein sequence search and identification

The identification of protein function was performed as detailed in the **Supplementary Information**: Section 2. Assembly and annotation of the nuclear genome and transcriptome of *Diplonema papillatum*.

Spliced-leader *trans*-splicing acceptor site detection

We detected SLTS sites by exploiting soft-clipped regions of RNA-Seq reads mapped onto the nuclear genome assembly. The SAM file of poly-A RNA reads for a given contig was parsed to retrieve soft-clipped sequences that exactly matched at least 11 of the 3′ terminal nucleotides of the SL sequence (5′–ACCAACGATTTAAAAGCTACAGTTTCT**GTACTTTATTG**–3′).
To each SLTS site we assigned a score corresponding to the number of instances that the soft-clipped sequence matched. If multiple SLTS sites were detected within a 5-kbp region, only a single representative (the one with the highest score) site was selected. The portion of the read that had not been soft-clipped was used to determine the position of the SLTS site in the contig sequence. Because the acceptor site sequence for the SL RNA is AG in the pre-mRNA and the terminal nucleotide of the SL sequence is a G, the SLTS site annotation was placed at the G of the mapped AG acceptor site. Although the G nucleotide is not encoded by the gene but originates from the trans-spliced SL, this choice of representing the SL allowed us to determine an SLTS site presence quickly, because all protein-coding gene models for which we detected an SLTS site start with a G. For statistical purposes, only unique SLTS sites were considered, i.e., if the region 60 bp up and downstream of the detected site was identical (e.g., tandem gene repeats), the site was counted only once.

Genome analysis — Splice-site collection and logo generation

Splice-site datasets of protein-coding genes were mainly collected from the VEuPathDB (https://veupathdb.org) (AMOS *et al.* 2021), except from datasets for *Drosophila melanogaster* (https://flybase.org; (LARKIN *et al.* 2021)), *Saccharomyces cerevisiae* (https://www.yeastgenome.org; (NG *et al.* 2020)), and *Oxytricha trifallax* (https://oxy.ciliate.org; (SWART *et al.* 2013)). Based on available annotations, we retrieved exon-intron boundaries together with up- and downstream flanking regions of ~30 bp, and extracted a sequence representative for each splice site. The sequence logos were created with WebLogo v3.7.4 (CROOKS *et al.* 2004).

AUTHOR CONTRIBUTIONS

Conceptualization, Data curation, Investigation, Formal analysis, Visualization, Writing, original draft – B.F.L., G.B., M.V.; **Writing, review & editing –** all co-authors.

REFERENCES

Alacid, E., N. A. T. Irwin, V. Smilansky, D. S. Milner, E. S. Kilias *et al.*, 2022 A diversified and segregated mRNA spliced-leader system in the parasitic Perkinsozoa. Open Biol 12**:** 220126.

Amos, B., C. Aurrecoechea, M. Barba, A. Barreto, E. Y. Basenko *et al.*, 2021 VEuPathDB: the eukaryotic pathogen, vector and host bioinformatics resource center. Nucleic Acids Res.

Bangs, J. D., P. F. Crain, T. Hashizume, J. A. McCloskey and J. C. Boothroyd, 1992 Mass spectrometry of mRNA cap 4 from trypanosomatids reveals two novel nucleosides. J Biol Chem 267**:** 9805-9815.

Commans, S., and A. Böck, 1999 Selenocysteine inserting tRNAs: an overview. FEMS Microbiology Reviews 23**:** 335-351.

Crooks, G. E., G. Hon, J. M. Chandonia and S. E. Brenner, 2004 WebLogo: a sequence logo generator. Genome Res 14**:** 1188-1190.

Dillon, L. A., K. Okrah, V. K. Hughitt, R. Suresh, Y. Li *et al.*, 2015 Transcriptomic profiling of gene expression and RNA processing during *Leishmania major* differentiation. Nucleic Acids Res 43**:** 6799-6813.

Eddy, S. R., 2011 Accelerated profile HMM searches. PLoS Comput Biol 7**:** e1002195.

Ehrlich, R., M. Davyt, I. López, C. Chalar and M. Marín, 2021 On the track of the missing tRNA genes: a source of non-canonical functions? Front Mol Biosci 8**:** 643701.

Frey, K. A.-O., and B. A.-O. Pucker, 2020 Animal, fungi, and plant genome sequences harbor different non-canonical splice sites. Cells 9**:** 458.

Gray, M. W., G. Burger, R. Derelle, V. Klimeš, M. M. Léger *et al.*, 2020 The draft nuclear genome sequence and predicted mitochondrial proteome of *Andalucia godoyi*, a protist with the most gene-rich and bacteria-like mitochondrial genome. BMC Biol 18**:** 22.

Günzl, A., 2010 The pre-mRNA splicing machinery of trypanosomes: complex or simplified? Eukaryot Cell 9**:** 1159-1170.

Hastings, K. E., 2005 SL trans-splicing: easy come or easy go? Trends Genet 21**:** 240-247.

Jackman, J. E., and J. D. Alfonzo, 2013 Transfer RNA modifications: nature's combinatorial chemistry playground. Wiley Interdiscip Rev RNA 4**:** 35-48.

Kalvari, I., E. P. Nawrocki, N. Ontiveros-Palacios, J. Argasinska, K. Lamkiewicz *et al.*, 2021 Rfam 14: expanded coverage of metagenomic, viral and microRNA families. Nucleic Acids Res 49**:** D192-d200.

Keller, M., L. H. Tessier, R. L. Chan, J. H. Weil and P. Imbault, 1992 In *Euglena*, spliced-leader RNA (SL-RNA) and 5S rRNA genes are tandemly repeated. Nucleic Acids Res 20**:** 1711-1715.

Larkin, A., S. J. Marygold, G. Antonazzo, H. Attrill, G. Dos Santos *et al.*, 2021 FlyBase: updates to the *Drosophila melanogaster* knowledge base. Nucleic Acids Res 49**:** D899-d907.

Larue, G. E., M. Eliáš and S. W. Roy, 2021 Expansion and transformation of the minor spliceosomal system in the slime mold *Physarum polycephalum*. Curr Biol 31**:** 3125-3131.e3124.

Lasda, E. L., and T. Blumenthal, 2011 Trans-splicing. Wiley Interdiscip Rev RNA 2**:** 417-434.

Martinez, G., S. G. Choudury and R. K. Slotkin, 2017 tRNA-derived small RNAs target transposable element transcripts. Nucleic Acids Res 45**:** 5142-5152.

Matzov, D., M. Taoka, Y. Nobe, Y. Yamauchi, Y. Halfon *et al.*, 2020 Cryo-EM structure of the highly atypical cytoplasmic ribosome of *Euglena gracilis*. Nucleic Acids Res 48**:** 11750-11761.

Megel, C., G. Morelle, S. Lalande, A. M. Duchêne, I. Small *et al.*, 2015 Surveillance and cleavage of eukaryotic tRNAs. Int J Mol Sci 16**:** 1873-1893.

Michaeli, S., 2011 Trans-splicing in trypanosomes: machinery and its impact on the parasite transcriptome. Future Microbiol 6**:** 459-474.

Nawrocki, E. P., and S. R. Eddy, 2013 Infernal 1.1: 100-fold faster RNA homology searches. Bioinformatics 29**:** 2933-2935.

Ng, P. C., E. D. Wong, K. A. MacPherson, S. Aleksander, J. Argasinska *et al.*, 2020 Transcriptome visualization and data availability at the *Saccharomyces* Genome Database. Nucleic Acids Res 48**:** D743-d748.

Patel, A. A., and J. A. Steitz, 2003 Splicing double: insights from the second spliceosome. Nat Rev Mol Cell Biol 4**:** 960-970.

Raina, M., and M. Ibba, 2014 tRNAs as regulators of biological processes. Frontiers in Genetics 5.

Rubio, M. A., K. W. Gaston, K. M. McKenney, I. M. Fleming, Z. Paris *et al.*, 2017 Editing and methylation at a single site by functionally interdependent activities. Nature 542**:** 494-497.

Rubio, M. A., F. L. Ragone, K. W. Gaston, M. Ibba and J. D. Alfonzo, 2006 C to U editing stimulates A to I editing in the anticodon loop of a cytoplasmic threonyl tRNA in *Trypanosoma brucei*. J Biol Chem 281**:** 115-120.

Russell, A. G., J. M. Charette, D. F. Spencer and M. W. Gray, 2006 An early evolutionary origin for the minor spliceosome. Nature 443**:** 863-866.

Santana, D. M., J. Lukeš, N. R. Sturm and D. A. Campbell, 2001 Two sequence classes of kinetoplastid 5S ribosomal RNA gene revealed among bodonid spliced leader RNA gene arrays. FEMS Microbiology Letters 204**:** 233-237.

Sharp, P. A., and C. B. Burge, 1997 Classification of introns: U2-type or U12-type. Cell 91**:** 875-879.

Sturm, N. R., D. A. Maslov, E. C. Grisard and D. A. Campbell, 2001 *Diplonema* spp. possess spliced leader RNA genes similar to the Kinetoplastida. J Eukaryot Microbiol 48**:** 325-331.

Swart, E. C., J. R. Bracht, V. Magrini, P. Minx, X. Chen *et al.*, 2013 The *Oxytricha trifallax* macronuclear genome: a complex eukaryotic genome with 16,000 tiny chromosomes. PLoS Biol 11**:** e1001473.

Tarn, W. Y., and J. A. Steitz, 1996a Highly diverged U4 and U6 small nuclear RNAs required for splicing rare AT-AC introns. Science 273**:** 1824-1832.

Tarn, W. Y., and J. A. Steitz, 1996b A novel spliceosome containing U11, U12, and U5 snRNPs excises a minor class (AT-AC) intron in vitro. Cell 84**:** 801-811.

Turunen, J. J., E. H. Niemelä, B. Verma and M. J. Frilander, 2013 The significant other: splicing by the minor spliceosome. Wiley Interdiscip Rev RNA 4**:** 61-76.

UniProtKB, 2021 UniProt: the universal protein knowledgebase in 2021. Nucleic Acids Res 49**:** D480-d489.

Yan, C., J. Hang, R. Wan, M. Huang, C. C. Wong *et al.*, 2015 Structure of a yeast spliceosome at 3.6-angstrom resolution. Science 349**:** 1182-1191.

Zhang, X., C. Yan, J. Hang, L. I. Finci, J. Lei *et al.*, 2017 An atomic structure of the human spliceosome. Cell 169**:** 918-929.e914.

Zhao, Y., W. Dunker, Y. T. Yu and J. Karijolich, 2018 The role of noncoding RNA pseudouridylation in nuclear gene expression events. Front Bioeng Biotechnol 6**:** 8.

5. Untranslated regions of nuclear genes

INTRODUCTION

The term UnTranslated Region (UTR) refers to the sequence region upstream of the start codon (5′ UTR) and downstream of the stop codon (3′ UTR) of a protein-coding gene; these regions are part of the mature transcript. The 5′ UTR and the 3′ UTR include signals for translation initiation and termination, respectively, and harbor binding sites for small RNAs and proteins that regulate mRNA localization, stability and translation. One of the recently discovered roles of UTRs is the regulation of protein features that are not specified in the amino acid sequence (HINNEBUSCH *et al.* 2016; MAYR 2019; RENZ *et al.* 2020).

In contrast to most eukaryotes that primarily control nuclear gene expression *via* transcription initiation, kinetoplastids —and likely all euglenozoans— transcribe genes in bulk (i.e., polycistronically) and regulate the expression of individual genes post-transcriptionally (reviewed in (CLAYTON 2019)). In these organisms, the control of mRNA stability seems essential. Translation and decay rates of an mRNA are first and foremost influenced by its 3′ UTR, which serves as a landing platform for a wide variety of RNA-binding proteins (RBPs) (KOLEV *et al.* 2014). Messenger RNAs of kinetoplastids have been known to carry 3′ UTRs of similar extended length as those from vertebrates and much longer than reported for other eukaryotes (KOLEV *et al.* 2014; DILLON *et al.* 2015).

RESULTS AND DISCUSSION

To explore the potential of nuclear mRNAs from *Diplonema* to interact with regulatory RNA-binding proteins, we analyzed the length of UTRs and compared them across several eukaryotic species. *Diplonema* stood out in two aspects. While 5′ UTRs are quite short (~70 bp median length) as seen in most eukaryotes, 3′ UTRs are remarkably long, with a median size of ~860 bp. Second, for most organisms, the 3′ UTR tends to be approximately twice as long as the 5′ UTR, but in *Diplonema*, the two median values differ by a factor of ~13 (**Supplementary Table S15, Supplementary Figure S15**). The corresponding analyses were also performed with the subset of about 300 expert-curated gene models. In the case of 5′ UTRs, the values for the curated and non-curated gene set differed only marginally (65 bp vs 70 bp), whereas the automatically predicted 3′ UTRs were about 30% too short (~720 bp vs 970 bp).

In euglenozoans, including diplonemids, 5′ UTRs of genes can be easily inferred *via* detection of spliced-leader acceptor (SLTS) sites. Still, when 3′ UTRs are long, the correct determination of their 3′ terminus is challenging unless RNA-Seq reads in the kbp-size range are available. In kinetoplastids, for example, 3′ UTR sizes have been underestimated by automated annotations because of the difficulty in mapping short transcriptomic reads to repetitive and low-complexity regions that occur in 3′ UTRs (CLAYTON 2019). Because of their importance for gene regulation, mapping the 3′ UTRs will be critical for future functional studies in all euglenozoans.

It is possible that what appears to be 3′ UTRs may contain unrecognized coding sequences. For the three longest contigs (see also the **Supplementary Information**: Section 2. Assembly and annotation of the nuclear genome and transcriptome of *Diplonema papillatum*, in particular the section on the expert validation of gene models), we screened 3′ UTRs for additional ORFs >100 codons long. A vast majority consist of very short ORFs that are probably spurious because no homologs were detected in other organisms, including diplonemids (not considering fragments of mobile-element ORFs). While we currently cannot rule out that genuine ORFs occur in long 3′ UTRs, this is probably not a frequent phenomenon in *Diplonema*. Alternatively, the short ORFs might serve a regulatory function, similar to short (mostly upstream) ORFs in fungi or plants (HELLENS *et al.* 2016; SCHLESINGER AND ELSÄSSER 2022). Resolving this issue will require the future application of experimental techniques such as ribosome profiling and deep proteomics.

Alternative processing events add another layer of complexity to the picture. For example, in *Leishmania*, the predominantly used SLTS and poly-adenylation (PA) sites are generally closer to CDS boundaries than the alternative sites, which seem to play important roles in the cell (DILLON *et al.* 2015). In *D. papillatum* we detected alternative SLTS sites in ~10% of genes. In addition, the observed variation in RNA-Seq coverage at the 3′ end of certain *Diplonema* genes may reflect alternative transcript processing events, indicating that gene regulation in this organism is at least as complex as in trypanosomes.

Supplementary Table S15. **Length of UTRs in nuclear genes from *D. papillatum* and other eukaryotes**.

Organism	Lineage	5'UTR median length [bp] [a]	3'UTR median length [bp] [b]	Gene number [c]	Source [d]
Aspergillus nidulans	Fungi; Ascomycota	116	208	6,037	FungiDB
Saccharomyces cerevisiae	Fungi; Ascomycota	49	116	2,849	SGD
Schizosaccharomyces pombe	Fungi; Ascomycota	177	267	4,524	FungiDB
Cryptococcus neoformans	Fungi; Basidiomycota	93	143	6,181	FungiDB
Allomyces macrogynus	Fungi; Blastocladiomycota	145	133	9,088	FungiDB
Rhizophagus irregularis	Fungi; Glomeromycota	100	157	11,216	FungiDB
Spizellomyces punctatus	Fungi; Chytridiomycota	103	159	6,331	FungiDB
Homo sapiens	Metazoa; Chordata	225	593	21,838	UTRDB; (GRILLO *et al.* 2010)
Drosophila melanogaster	Metazoa; Arthropoda	140	214	16,671	FlyBase
Phytophthora parasitica	SAR; Oomycota	88	160	6,986	FungiDB
Saprolegnia diclina	SAR; Oomycota	42	74	9,053	FungiDB
Toxoplasma gondi [e]	SAR; Apicomplexa	680	675	5,194	ToxoDB; (HASSAN *et al.* 2012)
Vitrella brassicaformis	SAR; Chromerida	90	253	16,306	CryptoDB
Oxytricha trifallax	SAR; Ciliophora	34	78	17,040	(SWART *et al.* 2013)
Leishmania major	Discoba; Euglenozoa	233	517	8,841	(DILLON *et al.* 2015)
Trypanosoma brucei	Discoba; Euglenozoa	102	389	5,474	TriTrypDB
Diplonema papillatum [f]	Discoba; Euglenozoa	66	857	15,369	this work

[a] Only 5' UTRs >10 bp were considered.
[b] Only 3' UTRs >20 bp were considered.
[c] The number of genes used for the analysis, selected by the criteria that both UTRs were annotated and had a 5' and 3' UTR length of ≥10 bp and ≥20 bp, respectively.
[d] References: FungiDB, https://fungidb.org/; SGD, http://www.yeastgenome.org ; UTRDB, http://utrdb.ba.itb.cnr.it/home/download ; FlyBase, https://flybase.org/ ; ToxoDB, https://toxodb.org/ ; CryptoDB, https://cryptodb.org/ ; TriTrypDB, https://tritrypdb.org/.
[e] *Toxoplasma gondi* is exceptional for having very long 5' and 3' UTRs with nearly identical median-length values.
[f] The numbers for *D. papillatum* refer to uncurated genes.

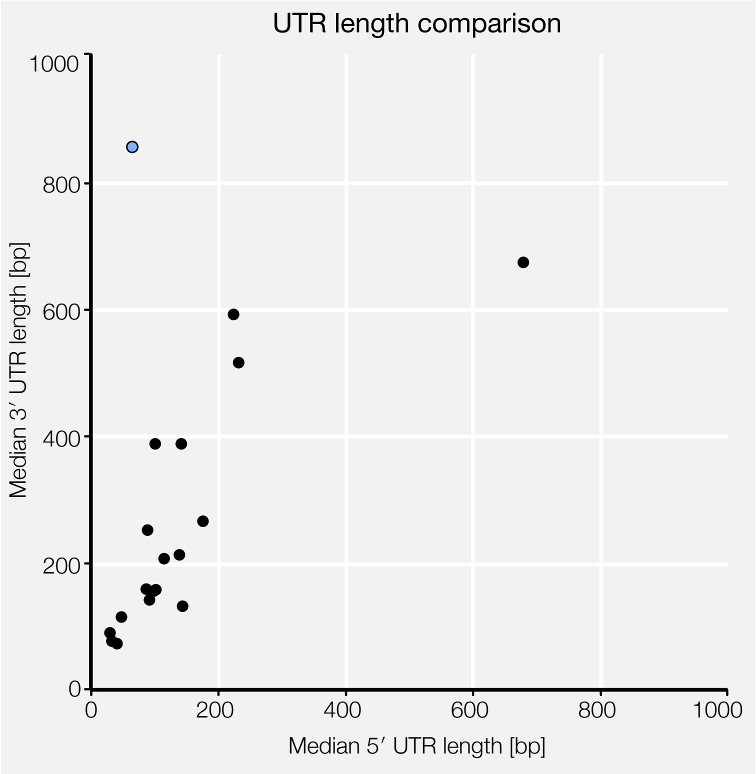

Supplementary Figure S15. Length of UTRs in nuclear genes from *D. papillatum* and other eukaryotes. The plot compares the length of 5′ and 3′ UTRs; *Diplonema* is highlighted in blue. For details, see (**Supplementary Table S15**).

MATERIALS AND METHODS

Data on untranslated regions (UTRs) of protein-coding genes were collected from several databases and publications (see **Supplementary Table S15**). Several filtering steps were implemented to ensure consistency across datasets. First, we considered only 5′ and 3′ UTRs of ≥10 bp and ≥20 bp length, respectively. Second, both 5′ and 3′ UTR had to be mapped for a gene to be considered. To avoid duplication, we focused on primary (predominantly used) mRNA processing sites; 100% identical UTR sequences were removed from *Saccharomyces cerevisiae* and *Homo sapiens* data, but otherwise, for practical reasons, alternative UTRs were kept. Lastly, we included only species for which the protein-coding genes that passed all filtering stages represented at least 50% of all genes in the corresponding nuclear genome. In addition to *Diplonema*, the final dataset contained 15 species representing the Fungi, Metazoa, SAR and Discoba lineages. We then calculated the median 5′ and 3′ UTR lengths for comparative purposes.

AUTHOR CONTRIBUTIONS

Conceptualization, Data curation, Investigation, Formal analysis, Visualization, Writing, original draft – M.V.; **Writing, review & editing –** all co-authors.

REFERENCES

Clayton, C., 2019 Regulation of gene expression in trypanosomatids: living with polycistronic transcription. Open Biol 9**:** 190072.

Dillon, L. A., K. Okrah, V. K. Hughitt, R. Suresh, Y. Li *et al.*, 2015 Transcriptomic profiling of gene expression and RNA processing during *Leishmania major* differentiation. Nucleic Acids Res 43**:** 6799-6813.

Grillo, G., A. Turi, F. Licciulli, F. Mignone, S. Liuni *et al.*, 2010 UTRdb and UTRsite (RELEASE 2010): a collection of sequences and regulatory motifs of the untranslated regions of eukaryotic mRNAs. Nucleic Acids Res 38**:** D75-80.

Hassan, M. A., M. B. Melo, B. Haas, K. D. Jensen and J. P. Saeij, 2012 *De novo* reconstruction of the *Toxoplasma gondi*i transcriptome improves on the current genome annotation and reveals alternatively spliced transcripts and putative long non-coding RNAs. BMC Genomics 13**:** 696.

Hellens, R. P., C. M. Brown, M. A. W. Chisnall, P. M. Waterhouse and R. C. Macknight, 2016 The emerging world of small ORFs. Trends Plant Sci 21**:** 317-328.
Hinnebusch, A. G., I. P. Ivanov and N. Sonenberg, 2016 Translational control by 5'-untranslated regions of eukaryotic mRNAs. Science 352**:** 1413-1416.
Kolev, N. G., E. Ullu and C. Tschudi, 2014 The emerging role of RNA-binding proteins in the life cycle of *Trypanosoma brucei*. Cell Microbiol 16**:** 482-489.
Mayr, C., 2019 What are 3' UTRs doing? Cold Spring Harb Perspect Biol 11.
Renz, P. F., F. Valdivia-Francia and A. Sendoel, 2020 Some like it translated: small ORFs in the 5′UTR. Experimental Cell Research 396**:** 112229.
Schlesinger, D., and S. J. Elsässer, 2022 Revisiting sORFs: overcoming challenges to identify and characterize functional microproteins. Febs j 289**:** 53-74.
Swart, E. C., J. R. Bracht, V. Magrini, P. Minx, X. Chen *et al.*, 2013 The *Oxytricha trifallax* macronuclear genome: a complex eukaryotic genome with 16,000 tiny chromosomes. PLoS Biol 11**:** e1001473.

6. Repetitive sequences in the nuclear genome of *Diplonema papillatum* (assembly v_1.0)

INTRODUCTION

Nuclear DNA of almost all eukaryotes contains a considerable portion of **intergenic regions**, from 70% in *Arabidopsis thaliana* (Arabidopsis Genome AGI 2000) to 30% in *Saccharomyces cerevisiae* (WOOD *et al.* 2001) and *Trypanosoma brucei* (ASLETT *et al.* 2010). A major portion of intergenic regions consists of sequence motifs that occur multiple times throughout the genome.

Repetitive DNA is commonly subdivided into two major families, tandem repeats and dispersed repeats (RICHARD *et al.* 2008). **Tandem repeats** are defined as a sequence motif of two or more nucleotides repeated multiple times adjacently head to tail. In more general terms, tandem repeats are a subclass of low-complexity regions (LCRs), since the latter category is defined as arrays of sequence patterns with biased composition (GGT-GTT-TTG; or GGT-GGT-GGT) and thus comprises arrays of identical repeat units. Long tandem repeats can contain multiple copies of a protein-coding gene or of the rDNA cluster consisting of the small subunit (SSU) rRNA, 5.8S rRNA, and large subunit (LSU) rRNA. Tandem repeats not including genes are often referred to as Satellite DNA.

Satellite DNA is subdivided into three subclasses according to the length of the repeat unit (note, however, that there is no strict convention as to size ranges): unit sizes of 2-10 bp define **microsatellites**, 11 to 99 bp-long units are categorized as **minisatellites**, and arrays of 100 bp and beyond as **macrosatellites**. Microsatellites, also denoted 'short tandem repeats' (STRs), include the much-studied trinucleotide repeats occurring within protein-coding regions, since retractions and expansions of such arrays have been linked to human disease (RAMAKRISHNAN AND GUPTA 2021). Minisatellites are typically located in intergenic and subtelomeric regions, whereas macrosatellites are prevalent in centromeric regions of nuclear chromosomes (MELTERS *et al.* 2013).

The second family of repetitive DNA encompasses the **dispersed** (or interspersed) **repeats**, i.e., sequence-motif copies that occur in different locations of the genome. Many of the dispersed repeat units have no apparent origin or function, and others include tRNAs, rDNA, gene copies, and transposons. The notion of 'junk DNA' or 'selfish elements' alludes to repetitive sequences being vestiges of self-replicating and mobile elements (OHNO 1972; ORGEL *et al.* 1980). However, in the meantime, certain genomic repeats have been shown to play a role in genetic variation and gene regulation, and they have been suggested to shape the three-dimensional folding of nuclear DNA (MEHROTRA AND GOYAL 2014). Locating and characterizing tandem repeats by bioinformatics methods remains a challenge because the various unit copies may contain substitutions and indels, and because of complex, nested structures of repeat arrays. As imperfect, degenerated repeat units are modelled differently by the various repeat-detection algorithms (e.g., RepeatMasker (SMIT AND GREEN), RepeatScout (PRICE *et al.* 2005) and Tandem Repeats Finder (BENSON 1999)), it is not surprising that the results for the same sequence but analyzed by different software often differ.

RESULTS AND DISCUSSION

Several publications have used RepeatMasker (SMIT AND GREEN) to analyze repeats in a genome, since it returns information about low-complexity regions, simple repeats, and satellites in a readily readable form. However, the RepeatMasker documentation explicitly states that the program is not suited for this task; its purpose is masking repeat regions in the genome prior to structural annotation to avoid spurious matches in database searches. To get an appreciation of the repeat content in the *D. papillatum* nuclear genome, we employed dedicated tools to identify tandem and interspersed repeats.

With less than 6%, **tandem repeats** of unit length 2 to 50 bp cover only a small portion of the *Diplonema* nuclear genome (**Supplementary Table S16**). The longest tandem array consists of 409 copies of a 48-bp unit that extends to nearly 20 kbp (tig00023097_5, positions 169,920-189,538) and is located in an intergenic region without any evidence for transcription. Homopolymers (1-bp units) of either A, C, G, or T were also found. Mononucleotide tracts longer than 600 bp turned out to be artifacts introduced by single PacBio reads. Still, shorter tracts have reliable long-read support, such as the 169-bp long T-run in contig tig00023828_2, positions 11,191-11,359.

Dispersed repeats represent the predominant type in the *D. papillatum* nuclear genome and constitute as much as 46% of the assembly. The number of distinct dispersed repeats detected by the RepeatScout tool is nearly 10,000 with a length of up to nearly 20 kbp long (e.g., R=44: 19,085 bp). The unit with the largest number of copies (≥90% identity) is R=0, a 226-bp long motif that occurs 6,030 times in the genome.

Tandem repeats rarely contain coding regions of validated protein-coding genes, but instead include parts or complete spurious gene models that lack support by RNA-Seq data. This contrasts with structural RNAs, which are often

arranged tandemly. In addition, tandem arrays can be part of dispersed repeats that occur in multiple copies across the genome. For example, half of the dispersed repeat R=121 (15,715 bp) is made up of the rDNA repeat unit that is composed of the genes for small subunit (SSU or 18S) rRNA, 5.8S rRNA, and large subunit (LSU or 28S) rRNA; (the genes are referred to as *rnl*, *rns*, *rrn5*, and *rn5.8*). R=121 is found nine times in the genome assembly, when considering copies of ≥90% identity. Similarly, the dispersed repeats R=75 (18,233 bp) and R=163 (7,201 bp), which occur in the assembly 4 and 7 times (at ≤90% identity), respectively, include tandem repeats of alternating 5S rRNA, spliced-leader RNA and spliceosomal U2 RNA-genes. Structural RNA genes identified in the *D. papillatum* nuclear genome assembly and their arrangement in repeat arrays are described in more detail in the **Supplementary Information**: Section 4. Intron splicing and structural RNAs.

About 2% of dispersed repeat motifs include small **pieces** of **rRNA** genes together with unrelated sequences of unknown identity. Among the four rRNA genes, fragments of *rnl* are most abundant, followed by *rns*, with pieces of 30-200 bp occurring ~2,400 and 1,100 times in the genome sequence, respectively.

Since **introns** in *Diplonema* nuclear genes can be several dozen kbp long, we investigated whether they include long dispersed repeats. One of the longest intronic regions covered by dispersed repeats is included in the gene DIPPA_27122 encoding a homolog of cholesterol-7-desaturase. The longest repeat unit that falls in the 9,167-bp long intron 1 is R=510 (7,674 bp), covering 84% of the intron 1. When considering copies of ≥90% sequence identity, R=510 occurs in 21 other positions in the *Diplonema* genome, totalling to six located in introns and 15 in untranscribed, intergenic regions. Introns of similar sequence but located in distinct genes are suggestive of containing **mobile genetic elements**. Conceptual translation indeed revealed a 1,866 amino acid-long reading frame (ORF; included in R=510 and named orf_R_510) carrying three protein domains diagnostic for mobile elements: an Exo_endo_phos domain in the N-terminal third of the sequence, an RT_like superfamily domain in the middle, and an RNase_H_like domain in the C-terminal third of the protein. A Pfam domain search retrieved RVT_1, RVT_3, Exo_endo_phos, and RNase_H. The search for free-standing sequences (i.e., outside introns) in the inferred *D. papillatum* proteome that are similar to orf_R_510 retrieved proteins from 1,008 distinct gene models. The corresponding function annotations indicated either ‘Retrovirus-related Pol polyprotein’, ‘Ribonuclease HI’, ‘RNA-directed DNA polymerase from mobile element’, or ‘LINE-1 reverse transcriptase homolog’. A search for nucleotide sequences in the *D. papillatum* nuclear genome that can be conceptually translated into orf_R_510-like proteins retrieved another 500 matches, of which 21 displayed 100% protein-sequence identity.

The above result is consistent with the extraordinary expansion of transposable element (TE)-ORFs detected in the analysis of gene family evolution in *D. papillatum*, indicating that the *Diplonema* nuclear genome is permeated with ORFs typically encoded by transposable elements. Indeed, the analysis of the function assignments of the inferred proteins revealed signatures of both transposon classes—retrotransposons and DNA transposons— and a total of 12 subclasses. Retrotransposon ORFs make up the majority of subclasses (75%) and the predominant portion of instances (95%; **Supplementary Table S17**). Retrospectively, the observed repetitiveness and complex repeat arrangement of the *D. papillatum* nuclear genome explains why the sequence assembly engines did not generate longer and fewer contigs. In turn, the fragmentation of the current assembly represents a limitation in the rigorous characterization of repeats.

Supplementary Table S16. Repeat analysis[a]**.**

Tool	Interspersed classified repeats	Dispersed unclassified repeats	Satellite DNA/ Tandem repeats
TandemRepeatsFinder	/	/	Count: 100,426<br>LenRange: 2-50 bp<br>CumulLen: 17,042,328 bp **(6%)**<br>ArrayLenRange: 25-29,859 bp
RepeatMasker	Count: 2,412[b]<br>CumulLen=264,094 bp	/	Count: 93,490<br>LenRange: 1-6 bp [c]<br>CumulLen: 5,388,838 bp [d]
RepeatScout (repeat unit detection) & glsearch (mapping of units to genome)	/	Count: 9,783<br>LenRange: 51 - 19,085 bp<br>MedLen=236 bp<br>CumulLen: 128,855,866 bp **(46%)**	/

[a] **Count**: number of motif instances; **LenRange**: length range of repeat motifs (units); **MedLen**=median length of repeat units; **CumulLen**, total (cumulative) length of all motif instances, after merging; **%** of total genome (size 280,385,187 bp); **ArrayLenRange**: length range of tandem arrays.
[b] RepeatMasker-reported types of retrotransposons and mobile DNA elements known from human: 1,577 LINEs, 321 SINEs, 22 LTRs; 492 DNA elements. A compilation of Euglenozoan transposable elements is not available.
[c] RepeatMasker uses a fixed unit length of 6 bp, referred to as simple repeats.
[d] These numbers are considerably smaller than NSEG and TandemRepeatsFinder’s results because RepeatMasker has not been designed to detect repeats or low-complexity DNA regions comprehensibly.

Supplementary Table S17. Similarity of inferred *D. papillatum* proteins to ORFs of transposable elements

Transposon class	Element designation	Count	Explicit function annotation
Retrotransposons (Class I transposons)	Copia-like	2	product=Retrovirus-related Pol polyprotein from transposon 17.6
	BS	115	product=Probable RNA-directed DNA polymerase from transposon BS; note=Reverse transcriptase (RNA-dependent DNA polymerase); product=Probable RNA-directed DNA polymerase from transposon BS; note=Endonuclease/Exonuclease/phosphatase family; product=Probable RNA-directed DNA polymerase from transposon BS; note=Astacin (Peptidase family M12A);
	LINE	245	product=LINE-1 reverse transcriptase homolog; product=LINE-1 retrotransposable element ORF2 protein;
	Opus	1	product=Retrovirus-related Pol polyprotein from transposon opus
	R1	1	product=Retrovirus-related Pol polyprotein from type-1 retrotransposable element R1;
	R2	304	product=Retrovirus-related Pol polyprotein from type-1 retrotransposable element R2;
	R2DM	212	product=Retrovirus-related Pol polyprotein from type-2 retrotransposable element R2DM;
	X	266	product=Probable RNA-directed DNA polymerase from transposon X-element; note=Reverse transcriptase (RNA-dependent DNA polymerase)
	SLACS	66	product=Retrotransposable element SLACS 132 kDa protein; product=SLACS reverse transcriptase, putative [Trypanosoma equiperdum]; product=SLACS reverse transcriptase, putative [Trypanosoma theileri];
All retrotransposons		1,212	
DNA transposons[b] (Class II transposons)	MULE	46	product=hypothetical protein; note= MULE transposase domain; product=hypothetical protein; note=Transposase, Mutator family;
	TATE	12	product=TATE DNA Transposon [Trypanosoma theileri]; note=Phage integrase family;
	Tc5	1	product=hypothetical protein; note=Tc5 transposase DNA-binding domain;
All DNA transposons		69	

[b] The nine *D. papillatum* proteins carrying the annotation "product=hypothetical protein; note=containing Helitron helicase-like domains" are not listed here, because the relationship of these proteins to Helitron transposons is uncertain.

NUMTs in the *D. papillatum* nuclear genome

A particular subtype of dispersed repeats in the nuclear genome of *D. papillatum* is NUclear MiTochondrial segments (NUMTs). These regions originate from intracellular transfer of the mitochondrial DNA (mtDNA) to the nucleus. NUMTs have been reported in almost all eukaryotic genomes studied so far, but the number and sizes of transferred mtDNA segments vary considerably (KLEINE *et al.* 2009). It is important to distinguish NUMTs from originally mitochondrion-encoded genes that have migrated to the nucleus, a process that took place during the transformation of the endosymbiotic alpha-proteobacterium to an integral organelle of the eukaryotic cell. These relocated genes code for mitochondrial components, implying that the corresponding gene products are targeted to organelles. NUMTs in contrast, are considered not to be expressed ((HAZKANI-COVO *et al.* 2010) but see (NOUTSOS *et al.* 2007; ROGERS AND GRIFFITHS-JONES 2012; WANG *et al.* 2014)). Here we examined to what degree the incorporation of mtDNA has contributed to the large size of the *Diplonema* nuclear genome.

Our search for NUMTs in the *D. papillatum* nuclear genome took into consideration the unusual **mitochondrial genome architecture and gene structure** in this organism, which appears to be a shared feature of diplonemids (KIETHEGA *et al.* 2011; VALACH *et al.* 2017; KAUR *et al.* 2020). Mitochondrial DNA in *Diplonema* is organized in about 80 distinct circular chromosomes of 7-8 kbp length. Only 40-550 bp of these circles is coding, constituting fragments of genes, also called 'modules'. Gene modules are flanked by short stretches of unique sequence ('flanking sequence'). In contrast, the remaining 90% of a mitochondrial chromosome, referred to as 'constant region', is common to all chromosomes in the *Diplonema* mitochondrial genome (KIETHEGA *et al.* 2013; MOREIRA *et al.* 2016; VALACH *et al.* 2016). Constant regions are characterized by tandem repeats and low sequence complexity.

Approximately **1.2%** of the ***D. papillatum* nuclear genome** assembly v1.0 consists of NUMTs. This percentage is likely an underestimation because NUMTs are known to accumulate mutations leading to divergent sequences that are difficult to spot. Therefore, only the relatively recently transferred NUMTs will be recognized. The NUMTs that we detected in the *Diplonema* nuclear genome fall into two categories (i) "anchored" NUMTs representing high-confidence matches including mitochondrial gene modules; and (ii) constant-region NUMTs (cr-NUMTs) that exclusively contain portions of the constant region of mitochondrial chromosomes.

We retrieved 60 **anchored NUMTs** with a cumulative length of ~82.8 kbp. These NUMTs contain sequences from 25 distinct modules. As many as 11 NUMTs represent entire mitochondrial chromosomes; the accuracy of the nuclear genome assembly in these regions was confirmed by inspecting long PacBio reads. Additional 26 NUMTs consist of a part of a gene module, and another 23 also contain segments of flanking or constant regions. NUMTs comprising partial mitochondrial chromosomes add up to ~9.7 kbp (**Additional File 2** numts). Most anchored NUMTs are located in intergenic regions of nuclear contigs or in an intron of a nuclear gene; intronic location also applied to two of the NUMTs consisting of full-length mitochondrial chromosomes. Six anchored NUMTs reside within UTRs of nuclear genes and are transcribed together with the rest of the host genes. Interestingly, about one third of anchored NUMTs are arranged in tandem repeats. The longest array, residing in contig tig00023746_1, is 485 bp long and consists of eight copies of a ~40-bp long portion of module X18, which is an unidentified mitochondrial gene or gene fragment (MOREIRA *et al.* 2016). A somewhat shorter array (379 bp) occurs in tig00023753 and contains four copies of a 95-bp piece of module #1 of the mitochondrial LSU RNA (*rnl*-m1).

Searches for **cr-NUMTs** retrieved 1,359 instances with a median size of 128 bp and totaling nearly 260 kbp. Certain cr-NUMTs are also arranged in tandem. The longest array consists of 164 copies of a 68-bp motif and makes up an 11-kbp region within an intron of the gene DIPPA_33001. Nevertheless, most cr-NUMTs are dispersed individually across the nuclear genome, just as their anchored counterparts. They occur predominantly in intergenic regions (1,097 instances) and non-coding segments of genes (168 in introns, 95 in UTRs). Only five cr-NUMTs overlap by up to 120 bp a protein-coding region, usually at a CDS terminus (**Additional File 2** numts).

The total length and proportion of the *Diplonema* nuclear DNA whose origin can be traced to mtDNA is within the range published for **other organisms**, especially animals and plants (MICHALOVOVA *et al.* 2013; KO AND KIM 2016; CALABRESE *et al.* 2017). However, it should be noted that the reported contribution of NUMTs to nuclear genomes is generally an approximation because the underlying assemblies, unless built from long reads, are probably not entirely correct. Moreover, even within a given species, NUMTs might vary across ecotypes, as was recently documented in *A. thaliana*. The commonly studied ecotype Columbia-0 contains an enormous NUMT corresponding to the entire mitochondrial genome, which was confirmed by fluorescence *in situ* hybridization (FISH) on metaphase chromosomes (STUPAR *et al.* 2001). In contrast, recent nuclear genome sequencing of the two ecotypes Niederzenz-1 and Landsberg by a long-read technology revealed the absence of these insertions (PUCKER *et al.* 2019).

METHODS

Repeat identification

RepeatMasker v.4.1.1. (http://www.repeatmasker.org/) was executed in sensitive mode, run with rmblastn v.2.9.0+. TandemRepeatsFinder v.4.09 (BENSON 1999) was used with the command line parameters 2 7 7 80 10 50 2000 -h -m –ngs, i.e. with a maximum unit length of 2,000 bp. The corresponding output was filtered for further analyses to retain the repeat units of up to 50 bp. Low-complexity repeats including satellites with units <500 bp were identified with the tool nseg v1.0.0 (Wooton and Federhen, 1993), but only used for comparison with the output of TandemRepeatsFinder, without further following up the nseg results. *De novo* detection of dispersed repeats was performed with RepeatScout v.1.0.5 (PRICE *et al.* 2005). The first step is the generation of an kmer (lmer) table

```
$ build_lmer_table -s sequence Dp_v1.0.fasta -freq lmer_table_v1.0.txt
```

that serves as input for RepeatScout

```
$ RepeatScout -sequence Dp_v1.0.fasta -output repS_v1.0.fasta -freq lmer_table_v1.0.txt
```

The output (`repS_v1.0.fasta`) is then filtered by the script filter-stage-1.prl that is provided with the RepeatScout distribution to remove from the initially determined repeat motifs those that consists of tandem repeats and low-complexity regions:

```
$ cat repS_v1.0.fasta | /share/supported/apps/RepeatScout-1.0.5/filter-stage-1.prl  > repS_v1.0-filter1.fasta
```

The output is a collection of distinct dispersed-repeat sequences but does not include their positions in the genome sequence. The authors of RepeatScout recommend mapping the repeat sequence to the genome by using RepeatMasker and providing the repS sequences as database. We generated a gff file that lists repS positions in the genome with the following command:

```
$ RepeatMasker -s -pa 38 -engine ncbi -dir .  -lib repS_v1.0-filter1.fasta  -gff -xsmall Dp_v1.0.fasta
```

However, the result was unsatisfactory because certain repeats were not identified in all expected positions. For finding repeat copies exhaustively, we used a global-query and local-database similarity search, notably glsearch of the fasta suite v.36.3.8g (PEARSON 2016). Glsearch allows defining the sequence identity threshold of repeat copies reported:

```
$ glsearch36 -T 40 –E 1.0e-5 -m8CB repS_v1.0-filter1.fasta  Dp_v1.0.fasta  >glsearch-out-repS_v1.0-filter1-vs-Dp_v1.0-e-5
```

The chosen generous E-value allows detection of copies of the shortest repS motifs (50 bp) at a minimum identity of ~60%. The output file was then filtered to extract motif copies of a given percentage of identity. We experimented with 75%, but

the subsequent analyses were performed with repeat copies of at least 90% identity. From the file `glsearch-out-repS_v1.0-filter1-vs-Dp_v1.0-0.9ident` we generated bed and gff files with in-house scripts, and calculated the number of occurrences (frequency) at which the repeat units occur in the genome sequence. To calculate the length and proportion of the genome covered by repeats, intervals covered by different repeats were merged with the BEDTools utility merge (BEDTools v.2-2.29.2 (QUINLAN 2014):

```
$ bedtools merge -i repS_v1.0-filter1-0.9ident.bed -c 4 -o distinct > repS_v1.0-filter1-0.9ident-merged.bed
```

To determine the overlap of repS copies with genes, exons, and introns, we generated from the conventional genome gff file (Dp_v1.0.gff3) one that lists only protein-coding genes and indicates introns explicitly. For that we used the GenomeTools system v.1.6.1 (http://genometools.org/tools.html) embedded in an awk script:

```
$ awk 'BEGIN {FS=OFS="\t"}s {split($9, a, "[;=]"); $9="gene_id \""a[4]"\"; transcript_id \""a[4]"\""}1'
Dp_v1.0.gff3 | gt gtf_to_gff3 -tidy 2>/dev/null | gt gff3 -sort -tidy -retainids -addintrons |grep -v '^#' >
out
```

Subsequently, we replaced the gene identifiers in the `out` file, which were introduced by the command `gt gtf_to_gff3`, with our gene identifiers DIPPA_<number>. The final gff file `Dp_v1.0-DIPPA+introns.gff3` contains explicit intron, exon, and gene features. This latter file was used to determine the overlaps of individual repeats with exons or introns employing BEDTools intersect:

```
$ bedtools intersect -wo -a repS_v1.0-filter1-0.9ident.bed -b Dp_v1.0-DIPPA+introns.gff3 > repS_v1.0-filter1-
0.9ident-intersect-genes
```

To determine the portions of genes, exons, or introns covered by repeats, the unmerged bed file was used as input file specified by –a. Finally, selected, long dispersed repeat motifs were conceptually translated with a home-made script and protein sequences >= 1,000 residues were retained. For example, the protein sequence file `orf_R=510.faa` represents the longest reading frame contained in the dispersed repeat R=510 detected by RepeatScout. Sequences in the inferred *Diplonema* proteome with similarity to `orf_R=510.faa` were detected with phmmer v.6.1 (EDDY 2011) using an E-value threshold of 1.0 e-50. Protein domains were retrieved from the output of BLAST searches (ALTSCHUL *et al.* 1990) in the non-redundant database at the National Center for Biotechnology Information (NCBI; https://www.ncbi.nlm.nih.gov/) and from a search in the protein-family database Pfam (http://pfam.xfam.org/; (EL-GEBALI *et al.* 2019)) using hmmscan via the HMM webserver v.2.41.2 employing HMM release 3.3.2; http://hmmer.org/ (EDDY 2011)) of EMBL-EBI (https://www.ebi.ac.uk/Tools/hmmer/search/hmmscan) (POTTER *et al.* 2018).

Ribosomal RNA genes and gene fragments included in the genome sequence were searched with BLAST v2.2.26 (ALTSCHUL *et al.* 1990).

The identification of **transposable-element** types described in the main text was performed by extracting distinctive keywords from the automated function annotation. More specifically, we parsed the 'product' field (populated via function transfer from matches with UniProt/SwissProt proteins) and the 'note' field (containing information derived from matches with unreviewed domains) of the gff3 file. The search terms included 'gag protein', 'ingi', 'LINE', 'L1Tc', 'mobile element', 'polyprotein', 'reverse transcriptase', 'retrotranspos', 'retrovirus', 'ribonuclease HI', 'TcC31', 'SLACS', 'transcriptase'. The retrieved annotations were human-inspected.

To validate the automated function annotation '(putative) **trans-sialidase'**, initially assigned to 330 genome-inferred proteins, we performed a local HMM search with phmmer against the collection of reviewed trans-sialidases in Swissprot and further by HMMscan against all PFAM protein domains using web service at https://www.ebi.ac.uk/Tools/hmmer/. We realized that the function assignment of ~95% 'trans-sialidase' proteins was erroneous due to annotation transfer from incorrectly annotated proteins in GenBank.

NUMT identification

The 82 mitochondrial gene modules identified previously (MOREIRA *et al.* 2016) were searched against the nuclear genome assembly v1.0 using the Discontiguous MegaBLAST tool of the BLAST+ v2.2.28 package (CAMACHO *et al.* 2009). The scoring scheme was optimized for ~90% identity searches (i.e., match|mismatch|open gap|extend gap = 2|–3|5|2) (STATES AND BOTSTEIN 1991), with an initial E-value threshold of 10e-5 and a size threshold of 34 bp (i.e., longer than the 3× search-word size of 11 bp). Matches with contigs lacking gene annotations were ignored as these contigs might originate from incomplete removal of mitochondrial contigs from the nuclear genome assembly. To extend the matches to mitochondrial gene modules, we extracted from the contig the sequence interval that spans 8 kbp upstream and downstream of the match, 8 kbp being the maximum length of a mitochondrial chromosome. This nuclear genomic interval was then used as a query sequence to search by BLAST against a comprehensive mitochondrial sequence database. The target database consisted of all complete mitochondrial chromosome sequences (three and two from A and B class, respectively), all 81 cassettes, and all 18 mature transcripts (KIETHEGA *et al.* 2013; MOREIRA *et al.* 2016; VALACH *et al.* 2018). To verify whether a nuclear genomic region that included a NUMT was correctly assembled, we examined whether multiple distinct

long PacBio reads covered the NUMT and connected it to unique and reliable nuclear sequences. PacBio reads were aligned with the nuclear genome sequence using Minimap2 v2.17 (LI 2018), and the resulting alignments were visually inspected. We considered only reads of a minimum length of 5 kbp; support by one or two such reads was counted as weak evidence, and support by five or more reads counted as strong evidence for the candidate being located in the nuclear genome. All other pairwise and multiple sequence alignments were performed using MAFFT v7.388 (KATOH AND STANDLEY 2013). For searching cr-NUMTs derived from constant regions of mitochondrial chromosomes, we used the corresponding sequences of the complete chromosomes A46 (encoding *nad7*-m6 and *nad9*-m3; GenBank Acc. nr. HQ288824) and B03 (*cox1*-m4; EU123537) as representatives of the A and B class, respectively. The same parameters of BLAST+ and filtering were used as for the module-anchored searches described above. Overlapping hits were merged. For example, when A- and B-class mitochondrial chromosomes share a highly similar sequence, they will match a similar but not necessarily identical nuclear region; in such cases, the entire matched region was considered as a single NUMT.

AUTHOR CONTRIBUTIONS

Conceptualization, Data curation, Formal analysis, Investigation, Writing, original draft – G.B., M.V.; **Writing, review & editing –** all co-authors.

REFERENCES

AGI, 2000 Analysis of the genome sequence of the flowering plant *Arabidopsis thaliana*. Nature 408**:** 796-815.

Altschul, S. F., W. Gish, W. Miller, E. W. Myers and D. J. Lipman, 1990 Basic local alignment search tool. J Mol Biol 215**:** 403-410.

Aslett, M., C. Aurrecoechea, M. Berriman, J. Brestelli, B. P. Brunk *et al.*, 2010 TriTrypDB: a functional genomic resource for the Trypanosomatidae. Nucleic Acids Res 38**:** D457-462.

Benson, G., 1999 Tandem repeats finder: a program to analyze DNA sequences. Nucleic Acids Res 27**:** 573-580.

Calabrese, F. M., D. L. Balacco, R. Preste, M. A. Diroma, R. Forino *et al.*, 2017 NumtS colonization in mammalian genomes. Sci Rep 7**:** 16357.

Camacho, C., G. Coulouris, V. Avagyan, N. Ma, J. Papadopoulos *et al.*, 2009 BLAST+: architecture and applications. BMC Bioinformatics 10**:** 421.

Eddy, S. R., 2011 Accelerated profile HMM searches. PLoS Comput Biol 7**:** e1002195.

El-Gebali, S., J. Mistry, A. Bateman, S. R. Eddy, A. Luciani *et al.*, 2019 The Pfam protein families database in 2019. Nucleic Acids Res 47**:** D427-d432.

Hazkani-Covo, E., R. M. Zeller and W. Martin, 2010 Molecular poltergeists: mitochondrial DNA copies (numts) in sequenced nuclear genomes. PLoS Genet 6**:** e1000834.

Katoh, K., and D. M. Standley, 2013 MAFFT multiple sequence alignment software version 7: improvements in performance and usability. Mol Biol Evol 30**:** 772-780.

Kaur, B., K. Záhonová, M. Valach, D. Faktorová, G. Prokopchuk *et al.*, 2020 Gene fragmentation and RNA editing without borders: eccentric mitochondrial genomes of diplonemids. Nucleic Acids Res 48**:** 2694-2708.

Kiethega, G. N., M. Turcotte and G. Burger, 2011 Evolutionary conserved *cox1* trans-splicing without cis-motifs. Mol Biol Evol 28**:** 2425-2458.

Kiethega, G. N., Y. Yan, M. Turcotte and G. Burger, 2013 RNA-level unscrambling of fragmented genes in *Diplonema* mitochondria. RNA Biology 10**:** 301-313.

Kleine, T., U. G. Maier and D. Leister, 2009 DNA transfer from organelles to the nucleus: the idiosyncratic genetics of endosymbiosis. Annu Rev Plant Biol 60**:** 115-138.

Ko, Y. J., and S. Kim, 2016 Analysis of nuclear mitochondrial DNA segments of nine plant species: size, distribution, and insertion loci. Genomics Inform 14**:** 90-95.

Li, H., 2018 Minimap2: pairwise alignment for nucleotide sequences. Bioinformatics 34**:** 3094-3100.

Mehrotra, S., and V. Goyal, 2014 Repetitive sequences in plant nuclear DNA: types, distribution, evolution and function. Genomics Proteomics Bioinformatics 12**:** 164-171.

Melters, D. P., K. R. Bradnam, H. A. Young, N. Telis, M. R. May *et al.*, 2013 Comparative analysis of tandem repeats from hundreds of species reveals unique insights into centromere evolution. Genome Biology 14**:** R10.

Michalovova, M., B. Vyskot and E. Kejnovsky, 2013 Analysis of plastid and mitochondrial DNA insertions in the nucleus (NUPTs and NUMTs) of six plant species: size, relative age and chromosomal localization. Heredity (Edinb) 111**:** 314-320.

Moreira, S., M. Valach, M. Aoulad-Aissa, C. Otto and G. Burger, 2016 Novel modes of RNA editing in mitochondria. Nucleic Acids Res 44: 4907-4919.
Noutsos, C., T. Kleine, U. Armbruster, G. DalCorso and D. Leister, 2007 Nuclear insertions of organellar DNA can create novel patches of functional exon sequences. Trends Genet 23: 597-601.
Ohno, S., 1972 So much "junk" DNA in our genome. Brookhaven Symp Biol 23: 366-370.
Orgel, L. E., F. H. Crick and C. Sapienza, 1980 Selfish DNA. Nature 288: 645-646.
Pearson, W. R., 2016 Finding protein and nucleotide similarities with FASTA. Curr Protoc Bioinformatics 53: 3.9.1-3.9.25.
Potter, S. C., A. Luciani, S. R. Eddy, Y. Park, R. Lopez *et al.*, 2018 HMMER web server: 2018 update. Nucleic Acids Res 46: W200-w204.
Price, A. L., N. C. Jones and P. A. Pevzner, 2005 De novo identification of repeat families in large genomes. Bioinformatics 21 Suppl 1: i351-358.
Pucker, B., D. Holtgräwe, K. B. Stadermann, K. Frey, B. Huettel *et al.*, 2019 A chromosome-level sequence assembly reveals the structure of the *Arabidopsis thaliana* Nd-1 genome and its gene set. PLoS One 14: e0216233.
Quinlan, A. R., 2014 BEDTools: the swiss-army tool for genome feature analysis. Curr Protoc Bioinformatics 47: 11.12.11-34.
Ramakrishnan, S., and V. Gupta, 2021 Trinucleotide repeat disorders in *StatPearls*. StatPearls Publishing Copyright © 2021, StatPearls Publishing LLC., Treasure Island (FL).
Richard, G. F., A. Kerrest and B. Dujon, 2008 Comparative genomics and molecular dynamics of DNA repeats in eukaryotes. Microbiol Mol Biol Rev 72: 686-727.
Rogers, H. H., and S. Griffiths-Jones, 2012 Mitochondrial pseudogenes in the nuclear genomes of *Drosophila*. PLoS One 7: e32593.
Smit, A. F. A., and P. Green, RepeatMasker, pp.
States, D. J., and D. Botstein, 1991 Molecular sequence accuracy and the analysis of protein coding regions. Proc Natl Acad Sci U S A 88: 5518-5522.
Stupar, R. M., J. W. Lilly, C. D. Town, Z. Cheng, S. Kaul *et al.*, 2001 Complex mtDNA constitutes an approximate 620-kb insertion on *Arabidopsis thaliana* chromosome 2: implication of potential sequencing errors caused by large-unit repeats. Proc Natl Acad Sci U S A 98: 5099-5103.
Valach, M., A. Léveillé-Kunst, M. W. Gray and G. Burger, 2018 Respiratory chain Complex I of unparalleled divergence in diplonemids. J Biol Chem 293: 16043-16056.
Valach, M., D. Moreira, D. Faktorová, J. Lukeš and G. Burger, 2016 Post-transcriptional mending of gene sequences: looking under the hood of mitochondrial gene expression in diplonemids. RNA Biol 13: 1204-1211.
Valach, M., S. Moreira, S. Hoffmann, P. F. Stadler and G. Burger, 2017 Keeping it complicated: mitochondrial genome plasticity across diplonemids. Sci Rep 7: 14166.
Wang, D., Z. Qu, D. L. Adelson, J. K. Zhu and J. N. Timmis, 2014 Transcription of nuclear organellar DNA in a model plant system. Genome Biol Evol 6: 1327-1334.
Wood, V., K. M. Rutherford, A. Ivens, M. A. Rajandream and B. Barrell, 2001 A re-annotation of the *Saccharomyces cerevisiae* genome. Comp Funct Genomics 2: 143-154.

7. Polycistronic transcription units in the nuclear genome of *Diplonema papillatum*

INTRODUCTION

In most eukaryotes, each nuclear protein-coding gene has its own promoter, with the consequence that neighboring genes may or may not be located on the same DNA strand. In contrast, all nuclear genomes of kinetoplastids analyzed to date have gene clusters of up to 100 kbp in length, grouping up to a hundred members arranged in the same orientation (EL-SAYED *et al.* 2005; JACKSON *et al.* 2016). Genes within these clusters are frequently co-transcribed, with the corresponding gene arrays referred to as Polycistronic Transcription Units (PTUs; (MARTÍNEZ-CALVILLO *et al.* 2003)). In *Trypanosoma*, promoters were pinpointed systematically upstream of PTUs, but some promoters were also found within PTU arrays (KOLEV *et al.* 2010). Polycistronic primary transcripts are processed by a specialized spliceosome via *trans*-splicing that involves the attachment of a short (39 nt-long in *Trypanosoma*) spliced-leader (SL) RNA —decorated by a hyper-modified cap— to the 5′ end of each individual mRNA (reviewed in (MICHAELI 2011)). Messenger RNA maturation is finalized by cleaving off the portion downstream of the transcript's 3′ UTR followed by the addition of a poly(A) tail (reviewed in (CLAYTON AND MICHAELI 2011)).

RESULTS AND DISCUSSION

Inspection of the annotated *Diplonema* genome assembly showed that many predicted protein-coding genes in a given contig were arranged head-to-tail, suggesting co-transcription. Therefore, we examined in more detail clusters of five or more protein-coding genes (referred to here as 'unidirectional gene arrays') occurring in contigs larger than 50 kbp.

About 87% (1,159) of these contigs carried at least one unidirectional gene array, with the median size of approximately nine genes in a row and with the longest array having >120 members (for details, see **Additional File 3** polycistTranscripts). The longer contigs, in particular, contained several shorter arrays in a row having the same orientation, most often separated by a single gene model on the other strand. Closer scrutiny of several dozen such cases revealed that virtually all 'trend-disrupting' genes lacked evidence for transcription. Further, most of them were related to open reading frames contained in transposons or dispersed repeat elements that are uniquely found in the *D. papillatum* genome sequence (see also the **Supplementary Information**: Section 2. Assembly and annotation of the nuclear genome and transcriptome of *Diplonema papillatum*, in particular, the section on the expert validation of gene models).

Based on the three longest contigs whose gene models we manually curated, we estimated the extent of polycistronic transcription units in the genome of *D. papillatum*. For example, of the initial 11 strand switches in contig tig00022679, only a single one remained after curation, leading to arrays comprising up to 52 genes. By extrapolation, the *Diplonema* genome is believed to contain two to three times longer unidirectional gene arrays compared to the prediction by the automated structural annotation. Among the expert-confirmed arrays, the longest extends to more than 1 Mbp (tig00022654_12).

In *Trypanosoma*, arrays of about a hundred unidirectional genes are transcribed into several polycistronic RNAs (KOLEV *et al.* 2010). Therefore, it is possible that the gene arrays in *Diplonema*, too, contain multiple promoters. These are likely located in intergenic regions that stand out for their large size. For example, contig tig00022654_12 includes six intergenic regions that are >10 kbp long. Given these predictions, it will be interesting to examine transcription initiation and termination positions of *Diplonema* nuclear genes by experimental means. For example, it was shown in kinetoplastids that modified bases provide the signal for transcription termination (VAN LUENEN *et al.* 2012; SCHULZ *et al.* 2016).

In conclusion, *D. papillatum* and kinetoplastids appear to share an organization of nuclear genes into sizeable PTUs, and a relatively small number of RNAP II transcription start sites per chromosome. While the organizational principle may be the same in the two sister taxa, the actual gene order is very different, since we did not detect any signs of synteny between the genomes of *D. papillatum* on the one hand and *Trypanosoma brucei*, *Leishmania tarentolae* or *Bodo saltans* on the other.

MATERIALS AND METHODS

The orientation of protein-coding genes along a contig was extracted from the genome annotation file (gff). The identification of spliced-leader trans-splicing sites is described in the **Supplementary Information** : Section 2. Assembly and annotation of the nuclear genome and transcriptome of *Diplonema papillatum*.

AUTHOR CONTRIBUTIONS

Conceptualization, Investigation – G.B., M.V.; **Formal analysis, Writing, original draft –** M.V.; **Writing, review & editing –** all co-authors.

REFERENCES

Clayton, C., and S. Michaeli, 2011 3' processing in protists. Wiley Interdiscip Rev RNA 2**:** 247-255.

El-Sayed, N. M., P. J. Myler, G. Blandin, M. Berriman, J. Crabtree *et al.*, 2005 Comparative genomics of trypanosomatid parasitic protozoa. Science 309**:** 404-409.

Jackson, A. P., T. D. Otto, M. Aslett, S. D. Armstrong, F. Bringaud *et al.*, 2016 Kinetoplastid phylogenomics reveals the evolutionary innovations associated with the origins of parasitism. Current Biology : CB 26**:** 161-172.

Kolev, N. G., J. B. Franklin, S. Carmi, H. Shi, S. Michaeli *et al.*, 2010 The transcriptome of the human pathogen *Trypanosoma brucei* at single-nucleotide resolution. PLoS Pathog 6**:** e1001090.

Martínez-Calvillo, S., S. Yan, D. Nguyen, M. Fox, K. Stuart *et al.*, 2003 Transcription of *Leishmania major* Friedlin chromosome 1 initiates in both directions within a single region. Mol Cell 11**:** 1291-1299.

Michaeli, S., 2011 Trans-splicing in trypanosomes: machinery and its impact on the parasite transcriptome. Future Microbiol 6**:** 459-474.

Schulz, D., M. Zaringhalam, F. N. Papavasiliou and H. S. Kim, 2016 Base J and H3.V regulate transcriptional termination in *Trypanosoma brucei*. PLoS Genet 12**:** e1005762.

van Luenen, H. G., C. Farris, S. Jan, P. A. Genest, P. Tripathi *et al.*, 2012 Glucosylated hydroxymethyluracil, DNA base J, prevents transcriptional readthrough in *Leishmania*. Cell 150**:** 909-921.

8. DNA modifications (5mC and J)

INTRODUCTION

Genomic DNA consists primarily of four bases, adenine (A), guanine (G), cytosine (C), and thymine (T). However, derivatives of this basic alphabet have been documented in numerous organisms, with around 50 naturally occurring modified bases identified so far (SOOD *et al.* 2019; DAI *et al.* 2021). Out of these, the two most frequently encountered modified bases are 5-methyl-cytosine (5mC) and N6-methyl-adenine (6mA) in eukaryotic and prokaryotic genomes, respectively, which serve as epigenetic marks (SUN *et al.* 2015; DE MENDOZA *et al.* 2019).

Most unconventional bases occur across the tree of life, but some display a constrained phylogenetic distribution. A prime example of the latter category is the hyper-modified thymine derivative, β-D-glucopyranosyl-oxymethyluracil, referred to as base J. This nucleotide is restricted to euglenozoans ((GOMMERS-AMPT *et al.* 1993; VAN LEEUWEN *et al.* 1998; DOOIJES *et al.* 2000), reviewed in (BORST AND SABATINI 2008)). The metabolism of base J has been extensively studied in trypanosomes and leishmanias, in which its biosynthesis relies on three protein families: JBP1/2, JGT, and JBP3. JBP1 and JBP2 are DNA-binding thymidine hydroxylases (TH). JBP2 generates *de novo* the modification of thymine to hydroxymethyl-uracil (5hmU), and JBP1 then propagates this base across a larger region (CROSS *et al.* 1999; DIPAOLO *et al.* 2005). Subsequently, the intermediate 5hmU is transformed by the glycosyltransferase JGT to base J (BULLARD *et al.* 2014). The most recently identified J-interacting protein JBP3 binds to DNA enriched in J and recruits additional factors to the modified sites forming a regulatory protein complex (KIEFT *et al.* 2020).

RESULTS

In *Diplonema* nuclear DNA, modified bases were experimentally identified more than two decades ago, notably the taxonomically wide-spread 5mC and the euglenozoan-specific base J (VAN LEEUWEN *et al.* 1998). We searched in the *D. papillatum* genome assembly for homologs of proteins known to be implicated in the metabolism of these two minor DNA bases.

The nuclear genome of *Diplonema* codes for five distinct proteins of the cytosine-5 DNA methyltransferase (DNMT) family, a class of enzymes responsible for the biosynthesis of 5mC (DIPPA_02667, DIPPA_11195, DIPPA_01326, DIPPA_70042, and DIPPA_70054). The latter three belong to the DNMT2 subfamily and most likely act as tRNA methyltransferases. Interestingly, DIPPA_02667 and DIPPA_11195, which are members of the more prominent DNMT3/5 family, affiliate in phylogenetic analyses with *de novo* DNA methyltransferases from bacteria that are also sporadically found in protists and certain fungi (BEWICK *et al.* 2019). This distribution suggests that *D. papillatum* acquired these genes *via* horizontal transfer. We also detected homologs of a dozen oxidative demethylases belonging to the AlkB family. Some of these predicted enzymes might be specifically responsible for the C5-demethylation of nuclear DNA.

We also searched in the inferred *Diplonema* proteome for homologs of the three protein families involved in the base J metabolism from kinetoplastids. *D. papillatum* (and all other examined diplonemids and *Euglena*) seems to lack JBP2, but encodes two paralogs of JBP1, namely DIPPA_70009 (DpJBP1B) and DIPPA_70011 (DpJBP1A). The latter possesses in addition to TH and J-DNA-binding domains, an atypical C-terminal methyltransferase domain (PF13489). The top candidate for being a JGT homolog is DIPPA_30303, which encodes a glycosyltransferase.

Lastly, we identified two JBP3 homologs in *Diplonema* (as well as in other diplonemids), which we refer to as JBP3A (DIPPA_17973) and JBP3B (DIPPA_28081). The latter protein is more closely related to the sole JBP3 in kinetoplastids. *Leishmania* JGT and JBP3 were shown to form a complex together with the proteins PP1, Wdr82/Swd2, and PNUTS (KIEFT *et al.* 2020). However, among dozens of potential homologs, *Diplonema* has no clear orthologs of PP1 and Wdr82, and further appears to lack PNUTS. The base J metabolism either has been rewired, or its components have considerably diverged during euglenozoan evolution.

DISCUSSION

In eukaryotes, C5 methylation plays various roles ranging from gene and transposon silencing to nucleosome positioning (HUFF AND ZILBERMAN 2014; SCHMITZ *et al.* 2019). Given the abundance of non-transcribed repetitive and transposable elements in the *D. papillatum* genome, we posit that 5mC is involved in their transcriptional repression.

Base J, a hallmark feature of euglenozoans, often has a predominantly telomeric localization but is also found elsewhere in the nuclear genome (GENEST *et al.* 2015). While this base functions as a critical genomic silencing marker, its precise role varies. In *Leishmania*, the rare extra-telomeric J bases participate in transcriptional termination of polycistronic units (VAN LUENEN *et al.* 2012), while in trypanosomes, the role of base J is auxiliary to a histone H3 variant (SCHULZ *et al.*

2016). Given the more than 100 kbp-long polycistron-like gene clusters in *Diplonema* (see main text), we predict that in this protist, base J has the same primary function as in kinetoplastids. At the same time, *D. papillatum* differs in two key aspects from kinetoplastids: the richness of silent repetitive DNA and transposable elements in the nuclear genome and the assortment of proteins involved in the base J metabolism. Hence, investigating the diplonemid system promises to provide new insights into the adaptability of this epigenetic pathway.

METHODS

The identification of protein function was performed as described in the **Supplementary Information**: Section 2. Assembly and annotation of the nuclear genome and transcriptome of *Diplonema papillatum*.

AUTHOR CONTRIBUTIONS

Conceptualization, Investigation, Formal analysis, Writing, original draft – M.V.; **Writing, review & editing –** all co-authors.

REFERENCES

Bewick, A. J., B. T. Hofmeister, R. A. Powers, S. J. Mondo, I. V. Grigoriev *et al.*, 2019 Diversity of cytosine methylation across the fungal tree of life. Nat Ecol Evol 3**:** 479-490.

Borst, P., and R. Sabatini, 2008 Base J: discovery, biosynthesis, and possible functions. Annu Rev Microbiol 62**:** 235-251.

Bullard, W., J. Lopes da Rosa-Spiegler, S. Liu, Y. Wang and R. Sabatini, 2014 Identification of the glucosyltransferase that converts hydroxymethyluracil to base J in the trypanosomatid genome. J Biol Chem 289**:** 20273-20282.

Cross, M., R. Kieft, R. Sabatini, M. Wilm, M. de Kort *et al.*, 1999 The modified base J is the target for a novel DNA-binding protein in kinetoplastid protozoans. Embo j 18**:** 6573-6581.

Dai, Y., B. F. Yuan and Y. Q. Feng, 2021 Quantification and mapping of DNA modifications. RSC Chem Biol 2**:** 1096-1114.

de Mendoza, A., R. Lister and O. Bogdanovic, 2019 Evolution of DNA methylome diversity in eukaryotes. J Mol Biol.

DiPaolo, C., R. Kieft, M. Cross and R. Sabatini, 2005 Regulation of trypanosome DNA glycosylation by a SWI2/SNF2-like protein. Mol Cell 17**:** 441-451.

Dooijes, D., I. Chaves, R. Kieft, A. Dirks-Mulder, W. Martin *et al.*, 2000 Base J originally found in kinetoplastida is also a minor constituent of nuclear DNA of *Euglena gracilis*. Nucleic Acids Res 28**:** 3017-3021.

Genest, P. A., L. Baugh, A. Taipale, W. Zhao, S. Jan *et al.*, 2015 Defining the sequence requirements for the positioning of base J in DNA using SMRT sequencing. Nucleic Acids Res 43**:** 2102-2115.

Gommers-Ampt, J. H., A. J. Teixeira, G. van de Werken, W. J. van Dijk and P. Borst, 1993 The identification of hydroxymethyluracil in DNA of *Trypanosoma brucei*. Nucleic Acids Res 21**:** 2039-2043.

Huff, J. T., and D. Zilberman, 2014 Dnmt1-independent CG methylation contributes to nucleosome positioning in diverse eukaryotes. Cell 156**:** 1286-1297.

Kieft, R., Y. Zhang, A. P. Marand, J. D. Moran, R. Bridger *et al.*, 2020 Identification of a novel base J binding protein complex involved in RNA polymerase II transcription termination in trypanosomes. PLoS Genet 16**:** e1008390.

Schmitz, R. J., Z. A. Lewis and M. G. Goll, 2019 DNA methylation: shared and divergent features across eukaryotes. Trends Genet 35**:** 818-827.

Schulz, D., M. Zaringhalam, F. N. Papavasiliou and H. S. Kim, 2016 Base J and H3.V regulate transcriptional termination in *Trypanosoma brucei*. PLoS Genet 12**:** e1005762.

Sood, A. J., C. Viner and M. M. Hoffman, 2019 DNAmod: the DNA modification database. J Cheminform 11**:** 30.

Sun, Q., S. Huang, X. Wang, Y. Zhu, Z. Chen *et al.*, 2015 N6-methyladenine functions as a potential epigenetic mark in eukaryotes. Bioessays 37**:** 1155-1162.

van Leeuwen, F., M. C. Taylor, A. Mondragon, H. Moreau, W. Gibson *et al.*, 1998 beta-D-glucosyl-hydroxymethyluracil is a conserved DNA modification in kinetoplastid protozoans and is abundant in their telomeres. Proc Natl Acad Sci U S A 95**:** 2366-2371.

van Luenen, H. G., C. Farris, S. Jan, P. A. Genest, P. Tripathi *et al.*, 2012 Glucosylated hydroxymethyluracil, DNA base J, prevents transcriptional readthrough in *Leishmania*. Cell 150**:** 909-921.

9. RNA interference (RNAi)

INTRODUCTION

RNA interference (RNAi) is a process thought to have evolved as a defence mechanism against retrotransposons and invading double-stranded (ds) RNA viruses (MALONE AND HANNON 2009). The last eukaryotic common ancestor (LECA) must have been already equipped with a minimal eukaryotic RNA silencing pathway comprising an RNase III-like endonuclease Dicer, PIWI-domain containing proteins (Argonaute [Ago] and Piwi), and an RNA-dependent RNA polymerase (RdRP) (BURROUGHS *et al.* 2014; SWARTS *et al.* 2014). Mechanistically, Dicer processes long dsRNAs into small interfering (si) RNAs that are then loaded onto PIWI-domain proteins. The siRNAs recognize target RNA molecules through complementary base pairing. Subsequently, the target transcript is cleaved, thereby silencing the expression of the corresponding gene. RdRP can either initiate or enhance the response by amplifying the amount of the dsRNA that triggers the cascade.

RESULTS

The *Diplonema* inferred proteome contains all the elementary components of a functional RNAi pathway (**Supplementary Table S18**). The genome assembly includes two homologs of the Dicer-like protein (DCL), notably DpDCL1 (DIPPA_01275) and DpDCL2 (DIPPA_00339). DCL1 is an unusually large gene spanning three contigs; the protein sequence was manually assembled with the help of transcriptome data. Other diplonemids appear to contain only a single Dicer—one that most closely resembles DpDCL1. DpDCL2 likely originated in *D. papillatum* by gene duplication and divergence, as this protein lacks a C-terminal domain that all other diplonemid DCLs otherwise share.

A total of eight PIWI domain-containing proteins were identified in the *D. papillatum* inferred proteome. The five genes coding for Piwi proteins (DIPPA_27834, DIPPA_27835, DIPPA_27840, DIPPA_27841, and DIPPA_70076) are identical in sequence and arranged in a tandem array; the correctness of the assembly at the corresponding locus was verified by examining PacBio reads. In contrast, three members of the Argonaute family (DIPPA_09821, DIPPA_23819, and DIPPA_13995) are distinct in sequence (pairwise identity 93%) and are encoded in different genomic loci. Other diplonemids seem to possess a similar assortment of homologs.

The *Diplonema* genome also encodes a single homolog of RdRP (DIPPA_03987). The protein is characterized by three distinct regions: an N-terminal helicase domain (Pfam families DEAD (PF00270) and Helicase_C (PF00271)), a central RdRP domain (Pfam family RdRP (PF05183)), and a C-terminal low-complexity region. While kinetoplastids are known to lack RdRP (KOLEV *et al.* 2011), other discobids, such as the heterolobosean *Naegleria gruberi*, have undergone an expansion of this family (BURROUGHS *et al.* 2014).

Lastly, we searched for homologs of two novel kinetoplastid-specific RNAi factors discovered in *Trypanosoma brucei*, i.e., TbRIF4, which is responsible for generating single-stranded siRNAs from duplexes, and TbRIF5, which is a cofactor of *Trypanosoma* DCL1 (BARNES *et al.* 2012). In *Diplonema* (and in several other diplonemids), we found a single homolog of TbRIF4 (DIPPA_70075, another large gene spanning three contigs in the assembly). Still, a counterpart of the Dicer cofactor was not detected.

Supplementary Table S18. Proteins involved in RNAi of *D. papillatum*.

Dicer	Argonaute		RdRP	Co-factors
	Piwis	Agos		
DIPPA_01275 (DCL1)	DIPPA_27834	DIPPA_09821	DIPPA_03987	DIPPA_70075 (RIF4)
DIPPA_00339 (DCL2)	DIPPA_27835	DIPPA_23819		
	DIPPA_27840	DIPPA_13995		
	DIPPA_27841			
	DIPPA_70076			

DISCUSSION

The predicted RNAi-implicated protein repertoire in *D. papillatum*, and diplonemids in general, indicates a less streamlined pathway compared to kinetoplastids. In particular the finding of a signal-amplifying RdRP gene in the *Diplonema* nuclear genome suggests a more conventional RNAi machinery.

In addition, all examined diplonemids possess multiple Piwi and Ago proteins, which contrasts with kinetoplastids. For instance, in *T. brucei*, its sole AGO1 is responsible for all forms of mRNA silencing, irrespective of whether siRNA duplexes are generated by the cytoplasmic Dicer-like (DCL) enzyme 1 or the nucleus-located DCL2 (KOLEV *et al.* 2011). The function of the PIWI-only TbPWI1 is unclear, but the protein is apparently not involved in RNAi (DURAND-DUBIEF AND BASTIN 2003). Curiously, in phylogenetic trees, kinetoplastid Piwi (Kiwi) and Ago proteins cluster together to the exclusion of Argonautes and Piwis from all other eukaryotes (SWARTS *et al.* 2014). Based on our phylogenetic analysis, all diplonemid Ago proteins belong to the same family, including their kinetoplastid counterparts (**Supplementary Figure S16**, 'euAgo' group, for euglenozoan Ago). In contrast, the Piwi proteins of diplonemids associate with the RNAi-implicated members of the Piwi-like family from other eukaryotes. This indicates that not only Agos, but also Piwis likely contribute to silencing responses in diplonemids.

Traditionally, RNAi has been considered to be involved in post-transcriptional gene regulation and transposon repression. Both processes are critical for *Diplonema* given polycistronic transcription and the large population of transposable elements lingering in its nuclear genome. However, while the *Diplonema* RNAi machinery might well suppress transcripts originating from mobile elements, it has apparently not contained their massive spread throughout the nuclear genome. Alternatively, RNAi in *Diplonema* could be predominantly involved in other processes such as small-RNA-driven translational silencing, genome methylation, or centromere formation, functions that have been documented in various organisms, including animals, plants, fungi and certain protists alike (KETTING 2011; GUTBROD AND MARTIENSSEN 2020). Determining more precisely what roles the individual RNAi pathway components perform in *Diplonema* will require experimental scrutiny.

METHODS

Protein identification and function assignment across diplonemids were performed by searches with BLAST and profile Hidden Markov Models (HMMs) as described in the Supplementary Information on functional genome annotation. To determine the phylogenetic relationships among Piwi and Ago proteins, sequences from diplonemids and selected eukaryotes and prokaryotes from a previous study (SWARTS *et al.* 2014) were pre-aligned with Muscle (EDGAR 2004) and realigned with an HMM search (built with the initial Muscle alignment) using hmmsearch (HMMER 3.3, Nov 2019) (EDDY 2011). Only amino acids aligned with a PP value of 1.0 were retained for phylogenetic analysis, which was performed by a Bayesian and maximum likelihood (ML) approach. For the former, we used PhyloBayes (LARTILLOT *et al.* 2013) by running four independent chains, six gamma categories and the CAT-GTR model. For the ML approach, we used IQ-TREE with default parameters and the option to calculate 1,000 ultrafast bootstrap replicates (MINH *et al.* 2020).

AUTHOR CONTRIBUTIONS

Conceptualization, Data curation, Investigation, Writing, original draft – C.B., M.V.; **Formal analysis, Visualization –** B.F.L., M.V.; **Writing, review & editing –** all co-authors.

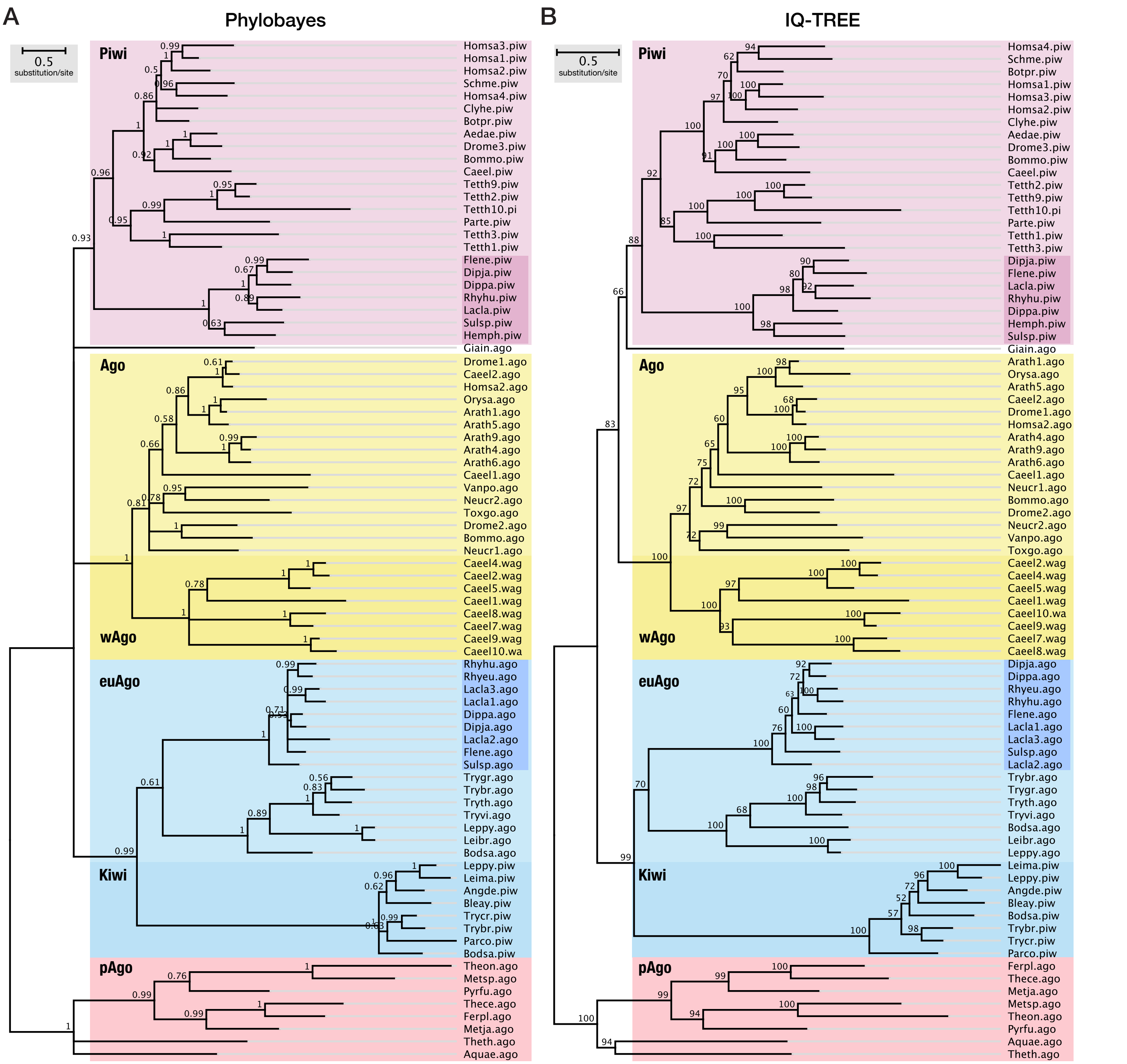

Supplementary Figure S16. Phylogeny of Piwi-domain containing proteins. The phylogenetic tree was constructed with Bayesian (**A**) and maximum likelihood (**B**) methods using the Piwi-domain sequences of proteins across eukaryotes and from several prokaryotes as outgroups (pAgos). Posterior probabilities (Phylobayes) and UF bootstrap support values (IQ-TREE) are indicated next to each branch. Both approaches resolve the tree into several clusters of related sequences, namely: conventional Piwi proteins from various eukaryotes (Piwi), conventional Ago proteins from various eukaryotes (Ago), nematode-specific Ago protein expansion (wAgo), kinetoplastid and diplonemid Ago proteins (euAgo), kinetoplastid-specific Piwi proteins (Kiwi), and prokaryotic Ago proteins (pAgo). Diplonemid taxa are highlighted in darker background shades.

REFERENCES

Barnes, R. L., H. Shi, N. G. Kolev, C. Tschudi and E. Ullu, 2012 Comparative genomics reveals two novel RNAi factors in *Trypanosoma brucei* and provides insight into the core machinery. PLoS Pathog 8**:** e1002678.

Burroughs, A. M., Y. Ando and L. Aravind, 2014 New perspectives on the diversification of the RNA interference system: insights from comparative genomics and small RNA sequencing. Wiley Interdiscip Rev RNA 5**:** 141-181.

Durand-Dubief, M., and P. Bastin, 2003 TbAGO1, an argonaute protein required for RNA interference, is involved in mitosis and chromosome segregation in *Trypanosoma brucei*. BMC Biol 1**:** 2.

Eddy, S. R., 2011 Accelerated profile HMM searches. PLoS Comput Biol 7**:** e1002195.

Edgar, R. C., 2004 MUSCLE: multiple sequence alignment with high accuracy and high throughput. Nucleic Acids Res 32**:** 1792-1797.

Gutbrod, M. J., and R. A. Martienssen, 2020 Conserved chromosomal functions of RNA interference. Nat Rev Genet 21**:** 311-331.

Ketting, R. F., 2011 The many faces of RNAi. Dev Cell 20**:** 148-161.

Kolev, N. G., C. Tschudi and E. Ullu, 2011 RNA interference in protozoan parasites: achievements and challenges. Eukaryot Cell 10**:** 1156-1163.

Lartillot, N., N. Rodrigue, D. Stubbs and J. Richer, 2013 PhyloBayes MPI: phylogenetic reconstruction with infinite mixtures of profiles in a parallel environment. Syst Biol 62**:** 611-615.

Malone, C. D., and G. J. Hannon, 2009 Small RNAs as guardians of the genome. Cell 136**:** 656-668.

Minh, B. Q., H. A. Schmidt, O. Chernomor, D. Schrempf, M. D. Woodhams *et al.*, 2020 IQ-TREE 2: New models and efficient methods for phylogenetic inference in the genomic era. Mol Biol Evol 37**:** 1530-1534.

Swarts, D. C., K. Makarova, Y. Wang, K. Nakanishi, R. F. Ketting *et al.*, 2014 The evolutionary journey of Argonaute proteins. Nat Struct Mol Biol 21**:** 743-753.

10. The cytosolic ribosome of *Diplonema papillatum*

Gel electrophoresis under denaturing conditions demonstrated that the *Diplonema* cytosolic ribosome (cytoribosome) contains the usual four eukaryotic rRNA species: 18S, 5.8S, 26S, and 5S. The bipartite *Diplonema* large subunit (LSU) rRNA (5.8S + 26S) stands in marked contrast to the cytosolic LSU rRNAs of other euglenozoans, where the 26S rRNA component is further fragmented into six distinct pieces in kinetoplastids (GRAY 1981; CAMPBELL *et al.* 1987; SPENCER *et al.* 1987; HASHEM *et al.* 2013; LIU *et al.* 2016) and 13 separate pieces in the euglenid *Euglena gracilis* (SCHNARE *et al.* 1990; SCHNARE AND GRAY 1990; HAŁAKUC *et al.* 2022). Although some of the additional processing sites are in a similar site in kinetoplastid and euglenid LSU rRNAs (SCHNARE *et al.* 1990), these positionally equivalent processing events must have arisen independently in the two lineages, given that diplonemids are evolutionarily intermediate between kinetoplastids and euglenids (HAŁAKUC *et al.* 2022).

The *Diplonema* genome encodes all 33 small subunit (SSU) ribosomal proteins (RPs) found in the human cytoribosome. In addition, the *Diplonema* SSU is predicted to contain an ortholog of a novel RNA-binding protein that has been identified by cryo-EM in the SSU of kinetoplastids (BRITO QUERIDO *et al.* 2017) and *E. gracilis* (MATZOV *et al.* 2020). We also identified all 47 LSU RPs found in the human cytoribosome.

To gain insight into the organization and evolution of cyt-RP genes in *Diplonema*, we carried out a detailed manual examination of SSU and LSU cyt-RP genes (of which there are in total 109 and 155, respectively), CDS and their predicted amino acid sequences. Several themes emerged from the study of identified cyt-RP genes.

- As in other eukaryotes (LECOMPTE *et al.* 2002), multiple copies exist for each of the *Diplonema* SSU and LSU cyt-RPs. Gene copies (2–9) may be localized in a single genomic scaffold (e.g., five copies of uS3 on tig00023122_3) or distributed among two or more scaffolds. When copies are located on the same scaffold, they are invariably found in the same transcriptional orientation and generally separated by several thousand base pairs.
- Just over half of the 109 SSU and 155 LSU cyt-RP gene copies (57 and 86, respectively) contain introns (1–3 per copy). With few exceptions, intron-containing copies have the same number of introns, and these are invariably located at the same position within the CDS. With very few exceptions (e.g., I3 in duplicate uS7 copies), positionally equivalent introns differ considerably in size and display no significant sequence similarity with one another. Intron size generally ranges from a few hundred to a few thousand bp, with an unusually long intron (23,385 bp) in one uS13 gene copy (vs. 416 bp in the other).
- In duplicate copies of the uS8 gene, a single intron is present in the 3′-UTR, inserted after position 14 in copy #1 and position 16 in copy #2. Comparison of genome and transcriptome sequence data provides evidence of alternative splicing of the copy #1 transcript, in which the 3′ UTR intron removed is either 233 or 301 nt long (both GT...AG).
- In comparing CDS sequences of different copies of the same gene, no consistent pattern of sequence conservation/divergence emerges. In some cases, CDSs are identical, in other cases quite divergent. For example, duplicate copies of the uS8 gene are present in two separate genomic scaffolds. The duplicates on each scaffold have an identical CDS, but the CDSs between duplicate pairs differ by 55 SNPs (86% identity) and predicted protein sequences differ at 9 positions (93% identity). In other cases, the CDSs in multiple copies differ by only a single or a few SNPs, all of which are at silent 1st or 3rd codon positions, whereas in other instances SNPs are relatively numerous (e.g., 21 SNPs and 95.5% identity between the duplicate uS13 copies). But again, all SNPs are silent so that the predicted amino acid sequence is unaffected.
- Yet another pattern is exemplified by the duplicate copies of uS2 (DIPPA_07808 and DIPPA_05951), whose CDSs differ by 162 SNPs (82% identity) and predicted protein sequences differ at 61 positions (75% identity). Nevertheless, despite their divergent sequences, BLASTp against NCBI nr with both protein variants retrieves the same top hit (‘40S small subunit ribosomal protein uS2 [Euglena gracilis]’).
- Another type of variation arises from terminal extensions or truncations. For instance, the two uS14 copies, located on different contigs, have a length of 150 aa (DIPPA_22991) and 100 aa (DIPPA_33894). Notably, all the genes discussed here are transcribed at an exceptionally high level.
- Overall, CDS identity between multiple copies of the same gene ranges from ~80% to 100% and inferred amino acid sequence between ~87% and 100%, although the latter is generally >95%. These results indicate the emergence of paralogs by gene duplication and divergence within the diplonemid lineages rather than by horizontal acquisition of a new homolog.

In summary, detailed examination of *Diplonema* cyt-RP genes did not reveal a consistent pattern that would allow one to draw meaningful conclusions about the mechanism of duplication and sequence divergence of these genes. The data do indicate the existence of sequence variants of a substantial number of individual *Diplonema* cyt-RPs. Although further work would be necessary to confirm transcription of individual cyt-RP gene variants that differ in sequence by only a few nucleotides, current evidence from transcript quantification indicates that essentially all copies of a particular cyt-RP are expressed in the form of mRNAs. It remains to be determined what the protein levels are, whether expression is constitutive, and if certain variants are preferred under certain environmental or physiological conditions. These observations leave open the possibility that *Diplonema* cytoribosomes may be heterogeneous with respect to their composition of individual RPs. Compositional ribosome heterogeneity has been a subject of considerable conjecture and experimentation (GILBERT 2011; SLAVOV *et al.* 2015; SHI *et al.* 2017; GENUTH AND BARNA 2018; FERRETTI AND KARBSTEIN 2019; GHULAM *et al.* 2020; MARTINEZ-SEIDEL *et al.* 2020; NORRIS *et al.* 2021).

AUTHOR CONTRIBUTIONS

Conceptualization, Data curation, Writing, original draft – M.W.G.; **Investigation –** M.W.G., M.V.; **Writing, review & editing –** all co-authors.

REFERENCES

Brito Querido, J., E. Mancera-Martínez, Q. Vicens, A. Bochler, J. Chicher *et al.*, 2017 The cryo-EM structure of a novel 40S kinetoplastid-specific ribosomal protein. Structure 25**:** 1785-1794.e1783.

Campbell, D. A., K. Kubo, C. G. Clark and J. C. Boothroyd, 1987 Precise identification of cleavage sites involved in the unusual processing of trypanosome ribosomal RNA. J Mol Biol 196**:** 113-124.

Ferretti, M. B., and K. Karbstein, 2019 Does functional specialization of ribosomes really exist? Rna 25**:** 521-538.

Genuth, N. R., and M. Barna, 2018 Heterogeneity and specialized functions of translation machinery: from genes to organisms. Nat Rev Genet 19**:** 431-452.

Ghulam, M. M., M. Catala and S. Abou Elela, 2020 Differential expression of duplicated ribosomal protein genes modifies ribosome composition in response to stress. Nucleic Acids Res 48**:** 1954-1968.

Gilbert, W. V., 2011 Functional specialization of ribosomes? Trends Biochem Sci 36**:** 127-132.

Gray, M. W., 1981 Unusual pattern of ribonucleic acid components in the ribosome of *Crithidia fasciculata*, a trypanosomatid protozoan. Mol Cell Biol 1**:** 347-357.

Hałakuc, P., A. Karnkowska and R. Milanowski, 2022 Typical structure of rRNA coding genes in diplonemids points to two independent origins of the bizarre rDNA structures of euglenozoans. BMC Ecol Evol 22**:** 59.

Hashem, Y., A. des Georges, J. Fu, S. N. Buss, F. Jossinet *et al.*, 2013 High-resolution cryo-electron microscopy structure of the *Trypanosoma brucei* ribosome. Nature 494**:** 385-389.

Lecompte, O., R. Ripp, J. C. Thierry, D. Moras and O. Poch, 2002 Comparative analysis of ribosomal proteins in complete genomes: an example of reductive evolution at the domain scale. Nucleic Acids Res 30**:** 5382-5390.

Liu, Z., C. Gutierrez-Vargas, J. Wei, R. A. Grassucci, M. Ramesh *et al.*, 2016 Structure and assembly model for the *Trypanosoma cruzi* 60S ribosomal subunit. Proc Natl Acad Sci U S A 113**:** 12174-12179.

Martinez-Seidel, F., O. Beine-Golovchuk, Y. C. Hsieh and J. Kopka, 2020 Systematic review of plant ribosome heterogeneity and specialization. Front Plant Sci 11**:** 948.

Matzov, D., M. Taoka, Y. Nobe, Y. Yamauchi, Y. Halfon *et al.*, 2020 Cryo-EM structure of the highly atypical cytoplasmic ribosome of *Euglena gracilis*. Nucleic Acids Res 48**:** 11750-11761.

Norris, K., T. Hopes and J. L. Aspden, 2021 Ribosome heterogeneity and specialization in development. Wiley Interdiscip Rev RNA 12**:** e1644.

Schnare, M. N., J. R. Cook and M. W. Gray, 1990 Fourteen internal transcribed spacers in the circular ribosomal DNA of *Euglena gracilis*. J Mol Biol 215**:** 85-91.

Schnare, M. N., and M. W. Gray, 1990 Sixteen discrete RNA components in the cytoplasmic ribosome of *Euglena gracilis*. J Mol Biol 215**:** 73-83.

Shi, Z., K. Fujii, K. M. Kovary, N. R. Genuth, H. L. Röst *et al.*, 2017 Heterogeneous ribosomes preferentially translate distinct subpools of mRNAs genome-wide. Mol Cell 67**:** 71-83.e77.

Slavov, N., S. Semrau, E. Airoldi, B. Budnik and A. van Oudenaarden, 2015 Differential stoichiometry among core ribosomal proteins. Cell Rep 13**:** 865-873.

Spencer, D. F., J. C. Collings, M. N. Schnare and M. W. Gray, 1987 Multiple spacer sequences in the nuclear large subunit ribosomal RNA gene of *Crithidia fasciculata*. EMBO J 6**:** 1063-1071.

11. Meiosis in *Diplonema papillatum*?

INTRODUCTION

Meiosis is a prerequisite for sexual reproduction as it produces haploid gamete cells. In *D. papillatum* and other diplonemids, meiotic processes have not been observed and are not induced by starvation as in many other systems. This raises the questions of (i) whether *D. papillatum* has the potential to reproduce sexually and, if so, (ii) whether the crossover is mediated by the synaptonemal complex, which is the preferred pathway in most examined eukaryotic species.

At the cell-biology level, meiosis proceeds in two principal stages. First, homologous chromosomes pair with each other, followed by programmed double-strand breaks and strand exchange. Second, meiotic crossovers are formed, and then the joint molecules are resolved (KOHL AND SEKELSKY 2013). In organisms for which cytological evidence is lacking or genetics tools are not available, the potential for sexual reproduction is examined by scrutinizing the genome for homologs of genes involved in the meiosis of model organisms (*Saccharomyces cerevisiae*, *Schizosaccharomyces pombe, Drosophila,* and *C. elegans*). Below, we will briefly discuss the genes involved in the various steps of meiosis and report for which genes we detected homologs in the *D. papillatum* nuclear genome assembly version 1.

RESULTS AND DISCUSSION

Genes involved in early meiosis

Pairing of homologous chromosomes proceeds in three steps, each catalyzed or controlled by a specific set of genes conserved across eukaryotes (KOHL AND SEKELSKY 2013). In the following, the names of genes and proteins that are meiosis-specific are shown in red, and those that also act in mitosis or other processes are shown in turquoise; genes detected in the ***D. papillatum*** nuclear genome are set in bold (**Supplementary Table S19**).
The three steps of homologous chromosome pairing and the proteins involved in these steps are:

1. Programmed DNA double-strand breaks (DSBs), induced by the **SPO11** endonuclease;
2. Processing of DSBs and ssDNA regions, performed by the Replication Protein A (**RPA**), a heteromeric complex;
3. Formation of extended D-loops resulting in joint molecules, catalyzed by the recombinases **DMC1** and **RAD51**, and assisted by **HOP2** and **MND1**.

In yeast, REC102, REC104 and REC114 have been shown to interact with **SPO11** (SASANUMA *et al.* 2007; KOHL AND SEKELSKY 2013), but they appear to have no homologs outside the fungi.

The second part of meiosis

Subsequent meiotic crossovers and the resolution of the joint molecule may follow either of two distinct crossover pathways, Pathway I or II. Most eukaryotes are equipped for both pathways but prefer using one or the other.

Crossover Pathway I is characterized by the formation of a synaptonemal complex during meiotic prophase I, a structure that is thought to stabilize meiotic pairing. This pathway is the preferred route in, e.g., *Caenorhabditis* and *Drosophila* ((KOHL AND SEKELSKY 2013) and references therein). Genes encoding components of the synaptonemal complex, such as the lateral-element proteins RED1 and HOP1 and the recombination-nodule proteins ZIP1, ZIP2, ZIP3, and SPO22 are specific for Pathway I. Further involved in this pathway are the DNA helicase MER3 and the mutS-homologs **MSH4** and **MSH5** (PAGE AND HAWLEY 2004). The latter two proteins also participate in general DNA repair. Note that eukaryotes typically encode four distinct mutS-homologs (yeast even has a total of six), which are highly similar to each other in their protein sequence. In the *Diplonema* nuclear genome, we were unable to retrieve any of the homologs of the meiosis-specific Pathway-I components RED1, HOP1 and ZIP1-4, except a moderately supported MER3 (see phylogeny below). However, we detected homologs of **MSH4** and **MSH5**, and further MSH2, MSH3, MSH6 that are all probably involved in general DNA repair.

Crossover Pathway II, the exclusive mode of crossover formation in the fission yeast, uses mitotic DNA-repair functions involving non-interfering crossovers. Thus, Pathway-II-specific meiosis genes do not exist. The complex that resolves meiotic crossovers in *S. pombe* is composed of **MUS81** and MMS4. Experimental evidence shows that *Tetrahymena thermophila* generates crossovers by the Class II pathway. According to their gene repertoire, i.e., absence of Pathway I genes and presence of **MUS81**, other ciliates are probably also capable of Class II crossovers (see (CHI *et al.* 2014) and references therein). Note that MMS4 has not been detected in ciliate genomes, suggesting that it is not essential for Pathway II. In the *Diplonema* nuclear genome, we detected a homolog of **MUS81**, but no significant hit with MMS4.

Meiotic divisions

Essentially all eukaryotes go through haploid and diploid phases, and depending on the predominant stage, a species is classified as being haploid or diploid. Consequently, there is no difference in the meiosis of haploid and diploid organisms. The (either interim or long-term) diploid undergoes two rounds of specialized cell divisions, Meiosis I and II, without intervening S-phase. Prior to entry into Meiosis I, homologous chromosomes (originating from each parent) pair and undertake meiotic recombination, thereby generating new genetic combinations in the offspring. During this step, homologous chromosomes are segregated into daughter cells, whereas sister chromatids are segregated during Meiosis II. In the case of diploid organisms, the haploid products of Meiosis II function as sexual spores that fuse to form a zygote, which then multiplies by mitosis. In the case of haploid species, the haploid product of Meiosis II corresponds to the 'adult' organism that directly multiplies by mitosis.

Meiosis II largely resembles mitotic division. One of the differences found, e.g., in yeast and human, is that the cohesin subunit **Rad21** (termed SSC1 in *S. cerevisiae*), which otherwise functions in mitosis, is substituted by a specialized meiotic form, named REC8 (WASSMANN 2013).

Distinction of closely related homologs

Several meiosis proteins have closely related counterparts and are therefore easily confused. These include **DMC1** and **RAD51**; MSH2, MSH3, **MSH4**, **MSH5** and MSH6; **RAD21** and REC8; and MER3 and other ATP-dependent DNA and RNA helicases. All of the above function assignments of *D. papillatum* proteins have been confirmed by protein-domain content and arrangement. In the case of confounding paralogs, we performed phylogenetic analyses and transferred the function annotation of a given SwissProt protein to the *Diplonema* protein, if they group together in a well-supported clade.

Diplonema **DMC1** and **RAD51** could be clearly distinguished based on their affiliation with the corresponding kinetoplastid homologs (**Supplementary Figure S17A**). Similarly, MSH2 to MSH6 from diverse eukaryotic groups including *D. papillatum* form coherent clades (**Supplementary Figure S17B**). To validate the DNA-helicase MER3 candidates of *Diplonema*, we added to the dataset DEAD/DEAH box RNA helicases and U5 small nuclear RNA helicase that have significant sequence similarity to MER3. Of the initial three MER3 candidates in *Diplonema*, one affiliates with the MER3 clade, including confirmed orthologs from plants, human, and yeast (**Supplementary Figure S17C**).

Evidence of meiosis in Discoba

Finding orthologs of genes whose involvement in meiosis has been demonstrated experimentally in model organisms does not prove that *D. papillatum* is indeed capable of sexual reproduction. The same caution applies, e.g., to the discoban *Trichomonas* (MALIK *et al.* 2007) and the dinoflagellate *Symbiodinium*. In these organisms, 'meiosis' genes had been identified, but otherwise, cytological or genetic evidence for sexual processes is lacking (CHI *et al.* 2014). . In addition, meiosis genes can be present in a genome despite the absence of sexual reproduction, as was demonstrated in *Giardia intestinalis* where **DMC1**, **SPO11**, and HOP1 act during *parasexual* genetic recombination (CARPENTER *et al.* 2012). Further, in *Acanthamoeba*, meiosis genes are expressed constitutively, which led to the proposition that the corresponding gene products are involved in biological processes other than meiosis (MACIVER *et al.* 2019).

Although protists had historically been considered to propagate only clonally, cytological evidence supporting meiotic processes exists for quite a number of groups (reviewed in (HEYWOOD 1976)). Among Discoba, conventional meiotic division has been demonstrated in trypanosomes by examining the inheritance of genetic markers. Meiosis is further supported by the finding that *Trypanosoma brucei* temporally expresses **DMC1**, HOP1, **MND1**, and **SPO11**, and forms a synaptonemal complex that was demonstrated by immunofluorescence experiments ((PEACOCK *et al.* 2011) and references therein).

In euglenids, traditionally believed to reproduce exclusively mitotically, indications for meiosis come from cytological studies of *Hyalophacus* (LEEDALE 1967). For *Euglena gracilis*, corresponding experimental data are not available. Still, its genome includes a complete set of meiosis-specific genes (EBENEZER *et al.* 2017), although **DMC1**, REC8, and **SPO11** seem not to be transcribed (under the conditions tested (EBENEZER *et al.* 2019)).

Sexual reproduction of the heterolobosean *Naegleria* was suggested based on isoenzyme analysis (PERNIN *et al.* 1992). However, isoenzymes can arise from conditions other than meiosis, for example paralogs that arose via gene duplication or post-translational processing. Therefore, isoenzyme analyses are only conclusive in connection with genetic crosses and the analysis of offspring as conducted for *T. brucei* (SCHWEIZER *et al.* 1994). Finally, several studies have provided indirect evidence for sexual processes in protists, e.g., by searching for the existence of genes encoding proteins that function in cell and nuclear fusion (SPEIJER *et al.* 2015).

Supplementary Table S19. ***D. papillatum*** **homologs of genes involved in meiosis**

Protein Name/component[a]	Gene[b]	Distribution[c]	Synonym	Dp protein ID	TPM (RNA-Seq)[d]
Meiotic recombination	**DMC1***	broad	LIM15	**DIPPA_08042**	32.47744 (7,852)
Mei-specific/SC lateral element	**HOP1***	broad	ASY1	**DIPPA_03672**	2.049079 (622)
Homol-pairing/Class II pathway	**HOP2***	broad	/	**DIPPA_04049**	0.756920 (101)
ATP-dep DNA helicase/SC?	**MER3**	broad	HFM1	**DIPPA_17120**	0.138232 (224)
Meiotic nuclear division	**MND1***	broad	/	**DIPPA_03210**	0.74791 (96)
DNA mismatch repair/SC	**MSH4**	broad	/	**DIPPA_32943**	0.426741 (408)
DNA mismatch repair/SC	**MSH5**	broad	/	**DIPPA_30171**	0.564389 (360)
Meiotic recombination	**REC114**	Ascom	REC7	/	/
Meiotic recombination	**REC8***	broad	SYN2	/	/
Reductive division/SC axial element	**RED1**	Ascom	REC10	/	/
Meiotic recombination	**SPO11***	broad	REC12	**DIPPA_09542**	0.507504 (130)
SC central element	**ZIP1**	Ascom	/	/	/
SC central element	**ZIP2**	Ascom	/	/	/
SC central element	**ZIP3**	broad	/	/	/
SC central element	**ZIP4**	Ascom	SPO22	/	/
Complex formation of SPO11, SC	REC102	Ascom	/	/	/
Meiosis specific	MEI4	Opisth	/	/	/
Recombination protein	MER2	Ascom	REC107	/	/
DSB-repair	MRE11	broad	/	DIPPA_08235	11.030102 (2,396)
DNA mismatch repair	MSH1	broad	/	/	/
DNA mismatch repair	MSH2	broad	/	DIPPA_28873	3.458633 (2,452)
DNA mismatch repair	MSH3	broad	/	DIPPA_30820	7.495374 (5,815)
DNA mismatch repair	MSH6	broad	/	DIPPA_04082	1.134347 (881)
Cross-over junction endonuclease	MUS81	broad	/	DIPPA_25323	1.026156 (1,926)
Sisterchromatid separation	RAD21	broad	SSC1	DIPPA_12003	24.029910 (9,023)
DNA repair	RAD50	Ascom	/	DIPPA_22997	3.062462 (1,702)
				DIPPA_20691	3.134435 (1,742)
				DIPPA_12463	5.720618 (2,516)
Homologous-recombination repair	RAD51	broad	/	DIPPA_21552	3.939032 (969)
Antiviral SKI8	REC103	Ascom	SKI8	/	/
Meiotic recombination	REC104	Ascom	/	/	/
Meiotic recombination	REC114	Opisth	/	/	/
Replication factor A protein 1	RPA1	broad	RPA 70 kDa	DIPPA_33311	22.036753 (8,573)
Replication factor A protein 2	RPA2	broad	RPA 32 kDa	DIPPA_08046	9.324137 (1,402)
DNA repair	XRS2	Ascom	/	/	/

[a] Protein name/function from SwissProt or literature; SC, synaptonemal complex.
[b] Asterisks indicate proteins considered to be a core meiotic component (PEACOCK *et al.* 2011; CHI *et al.* 2014); underscoring of protein names indicates homologs that require phylogenetic analysis for accurate function assignment; **red**, meiosis-specific proteins; **blue**, proteins that participate in meiosis and mitosis or other processes.
[c] Taxonomic distribution. Ascom, Ascomycetes; Opisth, Opisthokonts.
[d] Transcripts per Million (TPM) and RNA-Seq reads mapped, i.e., read count (in brackets). For details on transcript quantification based on RNA-Seq read mapping, see **Supplementary Information**: Section 2. Assembly and annotation of the nuclear genome and transcriptome of *Diplonema papillatum*.

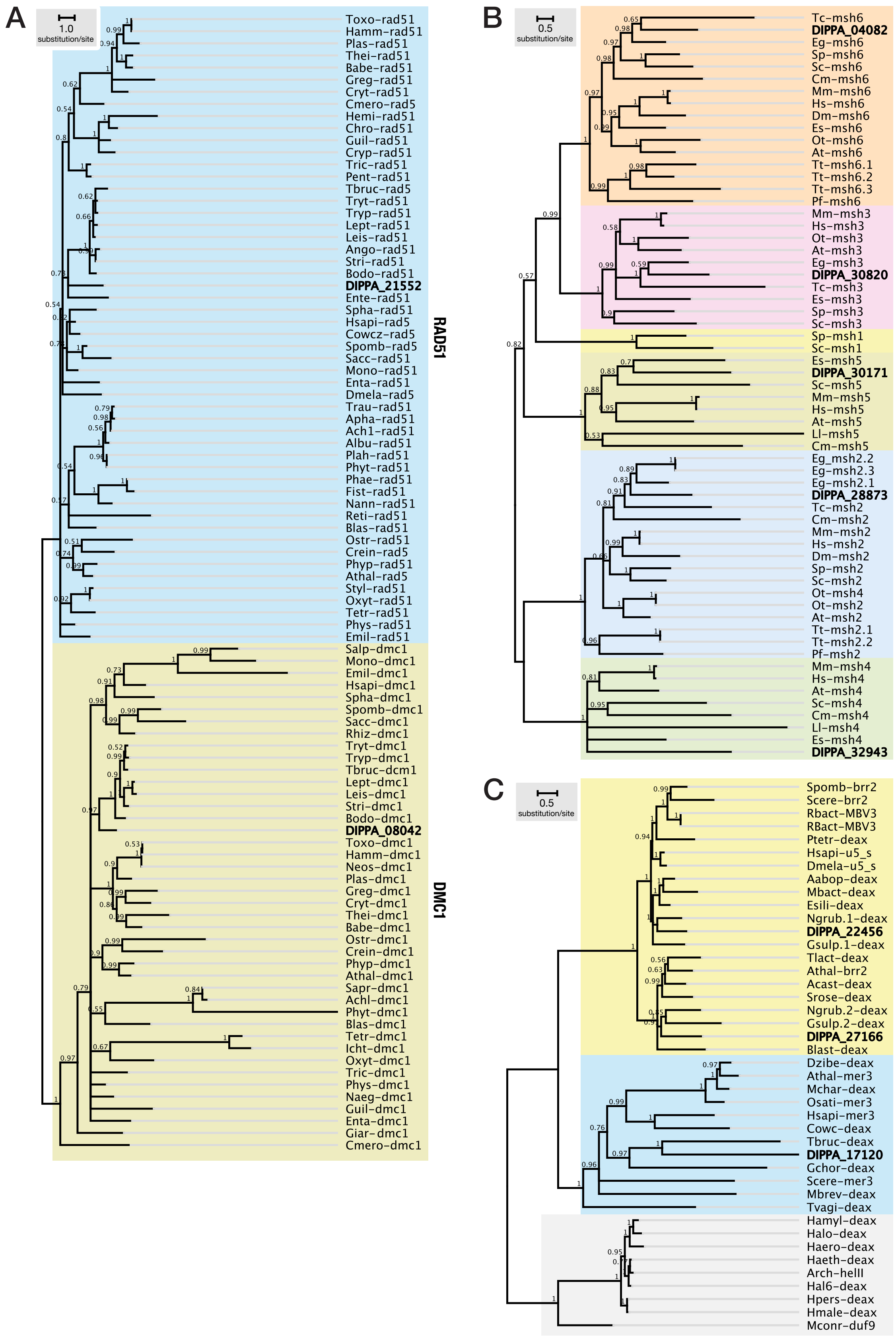
A
1.0
substitution/site
Toxo-rad51
Hamm-rad51
Plas-rad51
Thei-rad51
Babe-rad51
Greg-rad51
Cryt-rad51
Cmero-rad5
Hemi-rad51
Chro-rad51
Guil-rad51
Cryp-rad51
Tric-rad51
Pent-rad51
Tbruc-rad5
Tryt-rad51
Tryp-rad51
Lept-rad51
Leis-rad51
Ango-rad51
Stri-rad51
Bodo-rad51
DIPPA_21552
Ente-rad51
Spha-rad51
Hsapi-rad5
Cowcz-rad5
Spomb-rad5
Sacc-rad51
Mono-rad51
Enta-rad51
Dmela-rad5
Trau-rad51
Apha-rad51
Ach1-rad51
Albu-rad51
Plah-rad51
Phyt-rad51
Phae-rad51
Fist-rad51
Nann-rad51
Reti-rad51
Blas-rad51
Ostr-rad51
Crein-rad5
Phyp-rad51
Athal-rad5
Styl-rad51
Oxyt-rad51
Tetr-rad51
Phys-rad51
Emil-rad51
RAD51
Salp-dmc1
Mono-dmc1
Emil-dmc1
Hsapi-dmc1
Spha-dmc1
Spomb-dmc1
Sacc-dmc1
Rhiz-dmc1
Tryt-dmc1
Tryp-dmc1
Tbruc-dcm1
Lept-dmc1
Leis-dmc1
Stri-dmc1
Bodo-dmc1
DIPPA_08042
Toxo-dmc1
Hamm-dmc1
Neos-dmc1
Plas-dmc1
Greg-dmc1
Cryt-dmc1
Thei-dmc1
Babe-dmc1
Ostr-dmc1
Crein-dmc1
Phyp-dmc1
Athal-dmc1
Sapr-dmc1
Achl-dmc1
Phyt-dmc1
Blas-dmc1
Tetr-dmc1
Icht-dmc1
Oxyt-dmc1
Tric-dmc1
Phys-dmc1
Naeg-dmc1
Guil-dmc1
Enta-dmc1
Giar-dmc1
Cmero-dmc1
DMC1
B
0.5
substitution/site
Tc-msh6
DIPPA_04082
Eg-msh6
Sp-msh6
Sc-msh6
Cm-msh6
Mm-msh6
Hs-msh6
Dm-msh6
Es-msh6
Ot-msh6
At-msh6
Tt-msh6.1
Tt-msh6.2
Tt-msh6.3
Pf-msh6
MSH6
Mm-msh3
Hs-msh3
Ot-msh3
At-msh3
Eg-msh3
DIPPA_30820
Tc-msh3
Es-msh3
Sp-msh3
Sc-msh3
MSH3
Sp-msh1
Sc-msh1
MSH1
Es-msh5
DIPPA_30171
Sc-msh5
Mm-msh5
Hs-msh5
At-msh5
Ll-msh5
Cm-msh5
MSH5
Eg_msh2.2
Eg-msh2.3
Eg-msh2.1
DIPPA_28873
Tc-msh2
Cm-msh2
Mm-msh2
Hs-msh2
Dm-msh2
Sp-msh2
Sc-msh2
Ot-msh4
Ot-msh2
At-msh2
Tt-msh2.1
Tt-msh2.2
Pf-msh2
MSH2
Mm-msh4
Hs-msh4
At-msh4
Sc-msh4
Cm-msh4
Ll-msh4
Es-msh4
DIPPA_32943
MSH4
C
0.5
substitution/site
Spomb-brr2
Scere-brr2
Rbact-MBV3
RBact-MBV3
Ptetr-deax
Hsapi-u5_s
Dmela-u5_s
Aabop-deax
Mbact-deax
Esili-deax
Ngrub.1-deax
DIPPA_22456
Gsulp.1-deax
Tlact-deax
Athal-brr2
Acast-deax
Srose-deax
Ngrub.2-deax
Gsulp.2-deax
DIPPA_27166
Blast-deax
BRR2
Dzibe-deax
Athal-mer3
Mchar-deax
Osati-mer3
Hsapi-mer3
Cowc-deax
Tbruc-deax
DIPPA_17120
Gchor-deax
Scere-mer3
Mbrev-deax
Tvagi-deax
MER3
Hamyl-deax
Halo-deax
Haero-deax
Haeth-deax
Arch-helII
Hal6-deax
Hpers-deax
Hmale-deax
Mconr-duf9

Supplementary Figure S17. Phylogenetic analysis of proteins involved in meiosis. Posterior likelihood values of bipartitions are indicated. The function assignment of clades (vertical protein name) is based on that of the proteins in UniProtKB 'reviewed (SwissProt)' that are part of the corresponding clades. Branches of clades uniting kinetoplastid and *D. papillatum* sequences are colored in purple. Protein candidates from *D. papillatum* (DIPPA_) are shown in bold font. **A, DMC1 and RAD51.** Taxon names are abbreviated as follows: Ach1, Achl, *Achlya hypogyna;* Albu, *Albugo laibachii;* Ango, *Angomonas deanei;* Apha, *Aphanomyces astaci;* Athal, *Arabidopsis thaliana;* Babe, *Babesia microti;* Blas, *Blastocystis sp.;* Bodo, *Bodo saltans;* Chro, *Chroomonas mesostigmatica;* Cowcz, *Capsaspora owczarzaki;* Crein, *Chlamydomonas reinhardtii;* Cryp, *Cryptomonas paramecium;* Cryt, *Cryptosporidium andersoni;* Cmero, *Cyanidioschyzon merolae;* Dmela, *Drosophila melanogaster;* Emil, *Emiliania huxleyi;* Enta, *Entamoeba histolytica;* Ente, *Enterospora canceri;* Fist, *Fistulifera solaris;* Giar, *Giardia intestinalis;* Greg, *Gregarina niphandrodes;* Guil, *Guillardia theta;* Hamm, *Hammondia hammondi;* Hemi, *Hemiselmis andersenii;* Hsapi, *Homo sapiens;* Icht, *Ichthyophthirius multifiliis;* Leis, *Leishmania major;* Lept, *Leptomonas pyrrhocoris* and *seymouri;* Mono, *Monosiga brevicollis;* Nann, *Nannochloropsis gaditana;* Ostr, *Ostreococcus lucimarinus;* Oxyt, *Oxytricha trifallax;* Phae, *Phaeodactylum tricornutum;* Phyp, *Physcomitrella patens;* Phyt, *Phytophthora nicotianae;* Phys, *Physarum polycephalum;* Plah, *Plasmopara halstedii;* Reti, *Reticulomyxa filosa;* Rhiz, *Rhizophagus irregularis;* Sacc, *Saccharomyces cerevisiae;* Salp, *Salpingoeca rosetta;* Sapr, *Saprolegnia diclina;* Spha, *Sphaeroforma arctica;* Spomb, *Schizosaccharomyces pombe;* Tetr, *Tetrahymena thermophila;* Trau, *Thraustotheca clavata;* Stri, *Strigomonas culicis;* Styl, *Stylonychia lemnae;* Tryp, *Trypanosoma cruzi,* Tryt, *Trypanosoma theileri.* RAD51 in *Giardia* is probably a DMC1 ortholog. **B. MSH2 to MSH6**. Taxon names are abbreviated as follows: At, *Arabidopsis thaliana;* Cm, *Cyanidioschyzon merolae;* Dm, *Drosophila melanogaster;* Es, *Ectocarpus siliculosus;* Eg, *Euglena gracilis;* Hs, *Homo sapiens;* Ll, *Leishmania major;* Mm, *Mus musculus;* Ot, *Ostreococcus tauri;* Pf, *Plasmodium malariae and falciparum;* Sc, *Saccharomyces cerevisiae;* Tc, *Trypanosoma brucei, cruzi cruzi and theileri;* Tt, *Tetrahymena thermophila.* **C, MER3.** Taxon names are abbreviated as follows: Aabop, *Aureococcus anophagefferens;* Acast, *Acanthamoeba castellanii;* Athal, *rabidopsis thaliana;* Blast, *Blastocystis sp.;* Cowc, *Capsaspora owczarzaki;* Dzibe, *Durio zibethinus;* Esili, *Ectocarpus siliculosus;* Gchor, *Gracilariopsis chorda;* Csulp, *Galdieria sulphuraria;* Haero, *Haloplanus aerogenes;* Haeth, *Halorubrum aethiopicum;* Hal6, *Halorubrum sp.;* Hamyl, *Halogranum amylolyticum;* Hmale, *Halopenitus malekzadehii;* Hpers. *Halopenitus persicus;* Hsapi, *Homo sapiens;* Mbact, *Myxococcaceae bacterium;* Mbrev, *Monosiga brevicollis;* Hchar, *Momordica charantia;* Mconr, *Methanocella conradii;* Ngrub, *Naegleria gruberi;* Osati, *Oryza sativa;* Ptetr, *Paramecium tetraurelia;* RBact, Rbact, *Rickettsiales bacterium;* Scere, *Saccharomyces cerevisiae;* Spomb, *Schizosaccharomyces pombe;* Tbruc, *Trypanosoma brucei brucei;* Tlact, *Tieghemostelium lacteum;* Tvagi, *Trichomonas vaginalis.*

METHODS

Homology detection by pairwise sequence alignment and Hidden Markov model searches

We analyzed the proteins inferred from version 1.0 of the *D. papillatum* assembly. As query 'meiosis' proteins, we used those whose involvement in meiosis has been demonstrated (asterisks indicate proteins considered to be specific for meiosis): DMC1*, HOP1*, HOP2*, MER3, MND1*, MSH4, MSH5, REC114, REC8*, RED1, SPO11*, ZIP1, ZIP2, ZIP3, ZIP4, REC102, MEI4, MER3, MRE11, MSH1, MSH2, MSH3, MSH6, MUS81, RAD21, RAD50, RAD51, REC103, REC104, REC114, RPA1, RPA2, and XRS2. Sequences were downloaded from UniProtKB release 2021_3 (https://www.uniprot.org/help/uniprotkb). For similarity searches, we employed local blast and fasta as a rapid test. In addition, profile HMMs were generated for searches with HMMERsearch v3.3 from the HMMER suite (EDDY 2009) in the *Diplonema* inferred proteome. Further, we performed HMMscan of *D. papillatum* proteins against the PFAM profile database v35.0 (http://pfam.xfam.org) to test if candidates retrieved by similarity search do indeed contain the same conserved protein domains as the confirmed meiosis proteins.

Distinction of orthologs and paralogs

Meiosis proteins that are easily confused, notably DMC1 and RAD51, MSH1 to MSH6, RAD21 and REC8, and MER3 and other ATP-dependent DNA helicases, were distinguished *via* reciprocal blast and inspection of the protein-domain content and arrangement and *via* phylogenetic inference. For the latter analysis, proteins were pre-aligned with Muscle v3.8.155 and a profile HMM was built from the multiple alignment using HMMbuild with default parameters. The profile HMM served for building a final multiple protein alignment with HMMalign, default parameters, which then was used to construct a phylogenetic tree with Phylobayes v4.1c (LARTILLOT *et al.* 2009) using the options -cat -gtr -dgam 6 –dc, essentially as described previously (VALACH *et al.* 2017). The function assignment of clades as shown in the figures is based on that of the UniProt proteins labelled 'reviewed (SwissProt)' that make part of a clade.

AUTHOR CONTRIBUTIONS

Conceptualization, Data curation, Investigation, Formal analysis, Writing, original draft – G.B.; **Visualization –** M.V.; **Writing, review & editing –** all co-authors.

REFERENCES

Carpenter, M. L., Z. J. Assaf, S. Gourguechon and W. Z. Cande, 2012 Nuclear inheritance and genetic exchange without meiosis in the binucleate parasite *Giardia intestinalis*. J Cell Sci 125**:** 2523-2532.

Chi, J., F. Mahe, J. Loidl, J. Logsdon and M. Dunthorn, 2014 Meiosis gene inventory of four ciliates reveals the prevalence of a synaptonemal complex-independent crossover pathway. Mol Biol Evol 31**:** 660-672.

Ebenezer, T. E., M. Zoltner, A. Burrel, A. Nenarokova, A. M. G. N. Vanclová *et al.*, 2017 Unlocking the biological potential of *Euglena gracilis*: evolution, cell biology and significance to parasitism. bioRxiv**:** 228015.

Ebenezer, T. E., M. Zoltner, A. Burrell, A. Nenarokova, A. M. G. Novák Vanclová *et al.*, 2019 Transcriptome, proteome and draft genome of *Euglena gracilis*. BMC Biol 17**:** 11.

Eddy, S. R., 2009 A new generation of homology search tools based on probabilistic inference. Genome Inform 23**:** 205-211.

Heywood, P., 1976 Algal sexuality. Nature 259**:** 425.

Kohl, K. P., and J. Sekelsky, 2013 Meiotic and mitotic recombination in meiosis. Genetics 194**:** 327-334.

Lartillot, N., T. Lepage and S. Blanquart, 2009 PhyloBayes 3: a Bayesian software package for phylogenetic reconstruction and molecular dating. Bioinformatics 25**:** 2286-2288.

Leedale, G. F., 1967 Euglenida-euglenophyta. Annu Rev Microbiol 21**:** 31-48.

Maciver, S. K., Z. Koutsogiannis and A. de Obeso Fernández Del Valle, 2019 'Meiotic genes' are constitutively expressed in an asexual amoeba and are not necessarily involved in sexual reproduction. Biol Lett 15**:** 20180871.

Malik, S. B., A. W. Pightling, L. M. Stefaniak, A. M. Schurko and J. M. Logsdon, Jr., 2007 An expanded inventory of conserved meiotic genes provides evidence for sex in *Trichomonas vaginalis*. PLoS One 3**:** e2879.

Page, S. L., and R. S. Hawley, 2004 The genetics and molecular biology of the synaptonemal complex. Annu Rev Cell Dev Biol 20**:** 525-558.

Peacock, L., V. Ferris, R. Sharma, J. Sunter, M. Bailey *et al.*, 2011 Identification of the meiotic life cycle stage of *Trypanosoma brucei* in the tsetse fly. Proc Natl Acad Sci U S A 108**:** 3671-3676.

Pernin, P., A. Ataya and M. L. Cariou, 1992 Genetic structure of natural populations of the free-living amoeba, *Naegleria lovaniensis*. Evidence for sexual reproduction. Heredity (Edinb) 68**:** 173-181.

Sasanuma, H., H. Murakami, T. Fukuda, T. Shibata, A. Nicolas *et al.*, 2007 Meiotic association between Spo11 regulated by Rec102, Rec104 and Rec114. Nucleic Acids Res 35**:** 1119-1133.

Schweizer, J., H. Pospichal, G. Hide, N. Buchanan, A. Tait *et al.*, 1994 Analysis of a new genetic cross between two East African *Trypanosoma brucei* clones. Parasitology 109 (Pt 1)**:** 83-93.

Speijer, D., J. Lukeš and M. Eliáš, 2015 Sex is a ubiquitous, ancient, and inherent attribute of eukaryotic life. Proc Natl Acad Sci U S A 112**:** 8827-8834.

Valach, M., S. Moreira, S. Hoffmann, P. F. Stadler and G. Burger, 2017 Keeping it complicated: mitochondrial genome plasticity across diplonemids. Sci Rep 7**:** 14166.

Wassmann, K., 2013 Sister chromatid segregation in meiosis II: deprotection through phosphorylation. Cell Cycle 12**:** 1352-1359.

12. CAZyme-coding genes in *Diplonema papillatum*

INTRODUCTION

The complement of Carbohydrate-Active enzymes (CAZymes) of an organism can provide important clues about its metabolism and "lifestyle". CAZymes are highly diverse, with currently 173 Glycoside Hydrolase (GH) families, 42 Polysaccharide Lyase (PL) families, 20 Carbohydrate Esterase (CE) families, and 114 GlycosylTransferase (GT) families (DRULA *et al.* 2022) (http://www.cazy.org). CAZymes may also include, in addition to catalytic domains, Carbohydrate-Binding Modules (CBMs), which currently form 89 families. To get a glimpse at the nutrient resources and carbon storage of *D. papillatum*, we searched in its genome for homologs known from model organisms to be involved in the assembly and breakdown of glycans. We also compared the spectrum of CAZymes from *Diplonema* to that of two other euglenozoans: the free-living heterotroph *Bodo saltans* (Kinetoplastea) and the photoautotroph *Euglena gracilis* (Euglenida) (JACKSON *et al.* 2016; OPPERDOES *et al.* 2016; EBENEZER *et al.* 2019).

RESULTS

CAZyme complement of *D. papillatum*

The nuclear genome assembly of *D. papillatum* contains as many as 489 CAZymes for metabolizing diverse polysaccharides (**Supplementary Figure S18, Additional File 4** cazymeList). The repertoire includes 52 out of the 173 described GH families, 4 out of 20 CEs, 3 out of 42 PLs, 36 out of 114 GTs, and 11 out of 89 CBMs. Essentially all CAZyme genes we detected are transcribed, and most are likely translated, as peptides of 11% of the enzymes were identified in low-depth mass-spectrometry data (**Additional File 4** cazymeList).

Based on the repertoire of CAZyme domains, *D. papillatum* appears to specialize strongly in plant biomass degradation. Genes involved in the breakdown of **pectin** (consisting primarily of methyl-esterified α-1,4-galacturonic acid units) are the largest and most diverse group. The corresponding 122 genes (all expert-validated) belong to nine distinct CAZyme families (GH28, GH53, GH54, GH78, GH145, CE8, CE13, PL1, and PL4). Most of these enzyme families have undergone significant expansions, exemplified by the GH28 family, which comprises 25 members. In addition, a total of 82 proteins belong to CAZyme families that degrade the β-1,4-linked glucose polymer **cellulose** (GH5, GH6, GH7, and GH45, as well and the carbohydrate-binding module family CBM1) or **hemicelluloses**, i.e., hemicellulose side chains (GH51), xylan (GH10), xyloglucan (GH74 and GH115), and β-linked xylo- and gluco-oligosaccharides (GH3). Further, *Diplonema* encodes a large set of enzymes (27 proteins from the families GH79, GH154, GH47, GH76, and GH92) for the digestion of the **sulfated glucuronomannan** (α-1,3-mannan with β-D-glucuronic acid side chains), which is the main polysaccharide present in the cell walls of diatoms (LE COSTAOUËC *et al.* 2017).

The presence of genes encoding family members of GH55, GH72, GH128, GH152, GH81, and potentially GH16 in the *Diplonema* genome assembly suggests that this protist can digest the β-1,3-glucan **laminarin**, which is the storage polysaccharide of micro- and macro-algae such as haptophytes, stramenopiles, and diatoms (MICHEL *et al.* 2010), the latter organismal group being the most abundant primary producer in the oceans (FALKOWSKI *et al.* 1998). Laminarin is estimated to make up ~10% of the carbon produced globally (BECKER *et al.* 2020).

The *D. papillatum* genome assembly also revealed 18 genes encoding GH18, GH19, and GH20 enzymes, shown in model organisms to break down **chitin** (polymer of N-acetylglucosamine), together with several GH56 and GH114 enzymes, assumed to degrade glycoaminoglycans. Thus, *Diplonema* should be able to break down these polysaccharides usually encountered in animals and fungi.

Finally, the genome encodes genes for 90 additional CAZymes (about equally divided between GTs and CBMs), whose substrates cannot be inferred with confidence. The CBM13 family is particularly large (25 members), with ~75% of proteins containing multiple copies of this non-catalytic module. Seven CMB13 modules are appended to a PL4 domain likely to cleave pectin, suggesting that the PL4-CMB13 proteins in *Diplonema* are involved in pectin degradation.

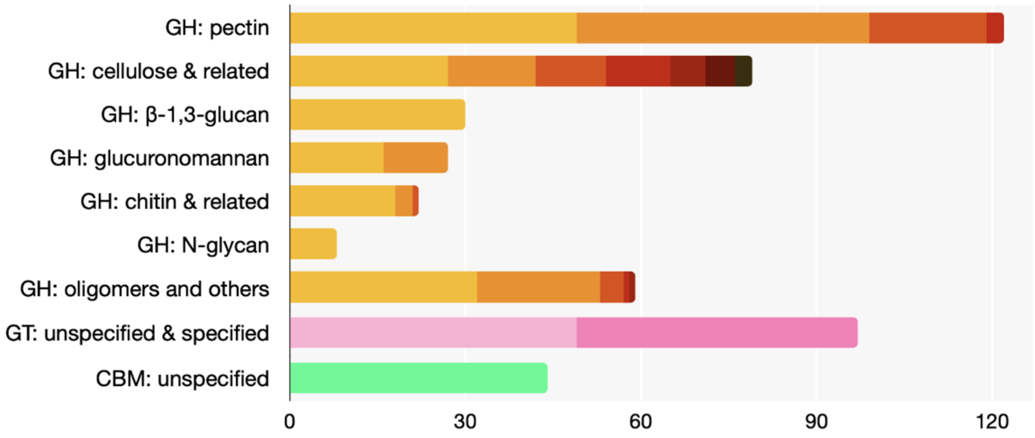

Supplementary Figure S18. Predicted carbohydrate-targeting proteins in *D. papillatum* containing at least one CAZyme domain. Proteins were grouped by their cognate substrate class. GH, glycosylhydrolases; GT, glycosyltransferases; CBM, carbohydrate-binding modules. The subdivision of the bars by different color shades represents the number of enzymes in the following subgroups. **GH: pectin** – pectin hydrolases, pectin lyases, pectin acetylesterases, and pectin methylesterases. **GH: cellulose & related** – cellulases, xylan-α-glucuronidases, xylan/cellulose and xylan/xyloglucan hydrolases, hemicellulases, β-glucan/β-xylan hydrolases, and β-mannanases. **GH: β-1,3-glucan** – no subgroups. **GH: glucuronomannan** – α-mannanases and β-glucuronidases. **GH: chitin & related** – chitinases, glycosaminoglycan, and glucosamine hydrolases. N-glycan – no subgroups. **GH: oligomers and others** – α-glycosidases (including α-mannosidases, α-fucosidases, α-glucosidases, and unspecified), β-glycosidases (including β-galactosidases, β-mannosidases, and unspecified), trehalases, an α-fucanase, and an invertase. **GT: unspecified & specified** – various substrates. **CBM: unspecified** – no subgroups (all substrates remain unspecified).

Comparative analyses of CAZymes profiles in Euglenozoa

The CAZyme portfolio of *D. papillatum* appears to lack enzymes from families the GH22, GH23, GH25, GH73, and CBM50 that target bacterial cell wall components, which points to a strictly eukaryotic diet. This finding contrasts with the gene repertoire and feeding behaviour of free-living members of *Diplonema*'s kinetoplastid sister group, represented by the marine and freshwater protist *B. saltans* (MITCHELL *et al.* 1988; OPPERDOES *et al.* 2016).

Further, the *Diplonema* genome assembly does not include any known α-glucan depolymerizing enzyme from families GH13 and GH77, which would suggest that this organism is unable to digest starch and glycogen, the carbon-storage compounds of Viridiplantae and Metazoa. However, we have demonstrated experimentally that *Diploma* readily utilizes amylopectin, a component of starch (see **Supplementary Information**: Section 13. Glycan and peptide assimilation by *Diplonema papillatum*). At the moment, it is impossible to identify the enzyme(s) responsible for this activity; they may be among the oligomer-targeting GH CAZymes or belong to novel families.

On the other hand, the absence of GH13 and GH77, together with the absence of the glycosyltransferase families GT3, GT5 and GT35, indicates that the form in which *Diplonema* stores carbon is not starch or glycogen. It is instead a β-1,3-glucan, as we found genes encoding members of the β-1,3-glucanase families GH55, GH72, GH128, GH152, and GH81. Supporting evidence for this form of carbon storage comes from the recent experimental identification of a β-1,3-glucan-containing polymer in *D. papillatum* (ŠKODOVÁ-SVERÁKOVÁ *et al.* 2020), a compound that has long been known from *Euglena*, called paramylon. *B. saltans* appears to store its carbon in the same form (reviewed in (RALTON *et al.* 2021)), which suggests that paramylon synthesis was already an attribute of the last common ancestor of Euglenozoa.

Lastly, we compared the distribution of CAZyme families in a systematic fashion across four euglenozoans, namely *D. papillatum*, its closest described relative *D. japonicum* (TASHYREVA *et al.* 2018), the free-living kinetoplastid *Bodo saltans* (JACKSON *et al.* 2016), and the euglenid *Euglena gracilis* (EBENEZER *et al.* 2019), as well as two representative, well-studied saprophytic fungi, *Trichoderma reesei* and *Phanerochaete chrysosporium* (**Supplementary Figure S19**). The *D. papillatum* genome encodes a number and diversity of GT families comparable to that of the above-listed species. However, it possesses a substantially larger and more diverse repertoire of carbohydrate-degrading enzymes, i.e., GH, CE, and PL families, as well as carbohydrate-recognizing proteins (CBM families). In particular, the 122-member pectin-targeting cohort of *D. papillatum* stands out, with the closest relative *D. japonicum* having only a quarter and the other four species less than 10% of that number. Enzymes and CBMs that target xylans and β-1,3-glucans are similarly enriched in *D. papillatum*. In contrast, *D. japonicum* has nearly twice as many chitin-metabolizing proteins (GH18, GH19, GH20) as *D. papillatum*.

The major limitation in comparing other diplonemids to *D. papillatum* is the lack of genomic information, with the proteome of *D. japonicum* being inferred from its reconstructed transcriptome alone. Although gene searches indicate that the coverage is good enough for retrieving well-conserved, low-expression genes (e.g., regulatory proteins involved in cell cycle, splicing, or endosome formation), the poly-A RNA-Seq sequencing depth is ~24 times lower than in our *D.*

papillatum dataset (~12 M vs. ~290 M read pairs). Thus, the *D. japonicum* CAZyme cohort inferred here is almost certainly underestimated. To get a more realistic measure of the scale of differences between the current and more genuine numbers, we compared the RNA-Seq coverage of transcripts between the two diplonemid species. Based on the bulk coverage difference and the number of reads mapped to individual CAZyme-encoding transcripts, we estimate that *D. japonicum* possesses 10–20% more CAZyme genes than the current tally (roughly equally distributed across functional categories).

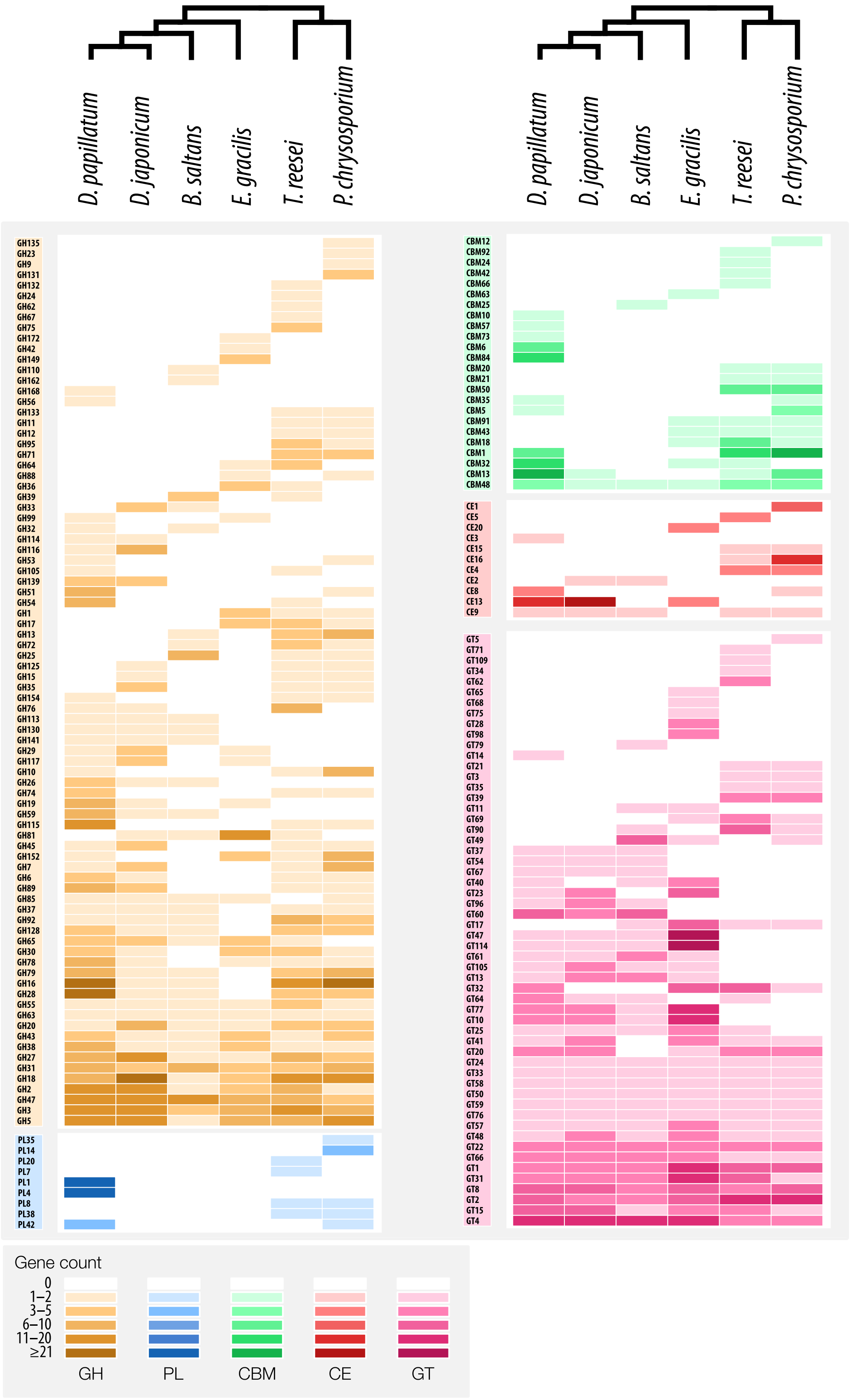

Supplementary Figure S19. Repertoire of carbohydrate-targeting proteins across euglenozoans and fungi. Proteins belonging to the CAZyme classes GH, PL, CBM, CE, and GT from four free-living euglenozoans (*D. papillatum*, *D. japonicum*, *Bodo saltans*, and *Euglena gracilis*) and two model fungi (*Trichoderma reesei* and *Phanerochaete chrysosporium*) are compiled. Rows correspond to individual CAZyme families within the classes GH, PL, CBM, CE, and GT. Heatmap shading indicates gene counts in each genome as detailed in the key (bottom). Within CAZyme classes, families are ordered from top to bottom based on the increasing number of members detected in the family.

DISCUSSION

In contrast to the nuclear genomes of parasitic and photoautotrophic euglenozoans, the genome of *D. papillatum* encodes an extremely high number of diverse CAZymes for the degradation of polysaccharides, comparable to the CAZyme complement of saprophytic fungi (ALMÁSI *et al.* 2019; DÍAZ-ESCANDÓN *et al.* 2022). With 52 GH, 3 PL, 11 CBM, and 36 GT families, the CAZyme repertoire in *D. papillatum* is as diverse as in the model saprophytic ascomycete *Trichoderma reesei* (55 GH, 4 PL, 12 CBM, and 32 GT families; (MARTINEZ *et al.* 2008)) or the basidiomycete *Phanerochaete chrysosporium* (50 GH, 5 PL, 11 CBM, and 28 GT families; (MARTINEZ *et al.* 2004)). The only category widely present in fungi but lacking in *Diplonema* are lytic polysaccharide monooxygenases (LPMOs) that oxidatively cleave polysaccharides.

This remarkably versatile CAZyme outfit of *D. papillatum* argues against the notion that this organism follows a parasitic lifestyle in its natural environment. Like other diplonemids (TRIEMER AND OTT 1990; PROKOPCHUK *et al.* 2022), *D. papillatum* takes up nutrients *via* phagocytosis and/or osmotrophy, indicating that it feeds on the biomass produced by photosynthetic microeukaryotes colonizing the surface layers of the oceans. For example, it has the potential to digest all carbohydrate polymers, including glucuronomannan, chitin, and pectin which are typically found in the most abundant marine primary producers, the diatoms. In particular, the expansion of pectin-degrading families suggests that pectin has, over the course of evolution, become a preferred food for *D. papillatum*. In addition, many marine eukaryotes, including phototrophic diatoms (CHEN AND THORNTON 2015) and mixotrophic dinoflagellates (LARSSON *et al.* 2022), produce extracellular polymers such as the transparent exopolymer particles (TEPs). The major components of TEPs are yet uncharacterized, complex mixtures of acidic polysaccharides (reviewed in (PASSOW 2002; DECHO AND GUTIERREZ 2017)). It is conceivable that the enzyme families of *D. papillatum* predicted to degrade pectin (which is acidic as well) are also capable of breaking down TEPs. Together with the extensive repertoire of proteins with unknown carbohydrate targets, the substrate range of *Diplonema* is probably much broader than we currently appreciate.

METHODS

Generation of proteomes

The section **Supplementary Information**: Section 2. Assembly and annotation of the nuclear genome and transcriptome of *Diplonema papillatum* describes how the 'submission' proteome of *D. papillatum* was prepared. For additional diplonemids, the proteomes were generated as follows. First, publicly available poly-A RNA-Seq reads (KAUR *et al.* 2020) were cleaned using cutadapt v1.16 (http://journal.embnet.org/index.php/embnetjournal/article/view/200) and assembled using Trinity v2.4.0 (GRABHERR *et al.* 2011) with default parameters. We removed from the transcriptomes isoforms and incomplete reverse-complementary transcript fragments by two rounds of clustering using CD-HIT-EST v4.6 (FU *et al.* 2012). In the first round, all transcripts with 100% identity were clustered, and TransDecoder v5.5.0 (https://github.com/TransDecoder/TransDecoder) was used to infer coding sequences over 150 bp (i.e., length >50 amino acid residues), employing the standard genetic code. In the second round, the predicted coding sequences were clustered with CD-HIT-EST in local mode (option '-G 0') at 99% sequence identity and local overlap over 90% length between the cluster representative and embedded cluster members. Finally, PRINSEQ-lite v0.20.3 (SCHMIEDER AND EDWARDS 2011) was used to select only coding sequences longer than 450 bp (corresponding to proteins >150 aa) and protein sequences were regenerated using gotranseq v0.3.2 (https://github.com/feliixx/gotranseq) (standard genetic code, forward frame 1).

Protein identification and classification

Detection and assignment of CAZyme families in the inferred proteome of *D. papillatum* and the other seven diplonemids was performed using the methodology applied for the daily updates of the CAZy database (www.cazy.org), including expert validation (CANTAREL *et al.* 2009; LOMBARD *et al.* 2014).

Analyses of mass spectrometry data

We reanalyzed mass spectrometry data (ProteomeXchange ID: PXD025411) generated in the context of a prior study (ŠKODOVÁ-SVERÁKOVÁ *et al.* 2021). Raw data were first converted from the Thermo RAW format to mzML using ThermoRawFileParser v1.3.4 (HULSTAERT *et al.* 2020). Peptide searches were performed using MSFragger v3.5 (KONG *et al.* 2017), followed by filtering and scoring by Philosopher v4.4.0 (DA VEIGA LEPREVOST *et al.* 2020) and intensity-based quantification by IonQuant v1.8.0 (YU *et al.* 2020). Briefly, we searched for peptide-spectrum matches (PSMs) in a custom database of *D. papillatum* nuclear and mitochondrial proteins supplemented with frequent contaminants. Precursor and fragment mass tolerance were set to 20 ppm. We allowed up to two missed cleavage sites per protein for trypsin digestion. Carbamidomethylation of cysteine was specified as a fixed modification. Methionine oxidation, N-terminal protein acetylation, serine and threonine phosphorylation, and conversion of glutamine and glutamate at peptide N-termini to

pyrrolidone-carboxylic acid (PCA) were specified as variable modifications (up to three per peptide). Minimum and maximum peptide sizes were set to 700 and 5,000 Da, respectively. False discovery rates (FDR) for PSM and protein-identification probability were determined by the target-reversed decoy approach and set to 1%. Data processing and normalization by IonQuant were done for all ions using the topN strategy but without the match-between-runs option.

AUTHOR CONTRIBUTIONS

Conceptualization, Data curation, Formal analysis, Investigation, Writing, original draft – B.H., P.L., M.V.; **Visualization –** M.V.; **Writing, review & editing –** all authors.

REFERENCES

Almási, É., N. Sahu, K. Krizsán, B. Bálint, G. M. Kovács *et al.*, 2019 Comparative genomics reveals unique wood-decay strategies and fruiting body development in the Schizophyllaceae. New Phytol 224**:** 902-915.

Becker, S., J. Tebben, S. Coffinet, K. Wiltshire, M. H. Iversen *et al.*, 2020 Laminarin is a major molecule in the marine carbon cycle. Proc Natl Acad Sci U S A 117**:** 6599-6607.

Cantarel, B. L., P. M. Coutinho, C. Rancurel, T. Bernard, V. Lombard *et al.*, 2009 The Carbohydrate-Active EnZymes database (CAZy): an expert resource for Glycogenomics. Nucleic Acids Res 37**:** D233-238.

Chen, J., and D. C. Thornton, 2015 Transparent exopolymer particle production and aggregation by a marine planktonic diatom (*Thalassiosira weissflogii*) at different growth rates. J Phycol 51**:** 381-393.

da Veiga Leprevost, F., S. E. Haynes, D. M. Avtonomov, H. Y. Chang, A. K. Shanmugam *et al.*, 2020 Philosopher: a versatile toolkit for shotgun proteomics data analysis. Nat Methods 17**:** 869-870.

Decho, A. W., and T. Gutierrez, 2017 Microbial Extracellular Polymeric Substances (EPSs) in ocean systems. Front Microbiol 8**:** 922.

Díaz-Escandón D., G. Tagirdzhanova, D. Vanderpool, C. C. G. Allen, A. Aptroot *et al.* 2022 Genome-level analyses resolve an ancient lineage of symbiotic ascomycetes. Curr Biol. 32**:** 5209-5218.

Drula, E., M. L. Garron, S. Dogan, V. Lombard, B. Henrissat *et al.*, 2022 The carbohydrate-active enzyme database: functions and literature. Nucleic Acids Res 50**:** D571-d577.

Ebenezer, T. E., M. Zoltner, A. Burrell, A. Nenarokova, A. M. G. Novák Vanclová *et al.*, 2019 Transcriptome, proteome and draft genome of *Euglena gracilis*. BMC Biol 17**:** 11.

Falkowski, P. G., R. T. Barber and V. V. Smetacek, 1998 Biogeochemical controls and feedbacks on ocean primary production. Science 281**:** 200-207.

Fu, L., B. Niu, Z. Zhu, S. Wu and W. Li, 2012 CD-HIT: accelerated for clustering the next-generation sequencing data. Bioinformatics 28**:** 3150-3152.

Grabherr, M. G., B. J. Haas, M. Yassour, J. Z. Levin, D. A. Thompson *et al.*, 2011 Full-length transcriptome assembly from RNA-Seq data without a reference genome. Nat Biotechnol 29**:** 644-652.

Hulstaert, N., J. Shofstahl, T. Sachsenberg, M. Walzer, H. Barsnes *et al.*, 2020 ThermoRawFileParser: modular, scalable, and cross-platform RAW file conversion. J Proteome Res 19**:** 537-542.

Jackson, A. P., T. D. Otto, M. Aslett, S. D. Armstrong, F. Bringaud *et al.*, 2016 Kinetoplastid phylogenomics reveals the evolutionary innovations associated with the origins of parasitism. Current Biology 26**:** 161-172.

Kaur, B., K. Záhonová, M. Valach, D. Faktorová, G. Prokopchuk *et al.*, 2020 Gene fragmentation and RNA editing without borders: eccentric mitochondrial genomes of diplonemids. Nucleic Acids Res 48**:** 2694-2708.

Kong, A. T., F. V. Leprevost, D. M. Avtonomov, D. Mellacheruvu and A. I. Nesvizhskii, 2017 MSFragger: ultrafast and comprehensive peptide identification in mass spectrometry-based proteomics. Nat Methods 14**:** 513-520.

Larsson, M. E., A. R. Bramucci, S. Collins, G. Hallegraeff, T. Kahlke *et al.*, 2022 Mucospheres produced by a mixotrophic protist impact ocean carbon cycling. Nat Commun 13**:** 1301.

Le Costaouëc, T., C. Unamunzaga, L. Mantecon and W. Helbert, 2017 New structural insights into the cell-wall polysaccharide of the diatom *Phaeodactylum tricornutum*. Algal Res 26**:** 172-179.

Lombard, V., H. Golaconda Ramulu, E. Drula, P. M. Coutinho and B. Henrissat, 2014 The carbohydrate-active enzymes database (CAZy) in 2013. Nucleic Acids Res 42**:** D490-495.

Martinez, D., R. M. Berka, B. Henrissat, M. Saloheimo, M. Arvas *et al.*, 2008 Genome sequencing and analysis of the biomass-degrading fungus *Trichoderma reesei* (syn. *Hypocrea jecorina*). Nat Biotechnol 26**:** 553-560.

Martinez, D., L. F. Larrondo, N. Putnam, M. D. Gelpke, K. Huang *et al.*, 2004 Genome sequence of the lignocellulose degrading fungus *Phanerochaete chrysosporium* strain RP78. Nat Biotechnol 22**:** 695-700.

Michel, G., T. Tonon, D. Scornet, J. M. Cock and B. Kloareg, 2010 Central and storage carbon metabolism of the brown alga *Ectocarpus siliculosus*: insights into the origin and evolution of storage carbohydrates in eukaryotes. New Phytol 188**:** 67-81.

Mitchell, G. C., J. H. Baker and M. A. Sleigh, 1988 Feeding of a freshwater flagellate, *Bodo saltans,* on diverse bacteria. J Protozool 35**:** 219-222.

Opperdoes, F. R., A. Butenko, P. Flegontov, V. Yurchenko and J. Lukeš, 2016 Comparative metabolism of free-living *Bodo saltans* and parasitic trypanosomatids. J Eukaryot Microbiol 63**:** 657-678.

Passow, U., 2002 Transparent exopolymer particles (TEP) in aquatic environments. Progress Oceanogr 55**:** 287-333.

Prokopchuk, G., T. Korytář, V. Juricová, J. Majstorović, A. Horák *et al.*, 2022 Trophic flexibility of marine diplonemids - switching from osmotrophy to bacterivory. ISME J 16**:** 1409-1419.

Ralton, J. E., M. F. Sernee and M. J. McConville, 2021 Evolution and function of carbohydrate reserve biosynthesis in parasitic protists. Trends Parasitol 37**:** 988-1001.

Schmieder, R., and R. Edwards, 2011 Quality control and preprocessing of metagenomic datasets. Bioinformatics 27**:** 863-864.

Škodová-Sveráková, I., G. Prokopchuk, P. Peña-Diaz, K. Záhonová, M. Moos *et al.*, 2020 Unique dynamics of paramylon storage in the marine euglenozoan *Diplonema papillatum*. Protist 171**:** 125717.

Škodová-Sveráková, I., K. Záhonová, V. Juricová, M. Danchenko, M. Moos *et al.*, 2021 Highly flexible metabolism of the marine euglenozoan protist *Diplonema papillatum*. BMC Biol 19**:** 251.

Tashyreva, D., G. Prokopchuk, J. Votýpka, A. Yabuki, A. Horák *et al.*, 2018 Life cycle, ultrastructure, and phylogeny of new diplonemids and their endosymbiotic bacteria. MBio 9**:** e02447-02417.

Triemer, R. E., and D. W. Ott, 1990 Ultrastructure of *Diplonema ambulator* Larsen & Patterson (Euglenozoa) and its relationship to *Isonema*. Eur J Protistol 25**:** 316-320.

Yu, F., S. E. Haynes, G. C. Teo, D. M. Avtonomov, D. A. Polasky *et al.*, 2020 Fast quantitative analysis of timsTOF PASEF data with MSFragger and IonQuant. Mol Cell Proteomics 19**:** 1575-1585.

13. Glycan and peptide assimilation by *Diplonema papillatum*

INTRODUCTION

The metabolism of *D. papillatum* has been examined experimentally regarding glycolysis and gluconeogenesis (MORALES *et al.* 2016), respiratory pathways (VALACH *et al.* 2018), carbon storage (ŠKODOVÁ-SVERÁKOVÁ *et al.* 2020), and adaptation to hypoxia (ŠKODOVÁ-SVERÁKOVÁ *et al.* 2021). A surprising result from this research was that *Diplonema* does not import glucose in any significant amount but instead takes up and preferentially metabolizes amino acids. This finding led to the conclusion that in its natural habitat, the primary energy source of this organism is not carbohydrates, as in the majority of heterotrophic eukaryotes, but rather poly- and oligo-peptides (MORALES *et al.* 2016; ŠKODOVÁ-SVERÁKOVÁ *et al.* 2020). The requirement of serum in the growth medium together with the prediction of numerous peptidase-encoding genes in the nuclear genome assembly (see the main text and **Supplementary Information**: Section 14. Secretome prediction), have corroborated this view.

The large ensemble of carbohydrate-metabolizing genes (CAZymes) detected in the inferred *Diplonema* proteome was, therefore, completely unexpected (see **Supplementary Information**: Section 12. CAZyme-coding genes in *Diplonema papillatum* and Section 14. Secretome prediction). About 70% of CAZyme genes carry domains that allow us to predict the likely substrates of these enzymes. A quarter of all CAZymes are homologs of enzymes degrading pectin, a heteropolysaccharide composed mainly of α–1,4-linked galacturonic acid (mostly methyl-esterified at its carboxyl groups) but also containing rhamnose, galactose, xylose, and arabinose units (CAFFALL AND MOHNEN 2009). Additional polysaccharides that *Diplonema* is presumably able to metabolize are cellulose (a β–1,4 glucan), hemi-celluloses (xylans, xylo-glucans), β–1,3 glucans (callose, laminarin, paramylon), glucuronomannans, mannans, and chitin, i.e., most cell-wall building blocks of diverse marine algae and plants.

Since many of the polysaccharide-degrading enzymes from *Diplonema* are predicted to be secreted outside the cell (see **Supplementary Information**: Section 14. Secretome prediction), we posit that this protist, in its natural habitat, uses its large enzymatic arsenal to break down the cell wall of microeukaryotic prey (for cell-wall composition, see (MYKLESTAD AND GRANUM 2009; SCHOLZ *et al.* 2014; LE COSTAOUËC *et al.* 2017; RAIMUNDO *et al.* 2017)). Such behaviour may serve different 'purposes': either to gain access to proteins inside the prey's cell or to exploit the prey's polycarbohydrates in the cell wall and intracellular storage (or both). For example, starch is a glycan stored by various marine plants and green algae (BALL *et al.* 2011), while laminarin and its variants are accumulated by diatoms, brown algae, and other photosynthetic microeukaryotes (CHABI *et al.* 2021).

We reasoned that if the only purpose of cell-wall degradation is to feed on the cytoplasm of the prey, then *D. papillatum* would not be able to use carbohydrates as the sole carbon source. To test this hypothesis, we performed growth experiments in media of various compositions, described below.

RESULTS

Nutrient assimilation experiments

D. papillatum was cultured in liquid medium supplemented with diverse carbon sources, including the cell-wall component pectin and the storage compound amylopectin; the latter is a polymer of α–1,4 and α–1,6 glucose units and constitutes the water-soluble component of starch. Compared to the control on medium without any carbon source, we observed that mono- and disaccharides, and polyols such as sorbitol, only poorly supported cell growth (**Supplementary Figure S20**). Cell counts were slightly higher in medium containing free amino acids, especially after eight days; we also noted that under these conditions, cells had large vacuoles. In contrast, when cultivated in the presence of serum, pectin or amylopectin, cell counts were ~2 times higher than of the control; **Supplementary Figure S20**). Cultures growing on tryptone and yeast extract achieved the highest titers (~3 to 4-fold increase; **Supplementary Figure S20**). The observed low cell proliferation on glycans compared to tryptone was likely due to the exhaustion of the internal nitrogen reserve that in *Diplonema* consists of free amino acids such as β-alanine and glutamate (MORALES *et al.* 2016; ŠKODOVÁ-SVERÁKOVÁ *et al.* 2021). We also observed that ammonium sulfate, which is added as a nitrogen source in synthetic media of numerous microbes (e.g., yeast), did not significantly influence the growth of *D. papillatum* (at 0.1% w/v) or even strongly inhibited it (at 0.5% w/v). Apparently, *Diplonema* is not able to use external ammonia as a nitrogen source.

Interestingly, *D. papillatum* cells grown on pectin and, particularly, amylopectin as a sole carbon source were significantly larger, and those from tryptone cultures considerably smaller, compared to cells cultivated in the standard serum-supplemented medium (**Supplementary Figure S21A–D**). However, the total biomass was quite similar because in tryptone-grown cultures, the number of cells was higher, whereas in pectin or amylopectin media, the cells had a lower titer (**Supplementary Figure S21E**).

The cytoplasm of large glycan-fed cells contained conspicuous granules readily visible by light microscopy (**Supplementary Figure S21**). The best candidates for the compound stored in these granules are (i) paramylon (a β-1,3-

glucan), which *Diplonema* can synthesize (ŠKODOVÁ-SVERÁKOVÁ *et al.* 2020), or alternatively (ii) lipids—known to be produced by many microeukaryotes and bacteria under nitrogen-limiting conditions (and considered for large-scale biotechnological production of biofuels (SUBRAMANIAM *et al.* 2010)). In contrast to glycans, lipids are readily detected by incubating cells with the Nile Red dye that stains specifically neutral lipids. Inspection of Nile Red-treated *D. papillatum* cells by fluorescence microscopy demonstrated that the granules formed by glycan-grown cells indeed represented lipid droplets (**Supplementary Figure S21F,G**).

Finally, a startling observation was that similar titers and cell sizes were obtained when *Diplonema* was cultivated on a mix of equal amounts of tryptone and a glycan (either pectin or amylopectin), compared with cultures on tryptone alone (**Supplementary Figure S20**). One possible explanation is that *Diplonema* prefers oligo-peptides over polysaccharides in situations where both substrates are readily available. It would be interesting to examine by metabolic labelling to what extent *D. papillatum* uses these substrates as an energy source or building blocks.

Expression of metabolic enzymes

Our RNA-Seq data indicated that essentially all genes encoding metabolic proteins produced transcripts. To test if these enzymes inferred from the *Diplonema* genome and transcriptome assemblies were indeed translated, we analyzed raw mass-spectrometry data, produced in another context, of total cellular proteins from *Diplonema* (ŠKODOVÁ-SVERÁKOVÁ *et al.* 2021). In that study, cells were cultivated in a standard medium (containing serum as a carbon and nitrogen source) or in a tryptone-supplemented medium, both in the presence or absence of oxygen. Our analysis shows that ~7% of all predicted CAZymes were expressed as proteins, which appears low, but can be explained by the moderate depth of the mass-spectrometry data (see **Supplementary Information**: Section 12. CAZyme-coding genes in *Diplonema papillatum*). More importantly, many CAZymes displayed similar levels under all tested conditions. Although polysaccharides were absent from the culture medium, we detected nine pectin-, seven cellulose-, and five glucuronomannan-degrading proteins, half of them at levels comparable to mitochondrial TCA-cycle enzymes (see **Supplementary Information**: Section 12. CAZyme-coding genes in *Diplonema papillatum*).

DISCUSSION

Our growth experiments with *D. papillatum* described above lead to two main conclusions. First, this organism prefers polymers (peptide- or carbohydrate-based) over their monomeric constituents, and second, it can utilize a carbohydrate-rich diet as efficiently as a peptide-rich one. Therefore, in its natural habitat, *Diplonema* most likely feeds on essentially all cellular components of its presumed eukaryotic prey, from cell wall to cytoplasm to storage glycans. The results obtained from the growth experiments are consistent with the large and diverse array of carbohydrate- and peptide-metabolizing enzymes inferred from the nuclear genome sequence, testifying to the extraordinary metabolic versatility of *Diplonema*.

β-1,3-glucans are abundant in marine habitats, serving as a cell-wall component (callose) in diatoms, haptophytes, and macroscopic brown algae, but also as a carbon storage material (with β–1,6 branching, laminarin) (RAIMUNDO *et al.* 2017; MYKLESTAD AND GRANUM 2009). One could argue that the presence of β-1,3-glucan-metabolizing enzymes in *Diplonema* simply reflects that paramylon is its carbon-storage compound rather than an ability to degrade the cell walls of prey (see **Supplementary Information**: Section 12. CAZyme-coding genes in *Diplonema papillatum*). However, multiple members of the corresponding CAZyme families from *D. papillatum* were predicted to be secreted outside the cell (see **Supplementary Information**: Section 14. Secretome prediction), which corroborates their role in digesting prey and in utilizing various β-1,3-glucans as an energy source.

Our finding that *D. papillatum* can feed on carbohydrates is at odds with previous studies, which reported that cells took up only negligible amounts of glucose (MORALES *et al.* 2016; ŠKODOVÁ-SVERÁKOVÁ *et al.* 2020; ŠKODOVÁ-SVERÁKOVÁ *et al.* 2021). In the corresponding experiments, this carbohydrate was added to the standard medium consisting of sea salts, 1% horse serum, and optionally 0.1% tryptone. The noted contradiction between the observed marginal glucose import and the presence of a gene encoding a putative sodium/glucose co-transporter (DIPPA_28570) led to the hypothesis that this transporter acted predominantly in intracellular transport (ŠKODOVÁ-SVERÁKOVÁ *et al.* 2021). While our growth experiments confirm that *Diplonema* does not readily feed on mono- and di-saccharides, we argue that the import rate of simple saccharides cannot be extrapolated to that of oligomers. Oligosaccharide importers are fairly frequent in many microbes, with some outer membrane transporters accommodating solutes of up to 2.5 kDa, corresponding to an atomic mass of 12-mers (FERREIRA *et al.* 2017; JECKELMANN AND ERNI 2020). Such transporters belong mainly to the ABC and MFS families. Since the genome of *D. papillatum* encodes ~100 members of each family (many carrying sugar-binding domains, see also **Supplementary Table S20**), this protist most likely imports carbohydrate oligomers. Lastly, we noted that the previous carbohydrate-assimilation experiments were conducted over a duration that might have been too short to detect the full extent of nutrient import (only 8 to 24h compared to multiple days in the study presented here). This aspect should be taken into account in the design of future experiments.

Another surprising finding was that *D. papillatum* can feed efficiently on amylopectin, although a gene encoding an amylase could not be detected in the genome assembly (see **Supplementary Information**: Section 12. CAZyme-coding genes in *Diplonema papillatum*). It is improbable that the amylopectin added to the growth medium was contaminated with proteins and/or lipids, because potato starch contains <0.1% proteins and lipids (per dry matter), and the amylopectin purified from starch contains even less of these compounds (SWINKELS 1985; DHITAL *et al.* 2011). In addition, such minute contaminants cannot explain the massive accumulation of carbon in the form of lipid droplets in *Diplonema* cells and the high biomass generated during growth on amylopectin. Therefore, we infer that *D. papillatum* can break down amylopectin, and that the genes encoding the corresponding enzymes are present in the genome but have not been recognized. The posited 'incognito' genes are likely among the 150 CAZymes of unknown substrate specificity detected in the *Diplonema* nuclear genome. Identifying these orphan genes might well unveil a new class of starch-degrading enzymes.

Finally, by analyzing protein mass spectrometry data, we observed that several CAZyme-encoding genes predicted by functional genome annotation of the *D. papillatum* nuclear genome sequence are highly expressed despite the absence of their glycan substrates in the culture medium. Constitutive expression or induced co-expression (e.g., during phagocytosis) of diverse nutrient-degrading genes would allow *Diplonema* to adapt rapidly to changes in the food landscape. In the same vein, *D. papillatum* also appears to barely modulate the expression of most enzymes upon changing oxygen levels in the medium (ŠKODOVÁ-SVERÁKOVÁ *et al.* 2021). While expressing genes that target substances absent at a given moment and place might seem wasteful, it is also an excellent strategy to be prepared for rapid shifts in marine environmental conditions. Thus, *D. papillatum* stands out for its highly versatile metabolic capability and its potential for rapid metabolic switching.

METHODS

Strain and regular culture conditions

Diplonema papillatum (ATCC 50162) was originally obtained from the American Type Culture Collection (ATCC). As described earlier (VALACH *et al.* 2018), the strain was cultivated axenically without shaking at 15–22 °C in ocean salt medium (OS) containing 33 g/L Instant Ocean Sea Salt (*Instant Ocean*) supplemented with 1% (v/v) horse serum (*Wisent*). For extended cultivations, chloramphenicol (*Sigma-Aldrich*) was added to the medium at 40 mg/L to prevent bacterial contamination.

Nutrient assimilation experiments

Most nutrient assimilation experiments were performed in multi-well plates, with each of the 48 wells holding 625 µL. Cells (~3×10^5 per well) from a preculture in the standard medium were extensively washed, then starved for 2–6 hr, and subsequently transferred to the new OS medium containing vitamins (biotin [1 µg/1 L], cyanocobalamin [2 µg/1 L], thiamine-HCl [20 µg/1 L]; *Thermo Scientific*), 20 mM HEPES-KOH, pH7.6 (*Bioshop Canada*), optionally horse serum to 0.2%. The following substrates served as primary energy sources: citrus pectin (purchased from two manufacturers: *TCI America* and *Pomona's Pectin/Green Link*); amylopectin (produced from waxy corn, *TCI America*, and from potato starch, *Fluka/Sigma-Aldrich*); mono- and disaccharides (glucose, galactose, maltose, saccharose; *Bioshop Canada*); sugar-derived poly-alcohols (polyols include mannitol, sorbitol; *Bioshop Canada*); yeast extract (a complex mixture of carbohydrates, proteins, ions, and cofactors; *BioBasic*); tryptone (trypsin-digested casein; *Difco/ThermoFisher*); and free amino acids (glycine, glutamate, glutamine; *Bioshop Canada*). Each substrate was added at the same weight-to-volume ratio (0.1% w/v) to provide a similar carbon content. Cells were monitored for up to eight days (~200 hr). Six biological replicates from three independent inocula were carried out. To determine the biomass, cells from the late exponential/early stationary phase were inoculated at a titer of ~5×10^5 per mL into 62.5 mL medium containing the substrate to be tested (horse serum, tryptone, pectin, or amylopectin). After 150–160 hr (~6–7 days), cells were counted under the microscope in triplicate, and the biomass was determined to calculate the wet weight per 10^8 cells. Four independent biological replicates were made.

Microscopy

For conventional light microscopy, cells harvested from 6 to 7 day-old cultures were washed twice in plain OS medium (2,000×*g*, 3 min, 4 °C) and resuspended in the same medium or in an isotonic SoH buffer (1.2 M sorbitol, 20 mM HEPES, pH7.5). For the visualization of lipid droplets by fluorescence microscopy, cells were harvested after six days of cultivation, washed twice in plain OS medium, and then incubated (45 min, 22 °C) in 0.5× SoH buffer plus 16 g/L OS, to which Nile Red (AdipoRed staining reagent, *Lonza*) was added at a ratio of 1:20 (vol:vol). The hypotonicity of this buffer increased the assimilation of the dye and ensured a more even staining throughout the cell population. Cells, which stayed alive during the treatment, adhered more than usual to the slides. The slowed-down movement of the cells allowed visualization of sub-cellular structures at a higher resolution. Mounted cells were examined using an Eclipse Ts2R microscope (*Nikon*), and images were taken using a DS-Fi3 camera, analyzed by the NIS Elements BR software (*Nikon*), and post-processed with the Affinity Photo software v1.10.4 (*Serif*).

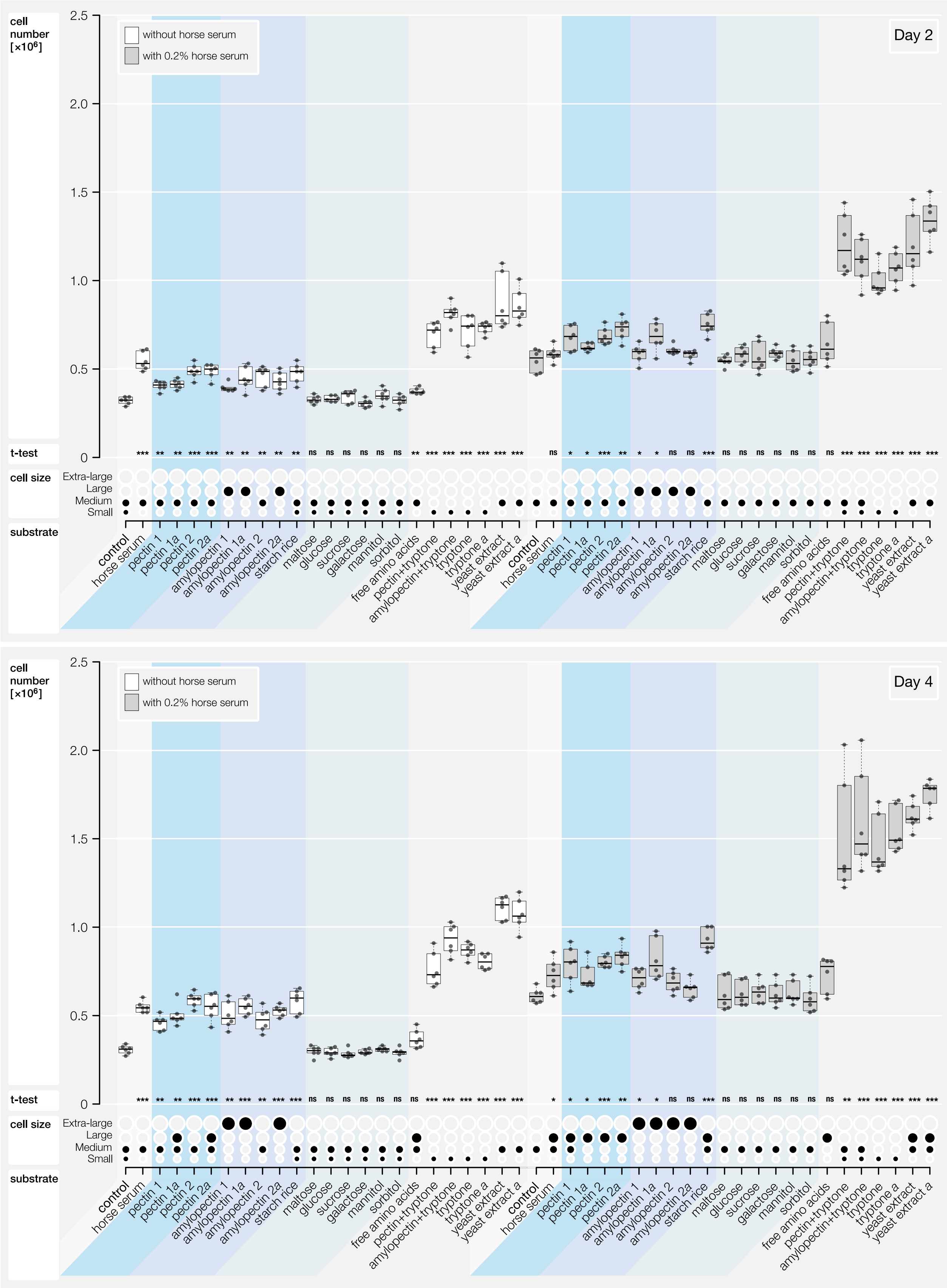
cell number [×10⁶]
2.5
2.0
1.5
1.0
0.5
0
without horse serum
with 0.2% horse serum
Day 2
t-test
cell size
Extra-large
Large
Medium
Small
substrate
control
horse serum
pectin 1
pectin 1a
pectin 2
pectin 2a
amylopectin 1
amylopectin 1a
amylopectin 2
amylopectin 2a
starch rice
maltose
glucose
sucrose
galactose
mannitol
sorbitol
free amino acids
pectin+tryptone
amylopectin+tryptone
tryptone
tryptone a
yeast extract
yeast extract a
Day 4

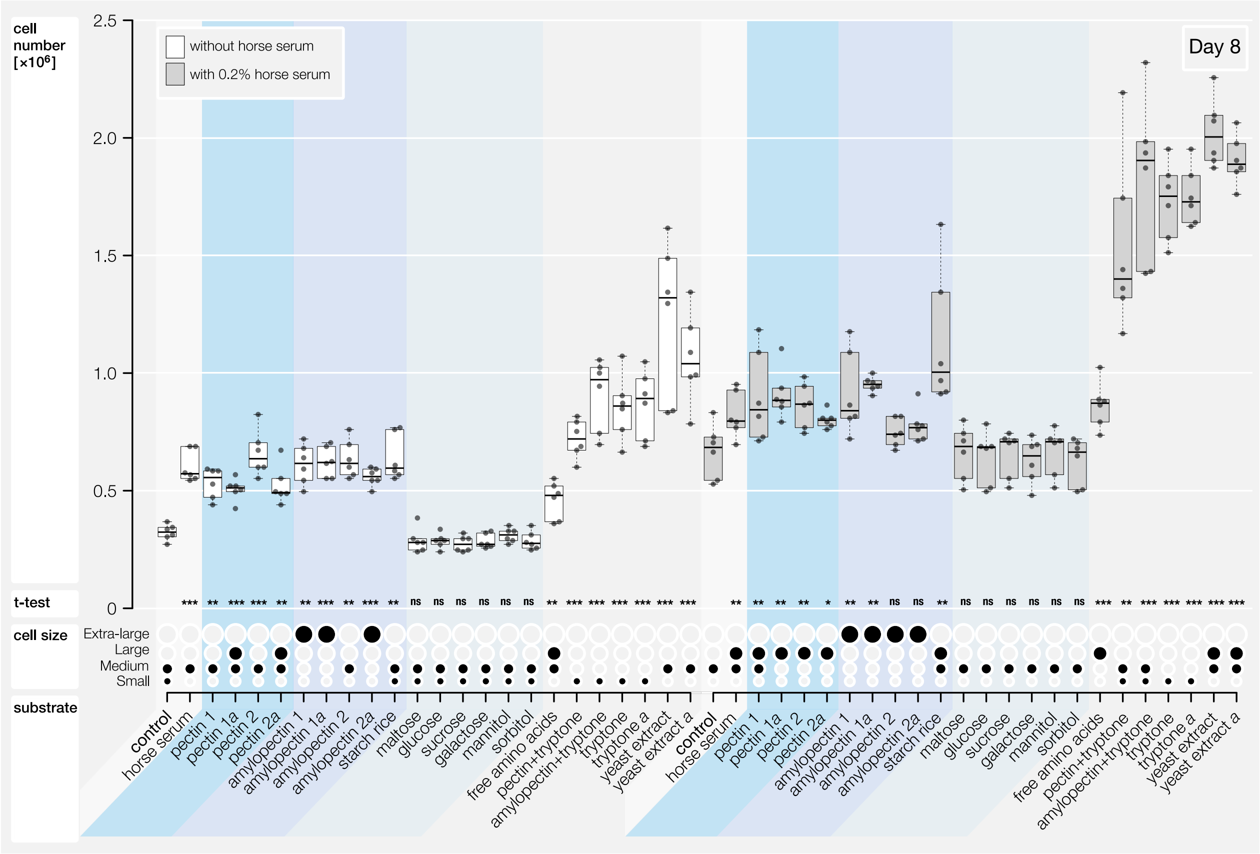

Supplementary Figure S20. **Growth of *D. papillatum* on various substrates.** Approximately 3×10^5 cells from mid-exponential phase were used to inoculate media containing the indicated substrates at 0.1% weight-to-volume (w/v) concentration. White and grey boxes indicate absence or presence of 0.2% horse serum, respectively, in the medium. The following substrates were tested: horse serum, pectin, and amylopectin, each from two different manufacturers, starch rice (~90% starch, ~10% proteins), maltose, glucose, sucrose, galactose, mannitol, sorbitol, free amino acids (glutamate, glutamine, glycine), a mixture of pectin or amylopectin with tryptone (1:1), tryptone, and yeast extract; *a*, autoclaved substrate. (For a detailed composition of the basic medium, which was also used for the 'control' sample, see the *Methods* section.) We made six biological replicates from three independent inocula. The graphs show cell counts after **2**, **4**, and **8** days post-inoculation. Circles of different diameters symbolize the observed cell sizes, with the predominant types indicated by the black fills. Growth rates were compared to the control sample. The two-tailed, paired Student's t-test probabilities are: *, $p < 0.05$; **, $p < 0.01$; ***, $p < 0.001$; ns, not significant). The boxplot whiskers extend to values below the 1.5-fold interquartile range beyond the 1st and 3rd quartile; the boxplot was generated using the BoxPlotR shiny application (SPITZER *et al.* 2014).

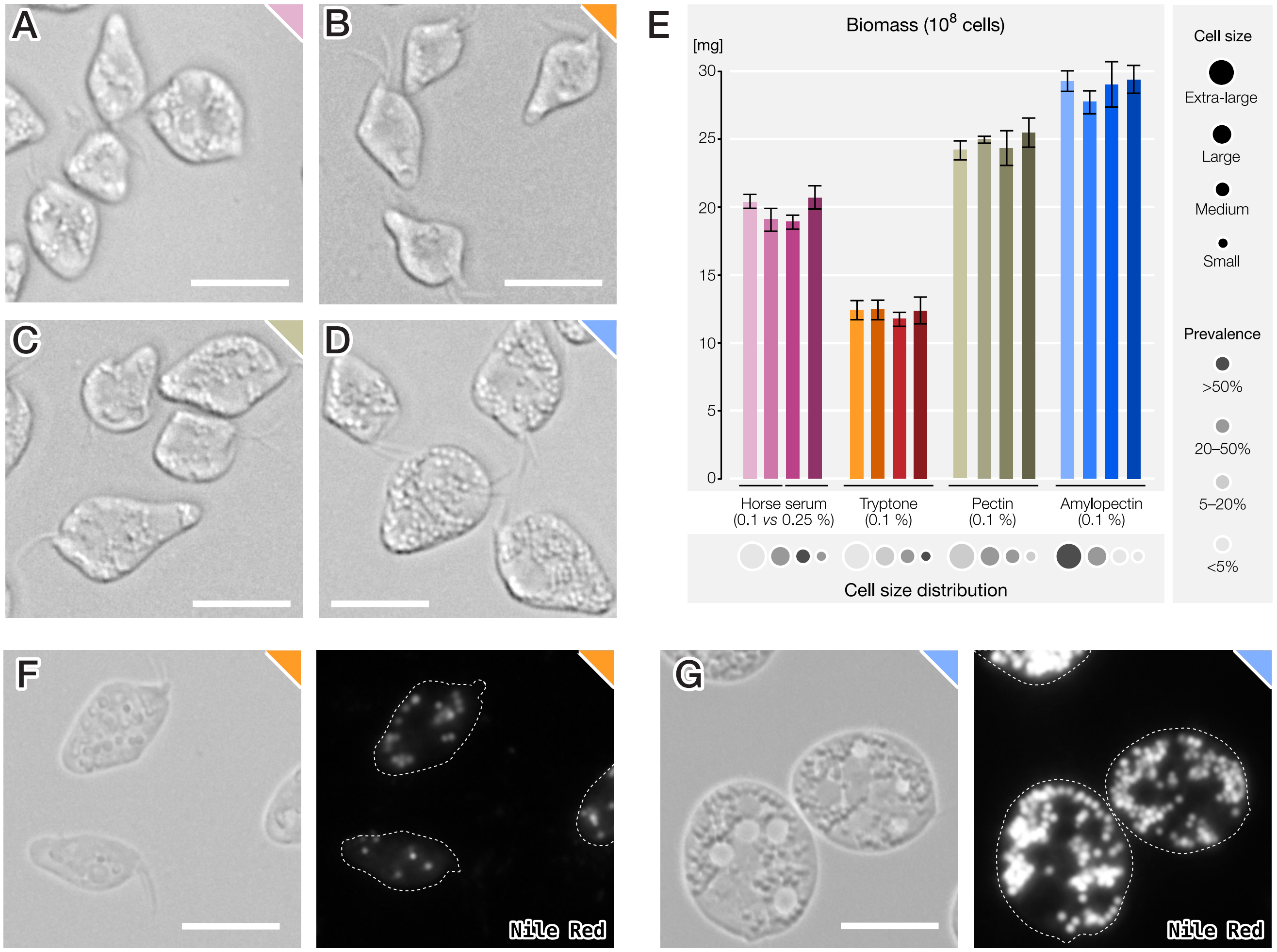

Supplementary Figure S21. *D. papillatum* cell size and biomass depend on the substrate. (**A–D**) Cells cultivated in a medium containing horse serum (**A**), tryptone (**B**), pectin (**C**), or amylopectin (**D**) as a sole carbon source were inspected by light microscopy. White bars correspond to 10 µm. (**E**) Biomass of *D. papillatum* cells grown in various substrates. Cells were counted in triplicate after six days and weighed to calculate their biomass (wet weight per 10^8 cells). Bars indicate the mean deviation of the cell counts for each of the four independent biological replicates. Note that the predominant cell size correlates with both biomass and the number of granules. (**F–G**) Granules, which accumulate especially during growth on glycans, are droplets of neutral lipids. Cells cultivated in a medium containing tryptone (**F**) or amylopectin (**G**) as a sole carbon source were stained with the Nile Red fluorescent reagent. Note that cells in **F–G** are rounder and larger than in **A–D**, because in the former, cells were incubated in a mildly hypotonic buffer to enhance staining. White bars correspond to 10 µm.

Supplementary Table S20. Sugar transporter genes identified in the predicted proteome of *D. papillatum*.

ID	Pfam Domain	Description	Transcripts Per Million (TPM) [a]	RNA-Seq Reads Mapped
DIPPA_16016.mRNA.1	PF00083	generic sugar transporters	3.023213	2406
DIPPA_24401.mRNA.1	PF00083	generic sugar transporters	1.472163	640
DIPPA_24417.mRNA.1	PF00083	generic sugar transporters	3.720192	509
DIPPA_33584.mRNA.1	PF00083	generic sugar transporters	1.558700	580
DIPPA_31431.mRNA.1	PF00083	generic sugar transporters	0.846433	426
DIPPA_31431.mRNA.2	PF00083	generic sugar transporters	0.921461	470
DIPPA_31439.mRNA.1	PF00083	generic sugar transporters	0.257623	274
DIPPA_11817.mRNA.1	PF00083	generic sugar transporters	2.829031	1601
DIPPA_27902.mRNA.1	PF00083	generic sugar transporters	0.958081	453
DIPPA_27956.mRNA.1	PF00083	generic sugar transporters	1.118480	526
DIPPA_27950.mRNA.1	PF00083	generic sugar transporters	4.544418	2141
DIPPA_02168.mRNA.1	PF00083	generic sugar transporters	0.396609	160
DIPPA_17708.mRNA.1	PF00083	generic sugar transporters	3.162026	1851
DIPPA_18224.mRNA.1	PF00083	generic sugar transporters	4.068439	1507
DIPPA_18217.mRNA.1	PF00083	generic sugar transporters	3.686166	1381
DIPPA_18239.mRNA.1	PF00083	generic sugar transporters	3.307667	1228
DIPPA_18361.mRNA.1	PF00083	generic sugar transporters	0.536121	273
DIPPA_18361.mRNA.2	PF00083	generic sugar transporters	0.561651	286
DIPPA_18361.mRNA.3	PF00083	generic sugar transporters	0.402806	170
DIPPA_18361.mRNA.4	PF00083	generic sugar transporters	0.543977	277
DIPPA_04043.mRNA.1	PF00083	generic sugar transporters	0.644033	256
DIPPA_04061.mRNA.1	PF00083	generic sugar transporters	0.915006	246
DIPPA_04061.mRNA.2	PF00083	generic sugar transporters	1.182485	429
DIPPA_34266.mRNA.1	PF00083	generic sugar transporters	2.955753	1335
DIPPA_35197.mRNA.1	PF00083	generic sugar transporters	3.277877	651
DIPPA_59166.mRNA.1	PF00083	generic sugar transporters	1.081599	440
DIPPA_62771.mRNA.1	PF00083	generic sugar transporters	0.942414	243
DIPPA_62771.mRNA.2	PF00083	generic sugar transporters	1.297940	461
TR56418__c0_g1_i1__m.7768	PF00083	generic sugar transporters	6.104713	5146
TR74098__c0_g1_i1__m.13842	PF00083	generic sugar transporters	3.513357	4601
TR116637__c0_g2_i1__m.27060	PF00083	generic sugar transporters	0.411792	74
TR121062__c0_g2_i1__m.28493	PF00083	generic sugar transporters	1.610895	1377
DIPPA_26420.mRNA.1	PF03083	sugar efflux transporters	2.915142	463
DIPPA_01763.mRNA.1	PF03083	sugar efflux transporters	3.803449	691
DIPPA_63739.mRNA.1	PF03083	sugar efflux transporters	1.190166	185
DIPPA_14432.mRNA.1	PF04142	nucleotide-sugar transporter	1.417889	344
DIPPA_16693.mRNA.1	PF04142	nucleotide-sugar transporter	0.786019	206
DIPPA_08985.mRNA.1	PF04142	nucleotide-sugar transporter	2.525701	694
DIPPA_26750.mRNA.1	PF04142	nucleotide-sugar transporter	16.764209	4351
DIPPA_01245.mRNA.1	PF04142	nucleotide-sugar transporter	5.568472	1318
DIPPA_18559.mRNA.1	PF04142	nucleotide-sugar transporter	2.487042	736
DIPPA_05515.mRNA.1	PF04142	nucleotide-sugar transporter	13.207931	3428
DIPPA_24052.mRNA.1	PF04142	nucleotide-sugar transporter	39.831333	8922
DIPPA_23546.mRNA.1	PF04142	nucleotide-sugar transporter	1.728252	431
DIPPA_07719.mRNA.1	PF04142	nucleotide-sugar transporter	0.615102	156
DIPPA_24216.mRNA.1	PF04142	nucleotide-sugar transporter	2.288946	689
DIPPA_09073.mRNA.1	PF04142	nucleotide-sugar transporter	2.096262	631
DIPPA_23121.mRNA.1	PF04142	nucleotide-sugar transporter	0.697580	135
DIPPA_59991.mRNA.1	PF04142	nucleotide-sugar transporter	34.402038	8492
DIPPA_65534.mRNA.1	PF04142	nucleotide-sugar transporter	9.882492	2498
TR66463__c0_g2_i1__m.11111	PF04142	nucleotide-sugar transporter	2.741823	1810
DIPPA_28795.mRNA.1	PF05631	12-TMH MFS-family sugar-transporters	0.978760	370
DIPPA_17988.mRNA.1	PF05631	12-TMH MFS-family sugar-transporters	2.002404	618
DIPPA_17988.mRNA.2	PF05631	12-TMH MFS-family sugar-transporters	2.006976	633

[a] For details on the calculation of transcript levels, see **Supplementary Information**: Section 2. Assembly and annotation of the nuclear genome and transcriptome of *Diplonema papillatum*.

AUTHOR CONTRIBUTIONS

Conceptualization – G.B., M.V.; **Data curation, Formal analysis, Investigation, Visualization, Writing, original draft** – M.V.; **Writing, review & editing** – all co-authors.

REFERENCES

Ball, S., C. Colleoni, U. Cenci, J. N. Raj and C. Tirtiaux, 2011 The evolution of glycogen and starch metabolism in eukaryotes gives molecular clues to understand the establishment of plastid endosymbiosis. J Exp Bot 62: 1775-1801.

Caffall, K. H., and D. Mohnen, 2009 The structure, function, and biosynthesis of plant cell wall pectic polysaccharides. Carbohydr Res 344: 1879-1900.

Chabi, M., M. Leleu, L. Fermont, M. Colpaert, C. Colleoni *et al.*, 2021 Retracing storage polysaccharide evolution in Stramenopila. Front Plant Sci 12: 629045.

Dhital, S., A. K. Shrestha, J. Hasjim and M. J. Gidley, 2011 Physicochemical and structural properties of maize and potato starches as a function of granule size. J Agric Food Chem 59: 10151-10161.

Ferreira, M. J., A. L. Mendes and I. de Sá-Nogueira, 2017 The MsmX ATPase plays a crucial role in pectin mobilization by *Bacillus subtilis*. PLoS One 12: e0189483.

Jeckelmann, J. M., and B. Erni, 2020 Transporters of glucose and other carbohydrates in bacteria. Pflugers Arch 472: 1129-1153.

Le Costaouëc, T., C. Unamunzaga, L. Mantecon and W. Helbert, 2017 New structural insights into the cell-wall polysaccharide of the diatom *Phaeodactylum tricornutum*. Algal Research 26: 172-179.

Morales, J., M. Hashimoto, T. A. Williams, H. Hirawake-Mogi, T. Makiuchi *et al.*, 2016 Differential remodelling of peroxisome function underpins the environmental and metabolic adaptability of diplonemids and kinetoplastids. Proc Biol Sci 283.

Myklestad, S. M., and E. Granum, 2009 Chapter 4.2 - Biology of (1,3)- β -glucans and related glucans in protozoans and chromistans, pp. 353-385 in *Chemistry, Biochemistry, and Biology of 1-3 Beta Glucans and Related Polysaccharides*, edited by A. Bacic, G. B. Fincher and B. A. Stone. Academic Press, San Diego.

Raimundo, S. C., S. Pattathil, S. Eberhard, M. G. Hahn and Z. A. Popper, 2017 β -1,3-Glucans are components of brown seaweed (Phaeophyceae) cell walls. Protoplasma 254: 997-1016.

Scholz, M. J., T. L. Weiss, R. E. Jinkerson, J. Jing, R. Roth *et al.*, 2014 Ultrastructure and composition of the *Nannochloropsis gaditana* cell wall. Eukaryot Cell 13: 1450-1464.

Škodová-Sveráková, I., G. Prokopchuk, P. Peña-Diaz, K. Záhonová, M. Moos *et al.*, 2020 Unique dynamics of paramylon storage in the marine euglenozoan *Diplonema papillatum*. Protist 171: 125717.

Škodová-Sveráková, I., K. Záhonová, V. Juricová, M. Danchenko, M. Moos *et al.*, 2021 Highly flexible metabolism of the marine euglenozoan protist *Diplonema papillatum*. BMC Biol 19: 251.

Spitzer, M., J. Wildenhain, J. Rappsilber and M. Tyers, 2014 BoxPlotR: a web tool for generation of box plots. Nat Methods 11: 121-122.

Subramaniam, R., S. Dufreche, M. Zappi and R. Bajpai, 2010 Microbial lipids from renewable resources: production and characterization. J Ind Microbiol Biotechnol 37: 1271-1287.

Swinkels, J. J. M., 1985 Composition and properties of commercial native starches. Starch - Stärke 37: 1-5.

Valach, M., A. Léveillé-Kunst, M. W. Gray and G. Burger, 2018 Respiratory chain Complex I of unparalleled divergence in diplonemids. J Biol Chem 293: 16043-16056.

14. Secretome prediction

INTRODUCTION

In unicellular eukaryotes, studying the secretome —the totality of proteins actively transported outside of the cell— provides insight into the organism's feeding behaviour and ecological role. Transport 'outside' of the cell means not only secretion into the environment. Certain microeukaryotic groups, such as ciliates and Discoba (including diplonemids), secret proteins also into their cytostome/cytopharynx, an invagination of the plasma membrane specialized for food ingestion. *Diplonema* is among the few microeukaryotes in which the ultrastructure of the feeding apparatus has been examined in great detail (TRIEMER AND OTT 1990; TRIEMER 1992; MONTEGUT-FELKNER AND TRIEMER 1996; TASHYREVA *et al.* 2018).

Most proteins translated in the cytosol follow the secretory pathway to reach their final destination; only those targeted to the nucleus, mitochondria, and peroxisomes take other distinct routes, while cytosolic proteins stay behind. The secretory pathway involves co-translational translocation of proteins into the endoplasmic reticulum (ER) and then transport into the Golgi complex (Golgi). According to the prevailing view, which is based on the 'bulk flow hypothesis', the transport outside the cell is the default track. In contrast, proteins that remain in the ER or the Golgi, or are destined to the vacuoles, carry a specific targeting code. Secreted proteins have a classical N-terminal signal peptide (SP) motif required for entering the secretory pathway; further, they lack an ER-retention signal, trans-membrane spanning domains (the latter include the Golgi-retention signal), and targeting motifs to the nucleus, mitochondrion, peroxisomes, and vacuoles (e.g., (GOGLEVA *et al.* 2018) and references therein).

Experimental secretome determination has recently made major technological advancements by introducing 'spatial proteomics', reducing contaminations with non-secreted proteins, e.g., due to cell injury or apoptosis (reviewed in IMAI AND NAKAI 2020). However, the limitation remains that the experimental results depend on the culture conditions and physiological and developmental stage of the organism, and thus give only a partial picture. The alternative to experimental determination is *in silico* secretome predictions. Available tools use not only the presence/absence of targeting or retention motifs detected by weight matrices or Hidden Markov models (HMMs), but also sequence alignments and general physicochemical properties for a variety of machine-learning algorithms (IMAI AND NAKAI 2020). Yet, predicted secretomes should be also taken with a grain of salt. While most tools have comparable accuracy (CHOO *et al.* 2009), the particular subset of a given proteome predicted by the different tools differ considerably, because the various machine learning algorithms have been trained with different datasets.

In the analysis presented here, the prediction of signal peptide (SP)-carrying proteins was performed with *Phobius* (SONNHAMMER *et al.* 1998). This tool has the advantage over other software such as *SignalP* (PETERSEN *et al.* 2011) and *TargetP* (EMANUELSSON *et al.* 2000) that it infers simultaneously SPs and transmembrane domains (TMDs), thus reducing the common problem of confusing certain TMDs at the N-terminus of proteins with SPs (SONNHAMMER *et al.* 1998).

RESULTS and DISCUSSION

About 10% of the total inferred proteome from *D. papillatum* was predicted to be secreted outside of the cell (**Supplementary Table S21**), which makes the size of the theoretical *Diplonema* secretome comparable with that from other free-living, heterotrophic microeukaryotes: the kinetoplastid *Bodo saltans* (6%; 1,187 out of 18,963 proteins (POWELL *et al.* 2016)); and, as a phylogenetically distant comparison point, fungi (2–8% (LUM AND MIN 2011)). It should be noted that the secretome sizes listed above are only tentatively comparable because different software tools and parameters have been used in the predictions.

To obtain insight into the feeding strategy of *D. papillatum*, we examined the number of inferred proteins with functions in lipid, protein, and carbohydrate degradation, and the percentage of these enzymes predicted to be secreted outside the cell (**Supplementary Table S22**). Lipases and lipoxygenases were identified based on the EC number assigned to them by the automated function-annotation pipeline (see **Supplementary Information**: Section 2. Assembly and annotation of the nuclear genome and transcriptome of *Diplonema papillatum*), whereas proteolytic enzymes were identified by searches against the peptidase database MEROPS, and carbohydrate-degrading enzymes were determined by the procedure used for the daily update of the CAZyme database (CANTAREL *et al.* 2009; LOMBARD *et al.* 2014; DRULA *et al.* 2022), followed by expert validation (see also Supplementary Information on CAZymes). The corresponding enzyme counts in the **Supplementary Table S22** are most likely underestimated because it is sometimes difficult to infer, based on sequence information alone, the exact substrates.

Since the three enzyme classes were identified in very different ways, the class sizes are not comparable to each other, but what is interpretable and relevant is the portion of non-secreted versus secreted proteins. In this latter comparison,

CAZymes stand out, with about 7% being part of the secretome. Among the secreted CAZymes, enzymes that degrade carbohydrates make up as much as 77%. The largest group within these secreted catabolic CAZymes are enzymes that break down pectin. Interestingly, secreted and non-secreted pectinases include the same CAZyme families, which indicates that *D. papillatum* is capable not only of intracellular but also of extracellular pectin degradation. As discussed more extensively in the section on feeding strategy, this finding corroborates the view that by secreting cell-wall and plasma-membrane-degrading enzymes, *Diplonema* is capable of feeding not only on microeukaryotes but also multicellular eukaryotes, i.e., prey that is much larger than it can engulf in the cytopharynx.

Supplementary Table S21. Summary of secretome prediction

Category	Nr. of proteins [a]	% of proteome)
Proteome	42,423	/
Signal-peptide bearing	5,160	12.2%
Secretome [b]	4,467	10.5%

[a] Count includes isoforms.
[b] Signal-peptide bearing proteins minus those with ER-retention signaled.

Supplementary Table S22. Secreted lipid, protein, and carbohydrate-degrading enzymes in *D. papillatum*

Enzyme class	Total nr. of proteins	% of proteome [a]	Secreted proteins in enzyme class		
			Nr.	% of secretome [a]	% of secreted CAZome
Lipases and lipoxygenases [b]	98	0.2%	21	0.5%	/
Proteases [c]	618	1.5%	178	4.0%	/
CAZymes [d]	527	1.2%	**306**	**6.9%**	/
Degraders[e]	369	0.9%	234	5.2%	76.5%
Pectin de-graders[f]	121	0.3%	78	1.7%	25.5%

[a] For the total number of proteins in the inferred proteome and secretome, see **Supplementary Table S21**.
[b] Enzymes were identified by the EC number assigned to the proteins by the function-annotation pipeline (see **Supplementary Information**: Section 2. Assembly and annotation of the nuclear genome and transcriptome of *Diplonema papillatum*). Lipases (EC 3.1.1.1, 2, 4, 7, 32, 47, 24) include carboxylesterases, triacylglycerol lipases, phospholipases A(1) and (2), acetylcholinesterases, and 1-alkyl-2-acetylglycerophosphocholine esterases. Lipoxygenases (EC 1.13.11.12, 75; seven proteins) include linoleate 13S-lipoxygenases and all-trans-8'-apo-beta-carotenal 15,15'-oxygenases.
[c] Enzymes were identified by a search against peptidase subsequences of the Merops peptidase database. From the corresponding peptunit.lib library file, we removed those units that correspond to peptidase inhibitors, transposable-element ORFs or proteasome components; see Methods.
[d] For CAZyme identification, see **Supplementary Information**: Section 12. CAZyme-coding genes in *Diplonema papillatum*.
[e] Proteins belong to the CAZyme classes glycosid hydrolases (GH), carboxyhydrate esterases (CE), and polysaccharide lyases (PL); see **Supplementary Information**: Section 12. CAZyme-coding genes in *Diplonema papillatum*.
[f] Proteins belonging to the CAZyme classes GH28, GH53, GH54, GH78, GH145, CE8, CE13, PL1, and PL4, see **Supplementary Information**: Section 12. CAZyme-coding genes in *Diplonema papillatum*.

METHODS

Secretome prediction

The prediction of signal peptide (SP)-carrying proteins was performed with *Phobius* 1.01 (SONNHAMMER *et al.* 1998). From the predicted SP-carrying proteins, we removed those carrying an ER-retention signal that we recognized by the presence of the typical motif `'[KRHQSA][DENQ]EL'`. The resulting protein collections represents the predicted secretome.

Prediction of lipid, protein and carbohydrate-degrading enzymes

Proteins involved in **lipid degradation** were identified in the inferred *Diplonema* proteome based on their EC number (https://enzyme.expasy.org) assigned by the automated annotation pipeline. For carboxylic-ester hydrolases (CEHs), we selected EC 3.1.1.1 (carboxyesterases), 3.1.1.3 (triacylglycerol lipases), 3.1.1.4 (phospholipases A(2)), 3.1.1.7 (acetylcholinesterases), 3.1.1.32 (phospholipases A(1)), and 3.1.1.47 (1-alkyl-2-acetylglycerophosphocholine esterases), combined with lipoxygenases EC 1.13.11.12 (linoleate 13S-lipoxygenases) and EC 1.13.75 (all-trans-8'-apo-beta-carotenal

15,15'-oxygenases). **Proteases** were predicted by a *BLASTP* search of the *D. papillatum* proteome (reporting threshold 1.0e-05) against peptidase units (pepunit.lib) downloaded from the MEROPS database release 12.4 (RAWLINGS *et al.* 2017). To obtain the list of proteases involved in nutrient digestions, we removed from pepunit.lib peptidase inhibitors, proteasome subunits, and peptidases associated with transposable elements. These sequences were recognized by the following terms in the header line: 'inhibitor unit', 'retrotransposon', 'virus', and 'ubiquitin'. By that, the search space of pepunit.lib was reduced by 15%, from 1,221,971 to 1,046,870 sequences. We also 'cleaned' the blastp output, by removing hits against *Diplonema* annotated as retrotransposon ORFs or containing a reverse-transcriptase domain, reducing the number of matches by 4%. Enzymes involved in the overall carbohydrate metabolism (**CAZymes**) were predicted by integrating gapped *BLASTP* and HMM searches against enzymes with experimentally confirmed function compiled in a 'high-quality library' at the Carbohydrate-Active Enzymes database (CAZy) (CANTAREL *et al.* 2009), followed by expert validation (see also **Supplementary Information**: Section 12. CAZyme-coding genes in *Diplonema papillatum*). The *carbohydrate-degrading* CAZyme subset ('Degraders' in the **Supplementary Table S22**) are those belonging to classes GH (glycosyl hydrolases), carboxyhydrate esterases (CE), or polysaccharide lyases (PL). 'Pectin degraders' are an enzyme subset composed of the classes GH28, GH53, GH54, GH78, GH145, CE8, CE13, PL1, and PL4; see main text and **Additional File 4** cazymeList.

AUTHOR CONTRIBUTIONS

Conceptualization, Data curation, Investigation, Formal analysis, Writing, original draft – G.B.; **Writing, review & editing –** all co-authors.

REFERENCES

Cantarel, B. L., P. M. Coutinho, C. Rancurel, T. Bernard, V. Lombard *et al.*, 2009 The Carbohydrate-Active EnZymes database (CAZy): an expert resource for Glycogenomics. Nucleic Acids Res 37**:** D233-238.

Choo, K. H., T. W. Tan and S. Ranganathan, 2009 A comprehensive assessment of N-terminal signal peptides prediction methods. BMC Bioinformatics 10 Suppl 15**:** S2.

Drula, E., M. L. Garron, S. Dogan, V. Lombard, B. Henrissat *et al.*, 2022 The carbohydrate-active enzyme database: functions and literature. Nucleic Acids Res 50**:** D571-d577.

Emanuelsson, O., H. Nielsen, S. Brunak and G. von Heijne, 2000 Predicting subcellular localization of proteins based on their N-terminal amino acid sequence. J Mol Biol 300**:** 1005-1016.

Gogleva, A., H. G. Drost and S. Schornack, 2018 SecretSanta: flexible pipelines for functional secretome prediction. Bioinformatics 34**:** 2295-2296.

Imai, K., and K. Nakai, 2020 Tools for the recognition of sorting signals and the prediction of subcellular localization of proteins from their amino acid sequences. Front Genet 11**:** 607812.

Lombard, V., H. Golaconda Ramulu, E. Drula, P. M. Coutinho and B. Henrissat, 2014 The carbohydrate-active enzymes database (CAZy) in 2013. Nucleic Acids Res 42**:** D490-495.

Lum, G., and X. J. Min, 2011 FunSecKB: the Fungal Secretome KnowledgeBase. Database (Oxford) 2011**:** bar001.

Montegut-Felkner, A. E., and R. E. Triemer, 1996 Phylogeny of *Diplonema ambulator* (Larsen and Patterson): 2. Homologies of the feeding apparatus. European Journal of Protistology 32**:** 64-76.

Petersen, T. N., S. Brunak, G. von Heijne and H. Nielsen, 2011 SignalP 4.0: discriminating signal peptides from transmembrane regions. Nat Methods 8**:** 785-786.

Powell, B., V. Amerishetty, J. Meinken, G. Knott, F. Yu *et al.*, 2016 ProtSecKB, pp.

Rawlings, N. D., A. J. Barrett, P. D. Thomas, X. Huang, A. Bateman *et al.*, 2017 The MEROPS database of proteolytic enzymes, their substrates and inhibitors in 2017 and a comparison with peptidases in the PANTHER database. Nucleic Acids Res 46**:** D624-D632.

Sonnhammer, E. L., G. von Heijne and A. Krogh, 1998 A hidden Markov model for predicting transmembrane helices in protein sequences. Proc Int Conf Intell Syst Mol Biol 6**:** 175-182.

Tashyreva, D., G. Prokopchuk, J. Votýpka, A. Yabuki, A. Horák *et al.*, 2018 Life cycle, ultrastructure, and phylogeny of new diplonemids and their endosymbiotic bacteria. MBio 9**:** e02447-02417.

Triemer, R. E., 1992 Ultrastructure of mitosis in *Diplonema ambulato*r Larsen and Patterson (Euglenozoa). Eur J Protistol 28**:** 398-404.

Triemer, R. E., and D. W. Ott, 1990 Ultrastructure of *Diplonema ambulator* Larsen & Patterson (Euglenozoa) and its relationship to *Isonema*. Eur J Protistol 25**:** 316-320.

15. Genes horizontally transferred from bacteria to *Diplonema papillatum*

INTRODUCTION

Horizontal (or lateral) gene transfer (HGT) between species, as opposed to the vertical descent of genes from parent to offspring, is a well-known and frequent phenomenon in prokaryotes. Only relatively recent methodological advances and careful experimental design provide convincing support of HGT and its critical contribution to the evolution of eukaryotes (ALSMARK *et al.* 2013; HUSNIK AND MCCUTCHEON 2018; VAN ETTEN AND BHATTACHARYA 2020). For instance, HGT from bacteria to eukaryotes has considerably expanded the capacities of certain eukaryotes to survive in hypoxic or anoxic environments (STAIRS *et al.* 2018; STAIRS *et al.* 2020). Several mutually non-exclusive mechanisms have been proposed for the horizontal transfer of genetic material across domains of life and from bacteria to eukaryotes in particular (SIBBALD *et al.* 2020). For diplonemids, which are free-living marine organisms, at least three pathways for acquiring bacterial genes are plausible; first *via* endosymbionts, since various diplonemids are known to harbour intracellular bacteria (TASHYREVA *et al.* 2018; PROKOPCHUK *et al.* 2019; GEORGE *et al.* 2020), second *via* food, because certain diplonemid species prey on bacteria (PROKOPCHUK *et al.* 2022), and third *via* marine viruses that have been shown to act as predominant HGT vectors in algae (e.g., NELSON *et al.* 2021) and to infect kinetoplastids, the sister clade of diplonemids (KOSTYGOV *et al.* 2021; IRWIN *et al.* 2022).

RESULTS

The identification of protein-coding genes potentially transferred horizontally from bacteria to *D. papillatum* consisted of three steps. First, we collected *D. papillatum* proteins of potential bacterial origin by reciprocal BLAST against NCBI's nr database. Then, we constructed phylogenetic trees for each candidate and its best hits against a taxonomically representative reference proteome database (see Methods) and selected *Diplonema* proteins nested with high support within bacterial clades. Expert validation included visual tree inspection and verification that the HGT candidate gene resides on a contig together with unambiguously nuclear genes.

The *D. papillatum* genome assembly contains 96 genes that most likely originate from bacteria *via* HGT. Phylogenetic analyses (not shown) did not point to any particular bacterial group as a preferential source but indicated a large number of donor clades. At least 90% of HGT genes in *D. papillatum* appear to be functional because they are transcribed, and their pre-mRNAs contain a 5′ spliced leader (SL), as do transcripts from ordinary genes. All genes discussed in more detail below adhere to these criteria.

HGT genes of *D. papillatum* can be grouped into 56 gene families (**Additional File 5** horizTransfer). Ten families comprise multiple members with ~15%–100% protein-sequence identity, while the remaining families consist of singletons. Most multi-member HGT families, especially those with >70% identity across all members, likely arose by amplification of the founder gene after its transfer to the new host. Half of the acquisitions took place specifically in the *D. papillatum* lineage and a quarter in the last common diplonemid ancestor, while the timing of the remaining events is uncertain.

Three-quarters of HGT families in *D. papillatum* have been assigned functions. Most (26 families, 41 proteins) are involved in various metabolic pathways, followed by transport (4 families, 5 proteins), detoxification of reactive oxygen species (3 families, 21 proteins), nucleic acids processing (3 families, 8 proteins) and regulatory functions and signalling (2 families, 2 proteins).

The largest HGT family in the *D. papillatum* genome assembly comprises 14 members of a gene encoding a PAP2-like superfamily protein, with top BLAST hits in the GenBank-nr database annotated as vanadium-dependent haloperoxidases but no significant matches among UniProtKB reviewed sequences. Most of the PAP2-family members are tandemly arranged in the *Diplonema* genome, with up to five genes clustering together (all expert-validated). Another large HGT family, the catalase group, forms a tandem repeat of six almost identical copies (98.6–100% protein-sequence identity; assembly validated by long-read mapping). Phylogenetic analyses of catalase sequences across euglenozoans suggest an alpha-proteobacteria ancestry of this gene and transfer specifically to *D. papillatum* since all other diplonemids possess a eukaryotic-type enzyme (ŠKODOVÁ-SVERÁKOVÁ *et al.* 2020). Catalase genes are known to be frequently transferred across the domains of life (FAGUY AND DOOLITTLE 2000; KRAEVA *et al.* 2017).

Since *D. papillatum* has the potential to degrade diverse polysaccharides, we analyzed in detail the four HGT families (17 genes in total) that are involved in carbohydrate breakdown (see also **Supplementary Information**: Section 12. CAZyme-coding genes in *Diplonema papillatum* and Section 14. Secretome prediction). The corresponding genes appear to be specific to *D. papillatum* to the exclusion of the other ten diplonemid taxa (although only transcriptome data are available for these latter species, the absence of detectable expression strongly indicates the lack of these genes).

With 12 members, the largest of the four HGT-CAZyme families encodes a xylan α-glucuronidase of the glycoside hydrolase family GH115. Ten proteins are full-length (DIPPA_15749 being a prototypical member of this family), whereas DIPPA_07185 and DIPPA_08958 are truncated versions representing an N-terminal and C-terminal moiety, respectively. Half of the genes are arranged in tandem pairs, and both pairs and singles are dispersed across the contigs (all verified by long-read mapping). Protein-sequence identity between family members is above 80%, and DIPPA_15749 and DIPPA_15740 (a tandem pair) are entirely identical. Phylogeny places *Diplonema*'s HGT-GH115-family proteins as a sister clade to sequences from Bacteroidetes, Planctomycetes, Gamma-proteobacteria and Verrucomicrobia (**Supplementary Figure S22**).

The *D. papillatum* genome assembly includes an additional GH115 domain-containing gene, DIPPA_19609, which is not affiliated with bacteria (and therefore was not retrieved during the HGT-detection procedure employed here), but rather with a clade that includes fungi and other eukaryotes (**Supplementary Figure S22**). Whatever the ancestral origin of the corresponding genes may be, they appear to have a long history of residence in eukaryotes. GH115 domain-bearing proteins were also detected in five additional diplonemids to the exclusion of *D. papillatum*; these form yet another clade together with bacterial sequences (**Supplementary Figure S22**).

A second HGT family specific to *D. papillatum* and involved in glycan metabolism comprises proteins characterized by the carbohydrate-binding module CBM6. The three family members share ~99% protein-sequence identity and are arranged in tandem in the genome (assembly verified by long-read mapping). Top BLAST hits in the GenBank-nr database are annotated as endo-1,4-β-xylanase from Delta- and Alpha-proteobacteria. However, the query coverage is low, including only two ~100 amino acid-long regions in the N-terminal and C-terminal CBM6 domains of the *Diplonema* proteins; the ~300 amino acids-long central region has only spurious matches. Therefore, the protein is probably not an enzymatically active xylanase. It should be noted that CBM6 domains occur also in other *D. papillatum* proteins, either as tandem repeats or attached to GH128 domains of β-1,3-glucanases (see also **Supplementary Information**: Section 12. CAZyme-coding genes in *Diplonema papillatum*).

A third type of carbohydrate-degrading genes that *D. papillatum* acquired apparently by HGT is DIPPA_18298, which contains CBM10 and GH26 domains. BLAST searches against a database of experimentally characterized CAZymes and CAZyme domains showed that the GH26 catalytic domain of this protein is >60% identical to the β-1,4-mannanase from *Cellvibrio japonicus* (HOGG *et al.* 2003) and the CBM10 domain resembled that of validated β-mannanases (YOON 2016). This strongly suggests that the *Diplonema* protein most likely targets β-1,4-mannans, which—in addition to glucose and optionally xylose and mannose units—are building blocks of hemi-celluloses.

Lastly, the HGT candidate DIPPA_19532 is predicted to be a D-arabinitol 4-dehydrogenase, an enzyme involved in xylulose reduction (which is part of mannose and fructose metabolic pathways and pentose interconversions). This *D. papillatum* protein is closely related to sequences from several Alpha-proteobacteria.

DISCUSSION

D. papillatum readily expresses at least a hundred genes that originate from bacteria and were acquired recently by either the ancestor of diplonemids or *D. papillatum* itself. The number of HGT genes is similar to that observed across diverse eukaryotic genomes, and the same applies to the nature of the genes: most bacteria-to-eukaryote transferred genes expand or rewire the metabolic capabilities of the recipient (reviewed in (HUSNIK AND MCCUTCHEON 2018; VAN ETTEN AND BHATTACHARYA 2020)). Genes encoding CAZymes have been observed among the most frequently acquired and expanded functional categories across eukaryotes from ciliates to nematodes and oomycetes (RICARD *et al.* 2006; DANCHIN *et al.* 2010; RICHARDS *et al.* 2011; ALSMARK *et al.* 2013). The four CAZyme families specific to *D. papillatum* and obtained from bacteria are most likely involved in the degradation of various hemi-celluloses, mannans, and their derivatives. The acquisition of such enzymes has, in all likelihood, broadened the capacity of *D. papillatum* to feed on the most diverse algae and plants in its natural marine habitat (PORTER 1973).

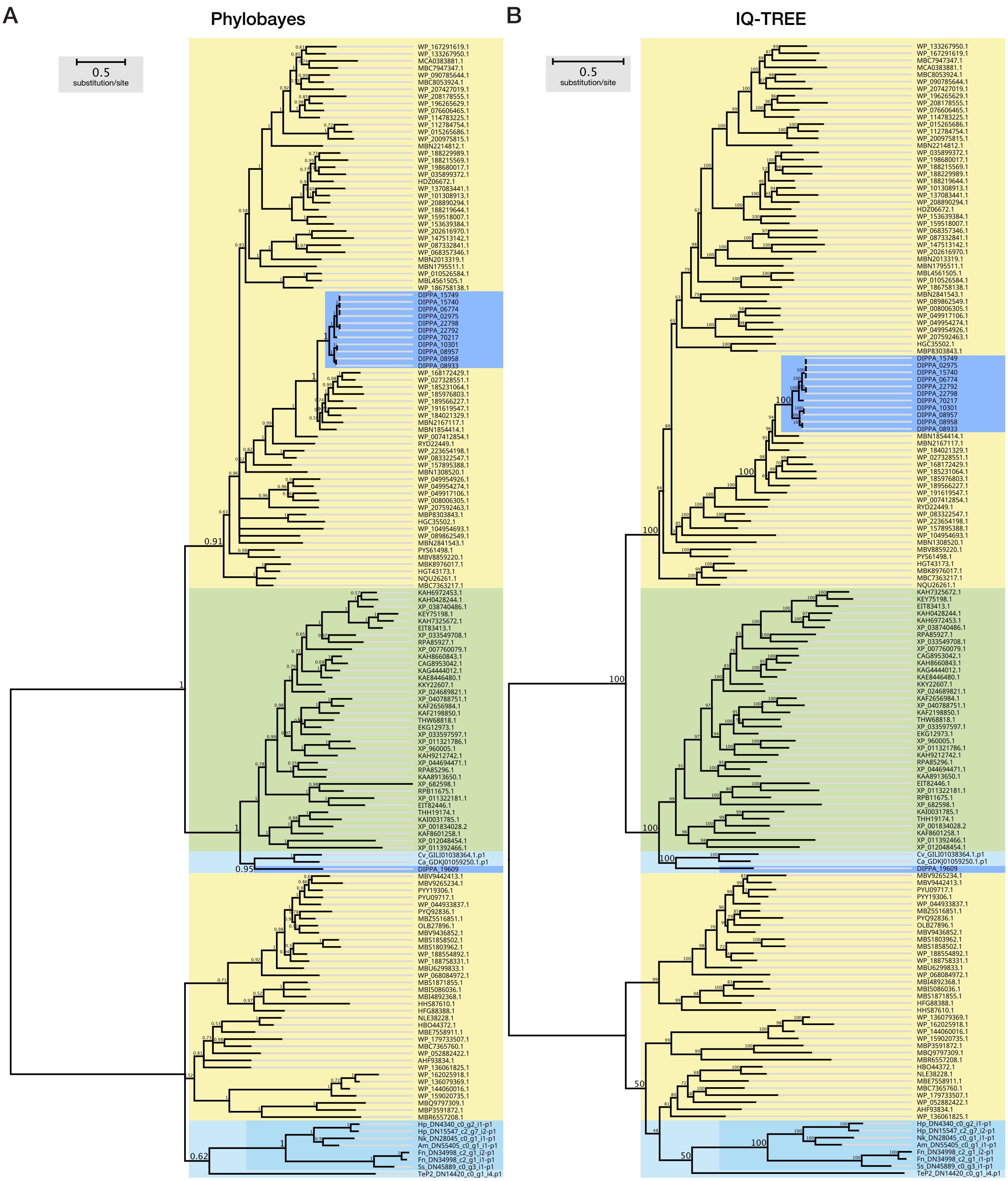

Supplementary Figure S22. Phylogeny of GH115 domain-containing proteins. The phylogenetic tree was constructed with Bayesian (**A**) and maximum likelihood (**B**) methods using the sequences of GH115 domains from a wide collection of proteins (see Methods). Posterior probabilities (Phylobayes) and UF bootstrap support values (IQ-TREE) are indicated next to each branch. Both approaches resolve the tree into several clusters containing bacterial (yellow) or eukaryotic sequences (fungi in green, other eukaryotes in light blue, diplonemids in blue). Sequences specifically from *D. papillatum* are highlighted in darker background shades. Note that DIPPA_07185 was not included in the tree because it lacks the GH115 domain (see text for details).

METHODS

The procedure to detect genes potentially transferred horizontally from bacteria to *D. papillatum* included the following steps. First, HGT candidates were predicted with the taxonomic-annotation algorithm of Diamond v2.0 with default parameters (BUCHFINK *et al.* 2015) using the GenBank-nr database as a reference dataset. This step resulted in 1,317 *D. papillatum* proteins, for which the best hit was a bacterial sequence. These candidates were then queried, using Diamond with default parameters, against the GenBank-nr database and a custom reference dataset comprising proteomes of 408 organisms, notably 148 bacteria, 146 archaea, and 114 eukaryotes, including *D. papillatum* and 17 other euglenozoans (**Additional File 5** horizTransfer). For each of the 1,317 *D. papillatum* proteins, we selected all the hits above the default e-value threshold (0.0001) from the reference database and up to 50 of the top hits in GenBank-nr. All *D. papillatum* sequences that produced reciprocal Diamond hits (e=0.0001) against a *D. papillatum* database were combined, resulting in 370 distinct protein groups. The *D. papillatum* protein groups together with their corresponding hits in the GenBank-nr database and the reference dataset, were aligned with MAFFT v7.4 (KATOH AND STANDLEY 2013) using default parameters. Each multiple alignment was trimmed using BMGE v1.12 with default parameters (CRISCUOLO AND GRIBALDO 2010) and used to build a phylogenetic tree using IQ-TREE v1.6.12 (NGUYEN *et al.* 2015) with the LG+G+F model, -bnni option and 1,000 bootstrap replicates. To detect a HGT signal in the phylogenetic trees, we applied the following algorithm implemented as a custom Python script (available at https://github.com/AnnaNenarokova/ngs/blob/master/projects/hgt/dpapi/check_hgt_trees.py). Branches with <70% bootstrap support were collapsed. If a branch consisted exclusively of one or more diplonemid sequences with two closest neighbouring branches comprising solely bacterial sequences, the diplonemid sequences were considered a result of a singular HGT from bacteria. The 64 trees that displayed the above-described pattern and the underlying multiple protein alignments were inspected in detail to eliminate errors. For example, five *D. papillatum* protein-coding genes, located on two small contigs, were identified as bacterial contamination (see **Supplementary Information**: Section 2. Assembly and annotation of the nuclear genome and transcriptome of *Diplonema papillatum*). Further, proteins in three other trees were considered false positives because the underlying alignment to bacterial sequences relied on marginal similarity. The remaining proteins were further queried against our database of transcriptome-inferred diplonemid proteomes (VALACH *et al.* 2017; BUTENKO *et al.* 2020; KAUR *et al.* 2020) to assess whether the HGT event occurred before or after the emergence of *D. papillatum*.

To construct a phylogeny tree of GH115 domain-containing proteins, we performed BLAST searches using DIPPA_15749 and DIPPA_19609 protein sequences against the full GenBank-nr database, and Diamond searches against a local copy of the EukProt compilation (https://github.com/beaplab/EukProt), as well as against our collection of diplonemid proteomes. The 100 top Blast hits against GenBank-nr were clustered via CD-HIT (FU *et al.* 2012) at a 70% identity threshold, and only the cluster-representative sequences were kept. Sequences were pre-aligned with Muscle v3.8.1551 (EDGAR 2004) and realigned with an HMM search (the profile HMM was built based on the initial Muscle alignment) using hmmsearch (HMMER v3.3) (EDDY 2011). Only those columns of the multiple protein alignment that aligned with a posterior probability of 1.0 were retained for the phylogenetic analysis. For tree construction, we used PhyloBayes v4.1b (LARTILLOT *et al.* 2013) by running four independent chains, six gamma categories and the CAT-GTR model. In addition, IQ-TREE v2.1.3 was used with default parameters and the option to calculate 1,000 ultrafast bootstrap replicates (MINH *et al.* 2020).

AUTHOR CONTRIBUTIONS

Conceptualization, Formal analysis – A.N.; **Data curation, Investigation, Writing, original draft –** A.N., M.V.; **Visualization –** B.F.L., M.V.; **Writing, review & editing –** all co-authors.

REFERENCES

Alsmark, C., P. G. Foster, T. Sicheritz-Ponten, S. Nakjang, T. Martin Embley *et al.*, 2013 Patterns of prokaryotic lateral gene transfers affecting parasitic microbial eukaryotes. Genome Biol 14**:** R19.

Buchfink, B., C. Xie and D. H. Huson, 2015 Fast and sensitive protein alignment using DIAMOND. Nat Methods 12**:** 59-60.

Butenko, A., F. R. Opperdoes, O. Flegontova, A. Horák, V. Hampl *et al.*, 2020 Evolution of metabolic capabilities and molecular features of diplonemids, kinetoplastids, and euglenids. BMC Biol 18**:** 23.

Criscuolo, A., and S. Gribaldo, 2010 BMGE (Block Mapping and Gathering with Entropy): a new software for selection of phylogenetic informative regions from multiple sequence alignments. BMC Evol Biol 10**:** 210.

Danchin, E. G., M. N. Rosso, P. Vieira, J. de Almeida-Engler, P. M. Coutinho *et al.*, 2010 Multiple lateral gene transfers and duplications have promoted plant parasitism ability in nematodes. Proc Natl Acad Sci U S A 107**:** 17651-17656.

Eddy, S. R., 2011 Accelerated profile HMM searches. PLoS Comput Biol 7**:** e1002195.

Edgar, R. C., 2004 MUSCLE: multiple sequence alignment with high accuracy and high throughput. Nucleic Acids Res 32**:** 1792-1797.

Eme, L., E. Gentekaki, B. Curtis, J. M. Archibald and A. J. Roger, 2017 Lateral gene transfer in the adaptation of the anaerobic parasite *Blastocystis* to the gut. Curr Biol 27**:** 807-820.

Faguy, D. M., and W. F. Doolittle, 2000 Horizontal transfer of catalase-peroxidase genes between archaea and pathogenic bacteria. Trends Genet 16**:** 196-197.

Fu, L., B. Niu, Z. Zhu, S. Wu and W. Li, 2012 CD-HIT: accelerated for clustering the next-generation sequencing data. Bioinformatics 28**:** 3150-3152.

George, E. E., F. Husnik, D. Tashyreva, G. Prokopchuk, A. Horák *et al.*, 2020 Highly reduced genomes of protist endosymbionts show evolutionary convergence. Curr Biol 30**:** 925-933.e923.

Hogg, D., G. Pell, P. Dupree, F. Goubet, S. M. Martín-Orúe *et al.*, 2003 The modular architecture of *Cellvibrio japonicus* mannanases in glycoside hydrolase families 5 and 26 points to differences in their role in mannan degradation. Biochem J 371**:** 1027-1043.

Husnik, F., and J. P. McCutcheon, 2018 Functional horizontal gene transfer from bacteria to eukaryotes. Nat Rev Microbiol 16**:** 67-79.

Irwin, N. A. T., A. A. Pittis, T. A. Richards and P. J. Keeling, 2022 Systematic evaluation of horizontal gene transfer between eukaryotes and viruses. Nat Microbiol 7**:** 327-336.

Katoh, K., and D. M. Standley, 2013 MAFFT multiple sequence alignment software version 7: improvements in performance and usability. Mol Biol Evol 30**:** 772-780.

Kaur, B., K. Záhonová, M. Valach, D. Faktorová, G. Prokopchuk *et al.*, 2020 Gene fragmentation and RNA editing without borders: eccentric mitochondrial genomes of diplonemids. Nucleic Acids Res 48**:** 2694-2708.

Kostygov, A. Y., A. Karnkowska, J. Votýpka, D. Tashyreva, K. Maciszewski *et al.*, 2021 Euglenozoa: taxonomy, diversity and ecology, symbioses and viruses. Open Biol 11**:** 200407.

Kraeva, N., E. Horáková, A. Y. Kostygov, L. Kořený, A. Butenko *et al.*, 2017 Catalase in *Leishmaniinae*: with me or against me? Infect Genet Evol 50**:** 121-127.

Lartillot, N., N. Rodrigue, D. Stubbs and J. Richer, 2013 PhyloBayes MPI: phylogenetic reconstruction with infinite mixtures of profiles in a parallel environment. Syst Biol 62**:** 611-615.

Minh, B. Q., H. A. Schmidt, O. Chernomor, D. Schrempf, M. D. Woodhams *et al.*, 2020 IQ-TREE 2: New models and efficient methods for phylogenetic inference in the genomic era. Mol Biol Evol 37**:** 1530-1534.

Nelson, D. R., K. M. Hazzouri, K. J. Lauersen, A. Jaiswal, A. Chaiboonchoe *et al.*, 2021 Large-scale genome sequencing reveals the driving forces of viruses in microalgal evolution. Cell Host Microbe 29**:** 250-266.e258.

Nguyen, L. T., H. A. Schmidt, A. von Haeseler and B. Q. Minh, 2015 IQ-TREE: a fast and effective stochastic algorithm for estimating maximum-likelihood phylogenies. Mol Biol Evol 32**:** 268-274.

Porter, D., 1973 *Isonema papillatum* sp. n., a new colorless marine flagellate: a light- and electronmicroscopic study. The Journal of Protozoology 20**:** 351-356.

Prokopchuk, G., T. Korytář, V. Juricová, J. Majstorović, A. Horák *et al.*, 2022 Trophic flexibility of marine diplonemids - switching from osmotrophy to bacterivory. Isme j.

Prokopchuk, G., D. Tashyreva, A. Yabuki, A. Horák, P. Masařová *et al.*, 2019 Morphological, ultrastructural, motility and evolutionary characterization of two new Hemistasiidae species. Protist 170**:** 259-282.

Ricard, G., N. R. McEwan, B. E. Dutilh, J. P. Jouany, D. Macheboeuf *et al.*, 2006 Horizontal gene transfer from bacteria to rumen ciliates indicates adaptation to their anaerobic, carbohydrates-rich environment. BMC Genomics 7**:** 22.

Richards, T. A., D. M. Soanes, M. D. Jones, O. Vasieva, G. Leonard *et al.*, 2011 Horizontal gene transfer facilitated the evolution of plant parasitic mechanisms in the oomycetes. Proc Natl Acad Sci U S A 108**:** 15258-15263.

Sibbald, S. J., L. Eme, J. M. Archibald and A. J. Roger, 2020 Lateral gene transfer mechanisms and pan-genomes in eukaryotes. Trends Parasitol 36**:** 927-941.

Škodová-Sveráková, I., K. Záhonová, B. Bučková, Z. Füssy, V. Yurchenko *et al.*, 2020 Catalase and ascorbate peroxidase in euglenozoan protists. Pathogens 9.

Stairs, C. W., J. E. Dharamshi, D. Tamarit, L. Eme, S. L. Jørgensen *et al.*, 2020 Chlamydial contribution to anaerobic metabolism during eukaryotic evolution. Sci Adv 6**:** eabb7258.

Stairs, C. W., L. Eme, S. A. Muñoz-Gómez, A. Cohen, G. Dellaire *et al.*, 2018 Microbial eukaryotes have adapted to hypoxia by horizontal acquisitions of a gene involved in rhodoquinone biosynthesis. Elife 7.

Tashyreva, D., G. Prokopchuk, J. Votypka, A. Yabuki, A. Horak *et al.*, 2018 Life cycle, ultrastructure, and phylogeny of new diplonemids and their endosymbiotic bacteria. MBio 9**:** e02447-02417.

Valach, M., S. Moreira, S. Hoffmann, P. F. Stadler and G. Burger, 2017 Keeping it complicated: mitochondrial genome plasticity across diplonemids. Sci Rep 7**:** 14166.

Van Etten, J., and D. Bhattacharya, 2020 Horizontal gene transfer in eukaryotes: not if, but how much? Trends Genet 36**:** 915-925.

Yoon, K.-H., 2016 Molecular cloning and characterization of β-mannanase B from *Cellulosimicrobium* sp. YB-43. Korean Journal of Microbiology 52**:** 336-343.

16. Evolution of gene families

INTRODUCTION

Gene families are groups of genes in a given organism that have evolved by duplication from a common ancestor. Family members (paralogs) diversify over time by 'dividing labor' (subfunctionalization) or adopting new functions (neofunctionalization). Gene families may continue to grow or shrink if there is no selective force to maintain all copies. New gene families can have different origins. They may emerge either from initially non-coding regions or by horizontal gene transfer. Alternatively, new families can originate from pseudogenes whose ultimate descent is unrecognizable.

A key factor in the genetic diversity of organisms is the evolution of gene families. Birth and death, expansion and contraction of families, provide a window on the particular adaptations of species to their environment. The aim of analyzing gene family evolution in diplonemids and related taxa was to delineate genetic factors potentially contributing to the success of diplonemids in marine ecosystems.

RESULTS and DISCUSSION

For comparative gene family analysis, we established a phylogenetically equilibrated reference dataset from 30 eukaryotic species (reference dataset; **Supplementary Table S23**). The genome-inferred proteomes from diplonemids and the reference species were clustered into ~30,000 families, each of which contained at least two proteins—more than a third included sequences from *D. papillatum*. To trace the evolution of those gene families that are widely represented across eukaryotes, we first constructed a phylogenetic tree using the 167 orthologous proteins that are present in 25 out of 30 species in the dataset. The resulting tree is well-resolved, with all the internal nodes of Euglenozoa having maximum support values (**Supplementary Figure S23**).

As the next step, we determined the evolution of protein families by fitting the data to a birth-death model (CSURÖS 2010), which involved the mapping the family counts onto the tree topology (**Supplementary Figure S23**). Interestingly, in Euglenozoa, the gains of new families and the expansions of existing families are much more frequent than losses and contractions. The by far highest count of gains (~7,000) in the entire tree is the ancestral diplonemid node (N10), demonstrating a substantial diversification of the gene repertoire in the last common ancestor of diplonemids. Similarly prolific is the expansion of gene families at that node.

The euglenozoan clade experienced predominantly family gains and expansions, but exceptions were observed in Kinetoplastea, including the ancestral node (N21) and free-living kinetoplastids. At this node, family contractions and member losses occurred predominantly in genes involved in metabolism, which is corroborated by previous reports of reduced metabolic capabilities in this group (OPPERDOES *et al.* 2016; BUTENKO *et al.* 2020).

In the following, we discuss gains and expansions of the diplonemids; details of the analysis are compiled in the **Additional File 6** geneFam.

Families gains in diplonemids

Out of the ~5,500 gains traced to the diplonemid ancestral branch, about 22% have a functional annotation, inferred from the closest homolog found in the Uniprot database (see **Supplementary Information**: Section 2. Assembly and annotation of the nuclear genome and transcriptome of *Diplonema papillatum*). Only ~200 protein families could be confidently assigned a KEGG ID and consequently classified into higher-order KEGG categories (e.g., 'metabolism', 'signal transduction', 'folding, sorting and degradation', etc.). The low number is presumably due to sequence divergence and sparsity of the KEGG database coverage among unconventional model organisms. A non-exhaustive inspection of the annotations of functionally annotated gene families, which could not be automatically assigned a KEGG ID, indicated that these were distributed across KEGG-type categories in similar proportions as the assigned ones. Thus, the distributions discussed hereafter, while limited to the KEGG-assigned dataset, likely mirror the distribution of the entire dataset in relative proportions (though not in absolute numbers).

Proteins involved in metabolic processes represent the largest group of KEGG-annotated families gained by the diplonemid ancestor. Families in the KEGG category '**carbohydrate metabolism**' have the largest share. Predicted glucoside hydrolases, and particularly glucanases, point to specialization in algal or plant food sources. Diverse gene families for carbohydrate-active enzymes have been specifically gained in *D. papillatum*. The remarkable repertoire of the type species has been analyzed in detail in another section (**Supplementary Information**: Section 12. CAZyme-coding genes in *Diplonema papillatum*).

Families of metabolic proteins falling in the KEGG category '**amino-acid metabolism**' are also numerous. In contrast to other Euglenozoa in our dataset, diplonemids appear to be capable of creatine biosynthesis because the genes for both enzymes, glycine amidinotransferase and guanidinoacetate N-methyltransferase, are present. Therefore, diplonemids likely represent an important source of creatine in the ocean, in addition to metazoans and diatoms (WAWRIK *et al.* 2017). All Euglenozoa, except the parasitic trypanosomatids (which use arginine phosphate as energy buffer (PEREIRA *et al.* 2000)), possess creatine kinase and thus can convert creatine to energy-rich creatine phosphate. It remains to be elucidated which compound serves as a creatine source for euglenozoans other than diplonemids or, alternatively, whether they can survive without it.

At least 40 KEGG-annotated families gained by the ancestral diplonemid branch are involved in **environmental-information processing** and **signal transductions**, with putative kinases, phosphatases and peptidases accounting together for ~12% of all functionally annotated protein families. Notably, two adenylate cyclase families (one with up to 80 members in diplonemids) were gained by the last common diplonemid ancestor. An analysis of kinase-domain-containing proteins (using Pfam PF00069 as a query) across diplonemids, euglenids and free-living prokinetoplastids, revealed between 500 and 1,000 kinase homologs in each analyzed species, numbers comparable to the situation in human (518) and plants (600–2,500) (MANNING *et al.* 2002; LEHTI-SHIU AND SHIU 2012).This observation points to an elaborate kinase repertoire and complex signaling pathways in free-living (unicellular) Euglenozoa, comparable to those in multicellular organisms.

Families expanded in diplonemids

In addition to family gains, expansions appear to have played a comparably important role in the evolution of diplonemids. Similarly to gene gains, expanded families often contribute to **metabolic processes and signaling** (126 and 35 families). In addition, 71 expanded families act in **translation and transcription**, including initiation factors and ribosomal proteins (RPs; see also **Supplementary Information**: Section 10. The cytosolic ribosome of *Diplonema papillatum*). The incorporation of various RP paralogs into ribosomes may allow translation regulation under diverse and fluctuating environmental conditions (HUMMEL *et al.* 2012).

The majority of families expanded specifically in *D. papillatum* are characterized by a small number of members (mainly two or three; **Supplementary Figure S24A**) with highly similar protein sequences (>90%; **Supplementary Figure S24B**), which indicates recent duplication events. In contrast, most families expanded in the diplonemid ancestor seem to have undergone an early diversification (**Supplementary Figure S25**). An interesting group of families expanded in *D. papillatum* but not in the other diplonemids are involved in **oxidative stress protection** (e.g., glutathione S-transferase, cytochrome *c* peroxidase, tryparedoxin, glutaredoxin). Therefore, the type species might be well adapted to life in the surface seawater layer penetrated by solar radiation or in coastal waters polluted with metals, polychlorinated biphenyls, and radioactive waste —conditions triggering the production of cytotoxic reactive oxygen species (LUSHCHAK 2011).

Limitations of the presented analysis

Several shortcomings have complicated our analysis and the consequent interpretation of its results. First, a high proportion of proteins from the understudied diplonemids is not annotated, which applies to 81% and 48% of gained and expanded families, respectively, on the ancestral diplonemid branch. Second, sequencing depth varies across diplonemids, with the effect that certain gene families appear absent in a given species only because the available dataset is less complete than another. Third, cytosolic rRNA phylogenies (TASHYREVA *et al.* 2018) and analyses of the mitochondrial genome (YABUKI *et al.* 2016; KAUR *et al.* 2020) point to an exceptionally accelerated evolution of the *Hemistasia* nuclear genome. Highly divergent protein sequences hamper recognizing homologs and thus artificially increase the count of family gains and lower the count of expansions. Finally, a major difficulty is that only transcriptome-inferred proteomes are available for diplonemids other than *D. papillatum* (**Supplementary Table S23**). In particular, the genome-inferred proteome of *D. papillatum* includes >250 transposon-related hypothetical proteins (see also **Supplementary Information**: Section 2. Assembly and annotation of the nuclear genome and transcriptome of *Diplonema papillatum* and **Supplementary Information**: Section 6. Repetitive sequences in the nuclear genome of *Diplonema papillatum* (assembly v_1.0)), but the corresponding genes seem not to be expressed under usual cultivation conditions because their mRNAs are absent in our RNA-Seq data. Therefore, the considerable family gains and expansions of the type species seen in **Supplementary Figure S23** should be taken with a grain of salt as some of these families are made up of non-expressed genes. Once genome information is available from other diplonemids, the evolution of gene families originating from transposable elements will be worth revisiting.

In sum, almost 80% gained and ~60% expanded gene families in diplonemids lack any functional annotation (i.e., are annotated as 'hypothetical protein'). Out of these, >60% occur in at least three of the four analyzed diplonemids (*D. papillatum*, *Sulcionema specki*, *Rhynchopus humris*, *Hemistasia phaeocysticola*). Most of these conserved genes have a

well-expressed representative in *D. papillatum*, which underscores their functional importance. Functional genetics studies will be critical in determining the biological roles of the genetic ‘dark matter’ comprised in diplonemid genomes.

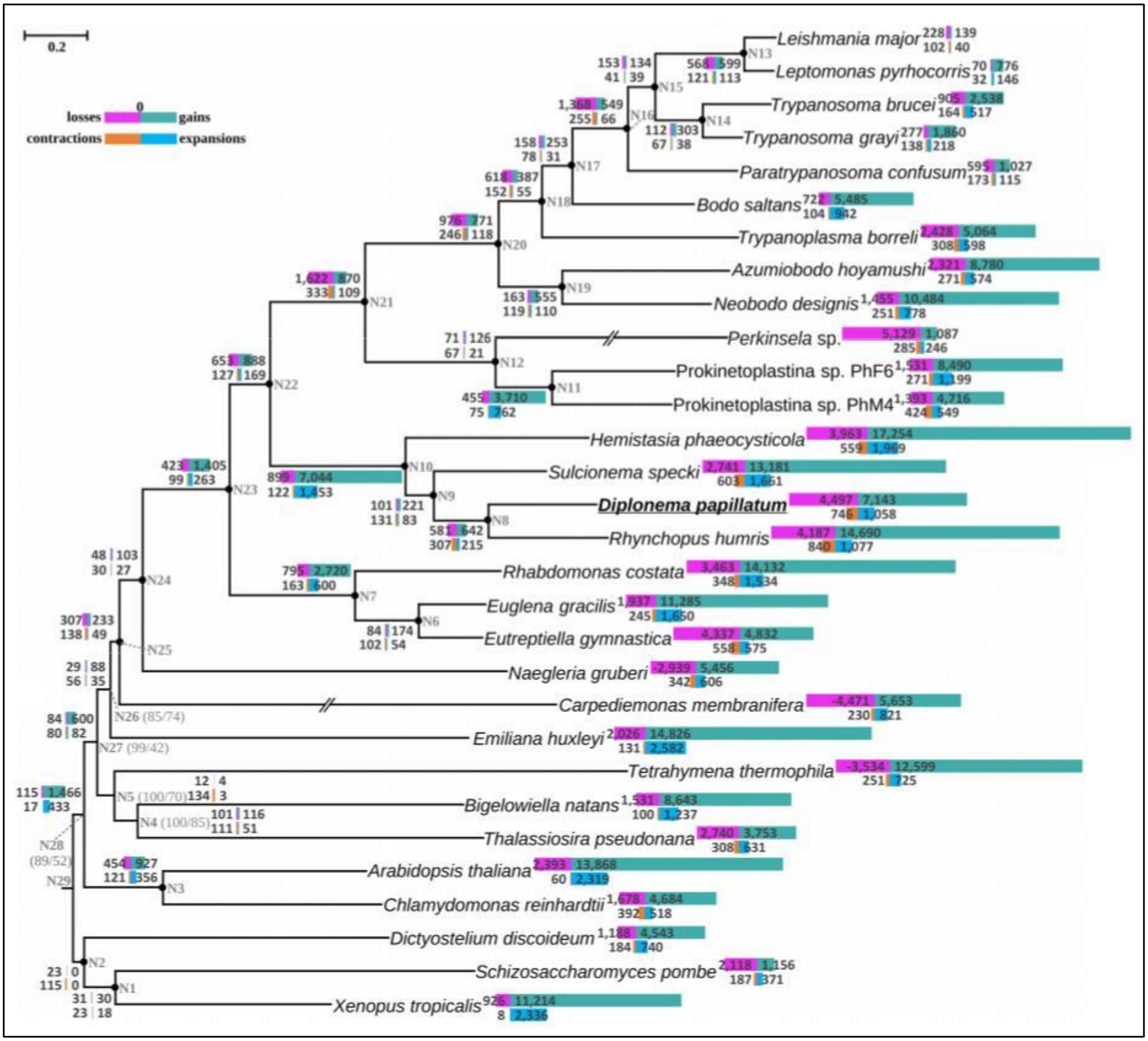

Supplementary Figure S23. Evolution of protein families. A maximum-likelihood phylogenetic tree based on the concatenated alignment of 167 proteins containing 57,565 amino acid positions. Nodes with maximal statistical support are indicated with black circles; for the remaining nodes the supports are in grey in the following format: (bootstrap support/SH-aLRT value). Double-crossed branches were reduced to half of their original length. Node numbers are shown in grey. Black horizontal bar indicates the number of substitutions per site. The number of protein families gained, lost, expanded, or contracted at certain nodes (based on the sum of probabilities of the respective events at each node/tip) is depicted with green, magenta, blue, and orange bars, respectively. Note the substantial gain of 7,044 families on the ancestral diplonemid branch, which is the largest gene gain on an internal node. See the discussion on *Limitations*.

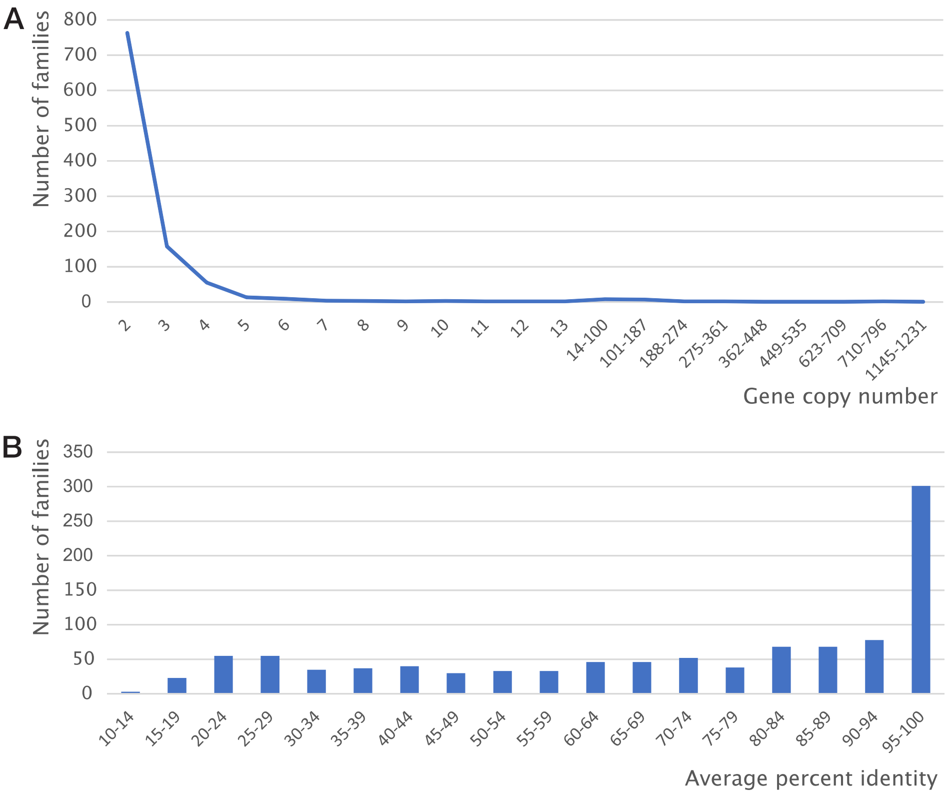

Supplementary Figure S24. Statistics for protein families expanded in *D. papillatum*. **A**) The distribution of families ranked by the number of family members (copies) they contain. **B**) The distribution of families ranked by the average percent of protein identity within the group.

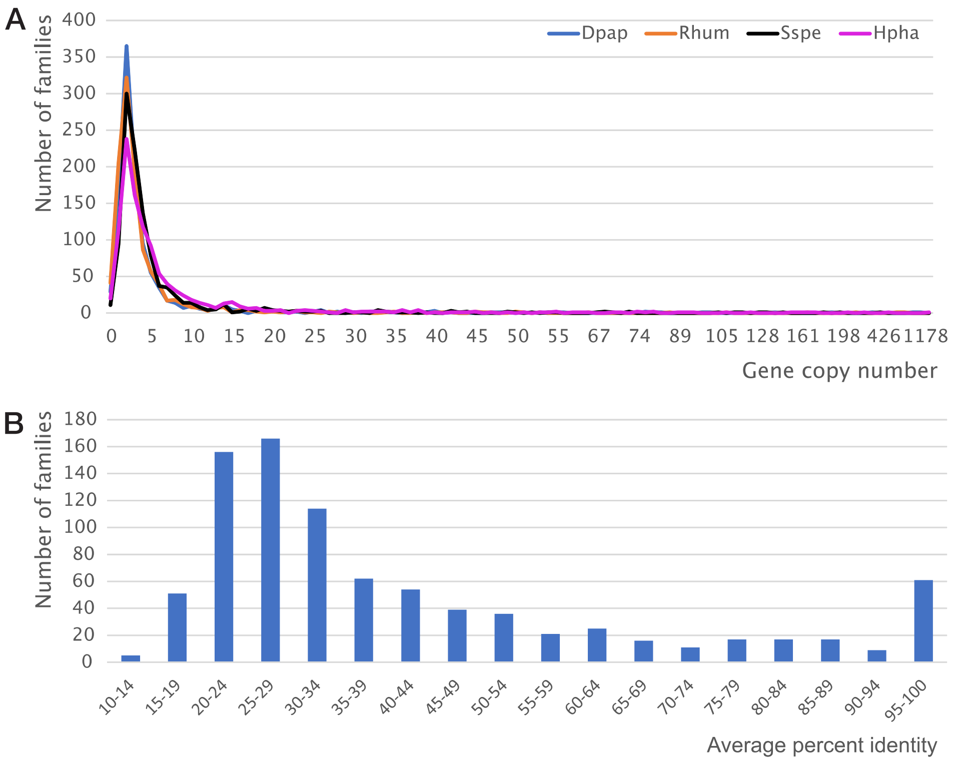

Supplementary Figure S25. Statistics for protein families expanded in the reconstructed diplonemid ancestor. **A**) The distribution of families according to the number of proteins they contain. **B**) The distribution of families according to the average *D. papillatum* protein percent identity within the groups. Species abbreviations are as follows: Dpap, *D. papillatum*; Rhum, *R. humris*; Sspe, *S. specki*; Hpha, *H. phaeocysticola*.

Supplementary Table S23. Data sources.

Species	Strain	Abbreviation	Lineage	Data source[a]	Proteome inferred
Arabidopsis thaliana	-	Atha	Streptophyta	NCBI Genomes	Genome
Azumiobodo hoyamushi	Hirose, Nozawa,	Ahoya	Euglenozoa	(YAZAKI *et al.* 2017)	Transcriptome
Bigelowiella natans		Bnat	Rhizaria		Genome
Bodo saltans	Konstanz	Bsal	Euglenozoa	Wellcome Trust	Genome
Carpediemonas	-	Cmem	CLO/Fornicata	NCBI Genomes	Genome
Chlamydomonas reinhardtii	-	Crei	Chlorophyta	NCBI Genomes	Genome
Dictyostelium discoideum	AX4	Ddis	Amoebozoa	NCBI Genomes	Genome
Diplonema papillatum	ATCC 50162	Dpap	Euglenozoa	this study	Genome
Emiliania huxleyi	CCMP1516	Ehux	Haptophyta	NCBI Genomes	Genome
Euglena gracilis	Z	Egra	Euglenozoa	(EBENEZER *et al.*	Transcriptome
Eutreptiella gymnastica	NIES-381	Egym	Euglenozoa	MMETSP	Transcriptome
Hemistasia phaeocysticola	YPF1303	Hpha	Euglenozoa	(BUTENKO *et al.*	Transcriptome
Leishmania major	Friedlin	Lmaj	Euglenozoa	TriTryp v.9.0	Genome
Leptomonas pyrrhocoris	H10	Lpyr	Euglenozoa	TriTryp v.9.0	Genome
Naegleria gruberi	NEG-M	Ngru	Heterolobosea	NCBI Genomes	Genome
Neobodo designis	CCAP 1951/1	Ndes	Euglenozoa	MMETSP	Transcriptome
Paratrypanosoma confusum	CUL13-MS	Pcon	Euglenozoa	TriTryp v.9.0	Genome
Perkinsela sp.	CCAP 1560/4	Perk	Euglenozoa	NCBI Genomes	Genome
Prokinetoplastina sp.	PhM-4	PhM-4	Euglenozoa	Patrick Keeling	Transcriptome
Prokinetoplastina sp.	PhF-6	PhF-6	Euglenozoa	Patrick Keeling	Transcriptome
Rhabdomonas costata	PANT2	Rcos	Euglenozoa	Vladimír Hampl	Transcriptome
Rhynchopus humris	YPF1608	Rhum	Euglenozoa	(BUTENKO *et al.*	Transcriptome
Schizosaccharomyces pombe	assembly ASM294v2	Spomb	Fungi	NCBI Genomes	Genome
Sulcionema specki	YPF1618	Sspe	Euglenozoa	(BUTENKO *et al.*	Transcriptome
Tetrahymena thermophila	SB210 (assembly JCVI-	Ttherm	Alveolata	NCBI Genomes	Genome
Thalassiosira pseudonana	CCMP1335	Tpse	Stramenopiles	NCBI Genomes	Genome
Trypanoplasma borreli	Tt-JH	Tbor	Euglenozoa	(BUTENKO *et al.*	Transcriptome
Trypanosoma brucei	TREU927	Tbru	Euglenozoa	TriTryp v.9.0	Genome
Trypanosoma grayi	ANR4	Tgra	Euglenozoa	TriTryp v.9.0	Genome
Xenopus tropicalis	-	Xtro	Metazoa	NCBI Genomes	Genome

[a] Download date of all datasets, except when specified otherwise: 06/2017-02/2018.

MATERIALS AND METHODS

Inference of protein families and phylogenomic tree construction

Clustering of proteins into families was performed using OrthoFinder v2.1.2 (EMMS AND KELLY 2019) with the default settings on a dataset of 30 species, including *D. papillatum*, 18 other euglenozoans and several additional species representing major eukaryotic outgroup lineages (**Supplementary Table S23**). In the case of proteins predicted from the euglenozoan transcriptomes, CD-HIT-EST (LI AND GODZIK 2006) with the 90% identity threshold was used to reduce protein redundancy caused by the presence of transcript isoforms. For phylogenomic tree construction, proteins present in two or more copies were removed from the OrthoFinder output, and 167 families containing proteins encoded by single-copy genes in at least 25 species out of 30 were used in further analysis. The respective sequences were aligned using MAFFT v7.402 with 1,000 iterations and the "localpair" option (KATOH AND STANDLEY 2013), trimmed with TrimAl v1.2 (CAPELLA-GUTIÉRREZ *et al.* 2009) to remove poorly-aligned positions and concatenated. The phylogenomic tree was inferred using IQ-Tree v1.6.12 (NGUYEN *et al.* 2015) with the LG+C60+F substitution model, which was the best-fitting model according to the BIC criterion; 1000 ultrafast bootstrap replicates were used to assess branch support (HOANG *et al.* 2018). Gene family evolution was modelled on the phylogenomic tree using the phylogenetic birth-and-death model implemented in Count (CSURÖS 2010). We fit a birth-death model with three rate categories for each, gene gain, loss, duplication, and family-specific rate multipliers. The family-wise posterior probabilities of gene presence, expansion, and contraction at each internal node of the phylogeny were used to compute the number of events at each node and the list of genes present with probability ≥0.5; the latter was used for ancestral metabolic reconstructions. Since we experienced problems fitting a birth-death model for the largest gene families, we employed a Wagner parsimony analysis with a gain penalty of 3 for the families containing more than 150 members (332 families in total).

Sequence analysis

For the analysis of general metabolism, BLASTp searches with an E-value cut-off of 10e-20 were conducted against the Euglenozoa species in the reference dataset (**Supplementary Table S23**) (CAMACHO *et al.* 2009). When necessary, additional homology searches were performed using the HMMER package v.3.1 with an E-value cut-off of 10e-5 (EDDY 2011). The average percent identity within OGs of interest was calculated using the alistat script from the HMMER package. Signal peptides were predicted using SignalP v. 5.0 server (ALMAGRO ARMENTEROS *et al.* 2019).

AUTHOR CONTRIBUTIONS

Conceptualization – A.B., T.W.; **Data curation, Investigation, Formal analysis** – A.B., T.W., C.P.; **Visualization** – A.B.; **Writing, original draft** – A.B., T.W., C.P., J.L.; **Writing, review & editing** – all authors.

REFERENCES

Almagro Armenteros, J. J., K. D. Tsirigos, C. K. Sønderby, T. N. Petersen, O. Winther *et al.*, 2019 SignalP 5.0 improves signal peptide predictions using deep neural networks. Nat Biotechnol 37**:** 420-423.

Butenko, A., F. R. Opperdoes, O. Flegontova, A. Horák, V. Hampl *et al.*, 2020 Evolution of metabolic capabilities and molecular features of diplonemids, kinetoplastids, and euglenids. BMC Biol 18**:** 23.

Camacho, C., G. Coulouris, V. Avagyan, N. Ma, J. Papadopoulos *et al.*, 2009 BLAST+: architecture and applications. BMC Bioinformatics 10**:** 421.

Capella-Gutiérrez, S., J. M. Silla-Martínez and T. Gabaldón, 2009 trimAl: a tool for automated alignment trimming in large-scale phylogenetic analyses. Bioinformatics 25**:** 1972-1973.

Csurös, M., 2010 Count: evolutionary analysis of phylogenetic profiles with parsimony and likelihood. Bioinformatics 26**:** 1910-1912.

Ebenezer, T. E., M. Zoltner, A. Burrell, A. Nenarokova, A. M. G. Novák Vanclová *et al.*, 2019 Transcriptome, proteome and draft genome of *Euglena gracilis*. BMC Biol 17**:** 11.

Eddy, S. R., 2011 Accelerated profile HMM searches. PLoS Comput Biol 7**:** e1002195.

Emms, D. M., and S. Kelly, 2019 OrthoFinder: phylogenetic orthology inference for comparative genomics. Genome Biol 20**:** 238.

Hoang, D. T., O. Chernomor, A. von Haeseler, B. Q. Minh and L. S. Vinh, 2018 UFBoot2: improving the ultrafast bootstrap approximation. Mol Biol Evol 35**:** 518-522.

Hummel, M., J. H. Cordewener, J. C. de Groot, S. Smeekens, A. H. America *et al.*, 2012 Dynamic protein composition of *Arabidopsis thaliana* cytosolic ribosomes in response to sucrose feeding as revealed by label free MSE proteomics. Proteomics 12**:** 1024-1038.

Katoh, K., and D. M. Standley, 2013 MAFFT multiple sequence alignment software version 7: improvements in performance and usability. Mol Biol Evol 30**:** 772-780.

Kaur, B., K. Záhonová, M. Valach, D. Faktorová, G. Prokopchuk *et al.*, 2020 Gene fragmentation and RNA editing without borders: eccentric mitochondrial genomes of diplonemids. Nucleic Acids Res 48**:** 2694-2708.

Lehti-Shiu, M. D., and S. H. Shiu, 2012 Diversity, classification and function of the plant protein kinase superfamily. Philos Trans R Soc Lond B Biol Sci 367**:** 2619-2639.

Li, W., and A. Godzik, 2006 Cd-hit: a fast program for clustering and comparing large sets of protein or nucleotide sequences. Bioinformatics 22**:** 1658-1659.

Lushchak, V. I., 2011 Environmentally induced oxidative stress in aquatic animals. Aquat Toxicol 101**:** 13-30.

Manning, G., D. B. Whyte, R. Martinez, T. Hunter and S. Sudarsanam, 2002 The protein kinase complement of the human genome. Science 298**:** 1912-1934.

Nguyen, L. T., H. A. Schmidt, A. von Haeseler and B. Q. Minh, 2015 IQ-TREE: a fast and effective stochastic algorithm for estimating maximum-likelihood phylogenies. Mol Biol Evol 32**:** 268-274.

Opperdoes, F. R., A. Butenko, P. Flegontov, V. Yurchenko and J. Lukeš, 2016 Comparative metabolism of free-living *Bodo saltans* and parasitic trypanosomatids. J Eukaryot Microbiol 63**:** 657-678.

Pereira, C. A., G. D. Alonso, M. C. Paveto, A. Iribarren, M. L. Cabanas *et al.*, 2000 *Trypanosoma cruzi* arginine kinase characterization and cloning. A novel energetic pathway in protozoan parasites. J Biol Chem 275**:** 1495-1501.

Tashyreva, D., G. Prokopchuk, A. Yabuki, B. Kaur, D. Faktorová *et al.*, 2018 Phylogeny and morphology of new diplonemids from Japan. Protist 169**:** 158-179.

Wawrik, B., D. A. Bronk, S. E. Baer, L. Chi, M. Sun *et al.*, 2017 Bacterial utilization of creatine in seawater. Aquat Microb Ecol 80**:** 153-165.

Yabuki, A., G. Tanifuji, C. Kusaka, K. Takishita and K. Fujikura, 2016 Hyper-eccentric structural genes in the mitochondrial genome of the algal parasite *Hemistasia phaeocysticola*. Genome Biol Evol 8**:** 2870-2878.

Yazaki, E., S. A. Ishikawa, K. Kume, A. Kumagai, T. Kamaishi *et al.*, 2017 Global Kinetoplastea phylogeny inferred from a large-scale multigene alignment including parasitic species for better understanding transitions from a free-living to a parasitic lifestyle. Genes Genet Syst 92**:** 35-42.

17. Feeding strategy and food of *Diplonema papillatum*

LITERATURE REVIEW and DISCUSSION

The feeding strategy of diplonemids has been controversial for several decades, ranging from parasitic to predatory and saprophytic/scavenging (e.g., SCHUSTER *et al.* 1968; TRIEMER AND OTT 1990; VICKERMAN 2000; VON DER HEYDEN *et al.* 2004). Documented eukaryvors are, for example, *Hemistasia phaeocysticola* (ELBRÄCHTER *et al.* 1996; YABUKI AND TAME 2015) and *Rhynchopus coscinodiscivorus* (SCHNEPF 1994), both of which have been observed in the act of predating diatoms, whereas *R. euleeides*, for instance, consumes bacteria (ROY *et al.* 2007). In addition, a given species may feed on the food that is available, as observed for *D. japonicum* and *R. humris*, both of which are osmotrophs in a rich medium containing serum, but gradually switch to bacterivory under nutrient-poor conditions (PROKOPCHUK *et al.* 2022).

D. papillatum almost certainly has similar dietary plasticity as the two latter diplonemids. Under laboratory conditions, the type species feeds osmotrophically, but equipped, as are all diplonemids, with a cytostome and cytopharynx (PORTER 1973), it likely engulfs small microbes and particles in the wild. However, *D. papillatum* seems not to consume bacteria, because (i) enzymes for degrading peptidoglycan (murein), the main cell wall constituent of most bacteria, have not been detected in its genome (see **Supplementary Information**: Section 12. CAZyme-coding genes in *Diplonema papillatum*), and (ii) feeding experiments offering diverse live bacteria that otherwise allow profuse growth of, e.g., *D. japonicum* and *R. humris*, do not support measurable proliferation of *D. papillatum* (PROKOPCHUK *et al.* 2022). Interestingly, it has been suggested that the flagella of *Diplonema* (and *Rhynchopus*) serve for the recognition of suitable solid food rather than for locomotion because their axoneme may be disordered and because the flagella are too short for support and bending (SCHNEPF 1994).

It is generally assumed that *D. papillatum* scavenges on the debris of dead microeukaryotes or plants. This view is in line with the particular morphology of this protist, characterized by a narrow (~0.5–1 µm; see **Fig. 1**, main text) and rigid, microtubule-reinforced cytostome (PORTER 1973), limiting the type of microeukaryotic species that can be ingested intact by *D. papillatum*. More specifically, the upper size limit of eukaryotic prey that can be devoured whole by *D. papillatum* would be in the range of the smallest microalga described, *Ostreococcus tauri* (~0.7 µm wide and 1 µm long; COURTIES *et al.* 1994; CHRÉTIENNOT-DINET *et al.* 1995). In contrast to *D. papillatum*, the eukaryvorous heterotrophic euglenids *Peranema* and *Heteronema* (BREGLIA *et al.* 2013) and raptorial ciliates (VERNI AND GUALTIERI 1997) have highly expandable cytostomes, allowing ingestion of prey nearly as wide as the predator itself.

The extensive arsenal of CAZyme-encoding genes that we detected in the *D. papillatum* nuclear genome strongly suggests that in its natural habitat, this protist feeds on live eukaryotes after piercing and rupturing prey cells enzymatically. Cell particles can then be engulfed in the cytopharynx through the cytostome and broken down extracellularly into oligomeric compounds. The subsequent steps most likely follow classical endocytosis, by which the material is taken up inside the cell via food vacuoles, which in turn fuse with lysosomes in which the oligomers are broken down to amino acids, fatty acids, and other monomeric molecules, for use as an energy source and building blocks of the cell.

The feeding strategy mentioned above would allow *D. papillatum* to forage on eukaryotes from an extensive taxonomic range and of any physical size. Such a feeding behaviour, together with an adequate swimming capacity of trophic cells, could explain the ecological distribution of *D. papillatum* in eukaryote-rich coastal regions.

AUTHOR CONTRIBUTIONS

Writing, original draft – G.B.; **Writing, review & editing –** all co-authors.

REFERENCES

Breglia, S. A., N. Yubuki and B. S. Leander, 2013 Ultrastructure and molecular phylogenetic position of *Heteronema scaphurum*: a eukaryovorous euglenid with a cytoproct. J Eukaryot Microbiol 60**:** 107-120.

Chrétiennot-Dinet, M. J., C. Courties, A. Vaquer, J. Neveux, H. Claustre *et al.*, 1995 A new marine picoeucaryote: *Ostreococcus tauri* gen. et sp. nov. (Chlorophyta, Prasinophyceae). Phycologia 34**:** 285-292.

Courties, C., A. Vaquer, M. Troussellier, J. Lautier, M. J. Chrétiennot-Dinet *et al.*, 1994 Smallest eukaryotic organism. Nature 370**:** 255-255.

Elbrächter, M., E. Schnepf and I. Balzer, 1996 *Hemistasia phaeocysticola* (Scherffel) comb. nov., redescription of a free-living, marine, phagotrophic kinetoplastid flagellate. Arch. Protistenkd. 147**:** 125-136.

Porter, D., 1973 *Isonema papillatum* sp. n., a new colorless marine flagellate: a light- and electronmicroscopic study. The Journal of Protozoology 20**:** 351-356.
Prokopchuk, G., T. Korytář, V. Juricová, J. Majstorović, A. Horák *et al.*, 2022 Trophic flexibility of marine diplonemids - switching from osmotrophy to bacterivory. ISME J.
Roy, J., D. Faktorova, O. Benada, J. Lukes and G. Burger, 2007 Description of *Rhynchopus euleeides* n. sp. (Diplonemea), a free-living marine euglenozoan. J Eukaryot Microbiol 54**:** 137-145.
Schnepf, E., 1994 Light and electron microscopical observations in *Rhynchopus coscinodiscivorus* spec. nov., a colorless, phagotrophic euglenozoon with concealed flagella. Arch. Protistenkd. 144**:** 63-74.
Schuster, F. L., S. Goldstein and B. Hershenov, 1968 Ultrastructure of a flagellate, *Isonema nigricans* nov. gen. nov. sp., from a polluted marine habitat. Protistologica 4**:** 141-149.
Triemer, R. E., and D. W. Ott, 1990 Ultrastructure of *Diplonema ambulator* Larsen & Patterson (Euglenozoa) and its relationship to *Isonema*. Eur J Protistol 25**:** 316-320.
Verni, F., and P. Gualtieri, 1997 Feeding behaviour in ciliated protists. Micron 28**:** 487-504.
Vickerman, K., 2000 Diplonemids (Class: Diplonemea Cavalier Smith, 1993). pp. 1157-1159 in *An Illustrated Guide to the Protozoa*, edited by J. J. Lee, G. F. Leedale and P. Bradbury. Society of Protozoologists, Lawrence, Kansas, U.S.A.
von der Heyden, S., E. E. Chao, K. Vickerman and T. Cavalier-Smith, 2004 Ribosomal RNA phylogeny of bodonid and diplonemid flagellates and the evolution of Euglenozoa. J Eukaryot Microbiol 51**:** 402-416.
Yabuki, A., and A. Tame, 2015 Phylogeny and reclassification of *Hemistasia phaeocysticola* (Scherffel) Elbrächter & Schnepf, 1996. J Eukaryot Microbiol 62**:** 426-429.

18. Environmental distribution of *Diplonema papillatum*

INTRODUCTION

Diplonemids are ubiquitous members of microbial communities in marine environments, found in the top layer down to abyssal/hadal zones (>6,000 m below the surface), in both the pelagic (planktonic) and benthic (sediment) habitats, and in all geographic regions from the tropics to the poles.

Estimates of the **relative abundance** of diplonemids vary greatly depending on sampling methods, depth, and geographic location. For example, an *in situ* fluorescence hybridization survey of the tropical Atlantic Ocean estimated that diplonemids make up 1–3% of eukaryotes in the water column at 100–7,000 m below the surface (MORGAN-SMITH *et al.* 2013). Other studies used metabarcoding, a large-scale bio-assessment method that involves PCR amplification of hypervariable regions of 18S rDNA or rRNA in environmental samples. The V9 hypervariable region of the 18S rDNA is best-suited for targeting diplonemids. In contrast, the widely used V4 metabarcoding (e.g., MASSANA *et al.* 2015) often fails to detect members of this group because their V4 region exceeds 500 bp, which is above the typical insert length of high-throughput amplicon sequencing pipelines (FLEGONTOVA *et al.* 2016). Analysis of a V9 metabarcoding dataset obtained from 0.8–2,000 µm marine-plankton size fractions from the temperate and tropical oceans (DE VARGAS *et al.* 2015) revealed that diplonemids represented ~1% of all microeukaryotes in the photic zone (0–200 m depth), and reached a peak of as much as 14% in the upper portion of the aphotic zone, at a depth of ~750–1,000 m (FLEGONTOVA *et al.* 2020). A similar V9 metabarcoding survey led to the estimate that diplonemids make up, on average, 5% of microeukaryotes in the bathyal-hadal marine sediment (SCHOENLE *et al.* 2021).

Microbial diversity is best represented by the number of operational taxonomic units (**OTUs**) that groups organisms into bins, which are traditionally based on sequence similarity, but more recently on sequence clustering (MAHÉ *et al.* 2015). According to several (but not all) global V9 metabarcoding studies, the total number of diplonemid OTUs detected in the plankton of the world ocean amounts to tens of thousands, surpassing the numbers of OTUs for planktonic metazoans, stramenopiles, dinoflagellates, and rhizarians (FLEGONTOVA *et al.* 2020; CORDIER *et al.* 2022) (see also **Supplementary Table S24**).

The large majority of the biomass of diatoms, pelagophytes, dinoflagellates and some other protists is constituted by a relatively small number of OTUs (KEELING AND CAMPO 2017). In diplonemids, the bias in the OTU abundance profile is even more pronounced. For example, in the Tara Oceans dataset, the hundred most abundant OTUs (out of 45,197) represent more than 92% of all diplonemid reads (FLEGONTOVA *et al.* 2016). Planktonic diplonemids of the Eupelagonemidae clade account for >97% of all diplonemid OTUs and reads, whereas the other three clades—Diplonemidae, Hemistasiidae, and DSPD II—each account for only ~1% or even less (DE VARGAS *et al.* 2015; FLEGONTOVA *et al.* 2016; FLEGONTOVA *et al.* 2020). It should be noted that Eupelagonemidae and DSPD II OTUs occur predominantly in the mesopelagic zone (at 200–1,000 m depth). In contrast, Diplonemidae and Hemistasiidae OTUs are primarily found in the surface zone (FLEGONTOVA *et al.* 2016).

Diplonemids living in freshwater are an exception. They have been detected in the plankton of lakes in Sweden (SKUJA 1948), Japan, the Czech Republic, and Switzerland (MUKHERJEE *et al.* 2020), and in the plankton and sediments of Lake Baikal (YI *et al.* 2017; DAVID *et al.* 2021; REBOUL *et al.* 2021). Freshwater diplonemids occur at a very low abundance (<1% of microeukaryotes), and their diversity is limited. Samples collected from the geographically most distant sites all fall within the Diplonemidae clade, consistent with Skuja's hypothesis of a recent habitat transition from a marine to a freshwater environment (MUKHERJEE *et al.* 2020; DAVID *et al.* 2021).

The true diversity of diplonemids in the environment has been a matter of controversy due to genomic variability of the 18S rRNA gene sequences found in single-cell genomes of ten distinct marine planktonic diplonemids (MUKHERJEE *et al.* 2020). However, such intra-genomic variability is not unique to diplonemids and probably affects nearly all estimates of diversity relying on high-throughput sequencing of marker-gene amplicons. For instance, not only multiple distinct sequences, but multiple clusters of sequences (OTUs of the V9 18S rRNA region) were found in mono-species ciliate cultures (FORSTER *et al*. 2019). The same is true for single radiolarian cells, where multiple OTUs of the V9 and V4 18S rRNA regions were found (DECELLE *et al.* 2014). Metabarcoding approaches can overestimate the diversity due to various causes: sequencing errors and PCR artefacts such as chimera formation, as well as intra-genomic and intra-population sequence variability (BÁLINT *et al*. 2016; SANTOFERRARA *et al*. 2020). MUKHERJEE *et al.* 2020 have not demonstrated that diplonemids are more prone to overestimation than other eukaryotes, and we believe that interpreting *relative* diversity of protist groups is possible, even though absolute diversity estimates are currently unreliable.

While we have a good understanding of the environmental and geographical distribution of the major diplonemid groups, the same parameters for the type species *Diplonema papillatum* are not known. Therefore, we searched available

barcoding resources for the presence of sequences from *D. papillatum*. To leverage information not only from V9, but also from the more frequent V4 datasets, we analyzed forward and reverse sequencing reads from the latter separately, allowing incomplete coverage of the V4 region.

Supplementary Table S24. Abundance of OTUs from selected taxa in the Tara project datasets [a].

	OTU abundance bins (reads per million) [b]								
Taxon	≥10,000	≥1,000–<10,000	≥100–<1,000	≥10–<100	≥1–<10	≥0.1–<1	≥0.01–<0.1	<0.01	Total OTUs
Diplonemea–Diplonemidae	0	0	0	1	9	16	81	94	201
Diplonemea–Hemistasiidae	0	0	0	5	7	61	83	58	214
Diplonemea–Eupelagonemidae	0	8	23	14	16	82	3,074	**8,087**	11,304
Diplonemea–DSPDII	0	0	0	3	0	1	14	**38**	56
Diplonemea	0	8	23	23	32	160	3,266	**8,277**	11,775
Kinetoplastea	0	0	4	7	10	36	50	60	167
Euglenida	0	0	1	0	7	11	49	59	127
Heterolobosea	0	0	0	0	5	5	33	24	67
Stramenopiles	0	20	107	208	293	567	1,745	2,870	5,810
Dinoflagellata	1	22	221	724	1,158	1,696	5,254	**10,865**	19,941
Ciliophora	0	4	23	90	164	263	563	781	1,888
Rhizaria	3	20	94	208	354	998	3,435	**5,641**	10,753
Haptophyta	0	1	15	34	46	62	128	228	514

[a] For this analysis, the Tara Oceans and Tara Arctic datasets were combined (IBARBALZ *et al.* 2019). In the Tara Oceans dataset, diplonemids emerged as the most OUT-rich taxon, whereas in the combined Tara datasets, diplonemids rank at second place, behind dinoflagellates.
[b] OTUs were defined by the SWARM algorithm (MAHÉ *et al.* 2015). Sequences sharing >90% identity to a particular reference sequence were merged for consistency with the 90% threshold used for *D. papillatum* sequences that form a single OTU. OTUs of <0.01 abundance that collectively includes>50% of all OTUs of a given taxon are highlighted in blue bold.

RESULTS and DISCUSSION

To gain a picture of the global and ecosystemic distribution of *D. papillatum*, we searched for the corresponding sequences in the global V9 metabarcoding datasets ('Tara Oceans' and 'Tara Arctic') generated by the Tara project (DE VARGAS *et al.* 2015; IBARBALZ *et al.* 2019). *D. papillatum* sequences were found in four out of 970 planktonic samples. All four samples originated from surface water: one collected off the Mediterranean Spanish coast (four *D. papillatum* reads; latitude 41.6686, longitude 2.7996) and three from coastal waters in the reef surrounding the Gambier Islands in the Pacific Ocean (29 reads; latitude cca. −23, longitude cca. −135; **Supplementary Figure S26**). The collection sites had a relatively high water temperature (23–25 ºC), a salinity between 36.46 and 36.52, an oxygen concentration between 201 and 207 µmol/L, and a low chlorophyll A concentration (0.01–0.07), a measure of the amount of suspended phytoplankton. With only 1 to 25 reads from *D. papillatum* per 1–2 million total reads, the abundance in these samples was very low. Since no *D. papillatum*-representing sequence was found in Tara samples from the open ocean, we prioritized, for further analyses, the datasets that include coastal samples.

Our search in the V4 and V9 metabarcoding datasets 'Ocean Sampling Day 2014' (KOPF *et al.* 2015) and 'Helgoland Roads 2016'(KÄSE *et al.* 2020), which comprise coastal samples, returned a small number of *D. papillatum* sequences. Specifically, among 31 metabarcoding samples of the Ocean Sampling Day project, nine contained 1–13 V4 and/or V9 reads per sample (4 reads on average, per 0.3-0.4 million total reads) from *D. papillatum*. The corresponding samples were collected at a depth of 0–10 m under the surface at temperatures ranging from 11 °C to 30 °C and originated from geographically distant locations off the coast of Japan, the East and West coasts of North America, the East coast of South America, and the North Sea coast (**Supplementary Figure S26**). Among 287 V4-metabarcoding samples collected during various seasons at the Helgoland Roads Long-Term Ecological Research site (Helgoland Islands coast, North Sea, depth of 1 m; latitude 54.1838333, longitude 7.9; temperature 6–13 °C) (KÄSE *et al.* 2020), *D. papillatum* sequences were identified in five samples (1–10 *D. papillatum* reads per sample, per 0.1-3 million total reads).

D. papillatum sequences were absent from the two other V4 metabarcoding datasets collected from coastal waters of Europe (MASSANA *et al.* 2015), Brittany (France) and Senegal (RAMOND *et al.* 2019). The failure to detect *D. papillatum* sequences in these latter data sets might be due to the relatively low number of total reads produced by these two studies (127–30,296 and 16,611–185,889 reads per sample, respectively).

We compared the relative abundance of V9 reads from *D. papillatum* with that of other diplonemids in Tara Oceans and Tara Arctic datasets (**Supplementary Table S25**). The table shows that *Hemistasia phaeocysticola* and the Diplonemidae isolate YPF1523 reached a total abundance of ~70, and more reads per million. Otherwise, the abundance was generally below 1 read per million, with *D. papillatum* being among the least abundant diplonemid species (~1–10 reads per million in samples in which it was detected and ~0.04 reads per million over all samples; **Supplementary Table S25, Additional File 7** envirDistr). To summarize, while apparently quite rare, *D. papillatum* seems to be broadly distributed in temperate coastal surface waters of the world ocean (**Supplementary Figure S26**).

We note, however, that the barcode-derived quantification of diplonemids remains rather approximative. In eukaryotes, the rDNA loci copy number can vary substantially based on environmental or cultivation cues within a species, but also between species irrespective of their degree of relatedness, thus introducing biases into abundance estimates (LAVRINIENKO *et al*. 2021). We currently lack information on the expansion of the rDNA loci in diplonemids other than *D. papillatum*. Taken together, the conclusion about the relative rarity of *D. papillatum* compared to other diplonemids rests upon the assumption that the rDNA copy number is both similar and relatively constant across diplonemids.

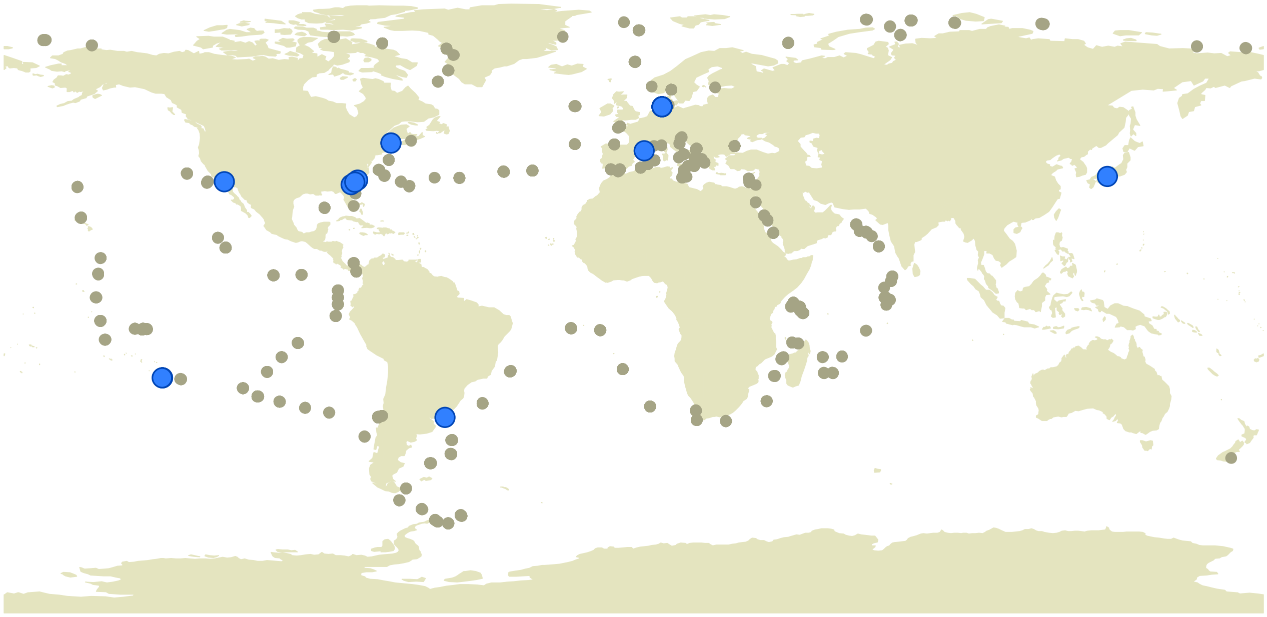

Supplementary Figure S26. **Oceanic distribution of *D. papillatum*.** The world map shows the distribution of sampling locations from the three datasets in which *D. papillatum* was detected, namely the Tara project, 'Ocean Sampling Day 2014', and 'Helgoland Roads 2016' (beige dots). Sites, where *D. papillatum*-representing OTUs were detected, are highlighted in blue (see the **Additional File 7** envirDistr for details).

Supplementary Table S25. **Abundance of selected diplonemids in the combined Tara project datasets.**

Family	Species	Abundance (V9 reads per million)[a]
Diplonemidae	*Diplonema papillatum*	0.04
	Diplonema aggregatum	0.04
	Diplonema japonicum	0.06
	Diplonema sp. ATCC 50232	0.10
	Rhynchopus humris	0.40
	Rhynchopus serpens	1.17
	Rhynchopus sp. SH-2004-I	1.26
	Flectonema neradi	<0.01
	Lacrimia lanifica	2.79
Hemistasiidae	*Hemistasia phaeocysticola*	69.13
Unknown	Diplonemidae sp. YPF1523	78.08

[a] Number of reads across all samples. In the subset of samples that contain reads from *D. papillatum*, the number of these reads per sample is 1–10.

METHODS

We searched for *D. papillatum* sequences in the datasets from six studies that reported 18S rDNA V4 or V9 metabarcoding reads from marine plankton (**Additional File 7** envirDistr; (DE VARGAS *et al.* 2015; KOPF *et al.* 2015; MASSANA *et al.* 2015; IBARBALZ *et al.* 2019; RAMOND *et al.* 2019; KÄSE *et al.* 2020). Amplification primers were removed from raw reads using *cutadapt* v1.16 (MARTIN 2001). The V9 and V4 regions of *D. papillatum* are 128 and 530 bp long, respectively. To simplify sequence analysis, we merged paired V9 reads using *bbmerge* (BBMap package v38.9) (BUSHNELL *et al.* 2017). The V4 reads could only be merged for a single study because in this case they were long enough to cover the whole V4 region (KÄSE *et al.* 2020). For the remaining studies, forward and reverse V4 reads were analyzed separately. Taxonomic annotation of the processed reads was performed using a global alignment tool *ggsearch* (FASTA package v3.5) (PEARSON 2000). As a reference database we used all 525 diplonemid 18S rRNA sequences from the EukRef-excavates collection (KOLISKO *et al.* 2020), including two *D. papillatum* sequences. To account for slightly different amplification primers used across the studies, we processed the database sequences so that they specifically corresponded to the amplified region in each study. Lastly, since the V9 and V4 regions are highly variable across species and can even vary within a single genome, we established a percent-identity cutoff for *ggsearch* hits. We compared V4 and V9 regions of *D. papillatum* and EukRef diplonemid sequences. We also compared all V4 and V9 sequence variants found in the *D. papillatum* genome assembly to the sequence deposited in NCBI (GenBank accession number KF633466). Within the *D. papillatum* genome assembly multiple copies of the V4 and V9 regions exist (as delimited by the amplification primers), but the identity of sequences from other diplonemids never exceeded 90%; therefore, we chose 90% as the identity cutoff. Relative abundance in reads per million (**Supplementary Tables S24, S25**) was calculated using a combined dataset of eukaryotic metabarcodes from Tara Oceans and Tara Arctic (DE VARGAS *et al.* 2015; IBARBALZ *et al.* 2019).

AUTHOR CONTRIBUTIONS

Conceptualization, Data curation, Formal analysis, Investigation, Writing, original draft – O.F.; **Visualization –** M.V.; **Writing, review & editing –** all co-authors.

REFERENCES

Bálint, M., M. Bahram, A. M. Eren, K. Faust, J. A. Fuhrman *et al.*, 2016 Millions of reads, thousands of taxa: microbial community structure and associations analyzed via marker genes. FEMS Microbiol Rev. 40**:** 686-700.

Bushnell, B., J. Rood and E. Singer, 2017 BBMerge - Accurate paired shotgun read merging via overlap. PLoS One 12**:** e0185056.

Cordier, T., I. B. Angeles, N. Henry, F. Lejzerowicz, C. Berney *et al.*, 2022 Patterns of eukaryotic diversity from the surface to the deep-ocean sediment. Sci Adv 8**:** eabj9309.

David, G. M., D. Moreira, G. Reboul, N. V. Annenkova, L. J. Galindo *et al.*, 2021 Environmental drivers of plankton protist communities along latitudinal and vertical gradients in the oldest and deepest freshwater lake. Environ Microbiol 23**:** 1436-1451.

de Vargas, C., S. Audic, N. Henry, J. Decelle, F. Mahe *et al.*, 2015 Ocean plankton. Eukaryotic plankton diversity in the sunlit ocean. Science 348**:** 1261605.

Decelle, J., S. Romac, E. Sasaki, F. Not and F. Mahé, 2014 Intracellular diversity of the V4 and V9 regions of the 18S rRNA in marine protists (radiolarians) assessed by high-throughput sequencing, PLoS One 9**:** e104297.

Flegontova, O., P. Flegontov, P. A. C. Londoño, W. Walczowski, D. Šantić *et al.*, 2020 Environmental determinants of the distribution of planktonic diplonemids and kinetoplastids in the oceans. Environ Microbiol 22**:** 4014-4031.

Flegontova, O., P. Flegontov, S. Malviya, S. Audic, P. Wincker *et al.*, 2016 Extreme diversity of diplonemid eukaryotes in the ocean. Curr Biol 26**:** 3060-3065.

Forster, D., G. Lentendu, S. Filker, E. Dubois, T.A. Wilding *et al*., 2019 Improving eDNA-based protist diversity assessments using networks of amplicon sequence variants, Environ Microbiol 21**:** 4109-4124.

Ibarbalz, F. M., N. Henry, M. C. Brandão, S. Martini, G. Busseni *et al.*, 2019 Global trends in marine plankton diversity across kingdoms of life. Cell 179**:** 1084-1097.e1021.

Käse, L., A. C. Kraberg, K. Metfies, S. Neuhaus, P. A. A. Sprong *et al.*, 2020 Rapid succession drives spring community dynamics of small protists at Helgoland Roads, North Sea. J Plankton Res 42**:** 305-319.

Keeling, P. J., and J. D. Campo, 2017 Marine protists are not just big bacteria. Curr Biol 27**:** R541-r549.

Kolisko, M., O. Flegontova, A. Karnkowska, G. Lax, J. M. Maritz *et al.*, 2020 EukRef-excavates: seven curated SSU ribosomal RNA gene databases. Database (Oxford) 2020.

Kopf, A., M. Bicak, R. Kottmann, J. Schnetzer, I. Kostadinov *et al.*, 2015 The ocean sampling day consortium. Gigascience 4**:** 27.

Lavrinienko, A., T. Jernfors, J. J. Koskimäki, A. M. Pirttilä and P. C. Watts, 2021 Does intraspecific variation in rDNA copy number affect analysis of microbial communities? Trends Microbiol. 29**:** 19-27.

Mahé, F., T. Rognes, C. Quince, C. de Vargas and M. Dunthorn, 2015 Swarm v2: highly-scalable and high-resolution amplicon clustering. PeerJ 3**:** e1420.

Martin, M., 2001 Cutadapt removes adapter sequences from high-throughput sequencing reads. EMBnet.journal 17.

Massana, R., A. Gobet, S. Audic, D. Bass, L. Bittner *et al.*, 2015 Marine protist diversity in European coastal waters and sediments as revealed by high-throughput sequencing. Environ Microbiol 17**:** 4035-4049.

Morgan-Smith, D., M. A. Clouse, G. J. Herndl and A. B. Bochdansky, 2013 Diversity and distribution of microbial eukaryotes in the deep tropical and subtropical North Atlantic Ocean. Deep Sea Research Part I: Oceanographic Research Papers 78**:** 58-69.

Mukherjee, I., M. M. Salcher, A. Andrei, V. S. Kavagutti, T. Shabarova *et al.*, 2020 A freshwater radiation of diplonemids. Environ Microbiol 22**:** 4658-4668.

Pearson, W. R., 2000 Flexible sequence similarity searching with the FASTA3 program package. Methods Mol Biol 132**:** 185-219.

Ramond, P., M. Sourisseau, N. Simon, S. Romac, S. Schmitt *et al.*, 2019 Coupling between taxonomic and functional diversity in protistan coastal communities. Environ Microbiol 21**:** 730-749.

Reboul, G., D. Moreira, N. V. Annenkova, P. Bertolino, K. E. Vershinin *et al.*, 2021 Marine signature taxa and core microbial community stability along latitudinal and vertical gradients in sediments of the deepest freshwater lake. Isme j 15**:** 3412-3417.

Santoferrara, L., F. Burki, S. Filker, R. Logares, M. Dunthorn *et al.*, 2020 Perspectives from ten years of protist studies by high-throughput metabarcoding. J Eukaryot Microbiol. 67**:** 612-622.

Schoenle, A., M. Hohlfeld, K. Hermanns, F. Mahé, C. de Vargas *et al.*, 2021 High and specific diversity of protists in the deep-sea basins dominated by diplonemids, kinetoplastids, ciliates and foraminiferans. Commun Biol 4**:** 501.

Yi, Z., C. Berney, H. Hartikainen, S. Mahamdallie, M. Gardner *et al.*, 2017 High-throughput sequencing of microbial eukaryotes in Lake Baikal reveals ecologically differentiated communities and novel evolutionary radiations. FEMS Microbiol Ecol 93.

19. DNA and RNA preparation for high-throughput sequencing

MATERIALS AND METHODS

Strains and culture conditions

Diplonema papillatum (ATCC 50162) was initially obtained from the American Type Culture Collection (ATCC) and cultivated axenically without shaking at 15–22 °C in liquid saline medium (OS) containing 33 g/L Instant Ocean sea salt and supplemented with 1% (v/v) horse serum as described earlier (VALACH *et al.* 2014). For extended cultivations, chloramphenicol (40 mg/L) was added to prevent bacterial contamination.

For nucleic acid extraction and sequencing at Genome Quebec (Illumina DNA- and RNA-Seq, PacBio DNA-Seq), the protist was cultured for ~1 week, yielding a total of 3×10^8 exponentially dividing cells, which were harvested by centrifugation of the culture at 3000×g for 10 min, then washed with ice-cold buffer (0.65 M sorbitol, 20 mM Tris, pH 7.5, 5 mM EDTA), and disrupted by nitrogen decompression at 600 psi (Parr Instruments Company). Alternatively, for nucleic acids extractions for their sequencing at Eurofins (Illumina DNA-Seq), Takara Bio (PacBio DNA-Seq), and Novogene (Illumina RNA-Seq), the organism was cultivated at 25 °C in an ATCC 1532 medium supplemented with 1% (v/v) horse serum and 0.1% (w/v) tryptone (MORALES *et al.* 2016).

Extraction of nucleic acids

Total cellular DNA was isolated from the disintegrated cells using the columns Genomic-tip 100/G (Qiagen). For long-read DNA sequencing, DNA was further size-selected to avoid copious mitochondrial DNA (6–7 kbp circular molecules) by employing the BluePippin instrument (Sage Science) with a 20-kbp cut-off. Total cellular RNA was extracted using a homemade Trizol substitute (RODRIGUEZ-EZPELETA *et al.* 2009), and residual DNA was removed by digestion with an RNase-free DNase followed by another round of Trizol-substitute extraction. After denaturation at 72 °C for 2 min and subsequent chilling on ice, poly(A) RNA was enriched by a passage through oligo(dT)-cellulose (Amersham). DNA and RNA samples were submitted for sequencing to the technology platforms Genome Quebec Innovation Center, Montreal. Alternatively, a commercial TRIZOL reagent (Invitrogen) and GenElute columns were used, and the resulting total RNA was submitted for mRNA enrichment and sequencing to Novogene.

A total of 10 libraries were constructed according to manufacturers' recommendations and sequenced with the Illumina or PacBio technologies. Information on library preparation and genome and transcriptome sequencing methodologies are described in the **Supplementary Information**: Section 2. Assembly and annotation of the nuclear genome and transcriptome of *Diplonema papillatum*.

REFERENCES

Morales, J., M. Hashimoto, T. A. Williams, H. Hirawake-Mogi, T. Makiuchi *et al.*, 2016 Differential remodelling of peroxisome function underpins the environmental and metabolic adaptability of diplonemids and kinetoplastids. Proc Biol Sci 283**:** 20160520.

Rodriguez-Ezpeleta, N., S. Teijeiro, L. Forget, G. Burger and B. F. Lang, 2009 Construction of cDNA libraries: focus on protists and fungi. Methods Mol Biol 533**:** 33-47.

Valach, M., S. Moreira, G. N. Kiethega and G. Burger, 2014 Trans-splicing and RNA editing of LSU rRNA in *Diplonema* mitochondria. Nucleic Acids Res 42**:** 2660-2672.
