# Supplementary material for: Recent expansion of metabolic versatility in Diplonema papillatum, the model species of a highly speciose group of marine eukaryotes
Source: BMC Biol. 2023 May 4;21:99. doi: 10.1186/s12915-023-01563-9 (PMC10161547; doi:10.1186/s12915-023-01563-9)

A [gel, left panel]

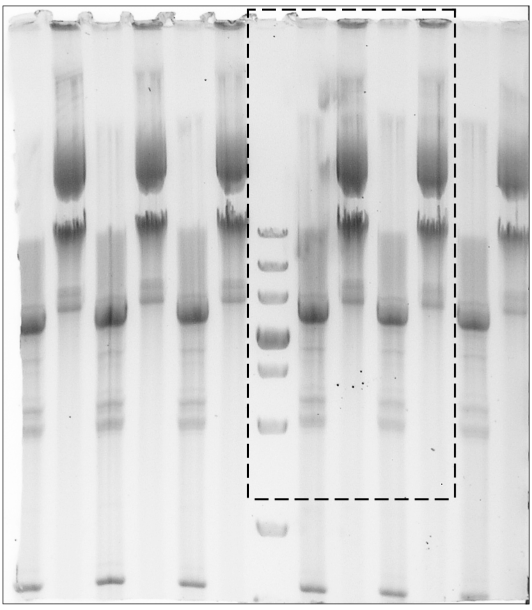

A [blot, right panel]

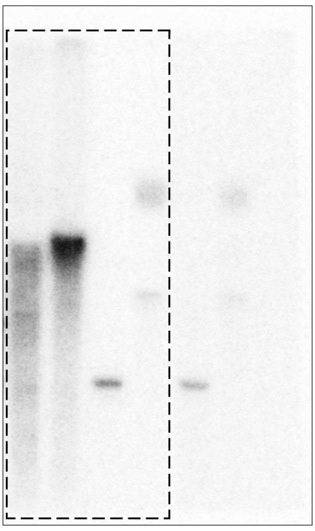

B [gel, left panel]

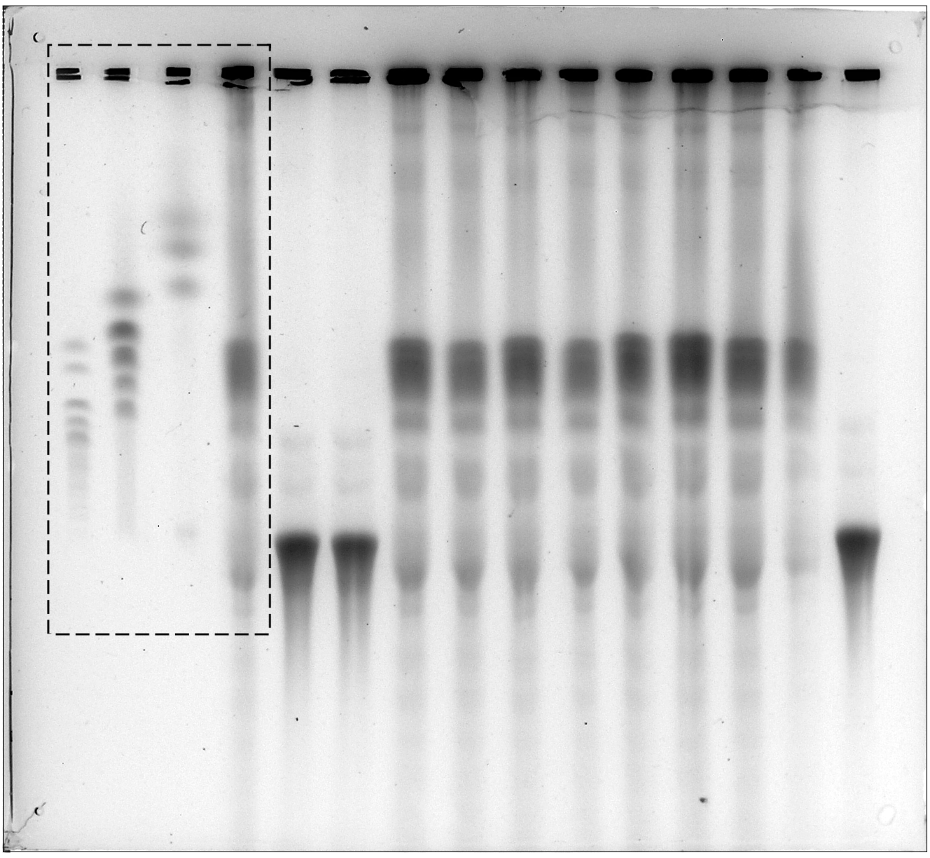

B [blot, right panel]

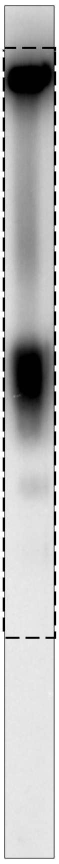

C [gel, left panel]

C [gel, middle panel]

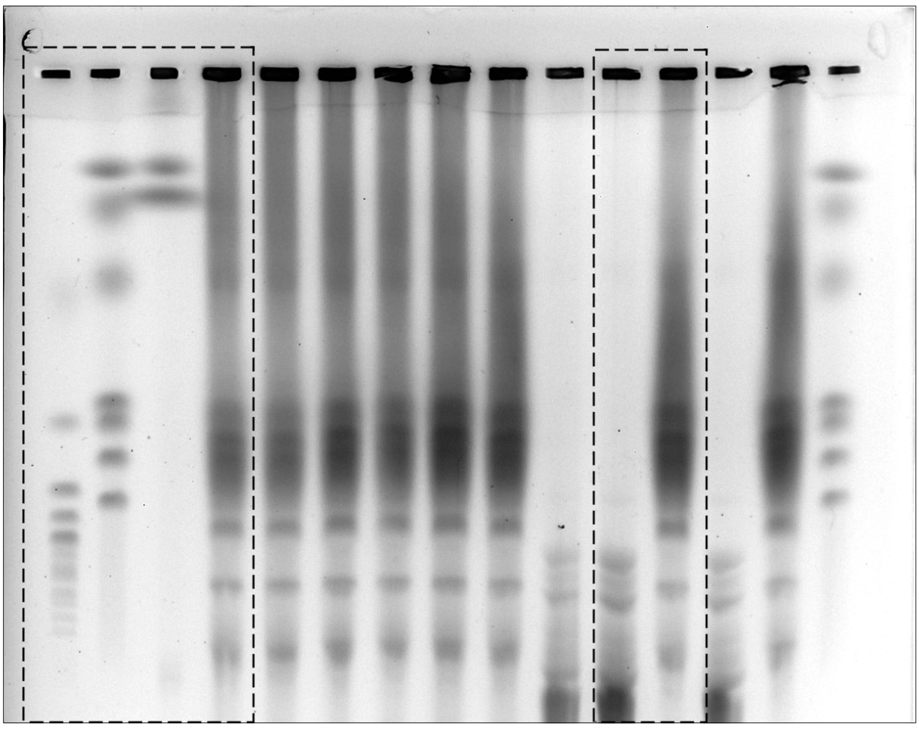

C [blot, right panel]

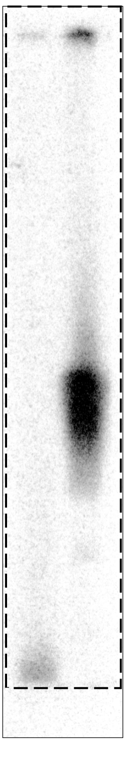

Supplement: Supplementary file 8 — Additional file 8. Uncropped gels and blots shown in the Supplementary Figure S2. See also Additional file 1: Section 1. Physical structure and size of the D. papillatum nuclear genome. [file 12915_2023_1563_MOESM8_ESM.pdf]
